# Synthesis of a Novel Series of Cu(I) Complexes Bearing Alkylated 1,3,5-Triaza-7-phosphaadamantane as Homogeneous and Carbon-Supported Catalysts for the Synthesis of 1- and 2-Substituted-1,2,3-triazoles

**DOI:** 10.3390/nano11102702

**Published:** 2021-10-13

**Authors:** Ivy L. Librando, Abdallah G. Mahmoud, Sónia A. C. Carabineiro, M. Fátima C. Guedes da Silva, Carlos F. G. C. Geraldes, Armando J. L. Pombeiro

**Affiliations:** 1 Centro de Química Estrutural, Instituto Superior Técnico, Universidade de Lisboa, Av. Rovisco Pais, 1049-001 Lisboa, Portugal; ivy.librando@tecnico.ulisboa.pt (I.L.L.); sonia.carabineiro@fct.unl.pt (S.A.C.C.); fatima.guedes@tecnico.ulisboa.pt (M.F.C.G.d.S.); pombeiro@tecnico.ulisboa.pt (A.J.L.P.); 2 Department of Chemistry, Faculty of Science, Helwan University, Ain Helwan, Cairo 11795, Egypt; 3 LAQV-REQUIMTE, Department of Chemistry, NOVA School of Science and Technology, Universidade NOVA de Lisboa, 2829-516 Caparica, Portugal; 4 Coimbra Chemistry Center, University of Coimbra, Rua Larga Largo D. Dinis, 3004-535 Coimbra, Portugal; geraldes@uc.pt; 5 Department of Life Sciences, Faculty of Science and Technology, Calçada Martim de Freitas, 3000-393 Coimbra, Portugal; 6 Research Institute of Chemistry, Peoples’ Friendship University of Russia (RUDN University), 6 Miklukho-Maklaya Street, 117198 Moscow, Russia

**Keywords:** carbon nanotubes, copper complexes, P-ligands, PTA, click chemistry, Azide-Alkyne cycloaddition, supported catalysis

## Abstract

The *N*-alkylation of 1,3,5-triaza-7-phosphaadamantane (PTA) with *ortho-*, *meta-* and *para-*substituted nitrobenzyl bromide under mild conditions afforded three hydrophilic PTA ammonium salts, which were used to obtain a new set of seven water-soluble copper(I) complexes. The new compounds were fully characterized and their catalytic activity was investigated for the low power microwave assisted one-pot azide–alkyne cycloaddition reaction in homogeneous aqueous medium to obtain disubstituted 1,2,3-triazoles. The most active catalysts were immobilized on activated carbon (AC), multi-walled carbon nanotubes (CNT), as well as surface functionalized AC and CNT, with the most efficient support being the CNT treated with nitric acid and NaOH. In the presence of the immobilized catalyst, several 1,4-disubstituted-1,2,3-triazoles were obtained from the reaction of terminal alkynes, organic halides and sodium azide in moderate yields up to 80%. Furthermore, the catalyzed reaction of terminal alkynes, formaldehyde and sodium azide afforded 2-hydroxymethyl-2*H*-1,2,3-triazoles in high yields up to 99%. The immobilized catalyst can be recovered and recycled through simple workup steps and reused up to five consecutive cycles without a marked loss in activity. The described catalytic systems proceed with a broad substrate scope, under microwave irradiation in aqueous medium and according to “click rules”.

## 1. Introduction

**Scheme 1:**
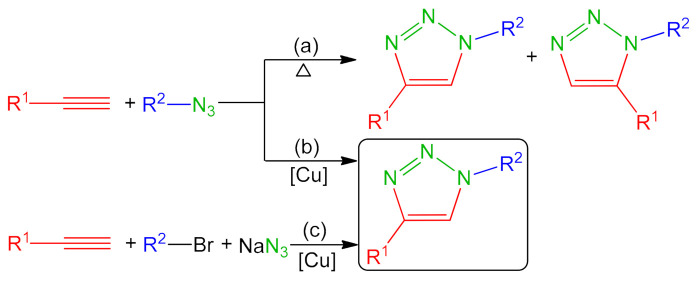
Huisgen’s 1,3-dipolar cycloaddition reaction to synthesize 1,2,3-triazoles (**a**) under classical conventional conditions, (**b**) via CuAAC reaction, and (**c**) via three components CuAAC reaction.

Click chemistry is a conception of preparative organic chemistry coined by Sharpless and co-workers at the beginning of this century, which is of paramount importance in modern chemistry [1]. Since its discovery in 2002 [2,3], the copper(I) catalysed azide-alkyne cycloaddition (CuAAC) for the regioselective synthesis of 1,4-disubstituted-1,2,3-triazoles constitutes the most useful click reaction considering the dramatic acceleration of the Huisgen’s 1,3-dipolar cycloaddition of organic azide and substituted alkyne in the presence of a copper(I) catalyst under mild conditions (Scheme 1). Another route of three components CuAAC reaction has been developed to avoid the difficulties associated with handling an organic azide [4,5], where it is formed in situ from the reaction of organic halide and sodium azide (Scheme 1c) [6]. Due to the high importance of 1,2,3-triazoles as components in pharmacologically active compounds, organic and inorganic polymers and materials, CuAAC has had a marked influence in several chemical applications [7,8,9].

Several studies indicated the positive influence of certain ligands to improve the efficiency of CuAAC, which are able to stabilize the Cu(I) catalyst and prevent its oxidation and disproportionation during the catalytic cycle [10,11,12]. In this context, several well-defined copper complexes bearing phosphines [13], polydentate amines [14], iminophosphoranes [15,16], N-heterocyclic carbenes [17,18,19] tris(triazolyl) methanol [20,21], tris(pyrazolyl) methanes [22], and arylhydrazones [23] as ligands have been applied as catalysts for the CuAAC reaction. The choice of ligands in the coordination sphere of the copper centre is very important to provide the obtained metal complex with specific characteristics such as stability and hydrophilicity. The hydrosoluble phosphanes of the type 1,3,5-triaza-7-phosphaadamantane (PTA) and its derivatives are among the most useful water-soluble phosphines in the field of organometallic chemistry and catalysis [24,25]. As a result of our interest in copper complexes based on PTA and its derivatives, we have recently reported water-soluble copper complexes with a high catalytic activity towards the CuAAC reaction in aqueous media [12,26,27,28]. Utilization of hydrosoluble copper complexes for CuAAC offers the possibility of being easily separable from the obtained organic triazoles by simple water washing to provide the product with high purity [12,23,26]. This last privilege fulfils one of the most important click criteria, the involvement of straightforward procedures for product isolation [1].

On the other hand, in contrast to 1-substituted-1,2,3-triazoles, there is no established universal approach to obtain the 2-substituted-1,2,3-triazoles and several synthetic protocols have been developed for that purpose [29,30]. The most known protocols to obtain 2*H*-1,2,3-triazoles are (i) alkylation of N*H*-1,2,3-triazoles using electrophilic agents, which is not regioselective and leads to the formation of an isomeric mixture of products [31], (ii) various cyclizations of hydrazones, which are limited to the synthesis of 2-aryl-substituted 1,2,3-triazoles [32], and (iii) three-component Pd-[33] or Pb/Cu-[34] catalytic systems, which involve the formation of a 1-allylpalladium-1,2,3-triazole complex intermediate that subsequently rearranges to the 2*H* isomer. Upon hydrolysis of 1*H*- and 2*H*-alkylated-4,5-dibromo-1,2,3-triazole mixture, the formation of the 2-hydroxymethyl isomer was observed [35], which suggests that it is thermodynamically more stable than its 1*-*substituted isomer. Such stability depends on the dynamic equilibrium between the *N*-hydroxymethyl triazole, formaldehyde, and the N*H*-triazole [36,37].

**Scheme 2:**
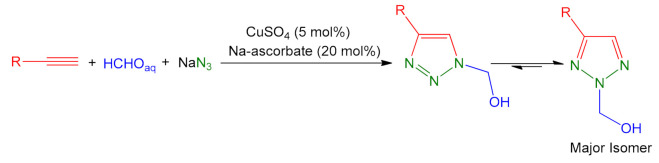
Synthesis of 2-hydroxymethyl-2*H*-1,2,3-triazoles via catalytic CuAAC reaction [38].

In 2008, Kalisiak and co-workers reported a three-component (terminal alkyne, formaldehyde and sodium azide) CuAAC reaction to afford the 2*-*hydroxymethyl-2*H-*1,2,3-triazoles (Scheme 2) [38], which are versatile precursors to a broad variety of 2*H-*substituted-1,2,3-triazoles and N*H*-triazoles, and can be readily converted to polyfunctional molecules. A sequence begins with a terminal alkyne, sodium azide and formalin to afford the regioisomeric mixture of 1- and 2-hydroxymethyl-1,2,3-triazole products, with the latter being the major isomer (Scheme 2) [38]. The copper(I) active species required to catalyze the reaction was generated in situ by mixing copper(II) sulfate salt with sodium ascorbate as a reducing agent [38]. To our knowledge, other catalytic well-defined Cu-complexes for CuAAC reaction to afford 2*-*substituted*-*1,2,3-triazoles have not been yet investigated. Since 2H-1,2,3-triazoles are being applied in many fields [39,40], the preparation of a new efficient and recyclable catalyst to obtain 2-hydroxymethyl-1,2,3-triazole constitutes one of the main objectives of this study.

In an effort to expand the existing library of water-soluble phosphanes, herein we describe the synthesis and characterization of new hydrosoluble *N*-alkylated derivatives of PTA incorporating *o-*, *m-* and *p-*substituted nitrobenzyls. The coordination properties of the obtained phosphane molecules have been studied towards copper(I) to produce a novel set of hydrophilic Cu(I) complexes. In continuation of our contribution to the field of click chemistry by means of homogeneous [12,22,23,26,41] and carbon-supported catalysts [28], the three-component approach of the CuAAC reaction for microwave-assisted synthesis of 1,4-disubstituted- and 2*-*hydroxymethyl-1,2,3-triazoles in aqueous medium is tackled using the new Cu(I) complexes as catalysts in a homogenous system, and as heterogenized catalysts supported on activated carbon (AC) and multi-walled carbon nanotubes (CNT), thus aiming also a comparison between both catalysis methods and these types of carbon supports.

## 2. Materials and Methods

### 2.1. General Procedures

Reagents and solvents were obtained from commercial sources and used without further purification. All reaction procedures were performed in air. Infrared spectra (4000–400 cm^−1^) were recorded on a Vertex 70 (Bruker Corporation, Ettlingen, Germany) instrument in KBr pellets. ^1^H, ^13^C{^1^H}, ^31^P{^1^H}, DEPT and 2D (COSY and HSQC) NMR spectra were obtained using Bruker Avance (Bruker, Billerica, MA, USA) 300 MHz and 400 MHz spectrometers at ambient temperature. The chemical shifts were reported in ppm using tetramethylsilane as an internal reference. Electrospray mass (ESI-MS) spectra were obtained on a Varian 500-MS LC Ion Trap Mass Spectrometer (Agilent Technologies, Santa Clara, CA, USA) equipped with an electrospray ion source. The compounds were observed and reported in the positive mode (capillary voltage = 80–105 V). The microwave irradiation was done using an Anton Paar Monowave 300 microwave reactor (Anton Paar GmbH, Graz, Austria). The loading of Cu on the carbon materials was determined by Inductively Coupled Plasma (ICP) at the Laboratório de Análises of Instituto Superior Técnico and at ReQuimTe Laboratory of Análysis. The carbon materials were previously degassed at 150 °C for 48 h and were characterized by N_2_ adsorption at 77 K in Micrometrics ASAP 2060 gas sorption instrument (Hiden Isochema, Warrington, UK). The multi-point Brunauer–Emmett–Teller (BET) theory and the t-plot method procedure were used for estimating the total and the external surface areas of the materials.

### 2.2. Synthesis of PTA Ammonium Salts 1–3

The synthesis of PTA ammonium salts was stemmed from a procedure described in literature for PTA alkylation [42]. To an acetone solution (350 mL) of PTA (9.55 mmol, 1.5 g, 1 equiv.), a solution of the nitrobenzyl bromide (2-nitrobenzyl bromide for **1**, 3-nitrobenzyl bromide for **2** or 4-nitrobenzyl bromide for **3**; 9.55 mmol, 2.06 g, 1 equiv.) in acetone (50 mL) was added dropwise with vigorous stirring under reflux. After stirring the mixture for additional 1 h, the obtained white solids were isolated by filtration, washed with acetone and dried in air. Colourless crystals of **2** suitable for SCXRD were obtained by recrystallization in MeOH.

*1-(2-nitrobenzyl)-1,3,5-triaza-7-phosphaadamantan-1-ium bromide* (PTA-CH_2_-*o*-NO_2_-C_6_H_4_)Br (**1**). Yield = 89.2% (3.18 g) based on PTA. Elemental analysis calcd (%) for C_13_H_18_BrN_4_O_2_P: C 41.84, H 4.86, N 15.01; found: C 41.76, H 4.80, N 14.84. FTIR (KBr): ν (cm^−1^) = 2978 (w), 2946 (w), 1578 (w), 1519 (s), 1449 (m), 1417 (m), 1388 (w), 1348 (s), 1316 (m), 1275 (m), 1258 (m), 1204 (w), 1130 (m), 1070 (m), 1032 (s), 976 (s), 937 (w), 908 (m), 893 (m), 850 (m), 812 (m), 789 (s), 748 (s), 726 (s), 665 (m), 606 (w), 549 (m), 439 (m). ^1^H NMR (400 MHz, DMSO-*d*_6_, *δ*)/COSY: 8.19 (d, *J* = 7.6 Hz, 1H, Ar−H, C*H*−CNO_2_), 7.87 (m, 3H, Ar−H), 5.16 (d, *J* = 10.8 Hz, 2H, NC*H*_2_N^+^), 5.01 (d, *J* = 10.8 Hz, 2H, NC*H*_2_N^+^), 4.56 (s, 2H, CC*H*_2_N^+^), 4.52 (d, *J* = 13.6 Hz, 1H, NC*H*_2_N), 4.42 (d, *J* = 13.2 Hz, 1H, NC*H*_2_N), 4.26 (br s, 2H, PC*H*_2_N^+^), 3.92–3.81 (m, 4H, PC*H*_2_N). ^31^P{^1^H} NMR (400 MHz, DMSO-*d*_6_, *δ*): −82.00. ^13^C{^1^H} NMR (400 MHz, DMSO-*d*_6_, *δ*)/DEPT/HSQC: 150.90 (*C*_quat_−NO_2_), 136.39 (Ar−*C*H), 133.74 (Ar−*C*H), 132.20 (Ar−*C*H), 125.87 (Ar−*C*H), 119.62 (*C*_quat_−CH_2_), 79.01 (N*C*H_2_N^+^), 68.99 (N*C*H_2_N), 59.55 (C*C*H_2_N^+^), 51.55 (^1^*J*_pc_ = 132.4 Hz, P*C*H_2_N^+^), 45.03 (^1^*J*_pc_ = 80.8 Hz, P*C*H_2_N). DEPT (400 MHz, DMSO-*d*_6_, *δ*): 136.14, 133.50, 131.96, 125.62, 78.75, 68.73, 59.29, 51.29, 44.77. ESI(-)MS in MeOH (*m*/*z* assignment, % intensity): 453 ([M + Br]^−^, 100). ESI(+)MS in MeOH (*m*/*z* assignment, % intensity): 665 ([M+{PTA-CH_2_-*o*-NO_2_-C_6_H_4_}]^+^, 23), 293 ([PTA-CH_2_-*o*-NO_2_-C_6_H_4_]^+^, 100).

*1-(3-nitrobenzyl)-1,3,5-triaza-7-phosphaadamantan-1-ium bromide* (PTA-CH_2_-*m*-NO_2_-C_6_H_4_)Br (**2**). Yield = 97.3% (3.47 g) based on PTA. Elemental analysis calcd (%) for C_13_H_18_BrN_4_O_2_P: C 41.84, H 4.86, N 15.01; found: C 41.86, H 4.91, N 15.10. FTIR (KBr): ν (cm^−1^) = 2944 (w), 2911 (w), 1535 (s), 1444 (m), 1347 (s), 1313 (m), 1240 (w), 1215 (w), 1128 (w), 1097 (w), 1084 (m), 1066 (m), 1031 (s), 980 (m), 942 (w), 917 (m), 891 (m), 849 (w), 802 (s), 759 (m), 744 (m), 726 (s), 696 (m), 671 (m), 553 (m), 441 (m). ^1^H NMR (300 MHz, DMSO-*d*_6_, *δ*)/COSY: 8.42 (s, 1H, Ar−H, C_quat_−C*H*−CNO_2_), 8.40 (d, *J* = 9 Hz, 1H, Ar−H, C*H*−CNO_2_), 8.02 (d, *J* = 7.5 Hz, 1H, Ar−H, C*H*−C−C−CNO_2_), 7.83 (t, *J* = 7.9 Hz, 1H, Ar−H, C*H*−C−CNO_2_), 5.20 (d, *J* = 11.4 Hz, 2H, NC*H*_2_N^+^), 4.95 (d, *J* = 11.1 Hz, 2H, NC*H*_2_N^+^), 4.57 (d, *J* = 12.9 Hz, 1H, NC*H*_2_N), 4.39 (s, 2H, CC*H*_2_N^+^), 4.37 (m, 1H, NC*H*_2_N, 2H, PC*H*_2_N^+^), 3.96–3.75 (m, 4H, PC*H*_2_N). ^31^P{^1^H} NMR (300 MHz, DMSO-*d*_6_, *δ*): −83.26. ^13^C{^1^H} NMR (300 MHz, DMSO-*d*_6_, *δ*)/DEPT/HSQC: 147.99 (*C*_quat_−NO_2_), 139.48 (Ar−*C*H), 130.56 (Ar−*C*H), 128.00 (Ar−*C*H), 127.64 (*C*_quat_−CH_2_), 125.00 (Ar−*C*H), 78.70 (N*C*H_2_N^+^), 69.23 (N*C*H_2_N), 62.96 (C*C*H_2_N^+^), 51.59 (^1^*J*_pc_ = 131.1 Hz, P*C*H_2_N^+^), 45.27 (^1^*J*_pc_ = 81 Hz, P*C*H_2_N). DEPT (300 MHz, DMSO-*d*_6_, *δ*): 139.27, 130.35, 127.43, 124.79, 78.47, 68.98, 62.75, 51.34, 45.02. ESI(-)MS in MeOH (*m*/*z* assignment, % intensity): 453 ([M + Br]^−^, 100). ESI(+)MS in MeOH (*m*/*z* assignment, % intensity): 665 ([M+{PTA-CH_2_-*m*-NO_2_-C_6_H_4_}]^+^, 35), 293 ([PTA-CH_2_-*m*-NO_2_-C_6_H_4_]^+^, 100). 

*1-(4-nitrobenzyl)-1,3,5-triaza-7-phosphaadamantan-1-ium bromide* (PTA-CH_2_-*p*-NO_2_-C_6_H_4_)Br (**3**). Yield = 98.2% (3.50 g) based on PTA. Elemental analysis calcd (%) for C_13_H_18_BrN_4_O_2_P: C 41.84, H 4.86, N 15.01; found: C 41.61, H 4.79, N 14.88. FTIR (KBr): ν (cm^−1^) = 3044 (w), 2943 (w), 1606 (w), 1518 (s), 1450 (m), 1388 (w), 1351 (s), 1317 (m), 1212 (w), 1129 (w), 1107 (w), 1072 (m), 1032 (m), 986 (m), 924 (m), 899 (w), 847 (m), 810 (m), 750 (m), 723 (m), 698 (m), 645 (w), 586 (m), 549 (m), 443 (w). ^1^H NMR (400 MHz, DMSO-*d*_6_, *δ*) / COSY: 8.35 (d, *J* = 8 Hz, 2H, Ar−H, C*H*−CNO_2_), 7.86 (d, *J* = 8 Hz, 2H, Ar−H, *C*_quat_−C*H*), 5.25 (d, *J* = 10.8 Hz, 2H, NC*H*_2_N^+^), 4.97 (d, *J* = 10.8 Hz, 2H, NC*H*_2_N^+^), 4.56 (d, *J* = 13.2 Hz, 1H, NC*H*_2_N), 4.41 (s, 2H, CC*H*_2_N^+^), 4.39 (br s, 1H, NC*H*_2_N, 2H, PC*H*_2_N^+^), 3.95–3.78 (m, 4H, PC*H*_2_N). ^31^P{^1^H} NMR (400 MHz, DMSO-*d*_6_, *δ*): −83.07. ^13^C{^1^H} NMR (400 MHz, DMSO-*d*_6_, *δ*)/DEPT/HSQC: 148.60 (*C*_quat_−NO_2_), 134.54 (Ar−*C*H), 133.38 (*C*_quat_−CH_2_), 123.80 (Ar−*C*H), 78.70 (N*C*H_2_N^+^), 69.16 (N*C*H_2_N), 62.79 (C*C*H_2_N^+^), 51.63 (^1^*J*_pc_ = 130.8 Hz, P*C*H_2_N^+^), 45.24 (^1^*J*_pc_ = 80.8 Hz, P*C*H_2_N). DEPT (400 MHz, DMSO-*d*_6_, *δ*): 134.30, 123.56, 78.47, 68.91, 62.57, 51.38, 44.99. ESI(-)MS in MeOH (*m*/*z* assignment, % intensity): 453 ([M + Br]^−^, 100). ESI(+)MS in MeOH (*m*/*z* assignment, % intensity): 665 ([M+{PTA-CH_2_-*p*-NO_2_-C_6_H_4_}]^+^, 18), 293 ([PTA-CH_2_-*p*-NO_2_-C_6_H_4_]^+^, 100).

### 2.3. Synthesis of the Complexes (4–7)

#### 2.3.1. Synthesis of [Cu(PTA-Me)_4_]Br_5_ (**4**)

To a methanol solution (20 mL) of **1** (180 mg, 0.48 mmol, 4 equiv.) was added an acetonitrile solution (10 mL) of [Cu(MeCN)_4_]BF_4_ (38 mg, 0.12 mmol, 1 equiv.) dropwise with stirring at room temperature. The produced colourless solution was stirred for further 3 h at room temperature, and then left in open air for slow evaporation to afford colourless crystals of **4** suitable for SXCRD. The crystals of **4** were isolated by filtration, washed with diethyl ether, and dried in air. Yield = 35% (48.4 mg) based on the metal salt.

Compound **4** can be obtained using the same procedure by replacing [Cu(MeCN)_4_]BF_4_ with CuBr (18 mg, 0.12 mmol, 1 equiv.). Yield = 37.4% (51.7 mg) based on the metal salt.

Elemental analysis calcd (%) for C_28_H_60_Br_5_CuN_12_P_4_: C 29.20, H 5.25, N 14.59; found: C 29.31, H 5.18, N 14.66. FTIR (KBr): ν (cm^−1^) = 1463 (s), 1268 (m), 1261 (m), 1149 (m), 1081 (s), 1029 (s), 639 (m), 556 (s), 520 (m). ^1^H NMR (400 MHz, DMSO-*d*_6_, *δ*)/COSY: 5.21 (d, *J* = 10.4 Hz, 2H, NC*H*_2_N^+^), 4.95 (d, *J* = 11.2 Hz, 2H, NC*H*_2_N^+^), 4.70 (s, 2H, PC*H*_2_N^+^), 4.64 (d, *J* = 13.6 Hz, 1H, NC*H*_2_N), 4.33 (d, *J* = 14 Hz, 1H, NC*H*_2_N), 4.13 (d, *J* = 12.8 Hz, 2H, PC*H*_2_N), 3.89 (d, *J* = 14 Hz, 2H, PC*H*_2_N), 2.72 (s, 3H, C*H*_3_N^+^). ^31^P{^1^H} NMR (400 MHz, DMSO-*d*_6_, *δ*): −80.21. ^13^C{^1^H} NMR (400 MHz, DMSO-*d*_6_, *δ*)/DEPT/HSQC: 79.21 (N*C*H_2_N^+^), 68.58 (N*C*H_2_N), 54.72 (^1^*J*_pc_ = 63.6 Hz, P*C*H_2_N^+^), 48.63 (*C*H_3_N^+^), 45.43 (^1^*J*_pc_ = 31.2 Hz, P*C*H_2_N). DEPT (400 MHz, DMSO-*d*_6_, *δ*): 78.95, 68.32, 54.46, 48.37, 45.18. ESI(+)MS in H_2_O (*m*/*z* assignment, % intensity): 1215 ([M+Cu]^+^, 7), 567 ([CuBr_2_{PTA-CH_3_}_2_]^+^, 37), 316 ([CuBr{PTA-CH_3_}]^+^, 71), 172 ([PTA-CH_3_]^+^, 100). 

#### 2.3.2. Synthesis of [CuBr_2_(PTA-CH_2_-m-NO_2_-C_6_H_4_)_2_]Br (**5a**)

To an acetonitrile solution (10 mL) of CuBr (24 mg, 0.16 mmol, 1 equiv.) was added **2** (120 mg, 0.32 mmol, 2 equiv.) in a solution mixture of methanol (6 mL) and water (6 mL) dropwise with stirring at room temperature. The obtained clear yellow solution was stirred for further 30 min at room temperature, and then left in open air for slow evaporation to afford yellow crystals of **5a** suitable for SXCRD after 2 days. The crystals of **5a** were isolated by filtration, washed with diethyl ether, and dried in air.

Yield = 46.4% (70 mg) based on the metal salt. Elemental analysis calcd (%) for C_26_H_36_Br_3_CuN_8_O_4_P_2_·3H_2_O: C 33.09, H 4.49, N 11.87; found: C 33.17, H 4.43, N 11.92. FTIR (KBr): ν (cm^−1^) = 3422 br (m), 2973 (m), 2942 (m), 1522 (m), 1448 (m), 1432 (m), 1396 (w), 1344 (w), 1320 (w), 1299 (m), 1278 (w), 1250 (m), 1120 (m), 1091 (s), 1027 (m), 1006 (m), 975 (m), 924 (m), 897 (s), 871 (m), 851 (m), 813 (s), 779 (m), 762 (m), 748 (m), 727 (m), 650 (w), 563 (s), 468 (m), 443 (m). ^1^H NMR (300 MHz, DMSO-*d*_6_, *δ*)/COSY: 8.42 (s, 2H, Ar−H, C_quat_−C*H*−CNO_2_), 8.39 (s, 2H, Ar−H, C*H*−CNO_2_), 7.97 (d, *J* = 7.5 Hz, 2H, Ar−H, C*H*−C−C−CNO_2_), 7.82 (t, *J* = 7.8 Hz, 2H, Ar−H, C*H*−C−CNO_2_), 5.07 (br s, 8H, NC*H*_2_N^+^), 4.57 (d, *J* = 13.2 Hz, 2H, NC*H*_2_N), 4.42 (br s, 4H, CC*H*_2_N^+^, 4H, PC*H*_2_N^+^), 4.31 (d, *J* = 13.5 Hz, 2H, NC*H*_2_N), 4.05-3.85 (m, 8H, PC*H*_2_N). ^31^P{^1^H} NMR (300 MHz, DMSO-*d*_6_, *δ*): −74.86. ^13^C{^1^H} NMR (300 MHz, DMSO-*d*_6_, *δ*)/DEPT/HSQC: 148.08 (*C*_quat_−NO_2_), 139.48 (Ar−*C*H), 130.70 (Ar−*C*H), 127.82 (Ar−*C*H), 127.75 (*C*_quat_−CH_2_), 125.23 (Ar−*C*H), 78.63 (N*C*H_2_N^+^), 68.86 (N*C*H_2_N), 62.91 (C*C*H_2_N^+^), 51.70 (P*C*H_2_N^+^), 46.16 (P*C*H_2_N). DEPT (300 MHz, DMSO-*d*_6_, *δ*): 139.59, 130.81, 127.94, 125.18, 78.71, 68.94, 62.86, 51.78, 46.18. ESI(+)MS in H_2_O (*m*/*z* assignment, % intensity): 953 ([M+Cu]^+^, 11), 809 ([CuBr_2_{PTA-CH_2_-*m*-NO_2_-C_6_H_4_}_2_]^+^, 38), 437 ([CuBr{PTA-CH_2_-*m*-NO_2_-C_6_H_4_}]^+^, 24), 293 ([PTA-CH_2_-*m*-NO_2_-C_6_H_4_]^+^, 100). 

#### 2.3.3. Synthesis of [CuBr_2_(PTA-CH_2_-m-NO_2_-C_6_H_4_)_2_]I (**5b**)

To an acetonitrile solution (7 mL) of CuI (30 mg, 0.16 mmol, 1 equiv.) was added a methanol solution (45 mL) of **2** (120 mg, 0.32 mmol, 2 equiv.) dropwise with stirring at room temperature. The obtained yellow suspension of compound **5b** was stirred for 2 h, and then filtered off, repeatedly washed with diethyl ether and dried in air.

Yield = 42.9% (68 mg) based on the metal salt. Elemental analysis calcd (%) for C_26_H_36_Br_2_CuIN_8_O_4_P_2_·3H_2_O: C 31.52, H 4.27, N 11.31; found: C 31.47, H 4.39, N 11.15. FTIR (KBr): ν (cm^−1^) = 3389 br (m), 2948 (w), 1617 (w), 1525 (s), 1449 (m), 1342 (s), 1306 (m), 1122 (w), 1066 (m), 1031 (s), 980 (m), 915 (w), 893 (w) 803 (s), 765 (m), 727 (s), 690 (m), 672 (m), 555 (m), 440 (m). ^1^H NMR (400 MHz, DMSO-*d*_6_, *δ*)/COSY: 8.41 (br s, 4H, Ar−H, C_quat_−C*H*−CNO_2_, C*H*−CNO_2_), 7.95 (d, *J* = 7.2 Hz, 2H, Ar−H, C*H*−C−C−CNO_2_), 7.83 (t, *J* = 8 Hz, 2H, Ar−H, C*H*−C−CNO_2_), 5.03 (br s, 8H, NC*H*_2_N^+^), 4.55 (d, *J* = 13.2 Hz, 2H, NC*H*_2_N), 4.39 (s, 4H, CC*H*_2_N^+^), 4.35 (s, 4H, PC*H*_2_N^+^), 4.30 (d, *J* = 13.6 Hz, 2H, NC*H*_2_N), 4.00–3.83 (m, 8H, PC*H*_2_N). ^31^P{^1^H} NMR (400 MHz, DMSO-*d*_6_, *δ*): −77.81. ^13^C{^1^H} NMR (400 MHz, DMSO-*d*_6_, *δ*)/DEPT/HSQC: 148.03 (*C*_quat_−NO_2_), 139.43 (Ar−*C*H), 130.65 (Ar−*C*H), 127.81 (Ar−*C*H), 127.70 (*C*_quat_−CH_2_), 125.16 (Ar−*C*H), 78.68 (N*C*H_2_N^+^), 68.87 (N*C*H_2_N), 62.92 (C*C*H_2_N^+^), 51.18 (P*C*H_2_N^+^), 46.42 (P*C*H_2_N). DEPT (400 MHz, DMSO-*d*_6_, *δ*): 139.18, 130.40, 127.44, 124.90, 78.41, 68.58, 62.66, 51.86, 46.17. ESI(+)MS in H_2_O (*m*/*z* assignment, % intensity): 1001 ([M+Cu]^+^, 6), 809 ([CuBr_2_{PTA-CH_2_-*m*-NO_2_-C_6_H_4_}_2_]^+^, <5), 665 ([Br{PTA-CH_2_-*m*-NO_2_-C_6_H_4_}_2_]^+^, <5), 293 ([PTA-CH_2_-*m*-NO_2_-C_6_H_4_]^+^, 100).

#### 2.3.4. Synthesis of [CuX(PTA-CH_2_-m-NO_2_-C_6_H_4_)_3_]Br_3_ (X = Br for **6a** or I for **6b**)

To a methanol solution (45 mL) of **2** (120 mg, 0.32 mmol, 4 equiv.) was added an acetonitrile solution (7 mL) of the copper salt (12 mg of CuBr for **6a** or 15 mg of CuI for **6b**; 0.08 mmol, 1 equiv.) dropwise with stirring at room temperature. The obtained colourless solution was stirred for further 3 h at room temperature, and then left in open air for slow evaporation to afford the products as colourless crystals, which were isolated by filtration, washed with diethyl ether, and dried in air.

*[CuBr(PTA-CH_2_-m-NO_2_-C_6_H_4_)_3_]Br_3_ (**6a**)*. Yield = 56.9% (60 mg) based on the metal salt. Elemental analysis calcd (%) for C_39_H_54_Br_4_CuN_12_O_6_P_3_·3H_2_O: C 35.57, H 4.59, N 12.76; found: C 35.80, H 4.43, N 12.79. FTIR (KBr): ν (cm^−1^) = 3483 br (m), 2953 (m), 1606 (m), 1521 (s), 1450 (m), 1349 (s), 1310 (m), 1214 (w), 1123 (m), 1104 (m), 1070 (m), 1034 (s), 982 (m), 927 (w), 893 (m), 852 (m), 808 (s), 764 (m), 732 (s), 690 (m), 552 (m), 439 (m), 426 (m), 412 (m). ^1^H NMR (400 MHz, DMSO-*d*_6_, *δ*) / COSY: 8.43 (s, 3H, Ar−H, C_quat_−C*H*−CNO_2_), 8.40 (d, 3H, *J* = 8.4 Hz, Ar−H, C*H*−CNO_2_), 7.99 (d, *J* = 7.6 Hz, 3H, Ar−H, C*H*−C−C−CNO_2_), 7.82 (t, *J* = 8.4 Hz, 3H, Ar−H, C*H*−C−CNO_2_), 5.09 (br s, 12H, NC*H*_2_N^+^), 4.60 (d, *J* = 13.6 Hz, 3H, NC*H*_2_N), 4.52 (s, 6H, PC*H*_2_N^+^), 4.44 (s, 6H, CC*H*_2_N^+^), 4.33 (d, *J* = 13.2 Hz, 3H, NC*H*_2_N), 4.08–3.88 (m, 12H, PC*H*_2_N). ^31^P{^1^H} NMR (400 MHz, DMSO-*d*_6_, *δ*): −73.78. ^13^C{^1^H} NMR (400 MHz, DMSO-*d*_6_, *δ*)/DEPT/HSQC: 148.05 (*C*_quat_−NO_2_), 139.43 (Ar−*C*H), 130.64 (Ar−*C*H), 127.81 (Ar−*C*H), 127.70 (*C*_quat_−CH_2_), 125.17 (Ar−*C*H), 78.57 (N*C*H_2_N^+^), 68.81 (N*C*H_2_N), 62.83 (C*C*H_2_N^+^), 51.65 (P*C*H_2_N^+^), 46.10 (P*C*H_2_N). DEPT (400 MHz, DMSO-*d*_6_, *δ*): 139.18, 130.39, 127.45, 124.92, 78.31, 68.56, 62.57, 51.42, 45.84. ESI(+)MS in H_2_O (*m*/*z* assignment, % intensity): 1327 ([M+Cu]^+^, <5), 1183 ([CuBr_3_{PTA-CH_2_-*m*-NO_2_-C_6_H_4_}_3_]^+^, <5), 809 ([CuBr_2_{PTA-CH_2_-*m*-NO_2_-C_6_H_4_}_2_]^+^, 10), 293 ([PTA-CH_2_-*m*-NO_2_-C_6_H_4_]^+^, 100), 172 ([PTA-CH_3_]^+^, 10). 

*[CuI(PTA-CH_2_-m-NO_2_-C_6_H_4_)_3_]Br_3_ (**6b**)*. Yield = 83% (87 mg) based on the metal salt. Elemental analysis calcd (%) for C_39_H_54_Br_3_CuIN_12_O_6_P_3_: C 35.76, H 4.15, N 12.83; found: C 35.54, H 4.14, N 12.67. FTIR (KBr): ν (cm^−1^) = 3412 br (m), 2943 (m), 1610 (m), 1525 (s), 1449 (m), 1346 (s), 1307 (m), 1258 (m), 1216 (m), 1121 (m), 1068 (m), 1033 (s), 982 (m), 915 (w), 893 (m), 851 (m), 803 (s), 765 (w), 728 (s), 689 (m), 672 (m), 558 (m), 483 (w), 441 (m). ^1^H NMR (300 MHz, DMSO-*d*_6_, *δ*)/COSY: 8.43 (s, 3H, Ar−H, C_quat_−C*H*−CNO_2_), 8.40 (d, 3H, *J* = 8.7 Hz, Ar−H, C*H*−CNO_2_), 7.98 (d, *J* = 7.8 Hz, 3H, Ar−H, C*H*−C−C−CNO_2_), 7.82 (t, *J* = 7.8 Hz, 3H, Ar−H, C*H*−C−CNO_2_), 5.08 (br s, 12H, NC*H*_2_N^+^), 4.59 (d, *J* = 13.2 Hz, 3H, NC*H*_2_N), 4.49 (s, 6H, PC*H*_2_N^+^), 4.43 (s, 6H, CC*H*_2_N^+^), 4.32 (d, *J* = 13.5 Hz, 3H, NC*H*_2_N), 4.08–3.87 (m, 12H, PC*H*_2_N). ^31^P{^1^H} NMR (300 MHz, DMSO-*d*_6_, *δ*): −75.05. ^13^C{^1^H} NMR (300 MHz, DMSO-*d*_6_, *δ*)/DEPT/HSQC: 148.04 (*C*_quat_−NO_2_), 139.41 (Ar−*C*H), 130.64 (Ar−*C*H), 127.78 (Ar−*C*H), 127.69 (*C*_quat_−CH_2_), 125.16 (Ar−*C*H), 78.58 (N*C*H_2_N^+^), 68.83 (N*C*H_2_N), 62.84 (C*C*H_2_N^+^), 51.98 (P*C*H_2_N^+^), 46.23 (P*C*H_2_N). DEPT (300 MHz, DMSO-*d*_6_, *δ*): 139.16, 130.39, 127.44, 124.91, 78.32, 68.56, 62.58, 51.61, 45.98. ESI(+)MS in H_2_O (*m*/*z* assignment, % intensity): 1375 ([M+Cu]^+^, <5), 1230 ([CuBr_2_I{PTA-CH_2_-*m*-NO_2_-C_6_H_4_}_3_]^+^, <5), 1183 ([CuBr_3_{PTA-CH_2_-*m*-NO_2_-C_6_H_4_}_3_]^+^, <5), 857 ([CuBrI{PTA-CH_2_-*m*-NO_2_-C_6_H_4_}_2_]^+^, 12), 809 ([CuBr_2_{PTA-CH_2_-*m*-NO_2_-C_6_H_4_}_2_]^+^, 6), 293 ([PTA-CH_2_-*m*-NO_2_-C_6_H_4_]^+^, 100), 172 ([PTA-CH_3_]^+^, <5).

#### 2.3.5. Synthesis of [CuBr_2_(PTA-CH_2_-p-NO_2_-C_6_H_4_)_2_]X (X = Br for **7a** or I for **7b**)

To an acetonitrile solution (10 mL) of the copper salt (24 mg of CuBr for **7a** or 30 mg of CuI for **7b**; 0.16 mmol, 1 equiv.) was added a methanol solution (30 mL) of **3** (120 mg, 0.32 mmol, 2 equiv.) dropwise with stirring at room temperature. The obtained yellow suspension of the product was stirred for 2 h, and then filtered off, repeatedly washed with diethyl ether and dried in air.

*[CuBr_2_(PTA-CH_2_-p-NO_2_-C_6_H_4_)_2_]Br (**7a**)*. Yield = 38.1% (54.2 mg) based on the metal salt. Elemental analysis calcd (%) for C_26_H_36_Br_3_CuN_8_O_4_P_2_: C 35.09, H 4.08, N 12.59; found: C 34.85, H 4.25, N 12.47. FTIR (KBr): ν (cm^−1^) = 3456 (w), 2952 (w), 1606 (w), 1519 (s), 1454 (w), 1421 (w), 1350 (s), 1310 (m), 1212 (w), 1123 (m), 1069 (m), 1032 (s), 984 (m), 927 (m), 902 (w), 887 (w), 851 (m), 811 (m), 750 (m), 731 (s), 700 (m), 646 (w), 587 (w), 551 (m), 440 (m), 416 (m). ^1^H NMR (400 MHz, DMSO-*d*_6_, *δ*)/COSY: 8.36 (d, *J* = 8 Hz, 4H, Ar−H, C*H*−CNO_2_), 7.82 (d, *J* = 8 Hz, 4H, Ar−H, *C*_quat_−C*H*), 5.08 (s, 8H, NC*H*_2_N^+^), 4.58 (d, *J* = 13.2 Hz, 2H, NC*H*_2_N), 4.44 (s, 4H, PC*H*_2_N^+^), 4.39 (s, 4H, CC*H*_2_N^+^), 4.34 (d, *J* = 13.6, 2H, NC*H*_2_N), 4.04-3.87 (m, 8H, PC*H*_2_N). ^31^P{^1^H} NMR (400 MHz, DMSO-*d*_6_, *δ*): −74.13. ^13^C{^1^H} NMR (400 MHz, DMSO-*d*_6_, *δ*)/DEPT/HSQC: 148.72 (*C*_quat_−NO_2_), 134.53 (Ar−*C*H), 133.11 (*C*_quat_−CH_2_), 123.94 (Ar−*C*H), 78.69 (N*C*H_2_N^+^), 68.80 (N*C*H_2_N), 62.95 (C*C*H_2_N^+^), 51.94 (P*C*H_2_N^+^), 46.14 (P*C*H_2_N). DEPT (400 MHz, DMSO-*d*_6_, *δ*): 134.28, 123.69, 78.43, 68.55, 62.59, 51.64, 45.89. ESI(+)MS in H_2_O (*m*/*z* assignment, % intensity): 953 ([M+Cu]^+^, <5), 809 ([CuBr_2_{PTA-CH_2_-*p*-NO_2_-C_6_H_4_}_2_]^+^, 6), 293 ([PTA-CH_2_-*p*-NO_2_-C_6_H_4_]^+^, 100), 172 ([PTA-CH_3_]^+^, 28).

*[CuBr_2_(PTA-CH_2_-p-NO_2_-C_6_H_4_)_2_]I (**7b**)*. Yield = 32% (48 mg) based on the metal salt. Elemental analysis calcd (%) for C_26_H_36_Br_2_CuIN_8_O_4_P_2_: C 33.33, H 3.87, N 11.96; found: C 32.98, H 3.85, N 11.85. FTIR (KBr): ν (cm−1) = 3485 (w), 2954 (w), 1604 (w), 1518 (s), 1423 (w), 1350 (s), 1309 (m), 1211 (m), 1123 (m), 1104 (m), 1071 (m), 1033 (m), 984 (m), 926 (w), 902 (w), 887 (w), 851 (m), 812 (s), 750 (m), 732 (s), 699 (w), 646 (w), 585 (m), 549 (w), 491 (w), 443 (m). ^1^H NMR (400 MHz, DMSO-d6, δ)/COSY: 8.37 (d, J = 8 Hz, 4H, Ar−H, CH−CNO_2_), 7.79 (d, J = 8 Hz, 4H, Ar−H, C_quat_−CH), 5.04 (s, 8H, NCH_2_N+), 4.55 (d, J = 13.2 Hz, 2H, NCH_2_N), 4.37 (s, 4H, CCH_2_N^+^), 4.33 (s, 4H, PCH_2_N^+^), 4.30 (s, 2H, NCH_2_N), 3.99–3.84 (m, 8H, PCH_2_N). ^31^P{^1^H} NMR (400 MHz, DMSO-d_6_, δ): −77.89. ^13^C{^1^H} NMR (400 MHz, DMSO-d_6_, δ)/DEPT/HSQC: 148.69 (C_quat_−NO_2_), 134.53 (Ar−CH), 133.12 (C_quat_−CH_2_), 123.90 (Ar−CH), 78.73 (NCH_2_N^+^), 68.82 (NCH_2_N), 62.91 (CCH_2_N^+^), 52.19 (PCH2N^+^), 46.38 (PCH_2_N). DEPT (400 MHz, DMSO-*d*_6_, δ): 134.28, 123.66, 78.47, 68.56, 62.65, 51.96, 46.12. ESI(+)MS in H_2_O (*m*/*z* assignment, % intensity): 1001 ([M+Cu]^+^, <5), 857 ([CuBrI{PTA-CH_2_-*p*-NO_2_-C_6_H_4_}_2_]^+^, <5), 809 ([CuBr_2_{PTA-CH_2_-*p*-NO_2_-C_6_H_4_}_2_]^+^, <5), 665 ([Br{PTA-CH_2_-*P*-NO_2_-C_6_H_4_}_2_]^+^, <5), 293 ([PTA-CH_2_-*p*-NO_2_-C_6_H_4_]^+^, 100), 172 ([PTA-CH_3_]^+^, 6).

### 2.4. X-ray Structure Determination of Compounds ***2***, ***4*** and ***5a***

X-ray quality crystals of the compound were immersed in cryo-oil, mounted in a Nylon loop and measured at ambient temperature. Intensity data were collected using a Bruker AXS-KAPPA APEX II PHOTON 100 diffractometer with graphite monochromated Mo-Kα (0.71069 Å) radiation. Data were collected using omega scans of 0.5° per frame and full sphere of data were obtained. Cell parameters were retrieved using Bruker SMART [43] software and refined using Bruker SAINT [43] on all the observed reflections. Absorption corrections were applied using the SADABS program [44]. The structures were solved by direct methods using SIR97 package [45] and refined with SHELXL-2014/7 [46]. Calculations were performed using the WinGX System-Version 2014.1 [47]. A positional disorder between P1 and N2 atoms has been observed in phosphane 2 which was modelled by means of PART instruction, their occupancy being refines to a ration of ca. 50% for each. Identical procedure was followed to model one of the bromide counter anions in 4; the occupancy of Br5a and Br5b was refined to a ratio of 72% and 28%, respectively. The hydrogen atoms were included in the refinement using the riding-model approximation; Uiso(H) were defined as 1.2Ueq of the parent carbon atoms for phenyl and methylene residues, and 1.5Ueq of the parent carbon atoms for methyl. Least square refinements with anisotropic thermal motion parameters for all the non-hydrogen atoms were employed. Least square refinements with anisotropic thermal motion parameters for all the non-hydrogen atoms and isotropic ones for the remaining atoms were employed.

Crystallographic data for the structural analysis have been deposited to the Cambridge Crystallographic Data Center [CCDC 2097590 (for **2**), 2097591 (for **4**) and 2097592 (for **5a**)].

### 2.5. Treatments of the Carbon Materials

The activated carbon (AC) and multi-walled carbon nanotube (CNT) materials were employed as solid supporting matrices, which were used as received, or subjected to surface treatments. The surface treatments include oxidation by refluxing a 1 g of carbon in 75 mL of a 5 M HNO_3_ solution for a period of 3 h, followed by filtration and washing with distilled water until neutral pH [48,49,50,51,52,53] to obtain the AC-ox and CNT-ox supports. To attain the AC-ox-Na and CNT-ox-Na supports, the previously prepared AC-ox and CNT-ox materials were added with 75 mL of a 20 mM NaOH solution and were refluxed for 1 h. After the indicated period, the materials were separated by filtration and washed with distilled water until neutral pH [48,49,50,52,53,54,55,56,57]. In this way, a total of six different carbon materials were used in the work. 

### 2.6. Heterogenization Protocol

The heterogenization procedure was done by immobilization of complexes 5a and 5b onto the six different carbon supports. A calculated amount of each complex was dissolved in 40 mL of a 1:1 (*v*/*v*) H_2_O:MeCN solvent mixture. Once completely dissolved, the solution was added with 0.15 g of carbon material to achieve the desired ~2 wt% Cu per mass of carbon by stirring continuously for 72 h at RT. After the indicated period, the mixture was filtered-off, washed with distilled water and dried overnight at 120 °C. 

### 2.7. Catalytic Essays

#### 2.7.1. Synthesis of 1,4-disubstituted 1,2,3-triazole

In a typical procedure, a tightly capped 10 mL borosilicate glass vial equipped with a magnetic stir bar and containing the catalyst, benzyl bromide (0.3 mmol), ethynylbenzene (0.3 mmol), and NaN_3_ (0.3 mmol) in 1.5 mL of the solvent mixture (H_2_O:MeCN, 1:1 *v*/*v*), was placed in a microwave reactor. The reaction mixture was simultaneously irradiated (30 W) and stirred (600 rpm) for 60 min at 125 °C. After the set reaction time, the mixture was allowed to cool at room temperature. 

For reactions in homogeneous system, the mixture was diluted with 5 mL of water and the product was collected by filtration, washed repeatedly with petroleum ether and dried under vacuum. 

For reactions using carbon supported catalysts, the heterogenized catalyst were separated by filtration, washed with water, dried in a vacuum oven and reused as suitable. The triazole product was extracted from the filtrate with ethyl acetate. The organic phase was collected and taken to dryness to afford the triazole product, which was washed with petroleum ether and no further purification steps ware required. 

#### 2.7.2. Synthesis of N-hydroxymethyl-1,2,3-triazole

A mixture of 37% HCHO_aq_ (451 µL, 6 mmol, 10 equiv.), glacial AcOH (52 µL, 0.9 mol, 1.5 equiv.), and 1,4-dioxane (451 µL) was put in a tightly capped 10 mL borosilicate glass vial equipped with a magnetic stir bar. The solution was stirred for 15 min, and NaN_3_ (59 mg, 0.9 mol, 1.5 equiv.) was added followed by ethynylbenzene (66 µL, 0.6 mmol, 1 equiv.). After an additional 10 min of stirring, the catalyst (5a, 3 mol % or the carbon-supported 5a, 2.5 mol % relative to ethynylbenzene) was added, followed by 500 µL of distilled H_2_O. The reaction vial was then placed in a microwave reactor where it was simultaneously stirred (600 rpm) and irradiated (30 W) for 60 min at 125 °C. After the set reaction time, the mixture was allowed to cool at room temperature and phases were observed. After extraction with ethyl acetate, the organic phase was collected and taken to dryness to afford the corresponding triazole product; it was washed with petroleum ether and no further purification step was performed. For the heterogenized catalyst, the separation was done by filtration, washed with water, oven-dried, and reused as suitable. 

## 3. Results and Discussion

### 3.1. Synthesis and Characterization of the Compounds

**Scheme 3:**
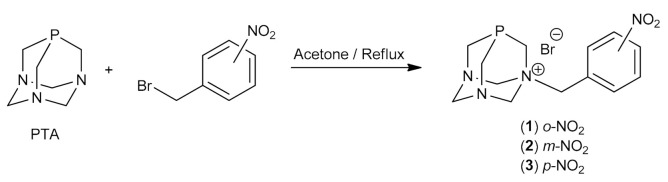
Synthesis of the PTA ammonium salts **1**–**3**.

The addition of bromide benzylic compounds of the type R-CH_2_Br (R = *o*-NO_2_-C_6_H_4_, *m*-NO_2_-C_6_H_4_ and *p*-NO_2_-C_6_H_4_) to an equimolar amount of PTA in acetone under reflux gave the corresponding *N*-alkylated PTA derivatives (PTA-CH_2_-*o*-NO_2_-C_6_H_4_)Br (**1**), (PTA-CH_2_-*m*-NO_2_-C_6_H_4_)Br (**1**) and (PTA-CH_2_-*p*-NO_2_-C_6_H_4_)Br (**3**), respectively, as air-stable white solids (Scheme 3). Due to the ammonium salt functionality of the compounds, they exhibit a high solubility in water and polar organic solvents such as methanol, acetonitrile, dimethyl sulfoxide and dimethyl formamide.

Elemental analysis of the compounds (**1**–**3**) supports the proposed formulations. In all cases, ESI(+)MS spectra exhibit the base peak at *m*/*z* 293 corresponding to the ammonium cation species upon the loss of the bromide counterion. Quaternization of the tertiary amine to produce the PTA ammonium salts **1**–**3** has been confirmed by NMR spectroscopy experiments in DMSO-*d*_6_ (Figures S1–S18). 

The ^1^H-NMR spectra of compounds **1**–**3** show a more complicated pattern for the methylene protons when compared to the simple pattern of signals observed in the parent PTA molecule due to the asymmetry generated by the *N*-alkylation of the phosphane (Figure S19). The methylene protons of **1**–**3** became inequivalent and diastereotopic, and therefore were observed as doublets with P-H or H-H coupling or multiplets. The ^1^H-NMR spectra of **1**–**3** also exhibit the presence of the aromatic moieties in each case as a set of signals assigned to four protons of the aromatic ring in the 8.43–7.80 ppm range. The methylene group that links the aromatic ring to the PTA cage is observed as a sharp singlet at 4.56, 4.39 or 4.41 ppm for **1**–**3**, respectively. 

The ^13^C{^1^H}-NMR spectra further proved the presence of the aromatic rings with six singlets for **1** and **2**, and four singlets for **3** in the 150–120 ppm range. The methylene carbons of N-*C*H_2_-N, N-*C*H_2_-N^+^ and Ar-*C*H_2_-PTA appeared as singlets, whereas those of P-*C*H_2_-N and P-*C*H_2_-N^+^ appeared as doublets due to the first order ^31^P-^13^C coupling (^1^*J*_PC_). The ^1^*J*_PC_ values for P-CH_2_-N^+^ (130.8–132.4 Hz) were found to be higher than those of P-CH_2_-N (80.8–81.0 Hz). 

The ^31^P{^1^H}-NMR spectra of **1**–**3** are simple with ^31^P resonances appearing as sharp singlets at −82.00, −83.26 and −83.07 ppm, respectively, downfield shifted from that of their precursor (Figure S20). 

**Scheme 4:**
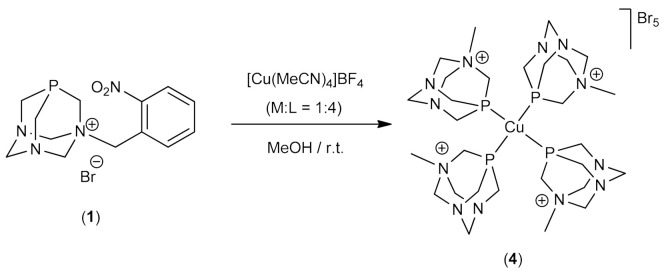
Synthesis of the complex [Cu(PTA-Me)]Br_5_ (**4**).

As an attempt to obtain a homoleptic copper complex bearing ligand **1**, the labile precursor [Cu(MeCN)_4_]BF_4_ was reacted with a fourfold excess of **1** in methanol at room temperature. As depicted in Scheme 4, the isolated product of the reaction is a Cu(I) complex bearing simpler alkylated PTA ligands, [Cu(PTA-Me)_4_]Br_5_ (**4**) {PTA-Me = *N*-methyl-1,3,5-triaza-7-phosphaadamantane}. Such a decomposition of **1** to a simpler form might be attributed to the effect of the high steric hindrance arising from the presence of the nitro-substituent in *ortho* position. Performing the reaction under the same conditions with decreasing the amounts of **1** did not lead to a different result. Compound **4** was obtained with a slightly better yield when [Cu(MeCN)_4_]BF_4_ was replaced with CuBr under the same reaction conditions. 

**Scheme 5:**
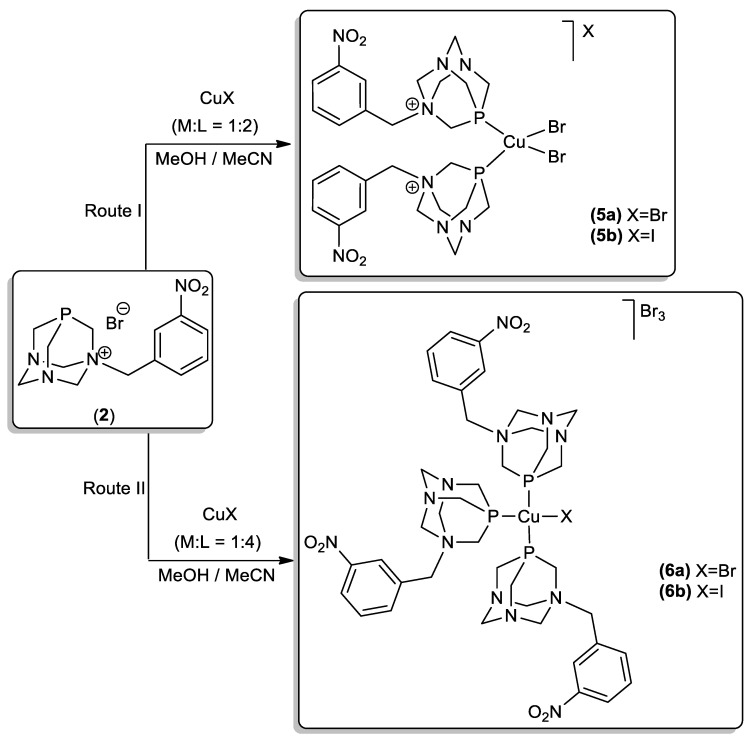
Synthesis of the complexes **5a**, **5b**, **6a** and **6b**.

Complexes with the general formula [CuBr_2_(PTA-CH_2_-*m*-NO_2_-C_6_H_4_)_2_]X (X = Br {**5a**} or I {**5b**}) were obtained by reacting copper(I) halide (i.e., bromide and iodide) salts with **2** using 1:2 metal-to-ligand ratio in a mixture of methanol and acetonitrile at room temperature (Scheme 5, route I). Under the same reaction conditions, but increasing the amount of **2** in the reaction medium (i.e., metal-to-ligand ratio = 1:4) afforded complexes with the general formula [CuX(PTA-CH_2_-*m*-NO_2_-C_6_H_4_)_3_]Br_3_ (X = Br {**6a**} or I {**6b**}; Scheme 5, route II).

**Scheme 6:**
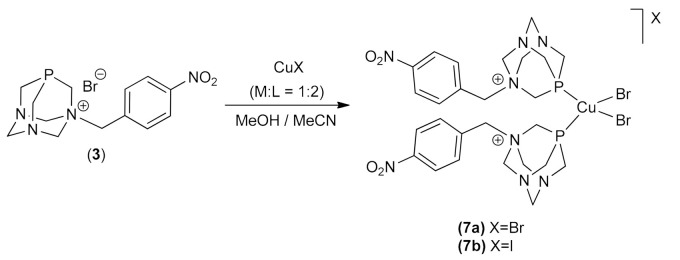
Synthesis of the complexes **7a** and **7b**.

Using the same synthetic technique, reacting copper(I) halide (i.e., bromide and iodide) salts with **3** in the 1:2 metal-to-ligand ratio afforded the Cu(I) complexes with the general formula [CuBr_2_(PTA-CH_2_-*p*-NO_2_-C_6_H_4_)_2_]X (X = Br {**7a**} or I {**7b**}; Scheme 6). Changing the stoichiometric ratio of the reactants by increasing the amount of compound **3** in the reaction medium does not lead to the formation of a different product.

Analogously to their free ligands, all complexes are water-soluble and are stable in solid and solution states. All complexes were characterized by elemental analysis, ESI(+)MS, FT-IR and NMR (^1^H, ^13^C{^1^H}, ^31^P{^1^H}) spectroscopies, which support the proposed formulations. NMR studies of the compounds were carried out in DMSO-*d*_6_ (Figures S21–S62). ^1^H-NMR spectra of complexes **5a**–**7b** show a set of signals correlated to the methylene protons of the corresponding ligands with different chemical shifts and splitting patterns when compared to the free phosphanes (**2** or **3**), while the aromatic protons seem to be unaffected by the coordination (Figures S63 and S64). The ^31^P{^1^H}-NMR spectra show one broad resonance for each compound, downfield shifted from that of the corresponding free ligands (Figures S65 and S66). Such broadness of the ^31^P resonance is typical for the [Cu(P)_n_] (P = PTA derivative) complexes due to the fluxional behaviour of the ligand in solution [26,58,59,60]. 

The ESI(+)MS analysis of the complexes was performed in water. In all cases, the spectra show the base peak (at *m*/*z* 172 for **4** and 293 for **5a**–**7b**) corresponding to the ammonium cation ligand, fragment ions of [M+Cu]^+^ and a set of peaks assigned to the compounds fragmentations.

### 3.2. X-ray Crystal Analysis of Compounds ***2***, ***4*** and ***5a***

The molecular structures of compounds **2**, **4** and **5a**·3H_2_O were established by SCXRD analysis at room temperature. The crystals for each compound were obtained as described in the experimental section. Selected crystallographic data and structure refinement details are provided in Table S1. Selected bond distances and angles are listed in Table 1.

**Figure 1:**
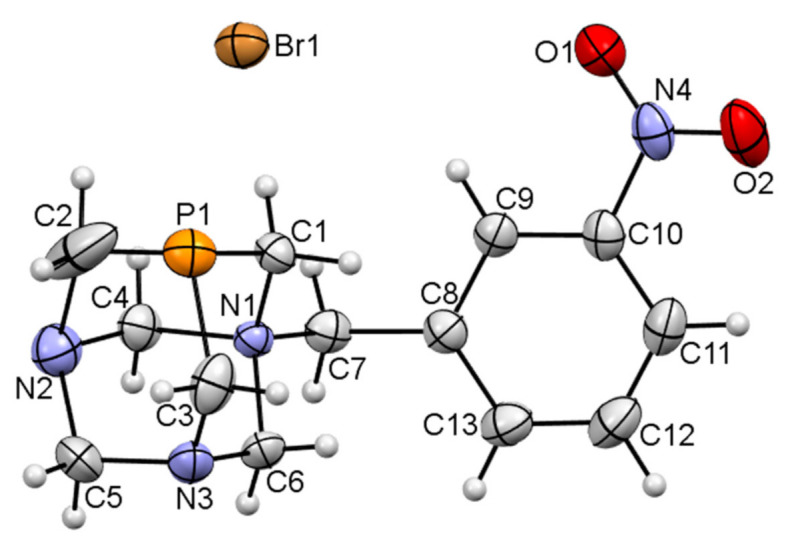
ORTEP diagrams of **2** with displacement ellipsoids shown at 40% probability level and partial atom numbering schemes. Only one component of the disorder is shown.

The phosphane **2** crystallized in the monoclinic space group *P* 21/*n* and the corresponding ORTEP diagram is presented in Figure 1. The phosphorus atom reveals a distorted pyramidal geometry with the average C−P1−C bond angle of 99.26°. The bond angles around the quaternary nitrogen atom N1 show a nearly ideal tetrahedral geometry with an average of 109.43°, while the geometries around the other cage nitrogen atoms N2 and N3 are distorted pyramidal with average C−N−C angles of 106.67° and 111.73°, respectively. The intermolecular interactions between the methylene groups of the cation and the bromide anion (C1−H1B···Br1 2.86(3) Å, C7−H7B···Br1 2.8837 Å, C2−H2B···Br1^i^ 2.94(6) Å, C3−H3A···Br1^i^ 2.93(6) Å, C4−H4B···Br1^ii^ 2.99(4) Å, C6−H6A···Br1^ii^ 2.80(4) Å) lead to a 3D architecture (Figure S67). Intermolecular hydrogen contacts between two cation moieties of **2** was also observed involving an oxygen atom from the nitro group and a hydrogen atom from a cage methylene group (C1−H1A···O2^i^ 2.45(3) Å, i: -x,-y,1-z; Figure S68).

**Figure 2:**
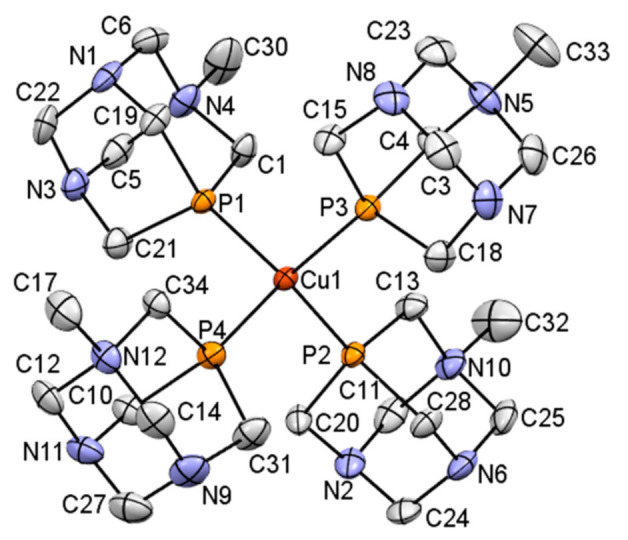
ORTEP diagrams of the cation in **4** with displacement ellipsoids shown at 30% probability level and partial atom numbering schemes. The hydrogen atoms and bromide anions are omitted for clarity.

Complex **4** crystallized in the triclinic space group *P -1*. The asymmetric unit of the compound consists of the [Cu(PTA-Me)_4_]^5+^ cation (Figure 2) balanced with five bromide anions. The Cu(I) centre possesses a tetrahedral geometry (*τ*_4_ = 0.98) [61] filled by four crystallografically independent phosphorus atoms with P−Cu−P bond angles ranging from 106.56(6)° to 111.04(6)° and the average bond angle around the Cu1 atom being 109.46°.

**Figure 3:**
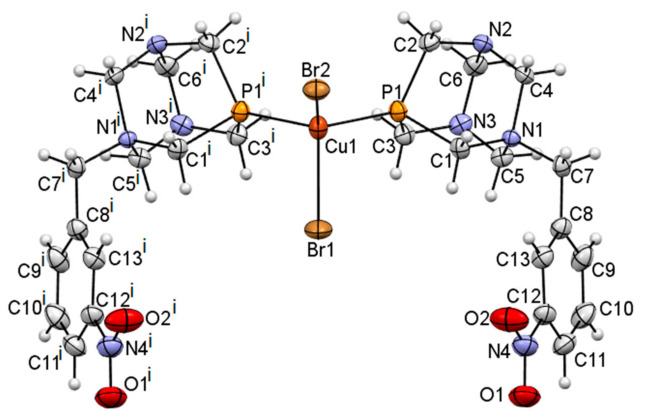
Perspective view of the cation in compound **5a·**3H_2_O with partial atom numbering scheme. Thermal ellipsoids are shown at 40% probability levels. The bromide counter anion and crystallization water molecules are omitted for clarity. Symmetry operation to generate equivalent atoms (i) x,1/2-y, z.

The orthorhombic structure of **5a**·3H_2_O consists of the discrete mononuclear complex [CuBr_2_(PTA-CH_2_-*m*-NO_2_-C_6_H_4_)_2_]^+^ cation (Figure 3), one bromide counter anion balancing its positive charge, and three crystallization water molecules. A crystallographic mirror plane aligned with the *a* axis contains the Cu1 metal centre, the coordinated and free bromide ligands and the crystallization water molecules; the complex cations are stack up along *c.* The Cu(I) cation adopts a slightly distorted tetrahedral geometry (*τ*_4_ = 0.91) [61] constructed from two phosphorus atoms and two bromide anions with the average bond angle around the metal centre being 109.18° and the major deviations from the idealized tetrahedral geometry being of 102.68(3)° (P1−Cu1−Br1) and 116.17(5)° (P1−Cu1−P1^i^). The average C−P1−C bond angles of the phosphanes decreased slightly upon coordination when compared to the free ligand (97.6° for **5a** vs. 99.26° for **2**).

### 3.3. Preparation and Characterization of the Carbon Materials

The carbon materials, AC and CNT, were treated with nitric acid (5M) under reflux to give the oxidized AC-ox and CNT-ox materials, which were subsequently treated with sodium hydroxide (20 mM) to obtain the AC-ox-Na and CNT-ox-Na carbon supports.

It was shown that the different treatments make significant structural modifications to the carbon materials [53,57,62,63]. Table 2 lists the values for the BET surface area, pore volume, and pore size of the commercial AC and CNT materials and their treated forms AC-ox, CNT-ox, AC-ox-Na and CNT-ox-Na. In the case of AC there was a significant decrease in the surface area, pore volume and pore size. Nitric acid treatment has been proved to be effective for the creation of surface oxygen-containing functionalities such as carboxylic groups which could inhibit the access of N_2_ to the micropores [57,64]. The subsequent NaOH treatment (AC-ox-Na) further reduced the surface area of the material. For CNTs, having a cylindrical structure and the pores result from the free space in the bundles, the acid treatment opens the CNT tips and slightly increases the BET surface area (as seen for CNT-ox). Surface oxygen functionalities such as carboxylic groups can be introduced on the outer and possibly the inner walls which result in the formation of edges and steps on the graphene sheets [51,65]. The oxidant effectively opened the carbon nanotubes, and aggregation of the individual CNTs results in a lower value of pore volume and size than in the untreated sample [57,66,67]. The subsequent NaOH treatment (to achieve CNT-ox-Na) which replaces the hydrogen atom with a larger sodium atom appeared to have blocked those slightly opened tips and prevent nitrogen access to the inner cavity of CNTs, hence the obtained lower BET surface area. 

### 3.4. Heterogenization Efficiency

The metal loadings of compounds **5a** and **5b** that were heterogenized on the six carbon materials varied in the 0.2–2.5% range of the initial amount of Cu available for heterogenization. The surface treatments proved to provide significant structural modifications to the carbon supports and to enhance the immobilization process. 

As shown in Table 3, although the different treatments affect the porous structure and surface chemistry of the supports, the obtained materials were able to anchor the Cu complexes, but with different efficiencies. In general, heterogenization of the complexes was better observed in the ox-Na supports and least effective on the untreated original materials, which suggests that the formation of phenolate and carboxylate groups obtained with the -ox-Na treatment can probably explain the higher heterogenization levels considering that these groups may act as coordinating sites for the Cu complexes [52,68]. Such functional groups are usually found attached to the ends of the nanotubes due to the enhanced reactivity of these areas [69,70]. Possible coordination and other interacting modes of Cu(I)-N-alkylated PTA complexes onto the carbon materials are shown in Figures S69–S72.

The immobilization of **5a** and **5b** on the carbon materials can occur by interaction of the Cu(I) complexes with the oxygenated functional groups of the carbon matrix, via possible formation of a Cu-O bond upon displacement of the halo (X) ligand (Figure S69), addition of one of these groups to the Cu atom (Figure S70), or a non-covalent interaction such as H-bonding (Figure S71) or ionic interaction (Figure S72). When compared to their copper(I) analogues bearing a neutral PTA derivative (i.e., 3,7-diacetyl-1,3,7-triaza-5-phosphabicyclo-[3.3.1]nonane) [28], complexes **5a** and **5b** exhibit higher heterogenization values on the same activated carbon materials due to the intermolecular charge interaction between the negatively charged carboxylate groups of the carbon matrix and the positively charged ligand 2 (Figure S72).

Figures S73–S75 show the corresponding FTIR spectra of the CNT-ox-Na support and the heterogenized complexes **5a** and **5b**, respectively. The peak at ~1720–1722 cm*^−^*^1^ concerns the C=O carboxylate group vibrations. By comparing the FTIR spectra before and after heterogenization (Figures S73–S75), an increase in intensity of a peak around 1385 cm^−1^, which corresponds to N-O vibration [71], is observed in both heterogenized materials. This can possibly suggest the involvement of the -NO_2_ groups of complexes **5a** and **5b** in their immobilization on the CNT-ox-Na support.

### 3.5. Catalytic Activity Study for the Azide-Alkyne Cycloaddition

#### 3.5.1. Synthesis of 1,4-disubstituted 1,2,3-triazoles

Initially, the copper complexes **4**–**7** were screened as potential catalysts to identify the most efficient one for the three-component CuAAC reaction of terminal alkynes with in situ generated azides. In a model reaction, ethynylbenzene was reacted with benzyl bromide and sodium azide under low power (30 W) microwave irradiation (MW) at 125 °C and in a H_2_O/MeCN (1:1) mixture. The experiments were performed in the presence of 3 mol% of catalyst **4**–**7** to afford 1-benzyl-4-phenyl-1*H*-1,2,3-triazole as the unique product that was easily isolated by filtration (Table 4). Under the aforementioned conditions, **5b** (followed by **7a**) shows the highest catalytic activity for the reaction when compared to the other complexes, affording the triazole product in 47% yield (Table 4, entry 2). Therefore, this compound was utilized for further optimization reaction studies in the homogeneous phase (Table 5) and was also immobilized on six different carbon materials to be used as heterogeneous catalyst for the same catalytic reaction (Table 6). 

Several experiments were performed in the presence of compound **5b** as a homogeneous catalyst to optimize the reaction conditions. Many reaction parameters were studied, including the amount of catalyst, reaction time and solvent mixture composition and volume. The results are listed in Table S2 and selected ones are given in Table 5. 

The reaction did not proceed under solvent free conditions due to the lack of solubility of sodium azide and the catalyst **5b** in the organic components of the reaction, thus the presence of water in the reaction medium is necessary to dissolve the hydrophilic components of the system. Miscible organic co-solvents were employed to solubilize the organic reactants and to improve the homogeneity of the reaction medium. When 1:1 mixtures of water with different organic polar co-solvents were used (Table 5, entries 1–4), the obtained yield using acetonitrile is higher than that obtained with 1,4-dioxane, DMF or DMSO.

Increasing the reactants concentrations improved the rate of the reaction (Table 5, entries 4–6), where the product yield raised from 47 to 92% by decreasing the total volume of the solvent mixture from 1.5 to 0.5 mL. Extending the reaction time from 15 to 60 min resulted in a slight yield improvement (Table 5, entries 4 and 7), and nearly a quantitative yield of the triazole product was obtained by increasing the catalyst loading to 5 mol% (Table 5, entry 8).

The catalytic activity of the copper complex **5b** immobilized on different carbon materials has been investigated for the one-pot synthesis of 1-benzyl-4-phenyl-1*H*-1,2,3-triazole, and several reaction parameters have been explored to identify the optimum reaction conditions (Table 6). Performing the reaction in the presence of the heterogenized materials with 0.5 mol % of catalyst loading indicated that the AC-based materials (Table 6, entries 1–3) gave lower yields than those of CNT-based supports (Table 6, entries 4–6). Although AC has a higher surface area and is a microporous material (Table 2), the reactants are not so accessible as they are for CNTs, since the latter have a cylindrical structure and the pores result from spaces between the bundles [51,68]. This results in a higher accessibility of the reactants to the active centres. The nature and concentration of surface functional groups or the enhanced interactions with the reactants or intermediate radicals caused by the electronic effects of the graphitic structure of CNT might also be plausible explanations [51,68]. Since the complex **5b** immobilized on CNT-ox-Na (thereafter denoted as **5b**_CNT-ox-Na) gave the best yield for the reaction (Table 6, entry 6), this support was further used to optimize the reaction conditions. Increasing the catalyst loading from 0.5 to 1.2 mol% improved the reaction yield from 25 to 44%, after 15 min at 125 °C (Table 6, entries 6 and 7). In the presence of 1.2 mol% of the catalyst, extending the reaction time from 15 to 60 min significantly increased the yield from 44 to 80% (Table 6, entries 7 and 8). Further extension of the reaction time did not improve the yield. A considerable yield drop was observed when the reaction temperature decreased to 80 °C (Table 6, entries 8 and 9).

The scope of the catalytic methodology has been explored by reacting various substituted benzyl bromides with ethynylbenzene or 1,4-diethynylbenzene in a mixture of water and acetonitrile (1:1), in the presence of catalyst **5b**_CNT-ox-Na (1.2 mol%) under MW at 125 °C for 60 min. All reactions proceeded to afford the corresponding 1,2,3-triazoles, which were isolated by simple filtration, in moderate yields (Table 7). It was generally observed that the presence of an electron withdrawing substituents in benzyl bromide led to the decrease in the product yields when compared to the non-substituted model substrate. With the presence of the electron withdrawing substituent, the yields were according to the following trend: *meta* > *ortho* > *para*. The drop in product yield was also observed when the dialkyne substrate was used instead of the monoalkyne one. A plausible explanation of such a drop in yield is that after the functionalization of the first alkyne group the substrate became bulkier and its accessibility to the active sites of the catalyst was thus hampered. 

**Figure 4:**
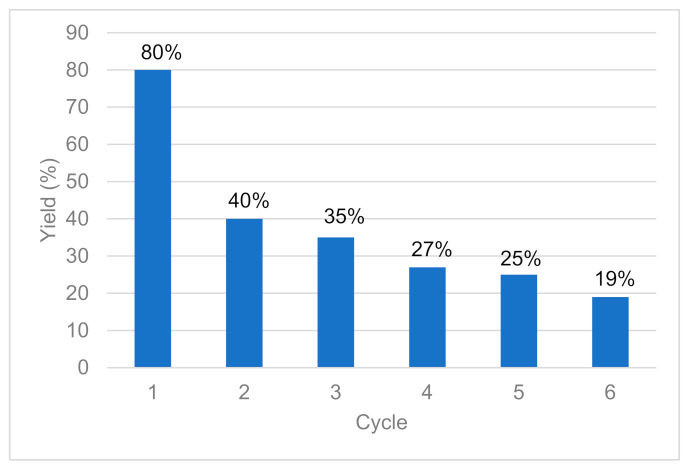
Effect of catalyst **5b**_CNT-ox-Na recycling on the yield of 1-benzyl-4-phenyl-1*H*-1,2,3-triazole.

The recyclability of catalyst **5b**_CNT-ox-Na was assessed using the previously described model reaction (ethynylbenzene, benzyl bromide and sodium azide) with 1.2 mol% of the catalyst, under MW for 60 min at 125 °C. After each reaction cycle, the catalyst was recovered and subjected to the next cycle under identical reaction conditions. As depicted in Figure 4, the catalyst was active and the triazole product was obtained for six consecutive cycles. However, a dramatic drop in the yield was observed after the first cycle from 80 to 40%, then it was gradually decreased to 19% at the sixth reaction cycle. This loss might be attributed to some catalyst leaching during the catalytic experiments. 

In a homogeneous system, catalyst 5b exhibits a high activity towards the CuAAC reaction comparable with those of catalysts bearing polydentate amines [14], N-heterocyclic carbenes [17,18,19], phosphines [13], tris(pyrazolyl) methanes [22] and tris(triazolyl) methanol [20,21] ligands in terms of high obtained yield and regioselectivity. However, **5b** has an important advantage of the high solubility in water, which enables its separation from the obtained organic product by simple washing with water to afford the triazole in a high purity, avoiding the utilization of complicated (e.g., chromatographic) techniques.

To show the advantages of the new carbon-supported catalyst for the synthesis of 1,4-disubstituted 1,2,3-triazoles, a comparative study of the present system with some of the previously reported catalysts is given in Table S3. The catalytic activity of the carbon-supported **5b** complex shows some improvements in comparison to the earlier systems. Copper-in-charcoal (Cu/C) from commercial sources has been employed as a cheap and effective catalysts for CuAAC reaction. In this context, Buckley et al. [72] reported gerhardite [Cu_2_NO_3_(OH)_3_] obtained via carbon impregnation method as a catalyst precursor, which transforms during the CuAAC reaction to the active Cu(I) acetylide polymeric species. The catalytic system afforded triazoles in excellent 92–99% yields within 10–120 min, but the reactions were performed in dioxane using relatively high catalyst loading of 10 mol % and in the presence of triethylamine. Nanoparticles of Cu_2_O immobilized onto graphene oxide (GO) nanosheets were obtained by the reduction of copper(II) acetate impregnated in GO at high temperature (600 °C) in Ar stream [73]. The catalysts Cu(I)/GO proved to be active for the CuAAC reaction with triazole yields in the 80–99% range, but the reactions were performed using deuterated THF as a solvent for a relatively long reaction time of 48 h. Highly dispersed copper nanoparticles (NPs) on reduced graphene oxide (RGO) and CNT were employed for CuAAC, with the former showing a higher catalytic activity and a better recyclability than the latter (triazole product yield decreased from 99 to 91% and 60 to 45% in 10 runs, respectively) [55]. However, an extended reaction time (up to 72 h) and substantial amounts of catalyst (up to 8 mol %) are necessary to obtain good yields. The Cu NPs immobilized on carbon (the carbon material was obtained by pyrolysis of tar) catalytic system gave a 97% yield of the product at 70 °C for 4 h using only 0.5 mol % catalyst loading [74]. Our catalyst is inferior to C/Cu NPs in the sense that it requires a higher temperature (125 °C) but superior in the sense that the catalyst preparation is quite easy and the time of reaction is much shorter (60 min). In our recent publication using Cu(I)-DAPTA complex supported on CNT-ox-Na as catalyst, only 48% yield was obtained at 80 °C for a period of 15 min using 1 mol % catalyst loading [28]. Our present system does not require any reducing agent and the reaction proceeds in high-good yields (99% in homogeneous system using **5b**, and 80% using **5b**_CNT-ox-Na) at low catalyst loading (5 mol% of **5b** or 1.2 mol % of **5b**_CNT-ox-Na). The easy synthesis and the air- and moisture-insensitivity of the complexes are some of the notable features that make it a more convenient catalytic system as compared to most of the previously reported catalysts.

#### 3.5.2. Synthesis of N-hydroxymethyl-1,2,3-triazoles 

For the preparation of these triazoles, the substrate composition of the reaction system was adjusted by replacing the organohalide (benzyl bromide) by formaldehyde (formalin aqueous solution). The catalytic activity of the copper complexes **5**–**7** was thus tested for the one-pot three-component (ethynylbenzene, formaldehyde and sodium azide) CuAAC reaction to determine the most active catalyst. The screening process was performed using 3 mol% of the catalyst, ethynylbenzene, formalin (40% aqueous solution) and sodium azide in 1,4-dioxane, under slightly acidic conditions (pH = 6.5, using acetic acid) for 15 min under MW (30 W) at 125 °C (Table 8). In all cases, the reaction proceeded to afford a regioisomeric mixture of 2-hydroxymethyl-4-phenyl-1,2,3-triazole (**A**) and 1-hydroxymethyl-4-phenyl-1,2,3-triazole (**B**), with the former being the major isomer. Complex **5a** revealed the highest activity with a total yield of 36% (Table 8, entry 1) for the triazole products (**A**+**B**) and was thus used as homogeneous and supported catalyst for the optimization of the reaction conditions

The formation of the hydroxymethyl triazoles was confirmed by ^1^H-NMR on account of the presence of resonance signals at 5.64–5.69 ppm raised from the aliphatic methylene protons (Figure S91). The formation of the regioisomeric mixture was confirmed by the two singlets at 8.61 ppm and 8.30 ppm that are attributed to the triazole ring proton of **B** and **A**, respectively. The ^13^C NMR spectrum exhibited 14 resonances, 7 for each isomer (Figure S92). The identity of the regioisomer products and the assignments of the ^1^H and ^13^C signals of methylene and triazole ring protons were determined by HSQC experiments (Figure S93). 

In order to find the optimum conditions to perform the reaction using catalyst **5a**, different reaction parameters were inspected including the solvent composition, amount of catalyst and reaction time (Table 9). In all experiments, the water is in the reaction medium due to the presence of aqueous formaldehyde (37%). The presence of organic co-solvents is important to dissolve the organic reactant (ethynylbenzene) and to improve the homogeneity of the catalytic system. Performing the reaction in the presence of 1,4-dioxane with the same amount of formalin, maintaining the volumetric ratio of water:co-solvent = 1:1, provided hydroxymethyl triazole with yield 25% after 60 min (Table 9, entry 1). Increasing the amount of water in the reaction medium (i.e., the volumetric ratio of water/dioxane = 2:1, by using formalin/distilled water/dioxane = 1:1:1) to improve the solubility of the hydrophilic components (NaN_3_ and **5a**) had a significant effect on the reaction performance whereby 73% of product yield was obtained after only 15 min (Table 9, entry 2). Several organic co-solvents (viz., 1,4-dioxane, MeCN, DMF and DMSO) were used to study their effect (Table 9, entries 2–5), and the best result was obtained using 1,4-dioxane as the organic co-solvent.

Increasing the reaction time from 15 to 60 min raises the product yield from 73 to 88% (Table 9, entries 2, 6 and 7), while extending the reaction time to 120 min decreased the yield to 75% (Table 9, entry 8). Increasing the water:1,4-dioxane ratio to 4:1 had a negative effect on the product yield (Table 9, compare entries 7 and 9). Decreasing the reaction temperature from 125 to 80 °C led to a significant drop in the reaction yield (Table 9, compare entries 2 and 7 with 10 and 11). A slight diminishing in the yield was observed on raising the catalyst loading from 3 to 5 mol% (Table 9, compare entries 2 and 7 with 12 and 13). 

The possibility of preparing a heterogenized catalyst that can be easily separated from the reaction mixture and regenerated to be recyclable was also studied. In this context, complex **5a** was immobilized on six different carbon supports and their catalytic reactivity for the synthesis of hydroxymethyl-1,2,3-triazole was investigated (Table 10).

In the presence of the heterogenized materials with 2 mol % of catalyst loading, the three-component (ethynylbenzene, formaldehyde and sodium azide) CuAAC reactions were performed in a mixture of water and 1,4-dioxane (2:1), under MW (30 W) at 125 °C for 60 min (Table 10, entries 1–6). All reactions proceeded to afford the regioisomeric mixture of N-hydroxymethyl-1,2,3-triazoles (**A**+**B**), with the complex **5a** immobilized on CNT-ox-Na support (thereafter denoted as **5a**_CNT-ox-Na) being the most active catalyst in terms of the high total yield (Table 10, entry 6). Considering selectivity, using CNT-based materials gave selectivity values comparable to those obtained by the homogeneous system (Table 9), while utilizing AC-based supports slightly improved the selectivity values towards isomer **A**. Increasing the catalyst loading from 0.5 to 2.5 mol % raised the reaction yield from 45 to 93% (Table 10, entries 6–8). Although decreasing the reaction temperature from 125 to 80 °C led to a significant drop in the yield from 93 to 33%, it was observed that the reaction selectivity was improved (Table 10, entries 8 and 9).

**Scheme 7:**
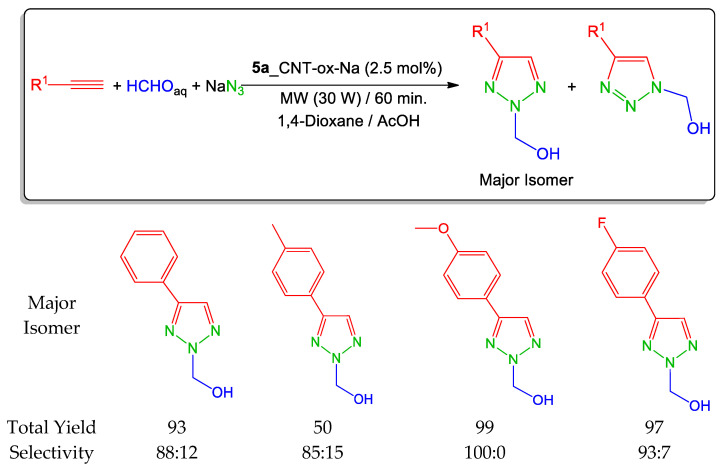
One-pot synthesis of N-hydroxymethyl-1,2,3-triazoles via CuAAC reaction catalysed by **5a**_CNT-ox-Na.

The activity and versatility of **5a**_CNT-ox-Na as a catalyst for the one-pot synthesis of N-hydroxymethyl-1,2,3-triazoles via this CuAAC reaction were probed using various terminal alkynes. As depicted in Scheme 7, all reactions were performed using 2.5 mol% of the catalyst by mixing the terminal alkyne with formaldehyde and sodium azide in water and 1,4-dioxane (2:1), under acidic conditions for 60 min under MW (30 W, 125 °C) to afford the corresponding 1- and 2-hydroxymethyl triazoles with yields ranging from 50% up to quantitative conversion.

**Scheme 8:**
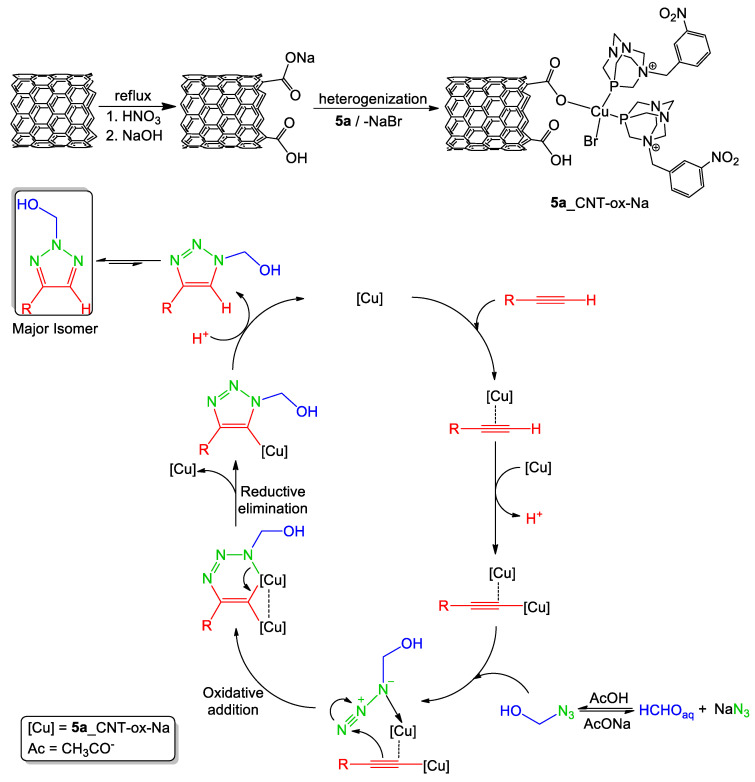
Postulated mechanism of the synthesis of 2-hydroxymethyl-1*H*-1,2,3-triazoles via Cu–AAC reaction. A possible structure of the supported **5a**_CNT-ox-Na catalyst is also presented.

The catalytic synthesis of 2-hydroxymethyl-1,2,3-triazoles using our system should follow the established general pathway for the CuAAC reactions. A conceivable reaction mechanism is depicted in Scheme 8, where several studies that have been performed for its elucidation were considered [75,76,77,78,79]. First, the alkyne coordinates to the Cu(I) centre as a *π*-ligand, and as a result, the C-H acidity increases. Upon deprotonation, a stable *σ*,*π*-di(copper) acetylide complex is formed [23,80]. From the protonated formaldehyde and sodium azide, the azidomethanol (HO-CH_2_-N_3_) intermediate is formed and binds to Cu(I) to produce a bridging dicopper *µ*-acetylide intermediate [77,81]. The acetylide–azide coupling occurs through an oxidative addition process to obtain an intermediate six-membered cupracycle [82], followed by a reductive elimination step to afford the triazolide intermediate. The 1-hydroxymethyl-1*H*-1,2,3-triazole is obtained through the fast protonation of the Cu(I) triazolide, and the active copper species catalyst is regenerated. Due to the instability of the 1-hydroxymethyl triazole, it undergoes rearrangement to the thermodynamically more stable 2-hydroxymethyl isomer [35,36,37].

**Figure 5:**
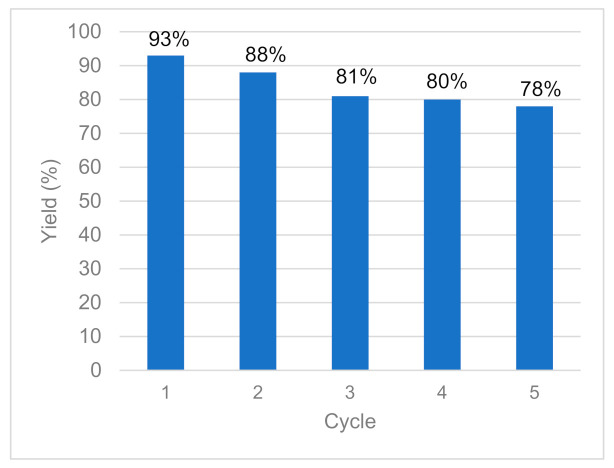
Effect of catalyst **5a**_CNT-ox-Na recycling on the total yield of hydroxymethyl-4-phenyl-1,2,3-triazole.

The recyclability of catalyst **5a**_CNT-ox-Na was investigated by reacting ethynyl benzene with formaldehyde and sodium azide in the presence of 2.5 mol% of the catalyst, in water and 1,4-dioxane (2:1) for 60 min under MW (30 W, 125 °C) for five reaction cycles. As illustrated in Figure 5, the catalyst is able to maintain a moderate activity after five consecutive cycles, where a total product yield of 78% was obtained in the fifth run. 

## 4. Conclusions

In the present study, the N-alkylation of PTA using *ortho-*, *meta-* and *para-*substituted nitrobenzyl bromide led to the formation of ammonium salts **1**–**3**, extending the library of water-soluble phosphanes. Reacting compounds **1**–**3** with simple Cu(I) halide salts using different stoichiometric ratios afforded a new set of water-soluble complexes with various structures that have been studied using ESI(+)MS, IR and NMR spectroscopies, as well as SCXRD (for complexes **4** and **5a**). Among the newly synthesized and fully characterized dibromo-diphosphine complexes [CuBr_2_(PTA-CH_2_-*m*-NO_2_-C_6_H_4_)_2_]X (X = Br {**5a**} or I {**5b**}) were found to be the most active catalysts for the MW assisted CuAAC reaction in homogeneous aqueous system, and thus immobilized on six carbon materials based on AC and CNT. The surface modification of carbon nanotubes brought by oxidation with HNO_3_ and neutralized with NaOH was favourable in terms of complexes heterogenization and catalytic activity. 

Under homogeneous reaction conditions, **5b** proved to be the most active catalyst for the three component CuAAC reaction to obtain 1-benzyl-4-phenyl-1*H*-1,2,3-triazole in excellent yield (99%) after 60 min under MW (30 W, 125 °C). The lower catalytic activity of the tetrakis- and triphosphine complexes (**4**, **6a** and **6b**) can possibly be accounted for by the higher steric effect of the bulky PTA derived ligands. The immobilized catalyst **5b**_CNT-ox-Na was found to be active for the one-pot three-component (alkyne, organohalide and sodium azide) CuAAC reaction to afford 1,4-disubstituted-1,2,3-triazoles in moderate yields up to 80% after 60 min, in a mixture of water and acetonitrile under MW (30 W, 125 °C) using 1.2 mol% of catalyst loading. The catalyst can be recovered and reused up to six consecutive cycles although with loss of activity, where the triazole product yield reduced from 80 to 19% after six runs.

The catalyst **5a**_CNT-ox-Na is an efficient and recyclable catalyst for the one-pot three-component (alkyne, formaldehyde and sodium azide) CuAAC reaction (where formalin is used instead of the organohalide) to afford 2-hydroxymethyl-2*H*-1,2,3-triazoles in excellent yields. Using 2.5 mol% of the catalyst, regioisomeric mixtures of 1- and 2-hydroxymethyl-1,2,3-triazoles were obtained, with the latter being the major isomers, in yields up to 99% after 60 min under MW (30 W, 125 °C). Catalyst **5a**_CNT-ox-Na is recyclable and can be reused for five consecutive cycles maintaining a moderate activity, where a total yield of 78% was obtained in the fifth run. The reusability of the catalyst is one of the most important benefits that makes it useful for potential commercial applications.

The main advantages of the immobilization technique utilized in this study include the simple heterogenization process, the availability of carbon materials and the facile procedures used to modify the carbon supports. The catalytic protocols described in the present study were all performed without requiring an inert atmosphere, in aqueous medium and using simple purification steps to isolate the triazole products as well as supported catalysts, fulfilling the “click reactions” criteria. 

## Figures and Tables

**Table 1:** Selected bond distances (Å) and angles (°) for compounds **2**, **4** and **5a**·3H_2_O ^a^.

2		4		5a·3H_2_O	
P1−C1	1.790(7)	Cu1−P1	2.271(2)	Cu1−P1	2.2282(9)
P1−C2	1.532(9)	Cu1−P2	2.261(1)	Cu1−Br1	2.4583(9)
P1−C3	1.690(8)	Cu1−P3	2.262(1)	Cu1−Br2	2.4283(8)
N1−C1	1.528(4)	Cu1−P4	2.272(2)	P1−C1	1.851(3)
N1−C4	1.522(4)	N4−C30	1.49(1)	P1−C2	1.837(4)
N1−C6	1.543(4)	N5−C33	1.50(1)	P1−C3	1.839(4)
N1−C7	1.514(4)	N10−C32	1.51(2)	N1−C1	1.503(4)
N2−C2	1.77(2)	N12−C17	1.48(1)	N1−C4	1.548(4)
N2−C4	1.52(2)	P1−Cu1−P2	110.52(6)	N1−C5	1.546(4)
N2−C5	1.59(2)	P1−Cu1−P3	111.04(6)	N1−C7	1.512(4)
N3−C3	1.465(5)	P1−Cu1−P4	106.56(6)	P1−Cu1−P1^i^	116.17(5)
N3−C5	1.458(4)	P2−Cu1−P3	109.81(6)	P1−Cu1−Br1	102.68(3)
N3−C6	1.423(5)	P2−Cu1−P4	108.42(6)	P1−Cu1−Br2	113.40(3)
C7−C8	1.504(5)	P3−Cu1−P4	110.41(6)	Br1−Cu1−Br2	106.76(3)
C1−P1−C2	99.4(4)	C1−P1−C19	98.6(3)	C1−P1−C2	97.8(2)
C1−P1−C3	93.3(4)	C1−P1−C21	98.2(3)	C1−P1−C3	96.6(2)
C2−P1−C3	105.1(5)	C19−P1−C21	97.9(3)	C2−P1−C3	98.4(2)
C1−N1−C4	109.9(2)	C13−P2−C20	98.6(3)	C1−N1−C4	110.2(2)
C1−N1−C6	108.6(2)	C13−P2−C28	97.9(3)	C1−N1−C5	110.4(2)
C1−N1−C7	112.6(2)	C20−P2−C28	98.5(3)	C1−N1−C7	111.7(2)
C4−N1−C6	107.9(2)	C4−P3−C15	98.0(3)	C4−N1−C5	106.6(2)
C4−N1−C7	107.0(2)	C4−P3−C18	98.1(3)	C4−N1−C7	106.2(2)
C6−N1−C7	110.6(2)	C15−P3−C18	98.0(3)	C5−N1−C7	111.6(2)
C2−N2−C4	105(1)	C10−P4−C31	97.8(4)	C2−N2−C6	111.8(3)
C2−N2−C5	103(1)	C10−P4−C34	97.9(3)	C2−N2−C4	112.7(3)
C4−N2−C5	112(1)	C31−P4−C34	98.6(3)	C4−N2−C6	109.8(3)
C3−N3−C5	112.1(3)			C3−N3−C5	112.7(3)
C3−N3−C6	111.4(3)			C3−N3−C6	112.0(3)
C5−N3−C6	111.7(3)			C5−N3−C6	110.7(3)
N1−C7−C8	115.0(3)			N1−C7−C8	115.2(3)

^a^ Symmetry operation to generate the i equivalent P1 is x,1/2-y,z.

**Table 2:** Characterization of carbon materials using BET analysis.

Carbon Material	*S*_BET_ (m^2^ g^−1^)	Pore Volume (cm^3^ g^−1^)	Pore Size (nm)
AC	866	0.45	5.2
AC-ox	223	0.22	5.0
AC-ox-Na	191	0.15	4.9
CNT	257	2.44	30.8
CNT-ox	301	1.62	19.0
CNT-ox-Na	126	1.03	15.5

**Table 3:** Copper loading (wt %) on the carbon materials ^a^.

Carbon Material	Cu Complex	
	5a	5b
AC	1.61	0.21
AC-ox	1.60	1.05
AC-ox-Na	2.16	2.53
CNT	1.59	1.73
CNT-ox	1.56	1.64
CNT-ox-Na	1.87	2.2

^a^ Results obtained from ICP analysis.

**Table 4:** Primary screening of catalysts **4**–**7** for the three-component catalytic CuAAC reaction of ethynylbenzene, benzyl bromide and sodium azide, under MW ^a^.

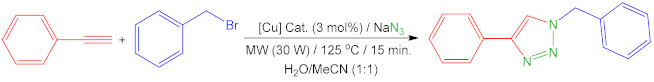			
**Entry**	**Catalyst**	**Yield ^b^** **(%)**	**TON ^c^**
1	**4**	22	8
2	**5a**	38	13
3	**5b**	47	16
4	**6a**	39	13
5	**6b**	35	12
6	**7a**	44	15
7	**7b**	27	9

^a^ Reaction conditions: benzyl bromide (0.3 mmol), ethynylbenzene (0.3 mmol), NaN_3_ (0.3 mmol), 1.5 mL of solvent mixture H_2_O/MeCN (1:1 *v*/*v*), MW irradiation (30 W), at 125 °C. ^b^ Isolated yield. ^c^ Turnover number (moles of product per mole of catalyst).

**Table 5:** Selected data for the optimization study of **5b** as a homogeneous catalyst for the three-component CuAAC reaction of ethynylbenzene, benzyl bromide and sodium azide to produce 1-benzyl-4-phenyl-1*H*-1,2,3-triazole ^a^.

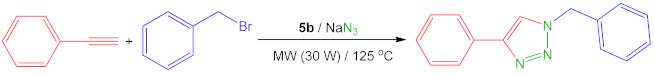						
**Entry**	**Catalyst Loading** **(mol%)**	**Time** **(min.)**	**Solvent**	**Total Volume of Solvent Mixture** **(mL)**	**Yield ^b^** **(%)**	**TON ^c^**
1	3	15	H_2_O:1,4-dioxane	1.0	71	24
2	3	15	H_2_O:DMF	1.0	51	17
3	3	15	H_2_O:DMSO	1.0	56	19
4	3	15	H_2_O:MeCN	1.0	84	28
5	3	15	H_2_O:MeCN	1.5	47	16
6	3	15	H_2_O:MeCN	0.5	92	31
7	3	60	H_2_O:MeCN	1.0	89	30
8	5	60	H_2_O:MeCN	0.5	99	20

^a^ Reaction conditions: benzyl bromide (0.3 mmol), ethynylbenzene (0.3 mmol), NaN_3_ (0.3 mmol), catalyst **5b**, solvent mixture H_2_O/organic (1:1 *v*/*v*), MW irradiation (30 W), at 125 °C. ^b^ Isolated yield. ^c^ Turnover number (moles of product per mole of catalyst).

**Table 6:** The three-component (ethynylbenzene, benzyl bromide, sodium azide) CuAAC reaction catalyzed by **5b** complex anchored on different carbon supports ^a^.

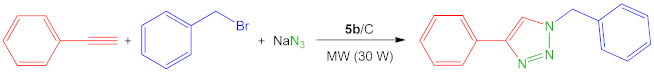						
**Entry**	**Carbon Support**	**Catalyst Loading** **(mol %)**	**Time** **(min.)**	**Temperature** **(**°**C)**	**Yield ^b^** **(%)**	**TON ^c^**
1	AC	0.5	15	125	13	25
2	AC-ox	0.5	15	125	7	14
3	AC-ox-Na	0.5	15	125	10	20
4	CNT	0.5	15	125	24	47
5	CNT-ox	0.5	15	125	23	46
6	CNT-ox-Na	0.5	15	125	25	50
7	CNT-ox-Na	1.2	15	125	44	21
8	CNT-ox-Na	1.2	60	125	80	65
9	CNT-ox-Na	1.2	60	80	41	34

^a^ Reaction conditions: benzyl bromide (0.3 mmol), ethynylbenzene (0.3 mmol), NaN_3_ (0.3 mmol), 1.5 mL of solvent mixture H_2_O/MeCN (1:1 *v*/*v*), MW irradiation (30 W). ^b^ Isolated yield. ^c^ Turnover number (moles of product per moles of catalyst).

**Table 7:** One-pot synthesis of 1,4-disubstituted-1,2,3-triazoles via CuAAC reaction catalysed by **5b**_CNT-ox-Na ^a^.

Entry	Alkyne	Benzyl Bromide	Product	Yield ^b^
1	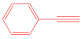	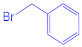	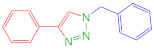	80
2	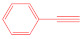	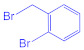	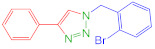	65
3	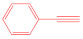	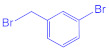	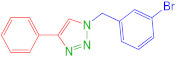	68
4	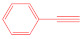	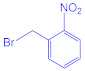	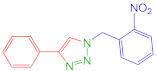	48
5	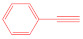	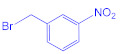	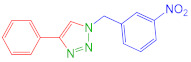	76
6	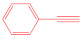	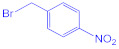	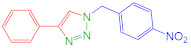	29
7 ^c^	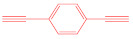	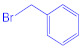	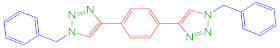	43
8 ^c^	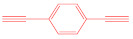	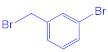	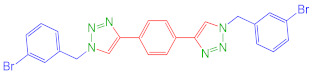	31

^a^ Reaction conditions: Organo halide (0.3 mmol), alkyne (0.3 mmol), NaN_3_ (0.3 mmol), catalyst **5b**_CNT-ox-Na (1.2 mol%), 1.5 mL of solvent mixture H_2_O/MeCN (1:1 *v*/*v*), MW irradiation (30 W, 125 °C for 60 min.). ^b^ Isolated yield. ^c^ Organo halide (0.6 mmol), 1,4-diethynylbenzene (0.33 mmol) and sodium azide (0.62 mmol).

**Table 8:** Primary screening of catalysts **5**–**7** for the three-component catalytic CuAAC reaction of ethynylbenzene, formaldehyde and sodium azide, under MW ^a^.

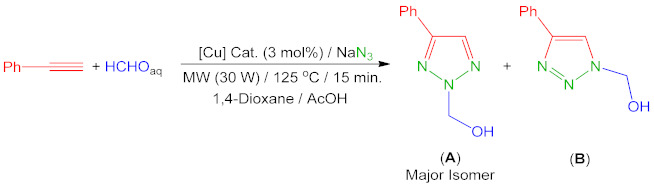				
**Entry**	**Catalyst**	**Yield ^b^** **(%)**	**Molar Ratio ^c^** **(A:B)**	**TON ^d^**
1	**5a**	36	82:18	12
2	**5b**	13	81:19	4
3	**6a**	24	85:15	8
4	**6b**	32	83:17	10
5	**7a**	18	83:17	6
6	**7b**	25	84:16	8

^a^ Reaction conditions: ethynylbenzene (0.6 mmol, 1 equiv.), formaldehyde (6 mmol, 10 equiv.), sodium azide (0.9 mmol, 1.5 equiv.), glacial AcOH (0.9 mmol, 1.5 equiv.), 1,4-dioxane (0.5 mL), catalyst (3 mol%, based on the alkyne), MW irradiation (30 W), at 125 °C. ^b^ Total yield (**A**+**B**) isolated by filtration, calculated on the basis of ethynylbenzene. ^c^ Determined via ^1^H-NMR by integration of the triazole proton (Figure S91). ^d^ Total number of moles of product (**A**+**B**) per mol of catalyst.

**Table 9:** Optimization study of **5a** as a homogeneous catalyst for the three-component catalytic CuAAC reaction of ethynylbenzene, formaldehyde and sodium azide to produce 1- and 2-hydroxymethyl-4-phenyl-1,2,3-triazole regioisomeric mixture ^a^.

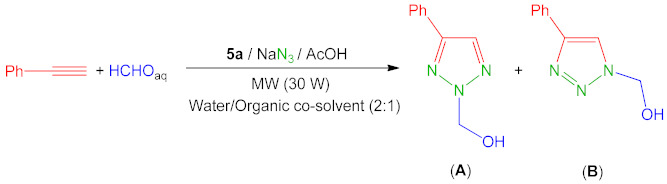							
**Entry**	**Catalyst Loading** **(mol %)**	**Time** **(min)**	**Temperature** **(**°**C)**	**Solvent**	**Yield ^b^** **(%)**	**Molar Ratio (A:B) ^c^**	**TON ^d^**
1 ^e^	3	60	125	H_2_O/dioxane	25	85:15	8
2	3	15	125	H_2_O/dioxane	73	84:16	24
3	3	15	125	H_2_O/MeCN	47	86:14	16
4	3	15	125	H_2_O/DMF	48	83:17	16
5	3	15	125	H_2_O/DMSO	68	84:16	23
6	3	30	125	H_2_O/dioxane	78	84:16	26
7	3	60	125	H_2_O/dioxane	88	88:12	29
8	3	120	125	H_2_O/dioxane	75	85:15	25
9 ^f^	3	60	125	H_2_O/dioxane	75	84:16	25
10	3	15	80	H_2_O/dioxane	5	85:15	2
11	3	60	80	H_2_O/dioxane	57	83.17	19
12	5	15	125	H_2_O/dioxane	69	85:15	14
13	5	60	125	H_2_O/dioxane	85	84:16	17

^a^ Reaction conditions: ethynylbenzene (0.6 mmol, 1 equiv.), formaldehyde (6 mmol, 10 equiv.), sodium azide (0.9 mmol, 1.5 equiv.), glacial AcOH (0.9 mmol, 1.5 equiv.), catalyst **5a**, solvent mixture water/organic (2:1 *v*/*v*), MW irradiation (30 W). ^b^ Total yield isolated by filtration. ^c^ Determined via ^1^H-NMR. ^d^ Total number of moles of product (**A**+**B**) per mol of catalyst. ^e^ Reaction performed in a solvent mixture of water/1,4-dioxane with 1:1 volumetric ratio. ^f^ Reaction performed in a solvent mixture of water/1,4-dioxane with 4:1 volumetric ratio.

**Table 10:** Three-component (ethynylbenzene, aqueous formaldehyde and sodium azide) CuAAC reaction catalyzed by **5a** complex anchored on different carbon supports ^a^.

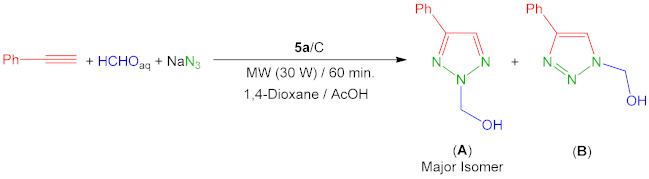						
**Entry**	**Carbon Support**	**Catalyst Loading** **(mol %)**	**Temperature** **(**°**C)**	**Yield ^b^** **(%)**	**Molar Ratio (A:B) ^c^**	**TON ^d^**
1	AC	2	125	48	91:09	23
2	AC-ox	2	125	71	90:10	34
3	AC-ox-Na	2	125	28	94:06	14
4	CNT	2	125	17	85:15	8
5	CNT-ox	2	125	39	85:15	19
6	CNT-ox-Na	2	125	86	88:12	42
7	CNT-ox-Na	0.5	125	45	88:12	92
8	CNT-ox-Na	2.5	125	93	88:12	38
9	CNT-ox-Na	2.5	80	33	95:05	13

^a^ Reaction conditions: ethynylbenzene (0.6 mmol, 1 equiv.), formaldehyde (6 mmol, 10 equiv.), sodium azide (0.9 mmol, 1.5 equiv.), glacial AcOH (0.9 mmol, 1.5 equiv.), 1.5 mL of solvent mixture H_2_O/1,4-dioxane (2:1 *v*/*v*), MW irradiation (30 W). ^b^ Total yield (**A**+**B**) isolated by filtration, calculated on the basis of ethynylbenzene. ^c^ Determined via ^1^H-NMR. ^d^ Total number of moles of product (**A**+**B**) per mol of catalyst.

## Data Availability

Not applicable.

## Supplementary Materials

The following are available online at https://www.mdpi.com/article/10.3390/nano11102702/s1, Figures S1–S66: NMR spectra of compounds **1**–**7**, Table S1: Crystallographic data and structure refinement details for **2** and **3**, Figures S67–S68: Intermolecular interactions in compound **2**, Figures S69–S72: Plausible anchorage of cationic Cu(I) complexes [CuBr_2_(PTA-CH_2_-*m*-NO_2_-C_6_H_4_)_2_]^+^ on carbon materials, Figures S73–S75: FTIR spectra of CNT-ox-Na materials, Figures S76–S99: NMR spectra of triazole products, Figures S100–S108: ESI-MS (positive mode) spectra of triazole products, Table S2: Optimization parameters for **5b** complex as a homogeneous catalyst for the microwave-assisted synthesis of 1-benzyl-4-phenyl-1H-1,2,3-triazole, Table S3: Comparison of the present system with other reported C-supported catalysts for the synthesis of 1,4-disubstituted 1,2,3-triazoles.
