# Supplementary material for: Synthesis of a Novel Series of Cu(I) Complexes Bearing Alkylated 1,3,5-Triaza-7-phosphaadamantane as Homogeneous and Carbon-Supported Catalysts for the Synthesis of 1- and 2-Substituted-1,2,3-triazoles
Source: Nanomaterials (Basel). 2021 Oct 13;11(10):2702. doi: 10.3390/nano11102702 (PMC8537716; doi:10.3390/nano11102702)
Supplement: Supplementary file 1 [file nanomaterials-11-02702-s001.zip › nanomaterials-1410249-supplementary.pdf]

Electronic Supplementary Materials

Synthesis of a Novel Series of Cu(I) Complexes Bearing Alkylated 1,3,5-Triaza-7-phosphaadamantane as Homogeneous and Carbon-Supported Catalysts for the Synthesis of 1- and 2-Substituted-1,2,3-triazoles

Ivy L. Librando [1], Abdallah G. Mahmoud [1,2,*], Sónia A. C. Carabineiro [1,3], M. Fátima C. Guedes da Silva [1], Carlos F. G. C. Geraldes [4,5] and Armando J. L. Pombeiro [1,6]

[1] Centro de Química Estrutural, Instituto Superior Técnico, Universidade de Lisboa, Av. Rovisco Pais, 1049-001 Lisboa, Portugal; ivy.librando@tecnico.ulisboa.pt (I.L.L.); sonia.carabineiro@fct.unl.pt (S.A.C.C.); fatima.guedes@tecnico.ulisboa.pt (M.F.C.G.d.S.); pombeiro@tecnico.ulisboa.pt (A.J.L.P.)
[2] Department of Chemistry, Faculty of Science, Helwan University, Ain Helwan, Cairo 11795, Egypt
[3] LAQV-REQUIMTE, Department of Chemistry, NOVA School of Science and Technology, Universidade NOVA de Lisboa, 2829-516 Caparica, Portugal
[4] Coimbra Chemistry Center, University of Coimbra, Rua Larga Largo D. Dinis, 3004-535 Coimbra, Portugal; geraldes@uc.pt
[5] Department of Life Sciences, Faculty of Science and Technology, Calçada Martim de Freitas, 3000-393 Coimbra, Portugal
[6] Research Institute of Chemistry, Peoples' Friendship University of Russia (RUDN University), 6 Miklukho-Maklaya Street, 117198 Moscow, Russia
* Correspondence: abdallah.mahmoud@tecnico.ulisboa.pt

Keywords: copper complexes; P-ligands; PTA; carbon nanotubes; click chemistry; CuAAC

Contents

1. NMR Spectra of Compounds 1–7 2
2. X-Ray Data and Analysis 59
3. Plausible Anchorage of Cationic Cu(I) Complexes $[CuBr_2(PTA\text{-}CH_2\text{-}m\text{-}NO_2\text{-}C_6H_4)_2]^+$ on Carbon Materials 60
4. FTIR Spectra of CNT-ox-Na Materials (2000–400 cm^{-1}) 63
5. NMR Spectra of Triazole Products 65
6. ESI-MS (Positive Mode) Spectra of Triazole Products 88

1. NMR Spectra of Compounds 1–7

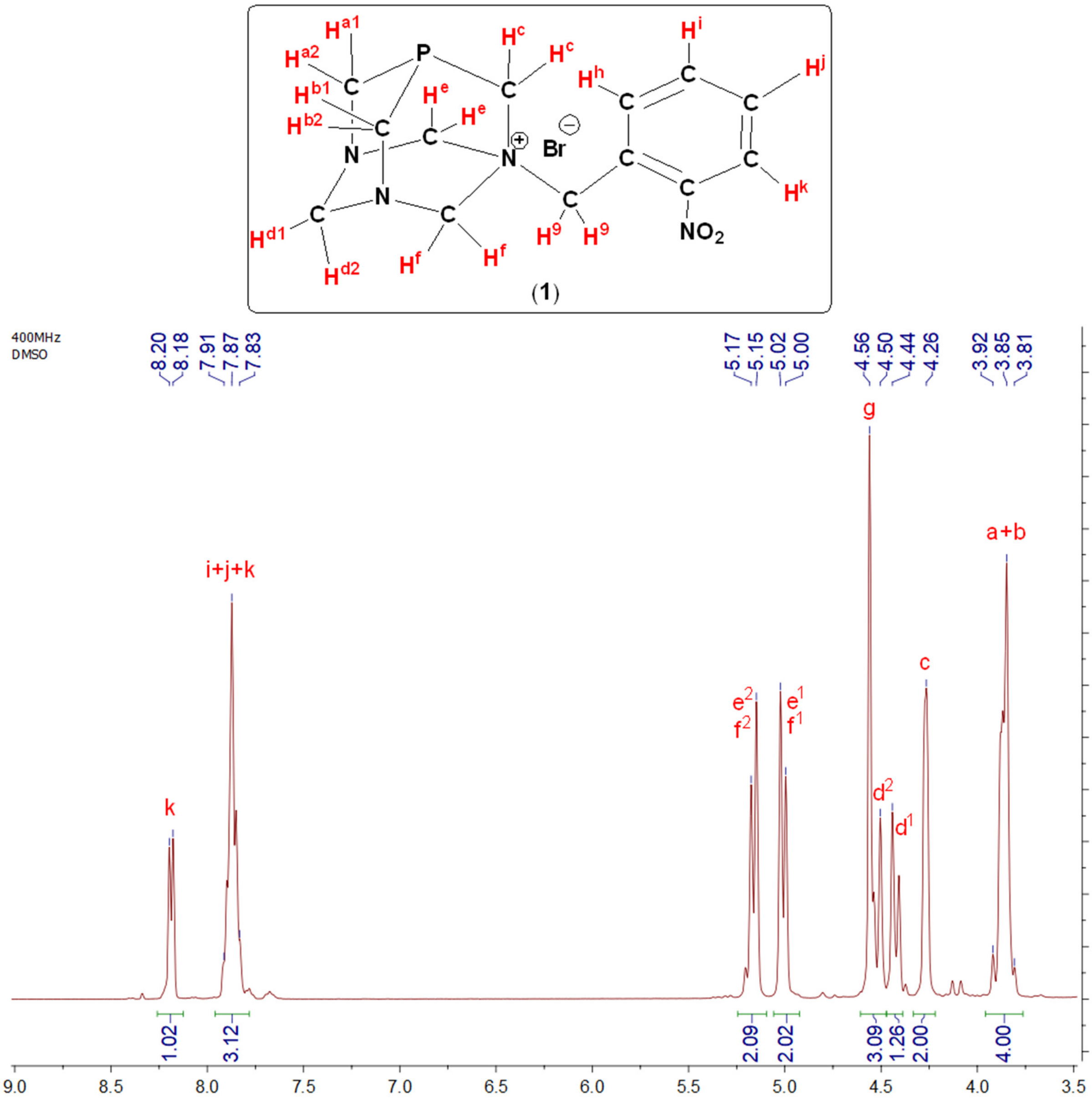

Figure S1. ^{1}H NMR spectrum of (PTA-CH_2-*o*-NO_2-C_6H_4)Br (**1**) in DMSO-d_6 (400 MHz).

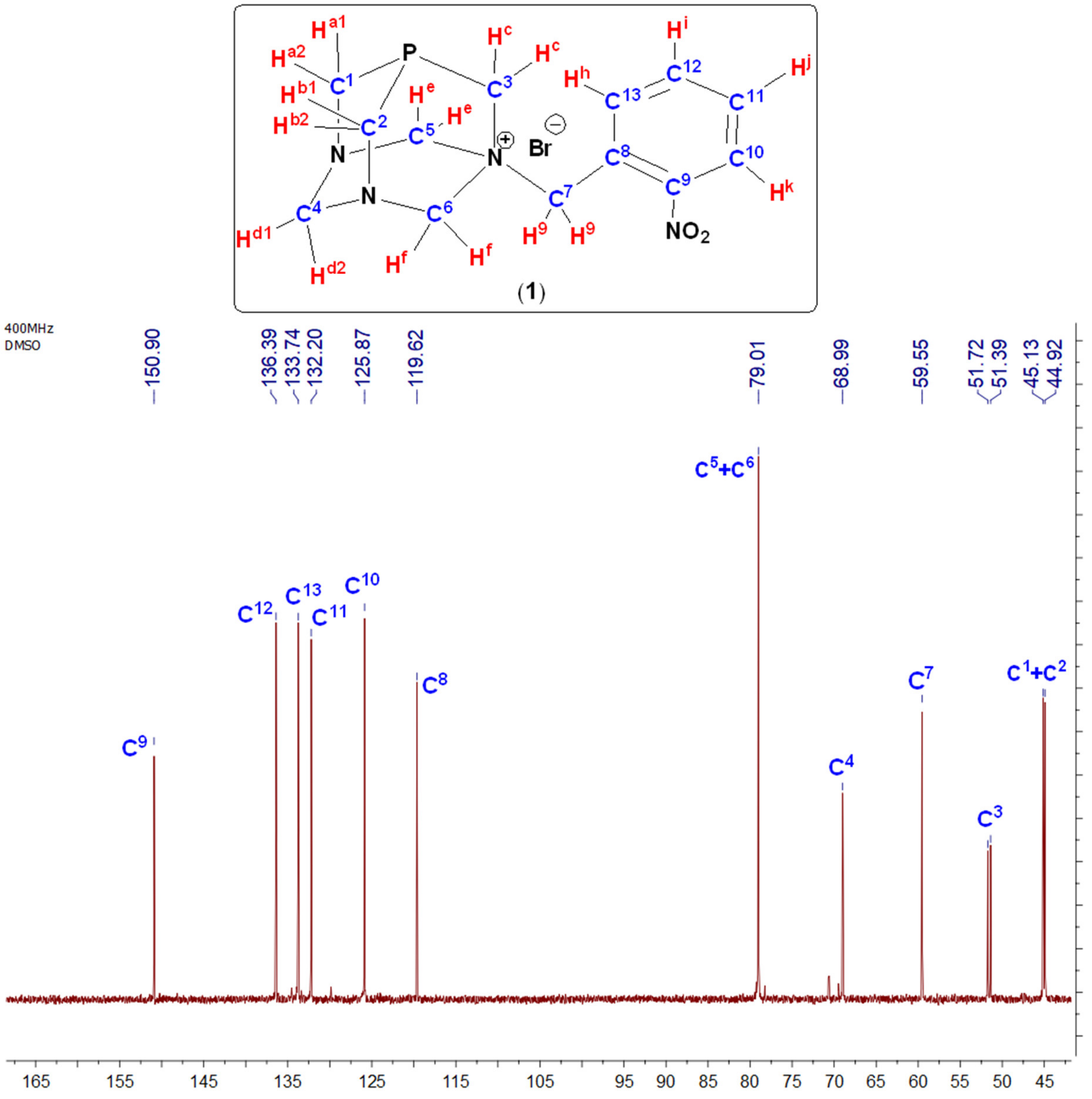

Figure S2. ^{13}C NMR spectrum of (PTA-CH_2-*o*-NO_2-C_6H_4)Br (**1**) in DMSO-d_6 (400 MHz).

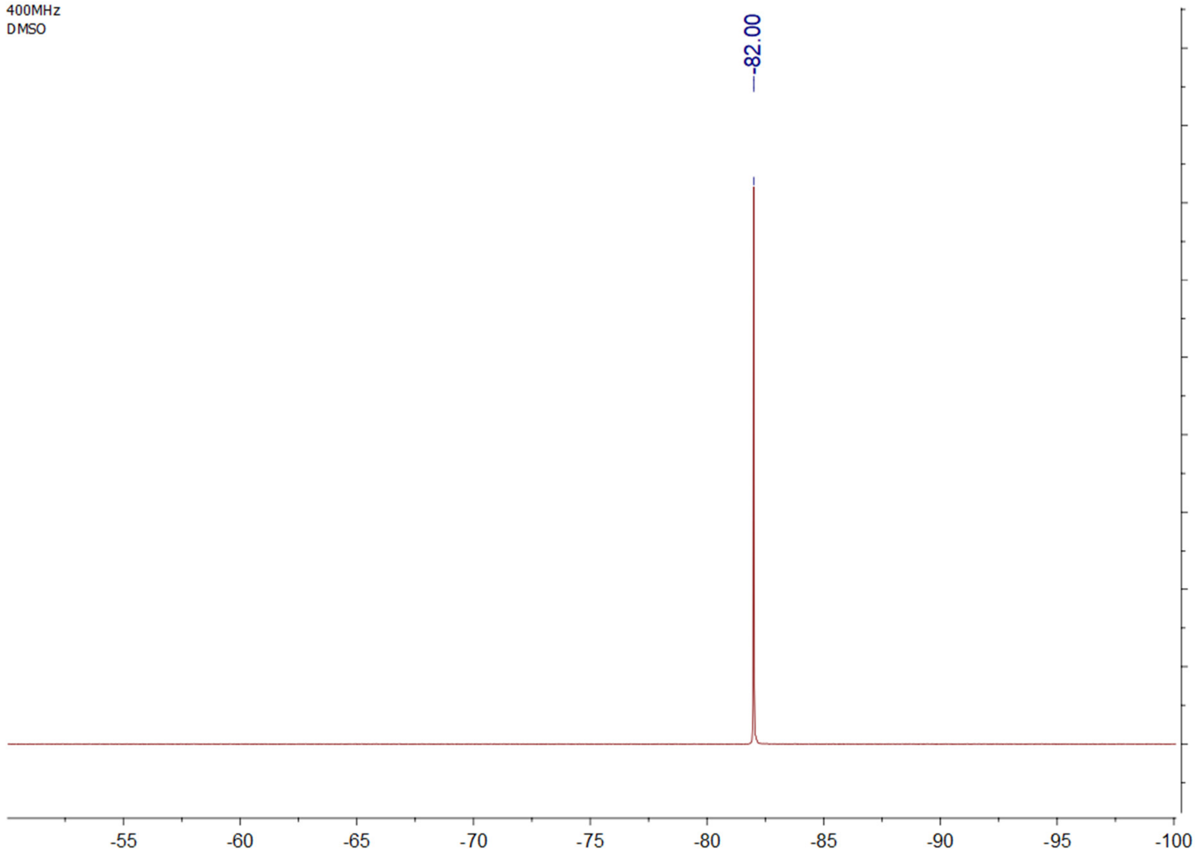

Figure S3. ^{31}P NMR spectrum of (PTA-CH_2-*o*-NO_2-C_6H_4)Br (**1**) in DMSO-d_6 (400 MHz).

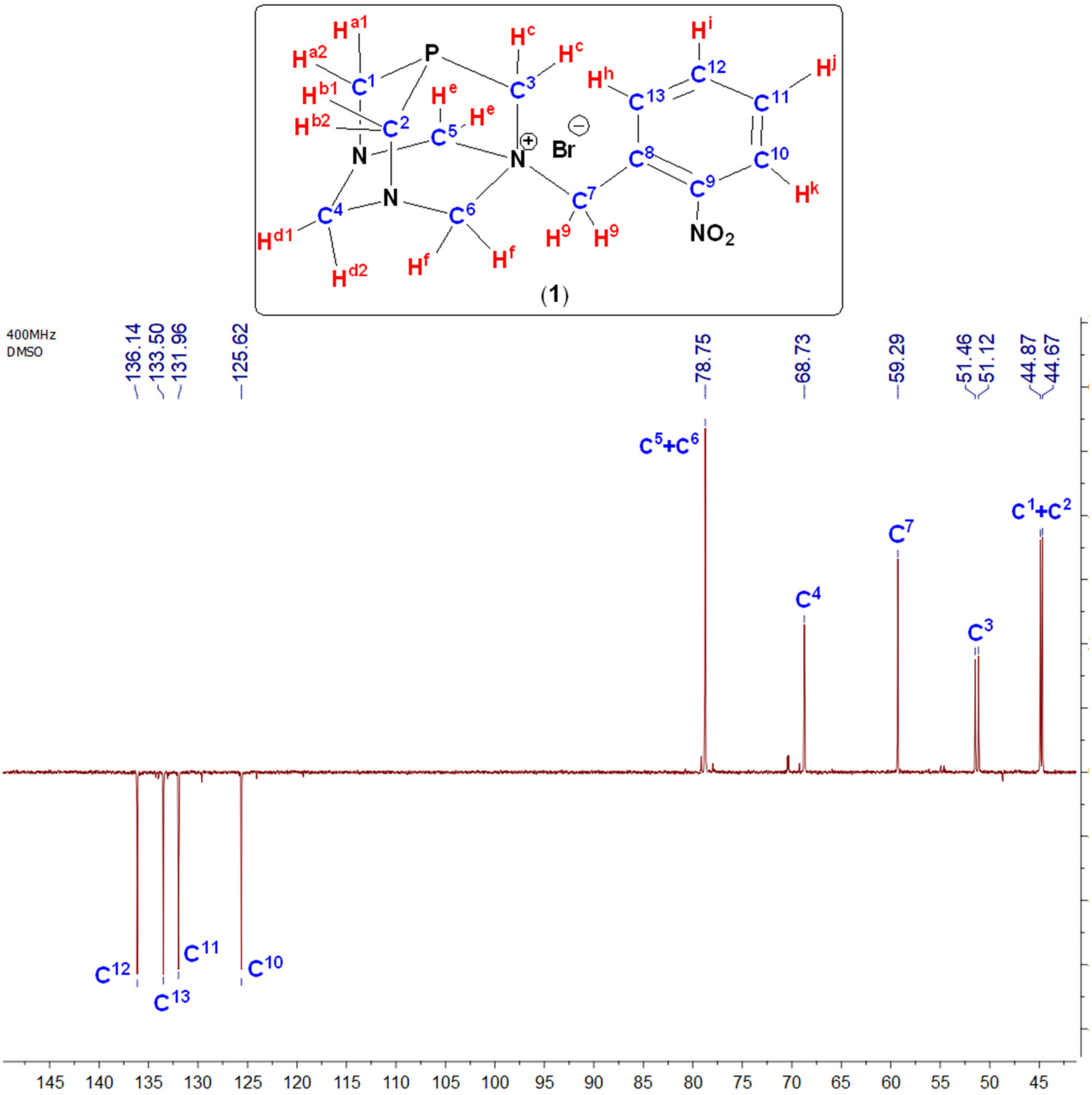

Figure S4. DEPT NMR spectrum of (PTA-CH$_2$-*o*-NO$_2$-C$_6$H$_4$)Br (**1**) in DMSO-*d*$_6$ (400 MHz).

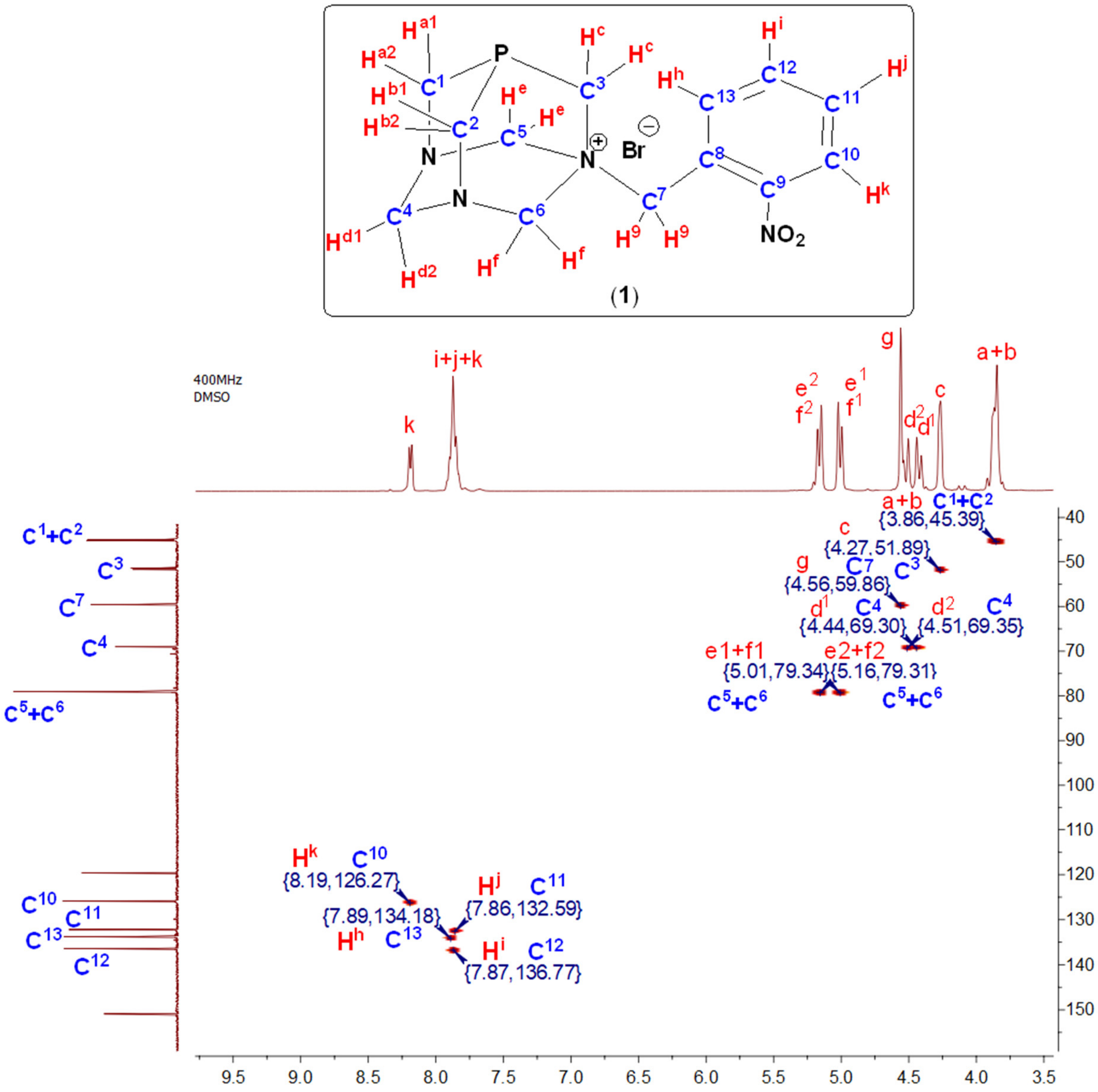

Figure S5. HSQC spectrum of (PTA-CH_2-*o*-NO_2-C_6H_4)Br (**1**) in DMSO-d_6 (400 MHz).

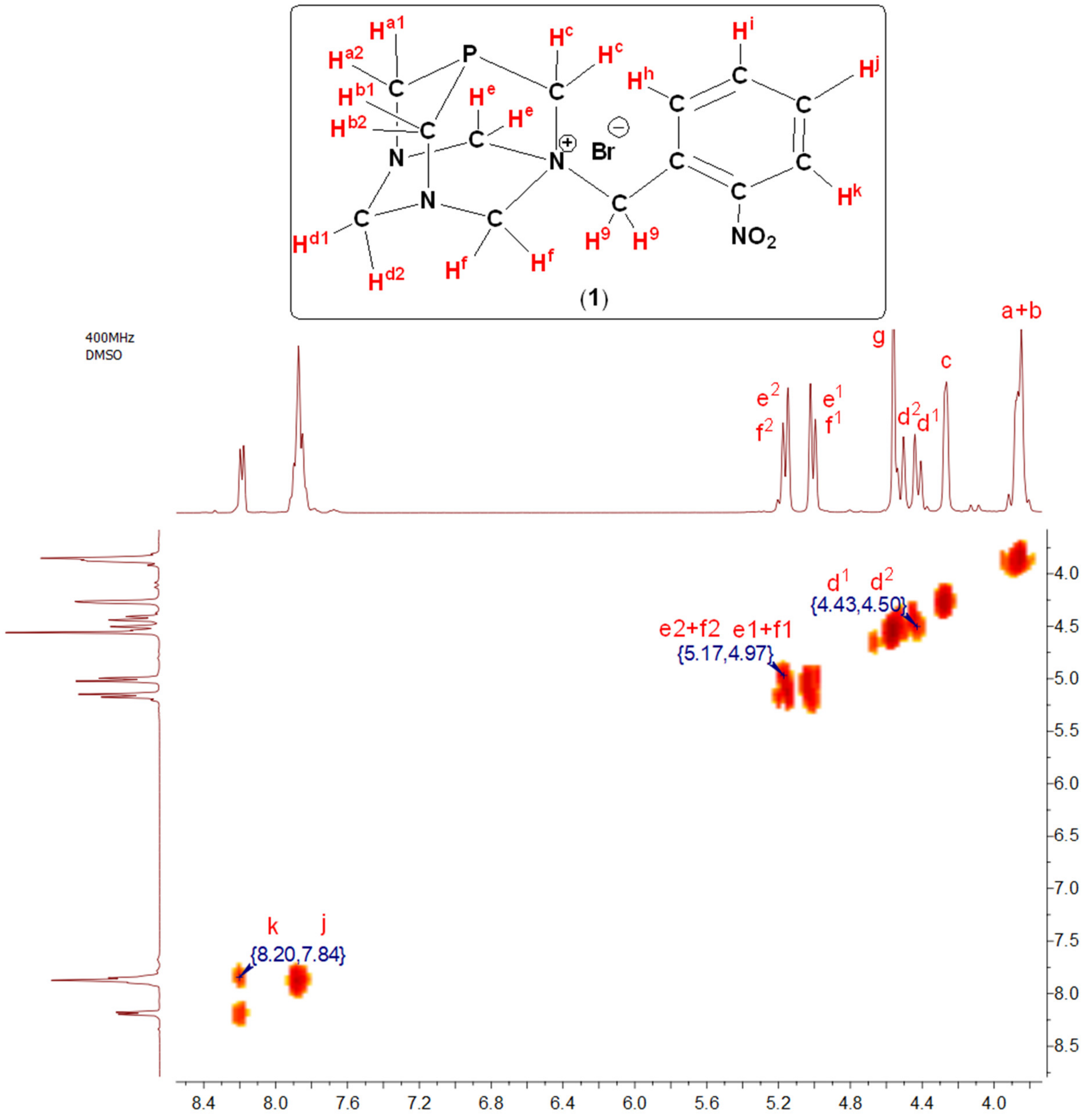

Figure S6. COSY spectrum of (PTA-CH_2-*o*-NO_2-C_6H_4)Br (**1**) in DMSO-d_6 (400 MHz).

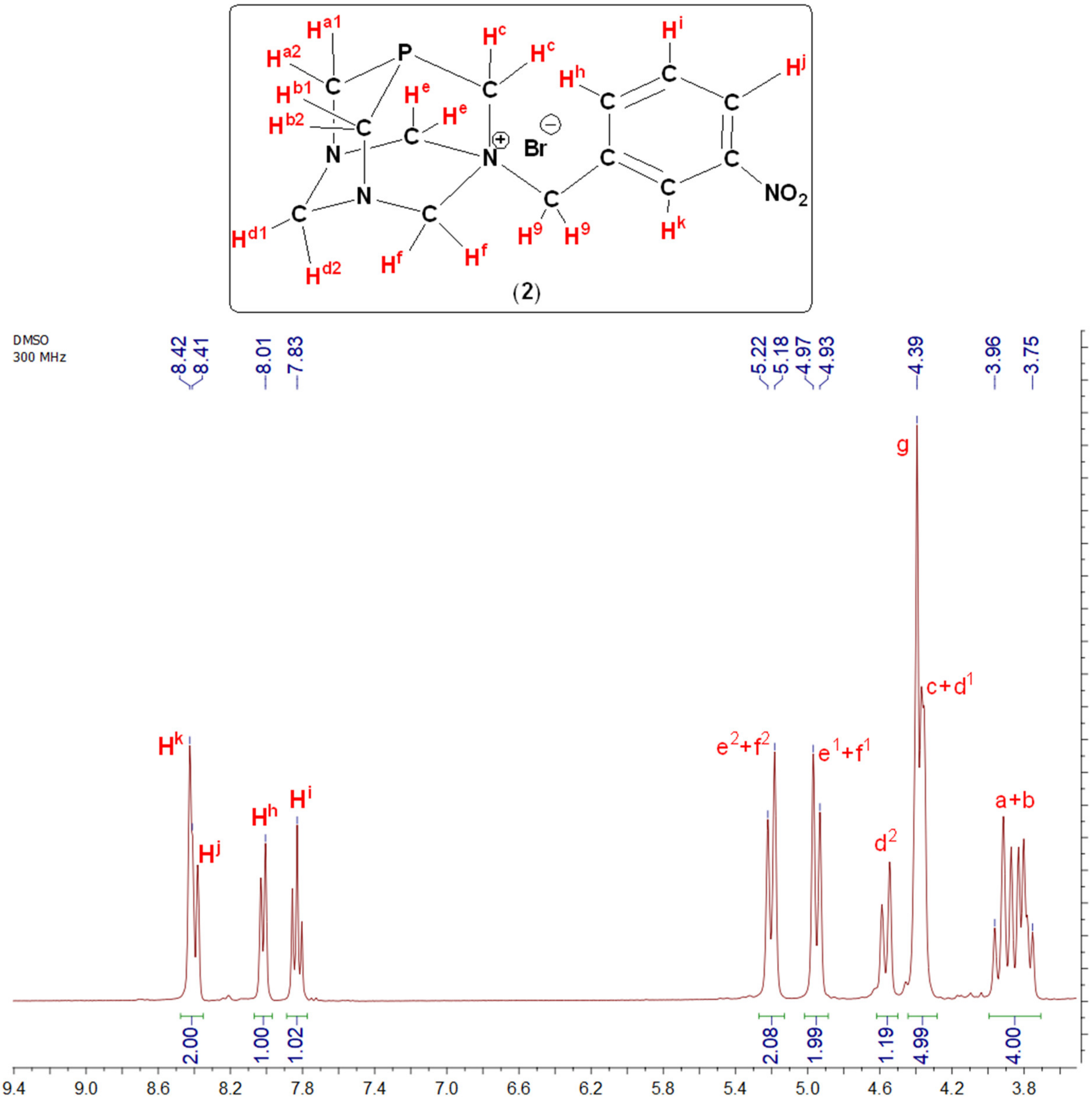

Figure S7. ${}^{1}H$ NMR spectrum of (PTA-CH_2-*m*-NO_2-C_6H_4)Br (**2**) in DMSO-d_6 (300 MHz).

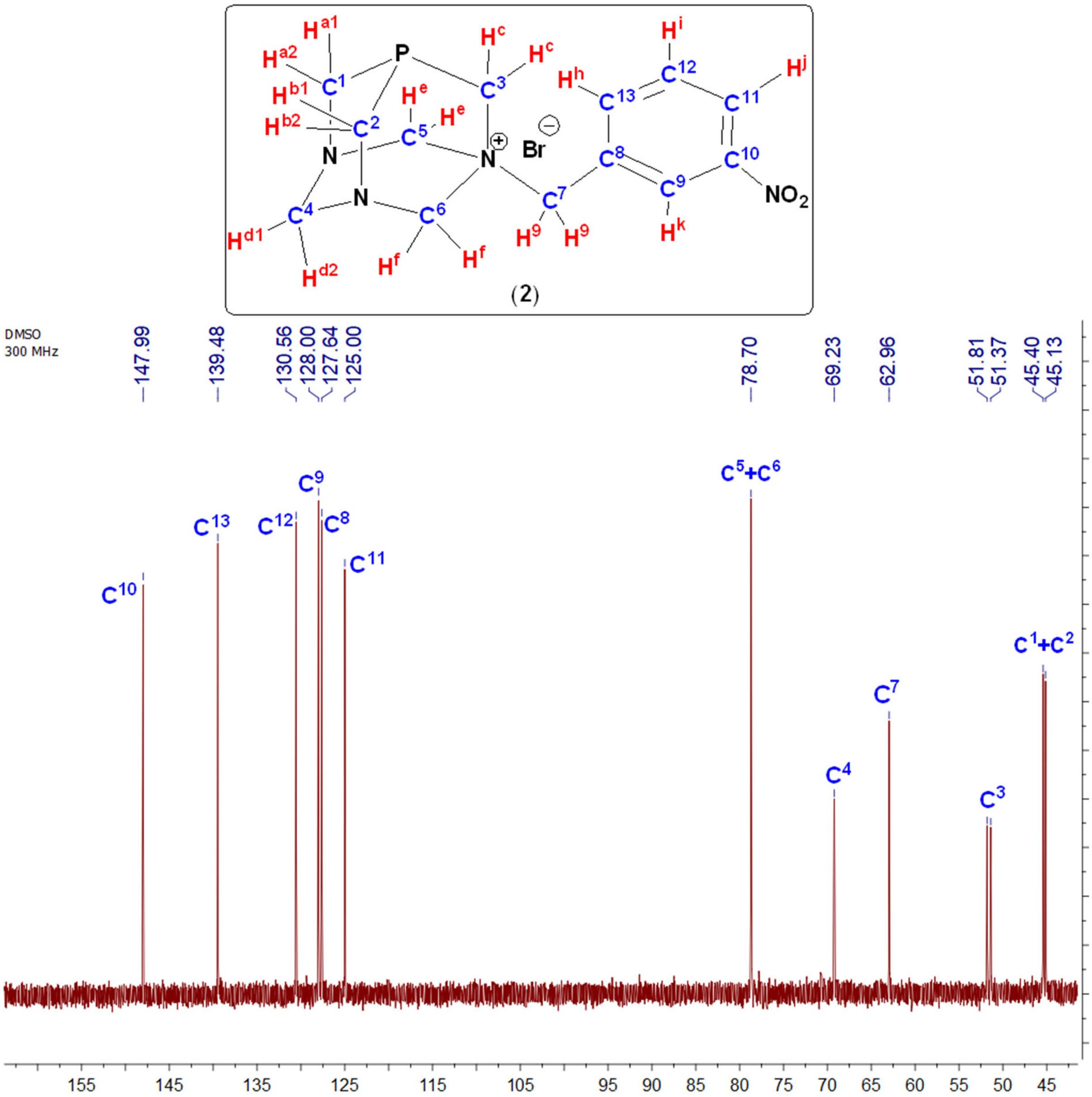

Figure S8. ^{13}C NMR spectrum of (PTA-CH_2-*m*-NO_2-C_6H_4)Br (**2**) in DMSO-d_6 (300 MHz).

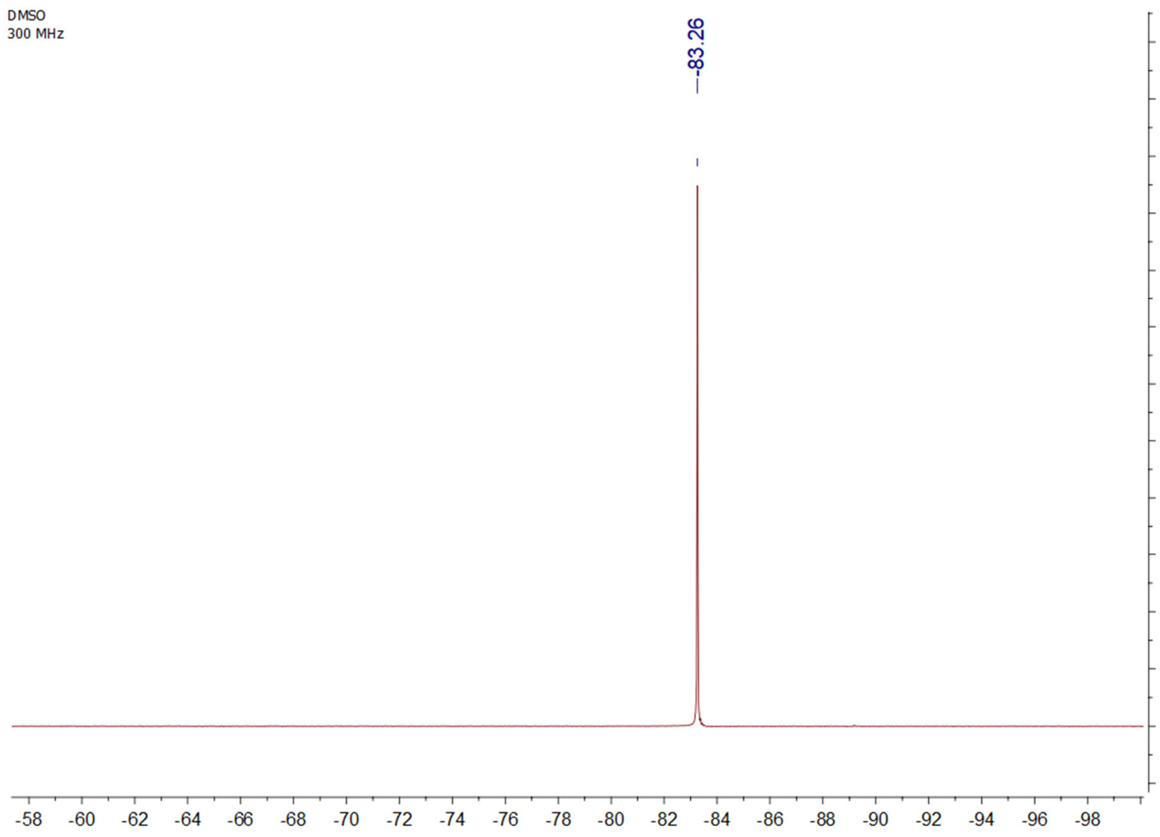

Figure S9. ^{31}P NMR spectrum of (PTA-CH_2-*m*-NO_2-C_6H_4)Br (**2**) in DMSO-d_6 (300 MHz).

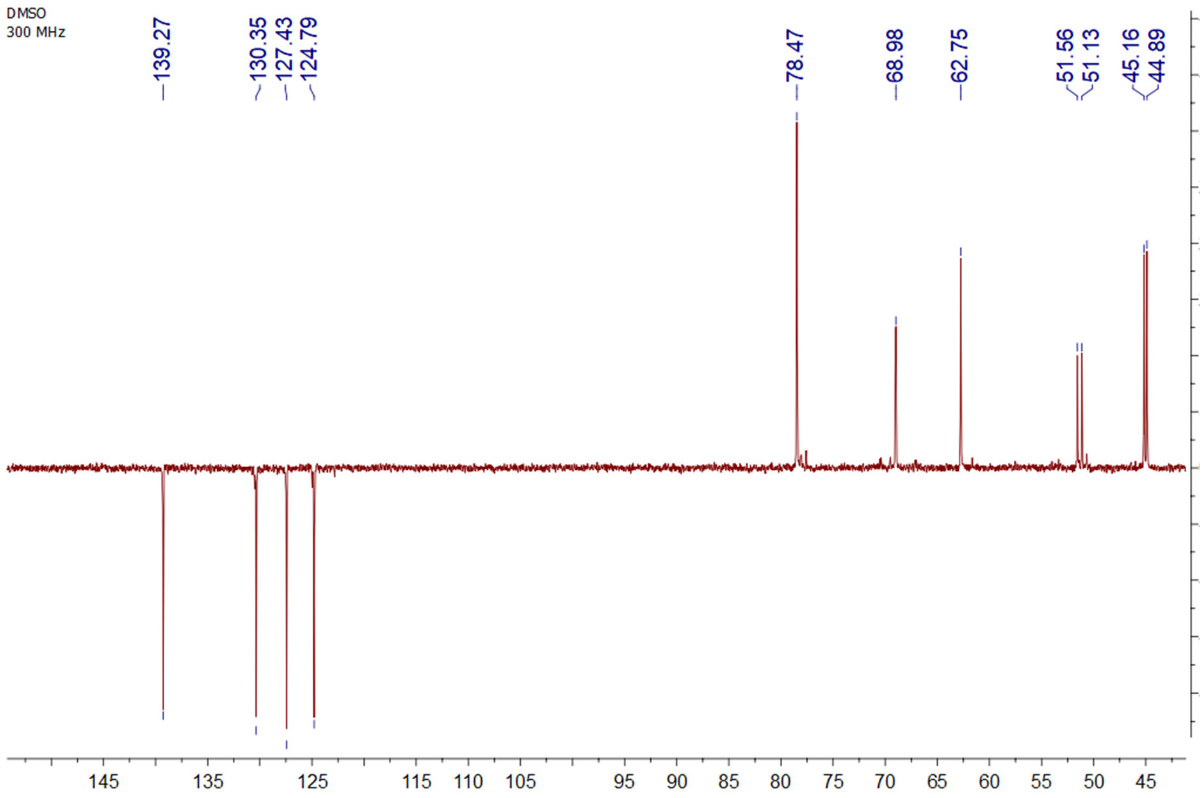

Figure S10. DEPT NMR spectrum of (PTA-CH_2-*m*-NO_2-C_6H_4)Br (**2**) in DMSO-d_6 (300 MHz).

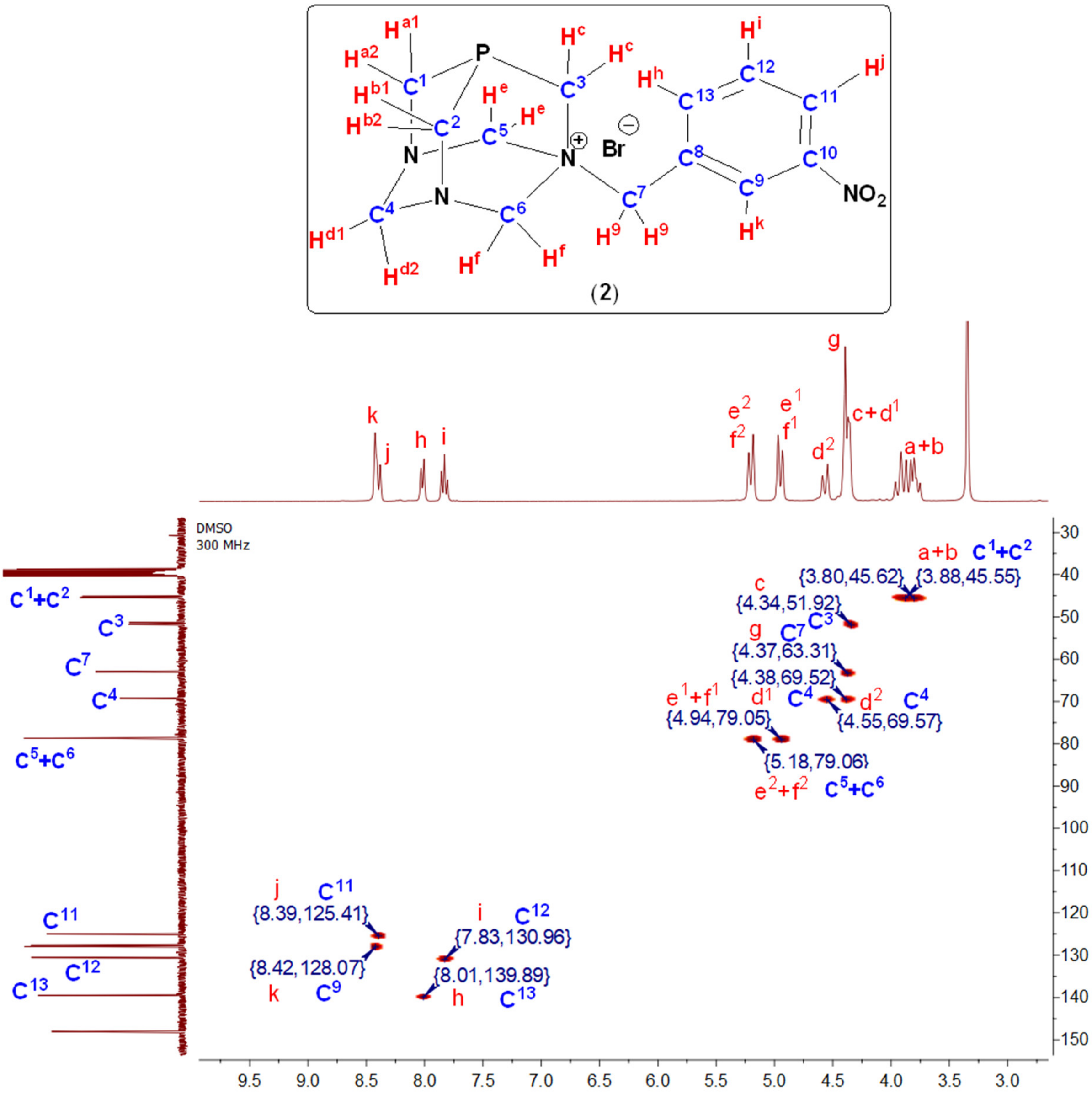

Figure S11. HSQC spectrum of (PTA-CH_2-*m*-NO_2-C_6H_4)Br (**2**) in DMSO-d_6 (300 MHz).

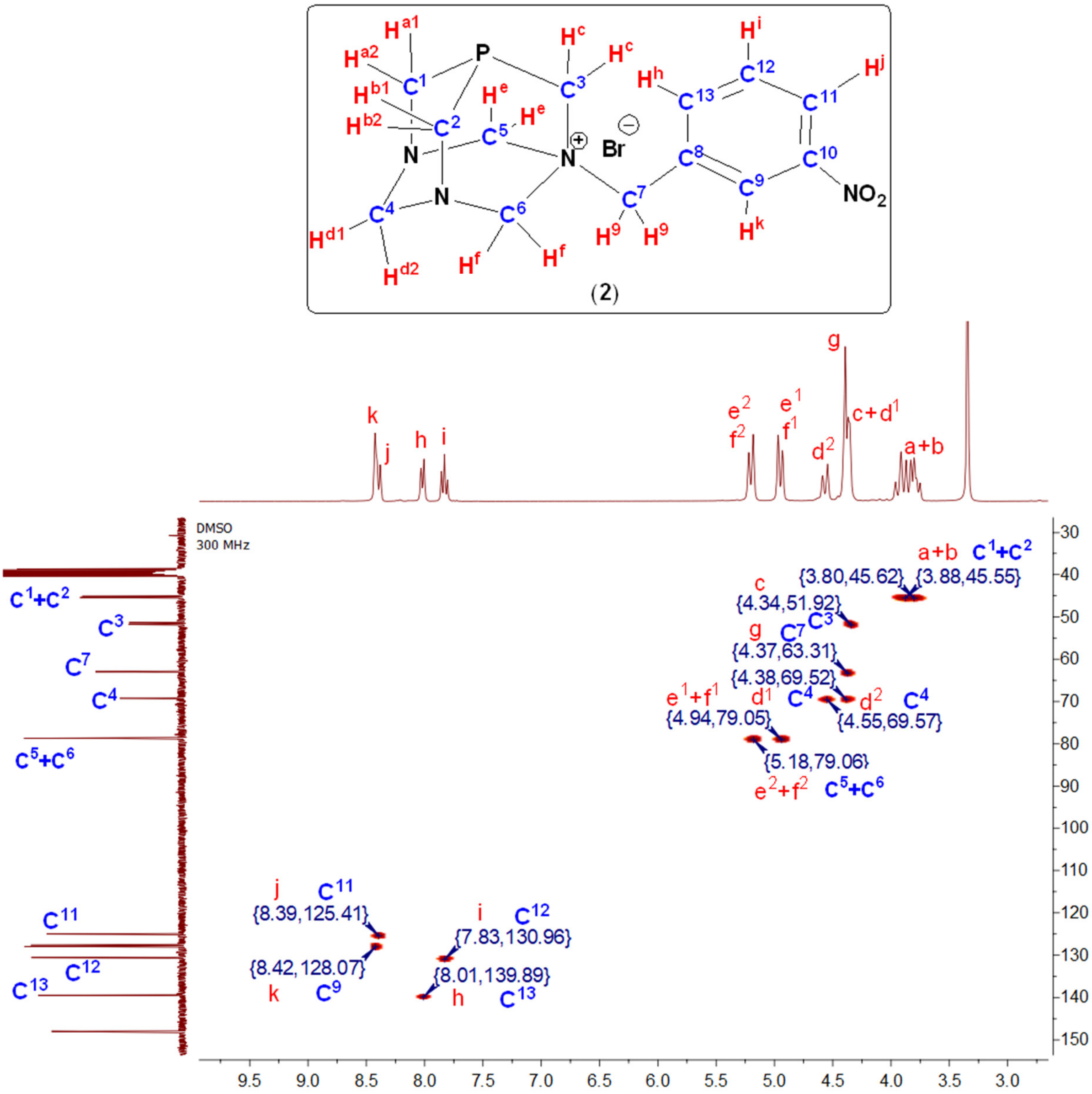

Figure S12. COSY spectrum of (PTA-CH_2-*m*-NO_2-C_6H_4)Br (**2**) in DMSO-d_6 (300 MHz).

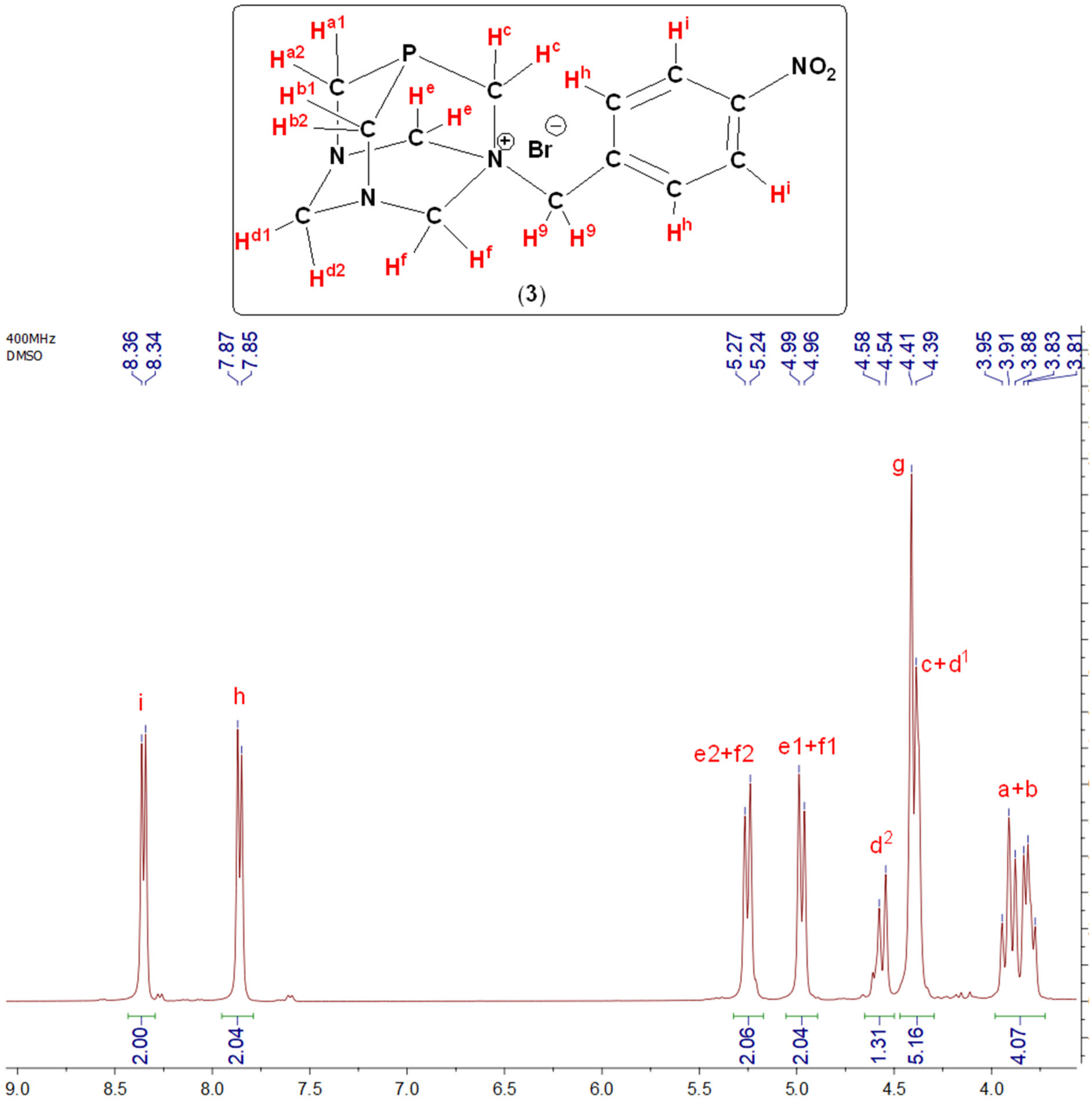

Figure S13. ^{1}H NMR spectrum of (PTA-CH$_2$-*p*-NO$_2$-C$_6$H$_4$)Br (**3**) in DMSO-d_6 (400 MHz).

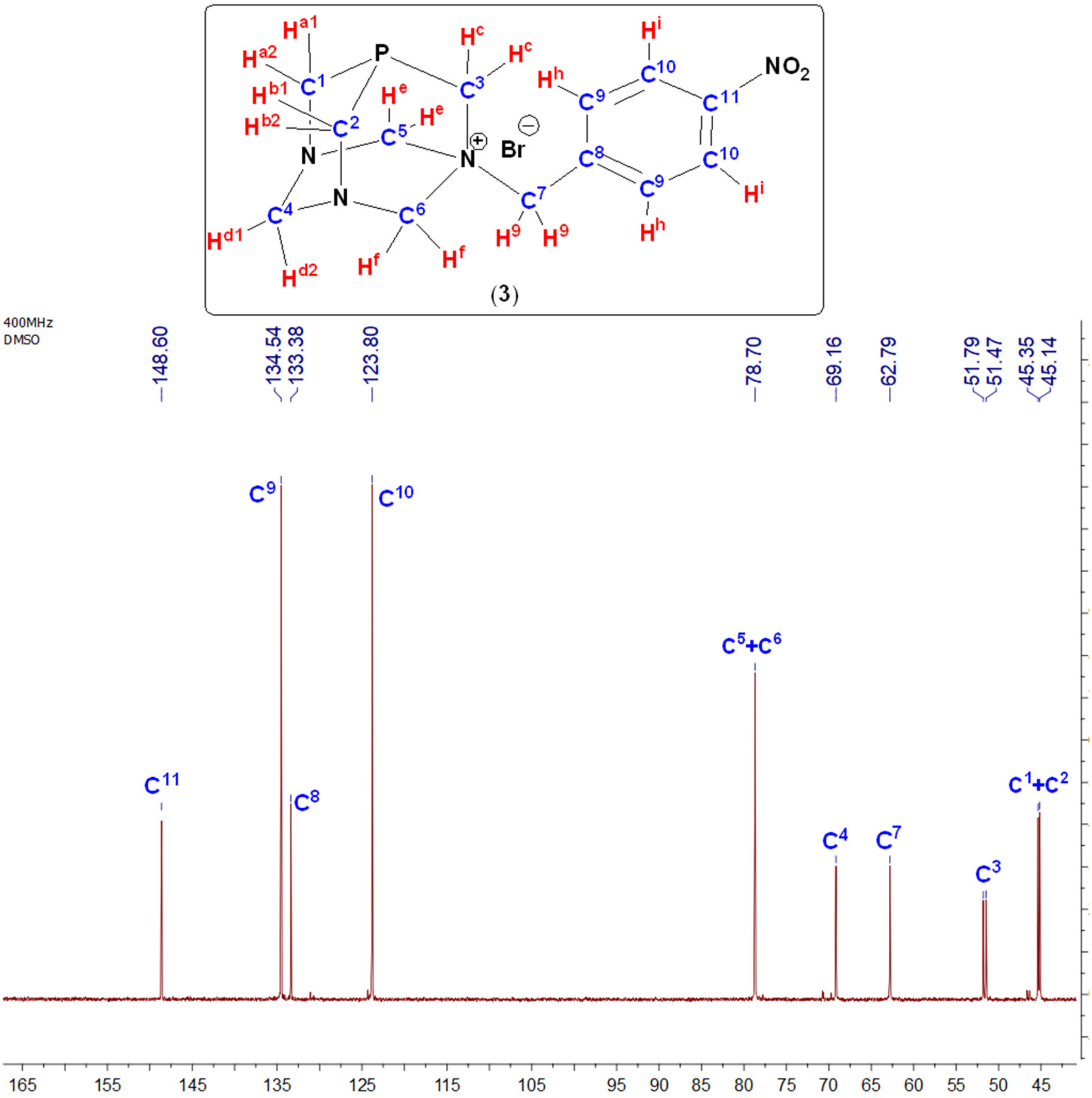

Figure S14. ^{13}C NMR spectrum of (PTA-CH$_2$-*p*-NO$_2$-C$_6$H$_4$)Br (**3**) in DMSO-*d*$_6$ (400 MHz).

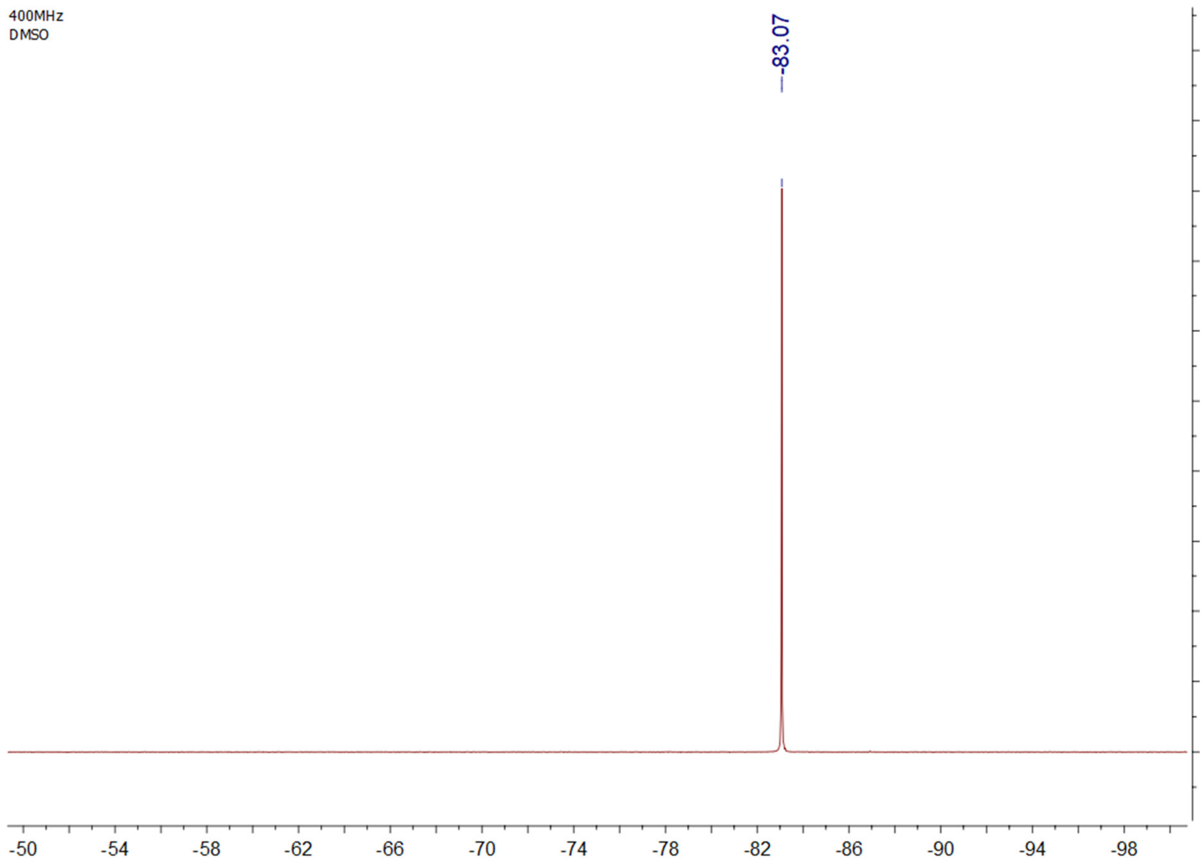

Figure S15. ^{31}P NMR spectrum of (PTA-CH_2-*p*-NO_2-C_6H_4)Br (**3**) in DMSO-d_6 (400 MHz).

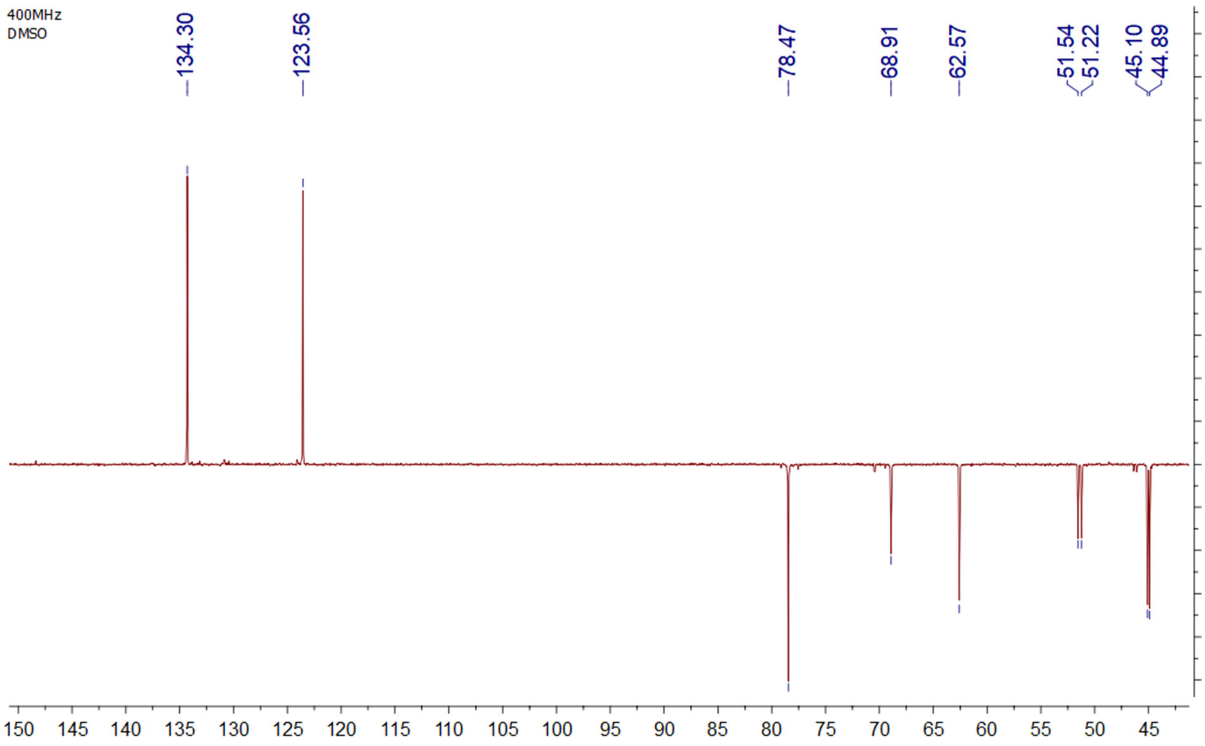

Figure S16. DEPT NMR spectrum of (PTA-CH_2-*p*-NO_2-C_6H_4)Br (**3**) in DMSO-d_6 (400 MHz).

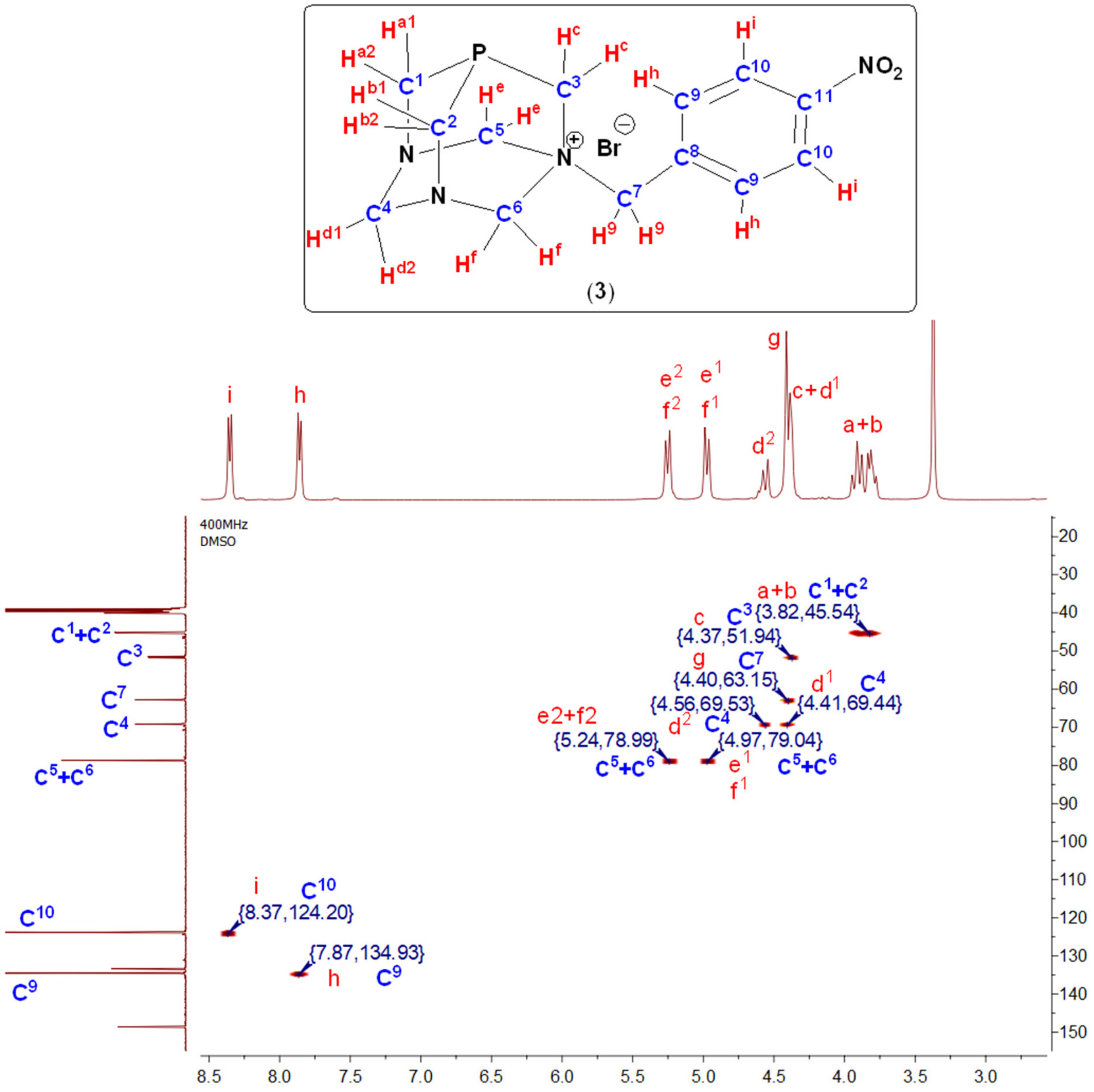

Figure S17. HSQC spectrum of (PTA-CH$_2$-*p*-NO$_2$-C$_6$H$_4$)Br (**3**) in DMSO-d_6 (400 MHz).

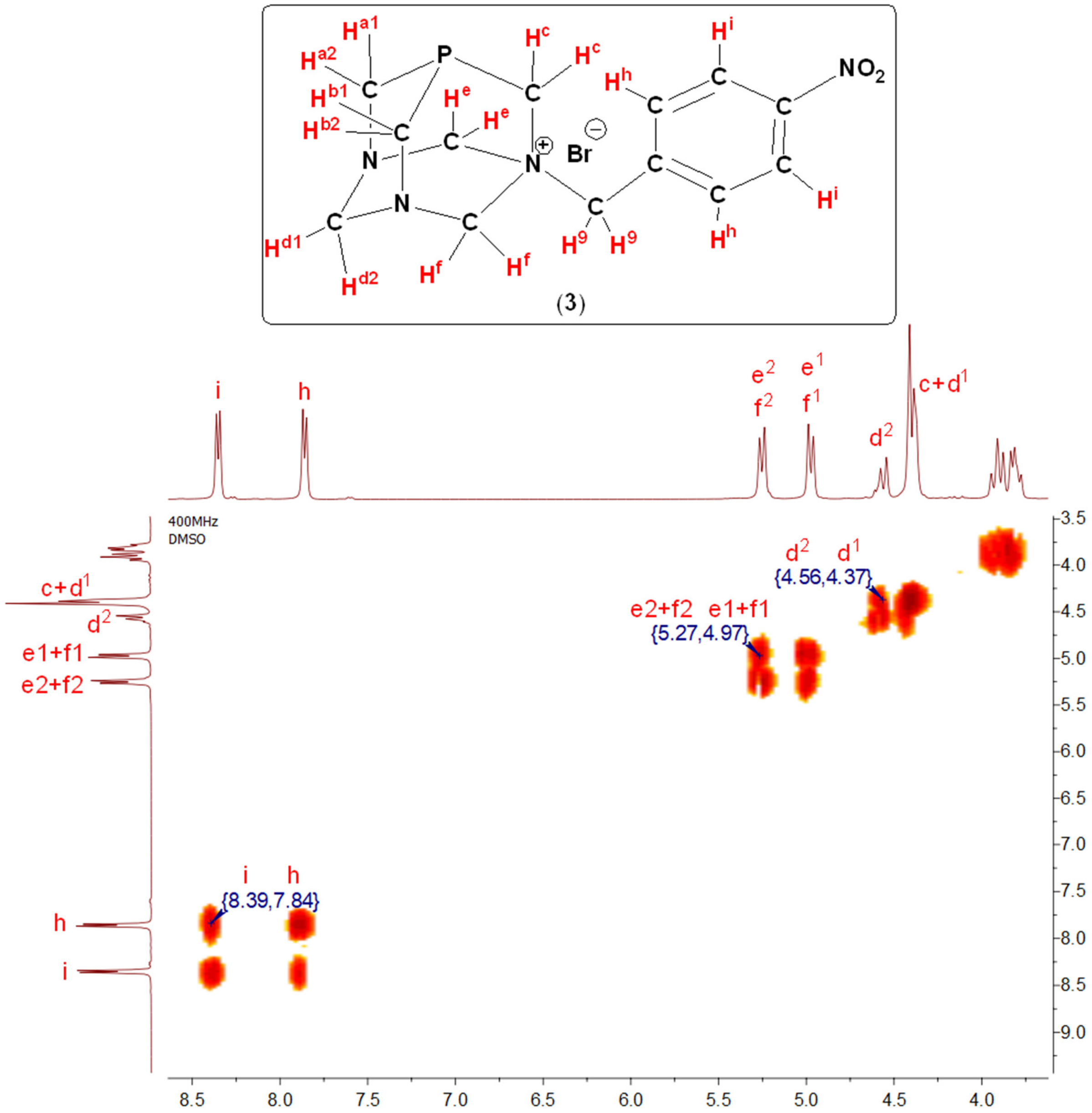

Figure S18. COSY spectrum of (PTA-CH_2-*p*-NO_2-C_6H_4)Br (**3**) in DMSO-d_6 (400 MHz).

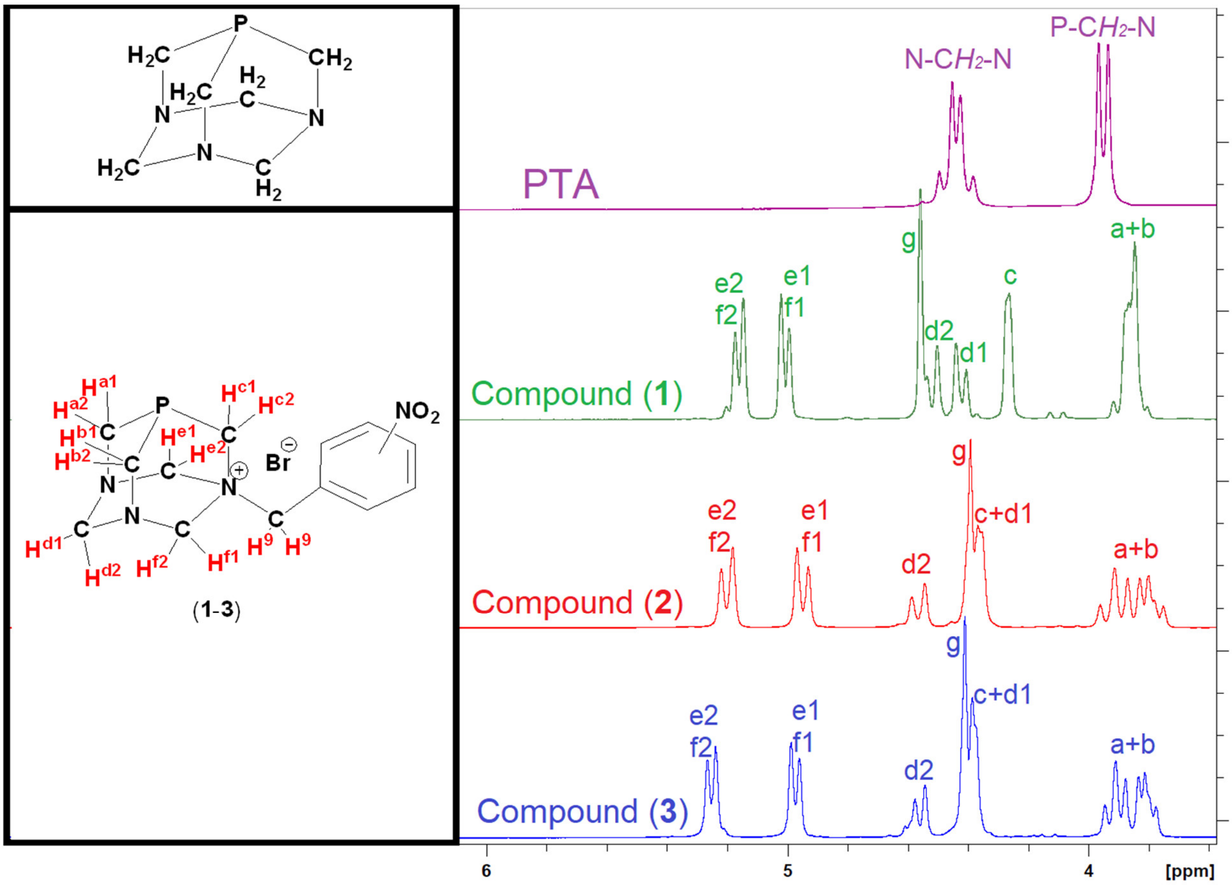

Figure S19. 1H NMR spectra of compounds 1-3 and their PTA precursor in DMSO-d6.

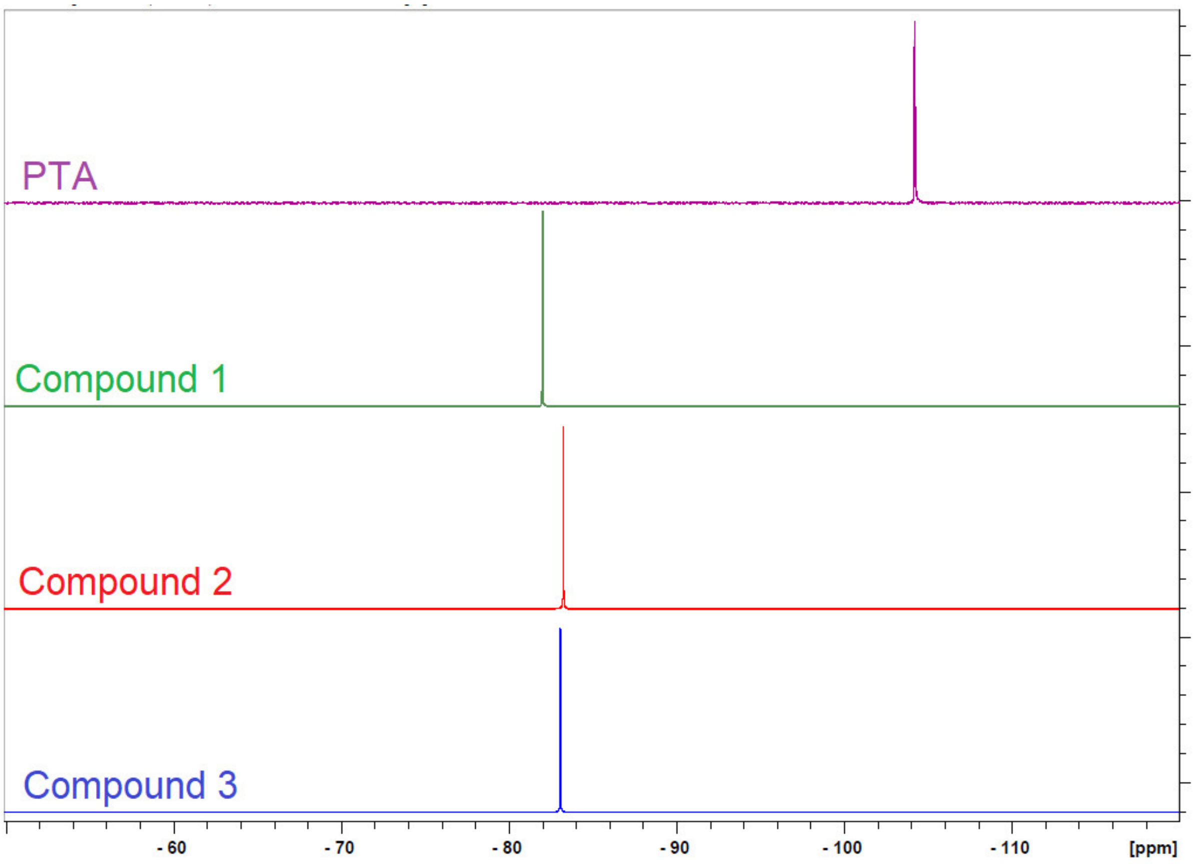

Figure S20. $^{31}P\{^{1}H\}$ NMR spectra of PTA and compounds (**1**-**3**) in DMSO-*d*6.

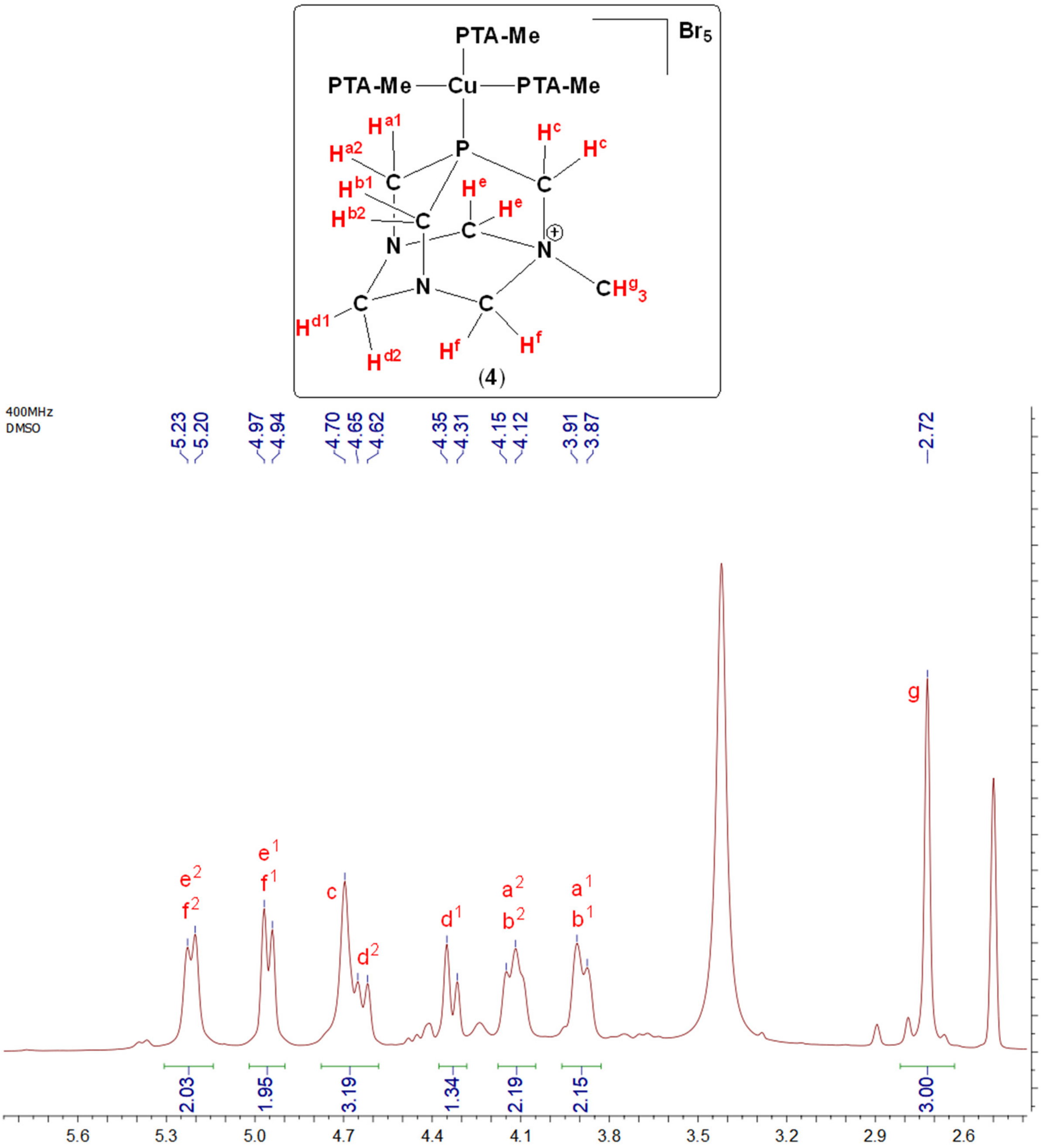

Figure S21. ^{1}H NMR spectrum of [Cu(PTA-Me]·Br_5 (**4**) in DMSO-d_6 (400 MHz).

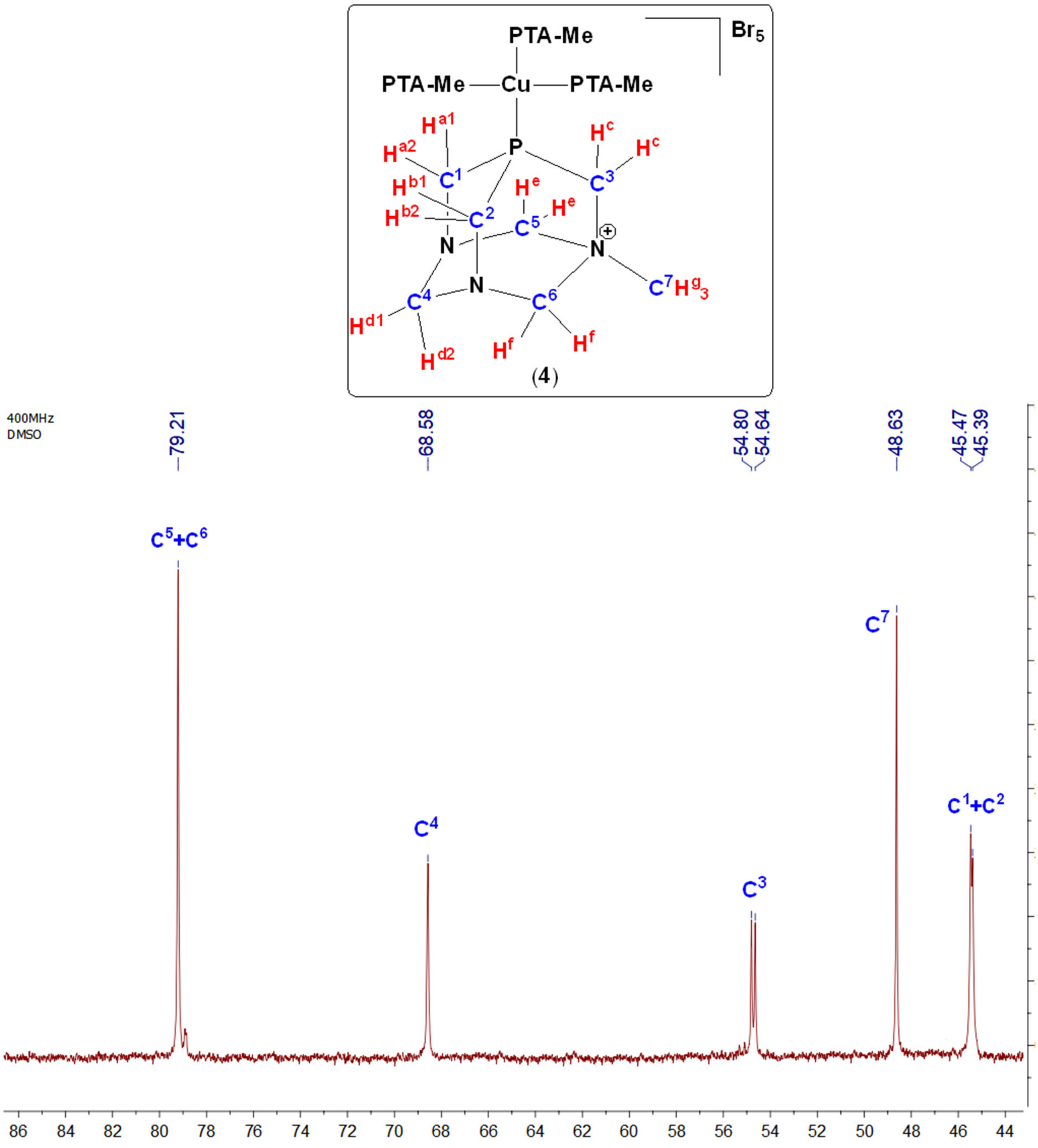

Figure S22. ^{13}C NMR spectrum of [Cu(PTA-Me]·Br_5 (**4**) in DMSO-d_6 (400 MHz).

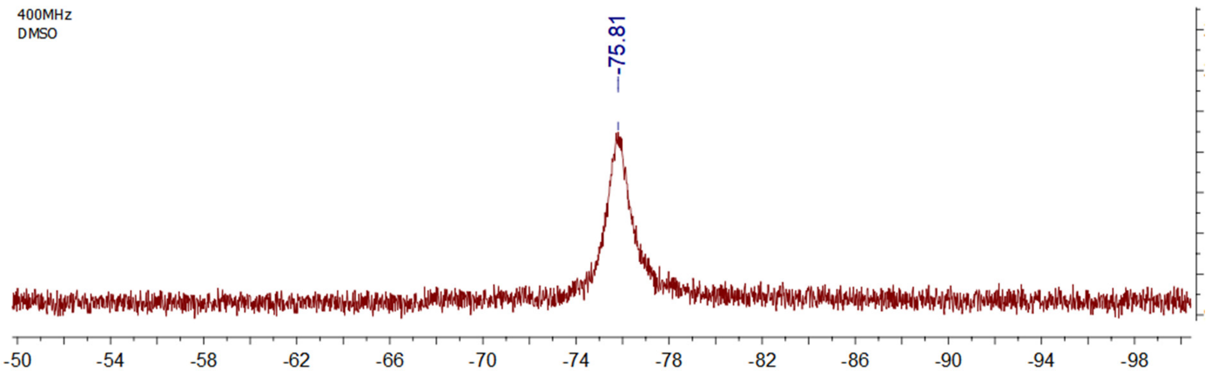

Figure S23. ^{31}P NMR spectrum of [Cu(PTA-Me]·Br_5 (**4**) in DMSO-d_6 (400 MHz).

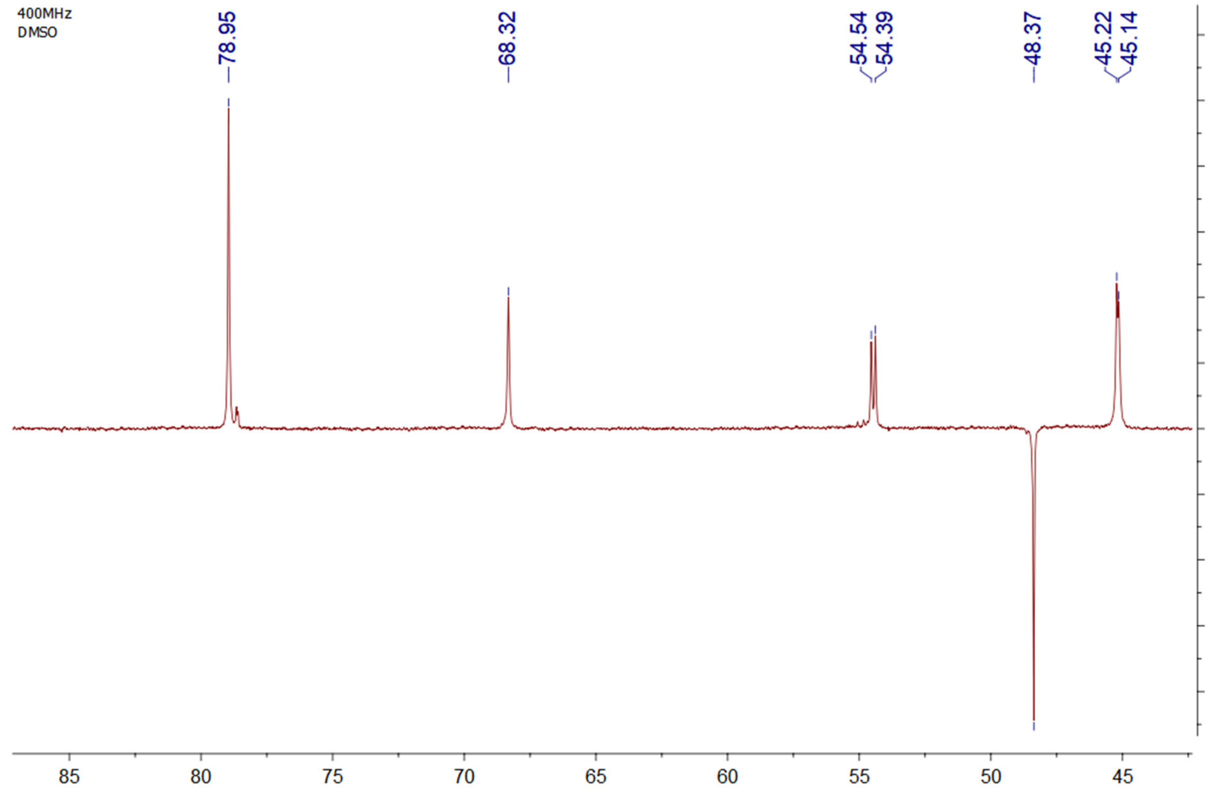

Figure S24. DEPT NMR spectrum of [Cu(PTA-Me]·Br_5 (**4**) in DMSO-d_6 (400 MHz).

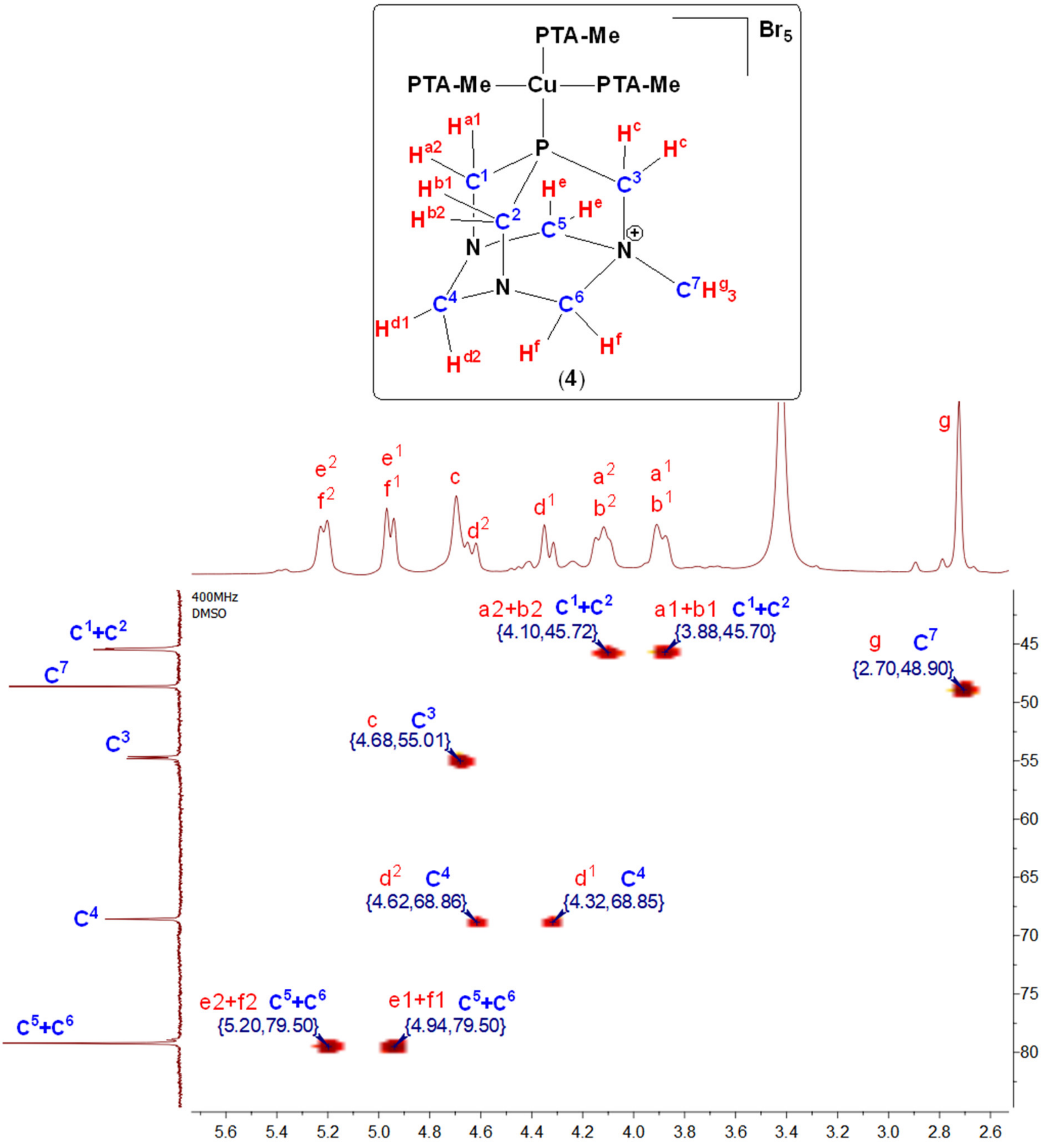

Figure S25. HSQC spectrum of [Cu(PTA-Me]·Br_5 (**4**) in DMSO-d_6 (400 MHz).

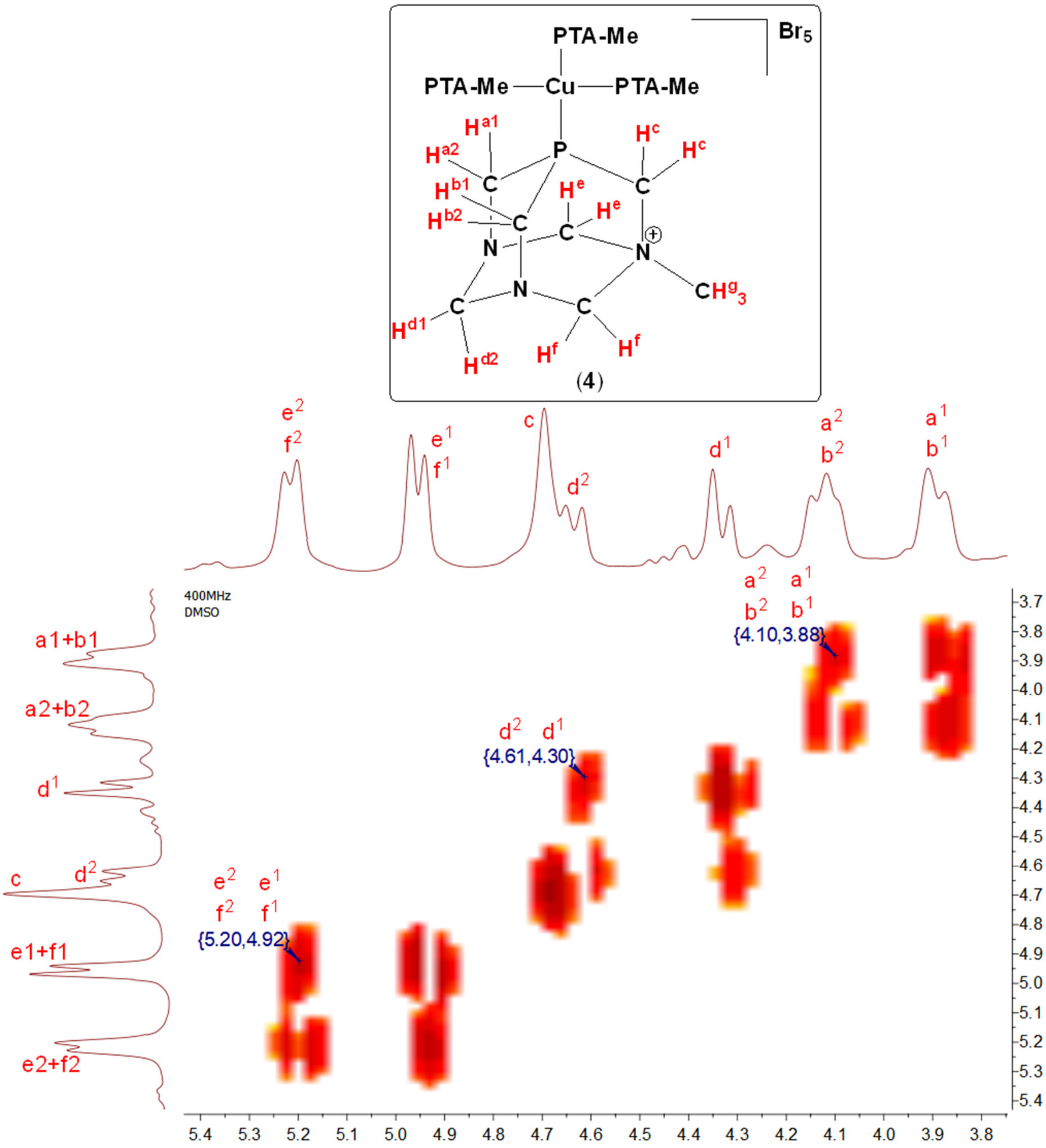

Figure S26. COSY spectrum of [Cu(PTA-Me]·Br_5 (**4**) in DMSO-d_6 (400 MHz).

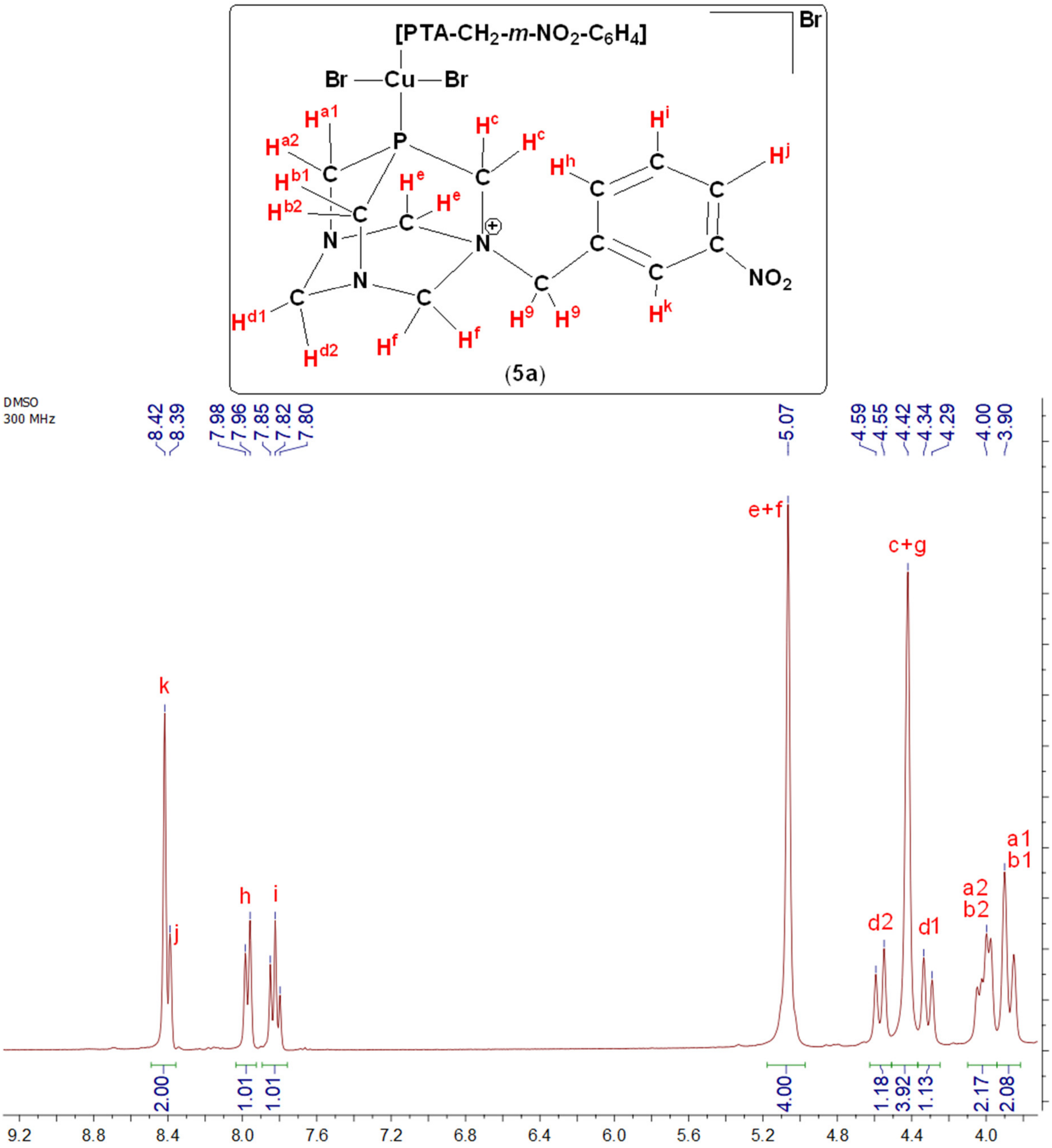

Figure S27. ^{1}H NMR spectrum of $[CuBr_2(PTA\text{-}CH_2\text{-}m\text{-}NO_2\text{-}C_6H_4)_2]\cdot Br$ (**5a**) in DMSO-d_6 (300 MHz).

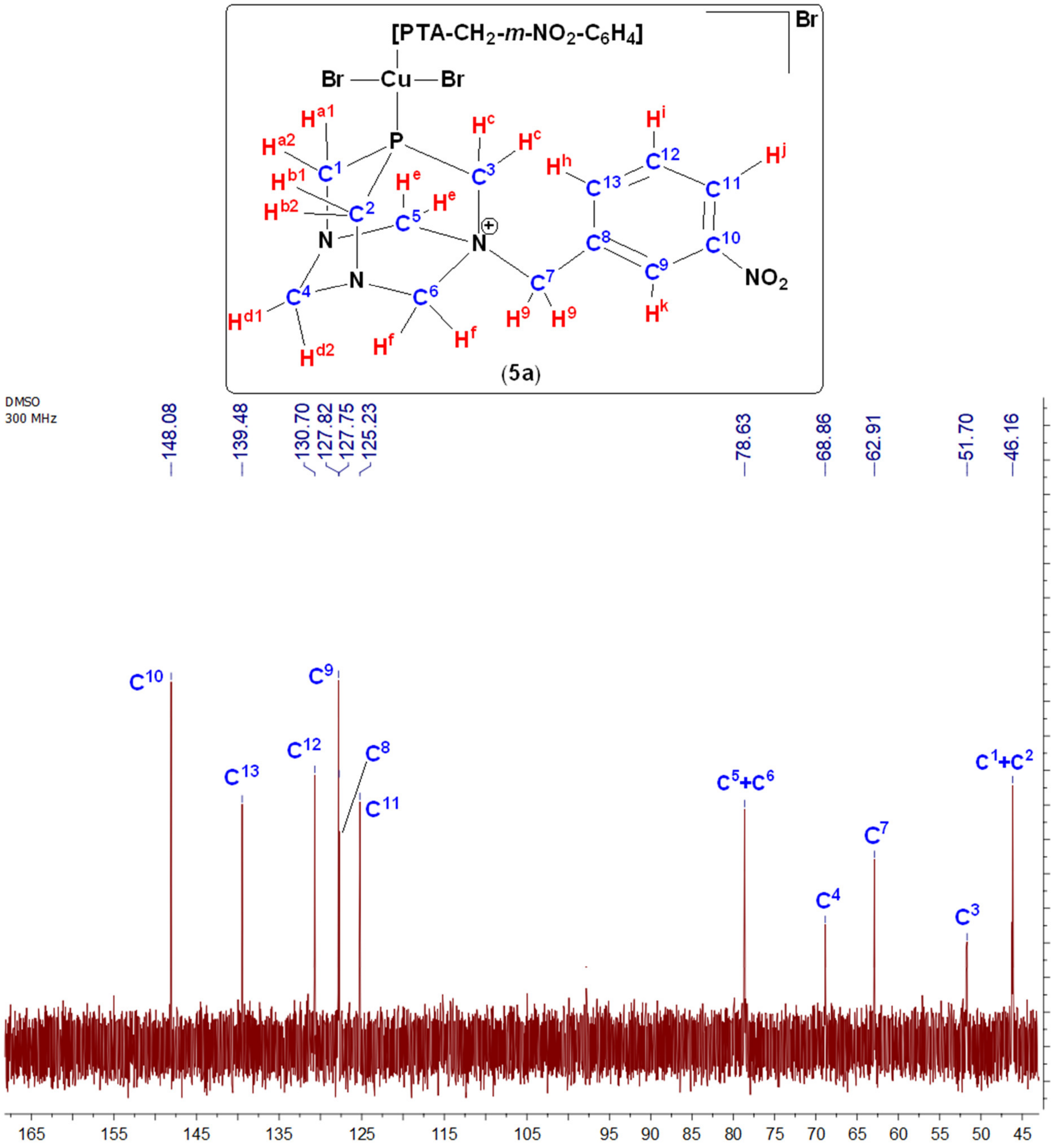

Figure S28. ^{13}C NMR spectrum of $[CuBr_2(PTA\text{-}CH_2\text{-}m\text{-}NO_2\text{-}C_6H_4)_2]\cdot Br$ (**5a**) in DMSO-d_6 (300 MHz).

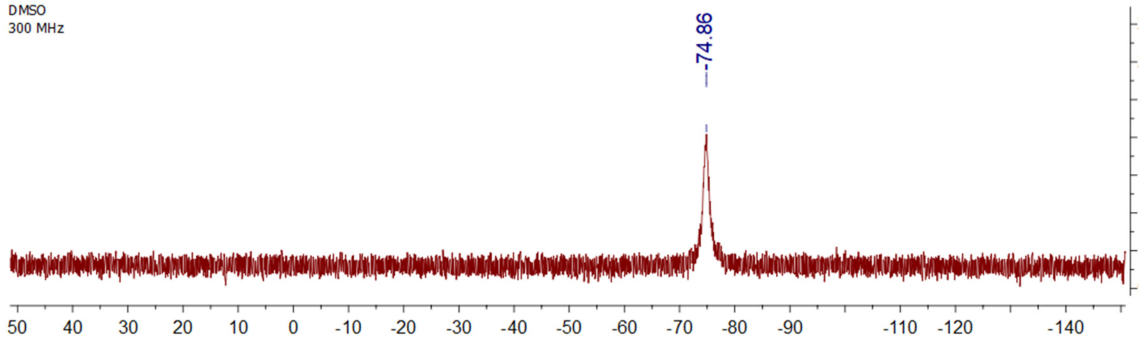

Figure S29. ^{31}P NMR spectrum of [$CuBr_2$(PTA-CH_2-*m*-NO_2-C_6H_4)$_2$]·Br (**5a**) in DMSO-d_6 (300 MHz).

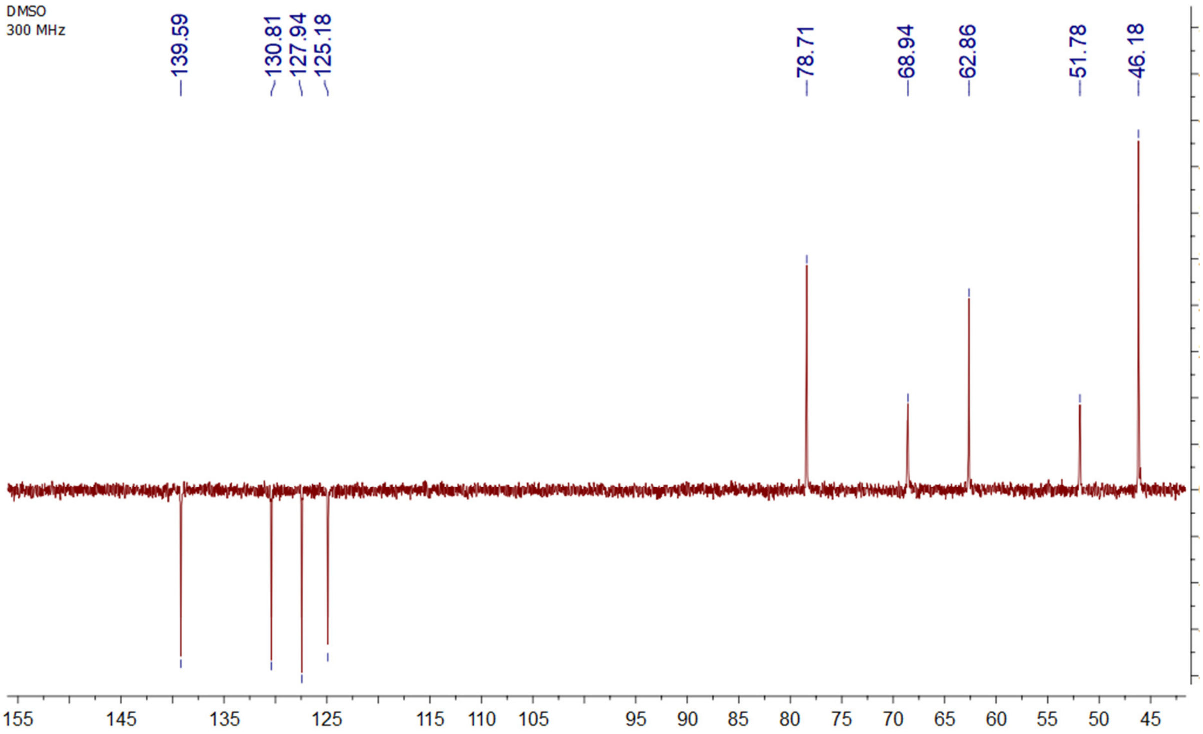

Figure S30. DEPT NMR spectrum of [$CuBr_2$(PTA-CH_2-*m*-NO_2-C_6H_4)$_2$]·Br (**5a**) in DMSO-d_6 (300 MHz).

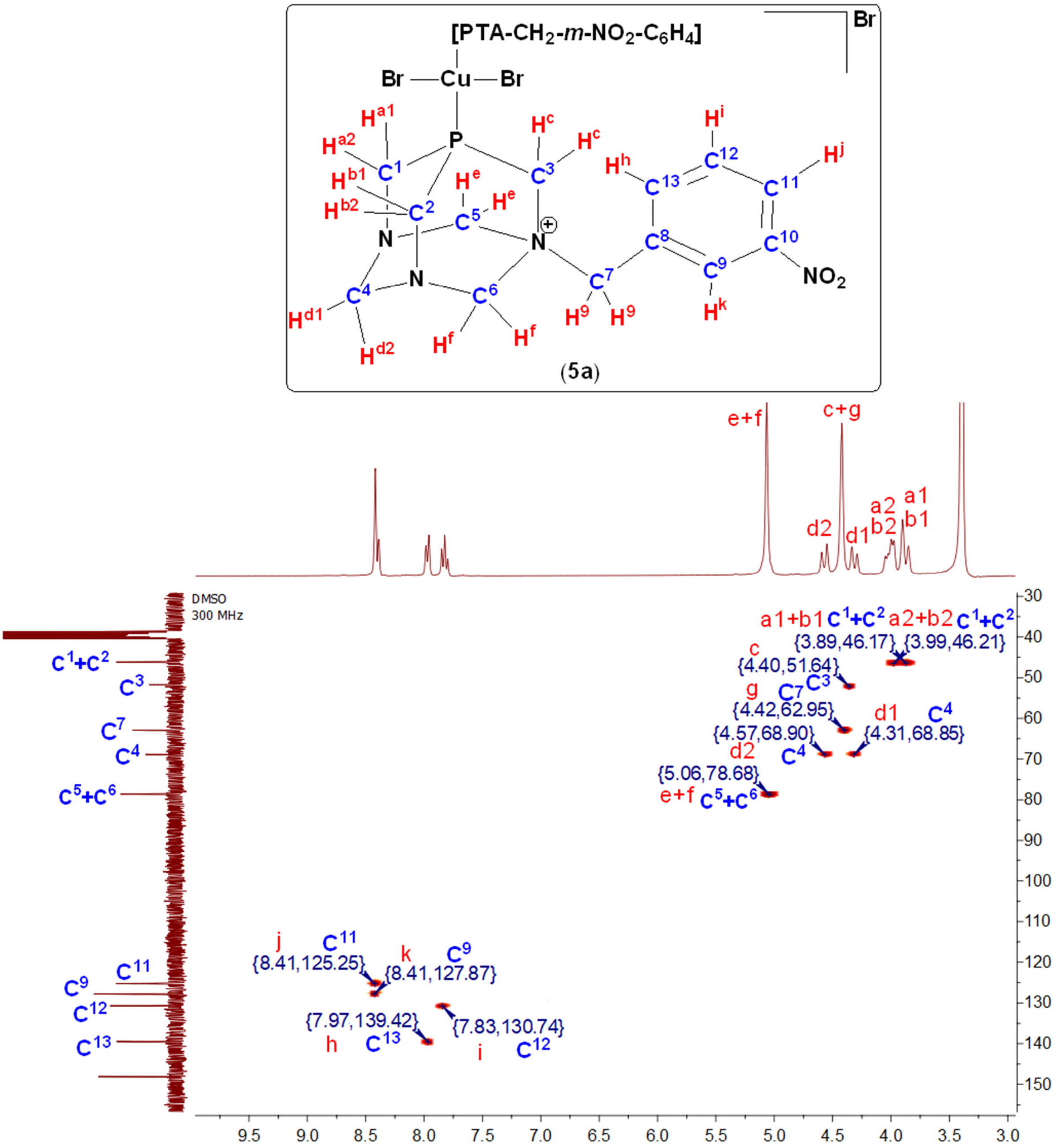

Figure S31. HSQC spectrum of $[CuBr_2(PTA\text{-}CH_2\text{-}m\text{-}NO_2\text{-}C_6H_4)_2]\cdot Br$ (**5a**) in DMSO-d_6 (300 MHz).

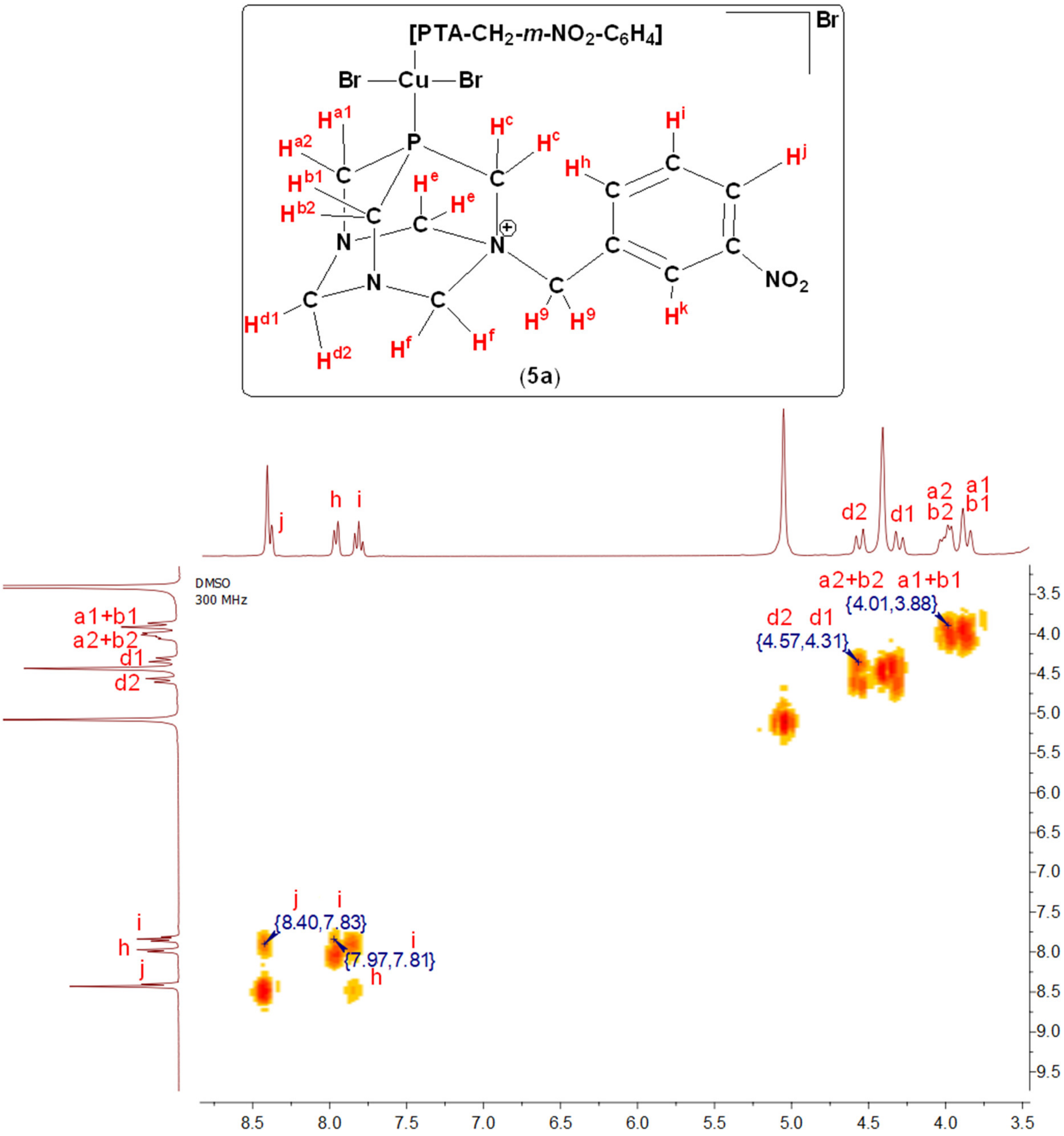

Figure S32. COSY spectrum of $[CuBr_2(PTA\text{-}CH_2\text{-}m\text{-}NO_2\text{-}C_6H_4)_2]\cdot Br$ (**5a**) in DMSO-d_6 (300 MHz).

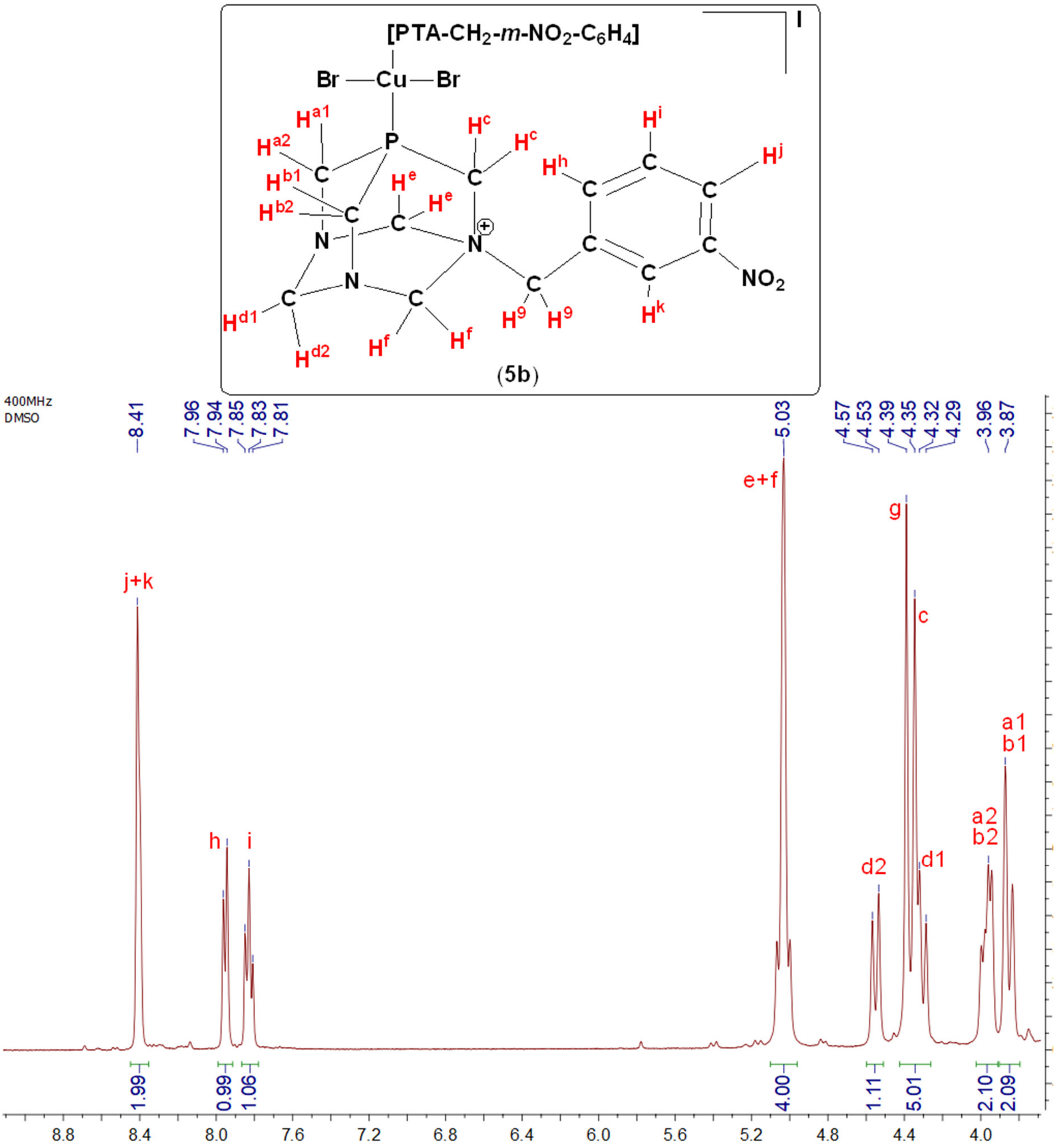

Figure S33. ^{1}H NMR spectrum of $[CuBr_2(PTA\text{-}CH_2\text{-}m\text{-}NO_2\text{-}C_6H_4)_2]\cdot I$ (**5b**) in DMSO-d_6 (400 MHz).

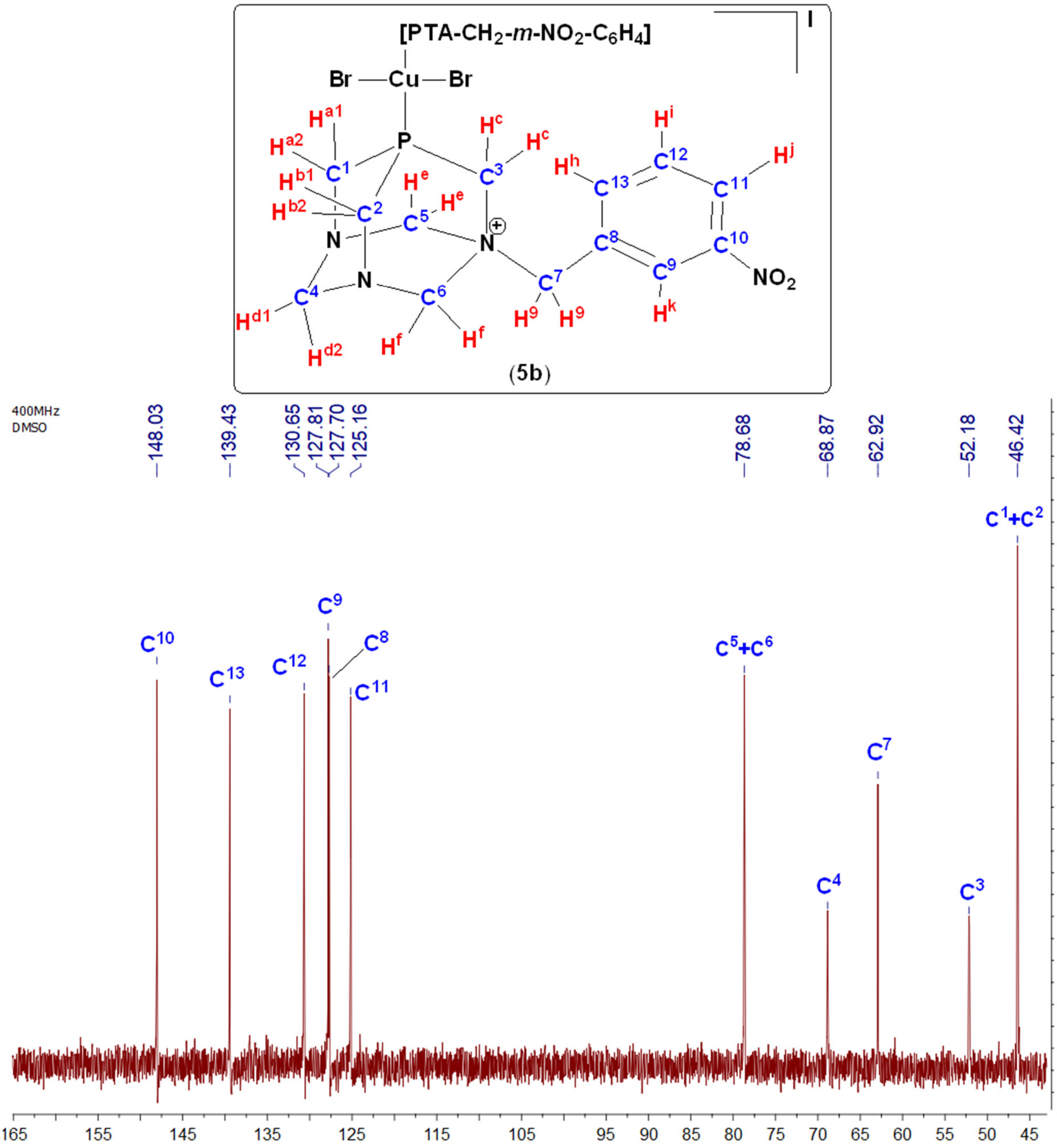

Figure S34. ^{13}C NMR spectrum of [$CuBr_2$(PTA-CH_2-*m*-NO_2-C_6H_4)$_2$]·I (**5b**) in DMSO-d_6 (400 MHz).

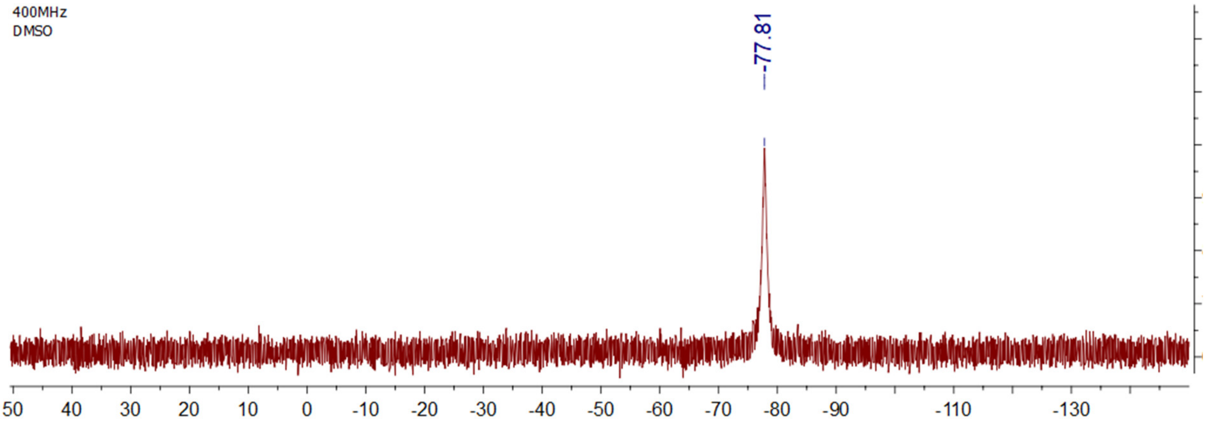

Figure S35. ^{31}P NMR spectrum of $[CuBr_2(PTA\text{-}CH_2\text{-}m\text{-}NO_2\text{-}C_6H_4)_2]\cdot I$ (**5b**) in DMSO-d_6 (400 MHz).

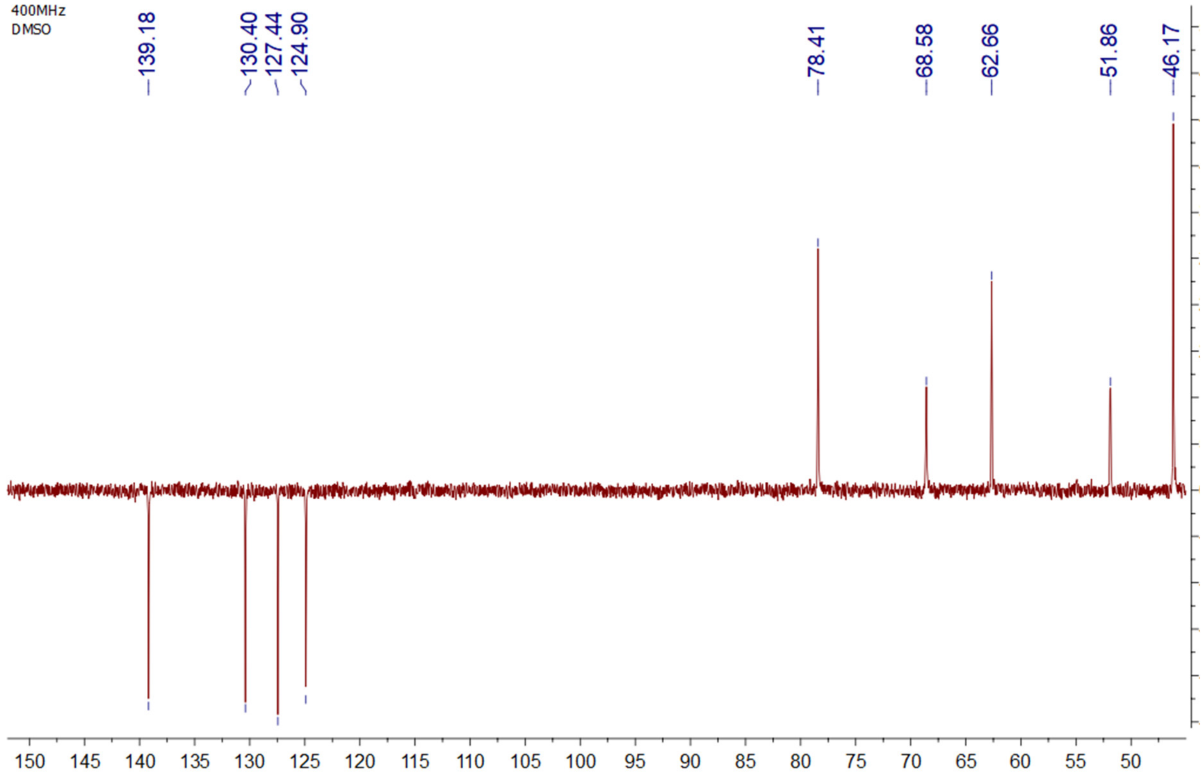

Figure S36. DEPT NMR spectrum of $[CuBr_2(PTA\text{-}CH_2\text{-}m\text{-}NO_2\text{-}C_6H_4)_2]\cdot I$ (**5b**) in DMSO-d_6 (400 MHz).

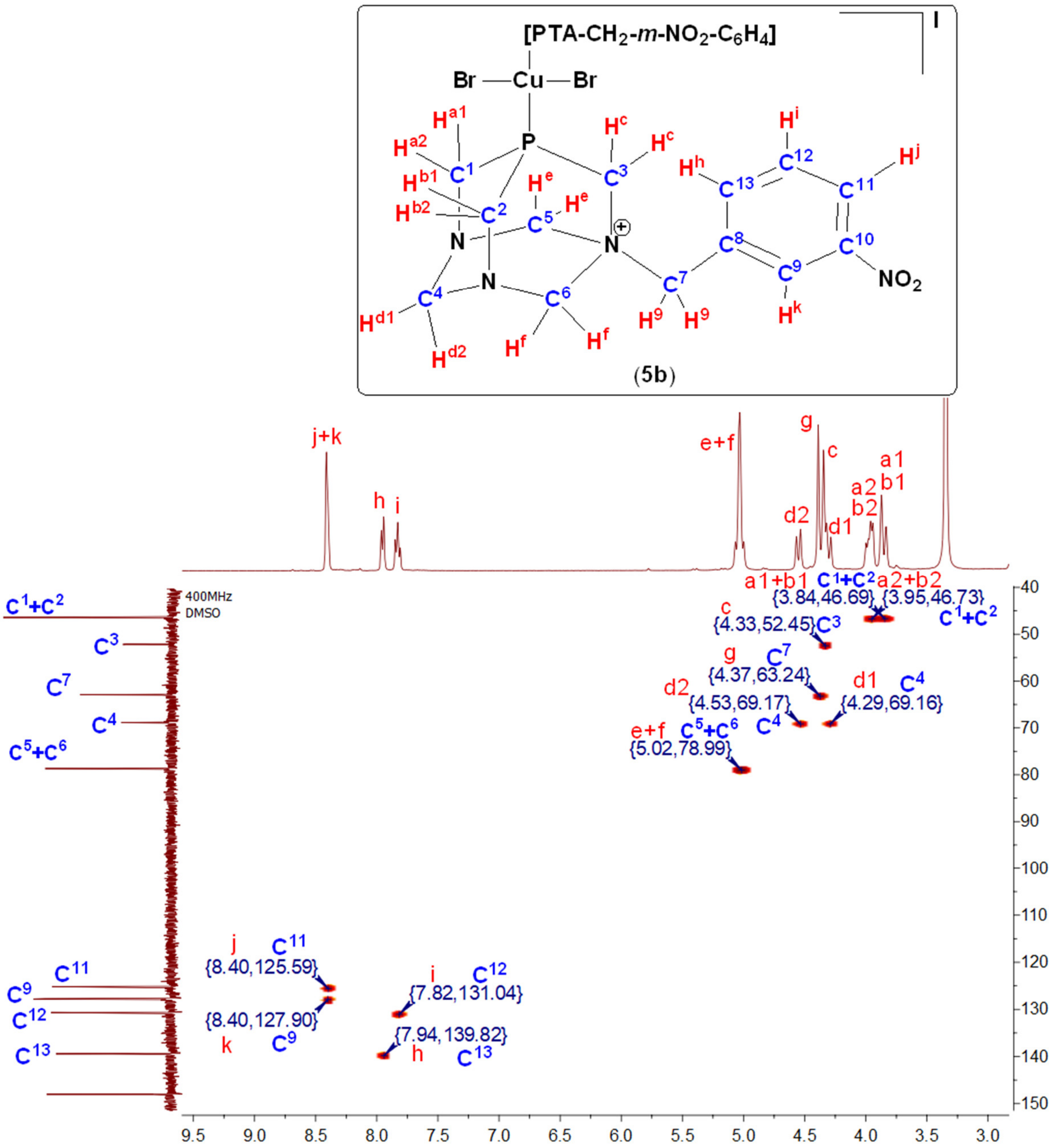

Figure S37. HSQC spectrum of [CuBr2(PTA-CH2-*m*-NO2-C6H4)2]·I (**5b**) in DMSO-*d*6 (400 MHz).

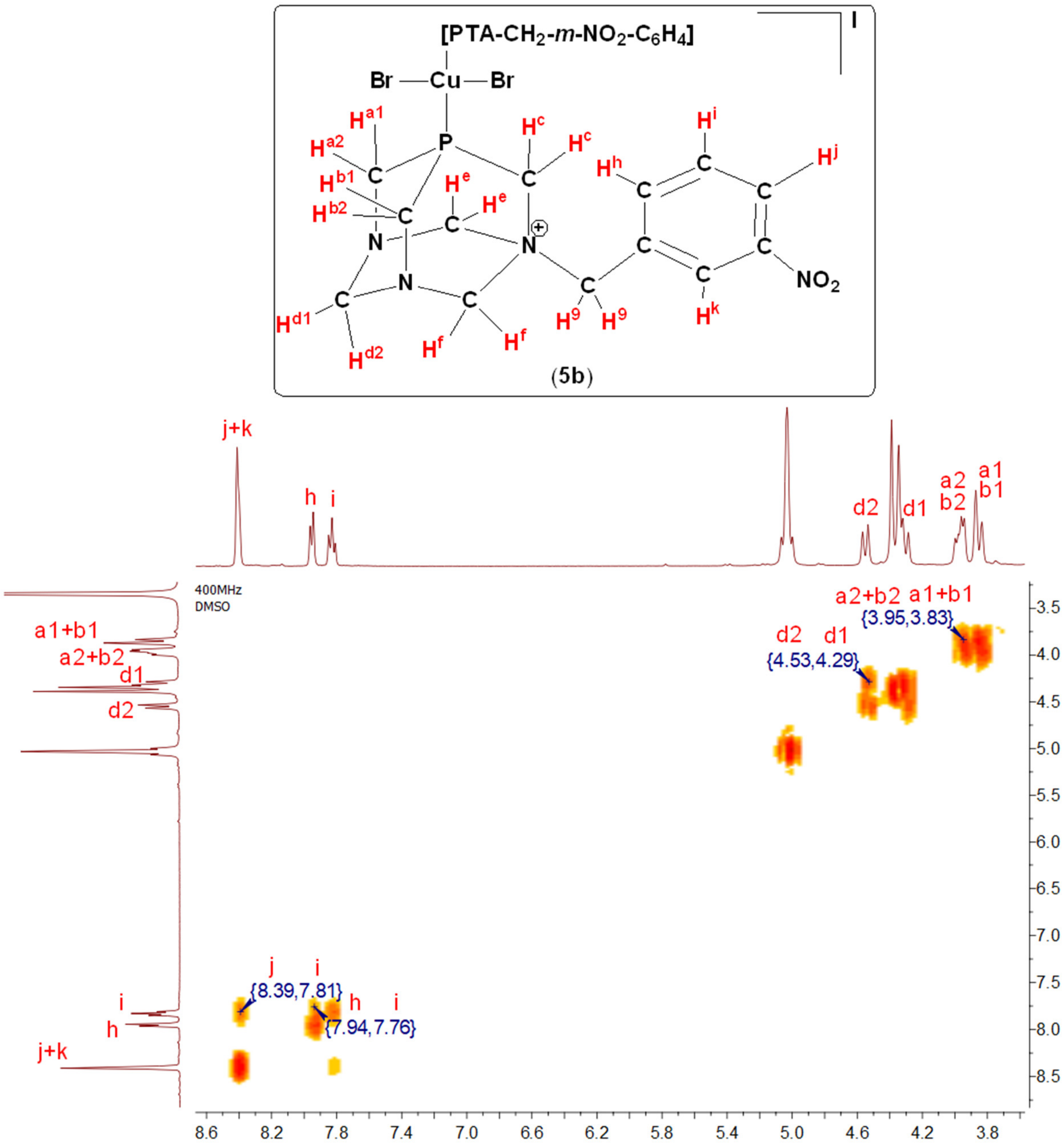

Figure S38. COSY spectrum of [CuBr$_2$(PTA-CH$_2$-*m*-NO$_2$-C$_6$H$_4$)$_2$]·I (**5b**) in DMSO-*d*$_6$ (400 MHz).

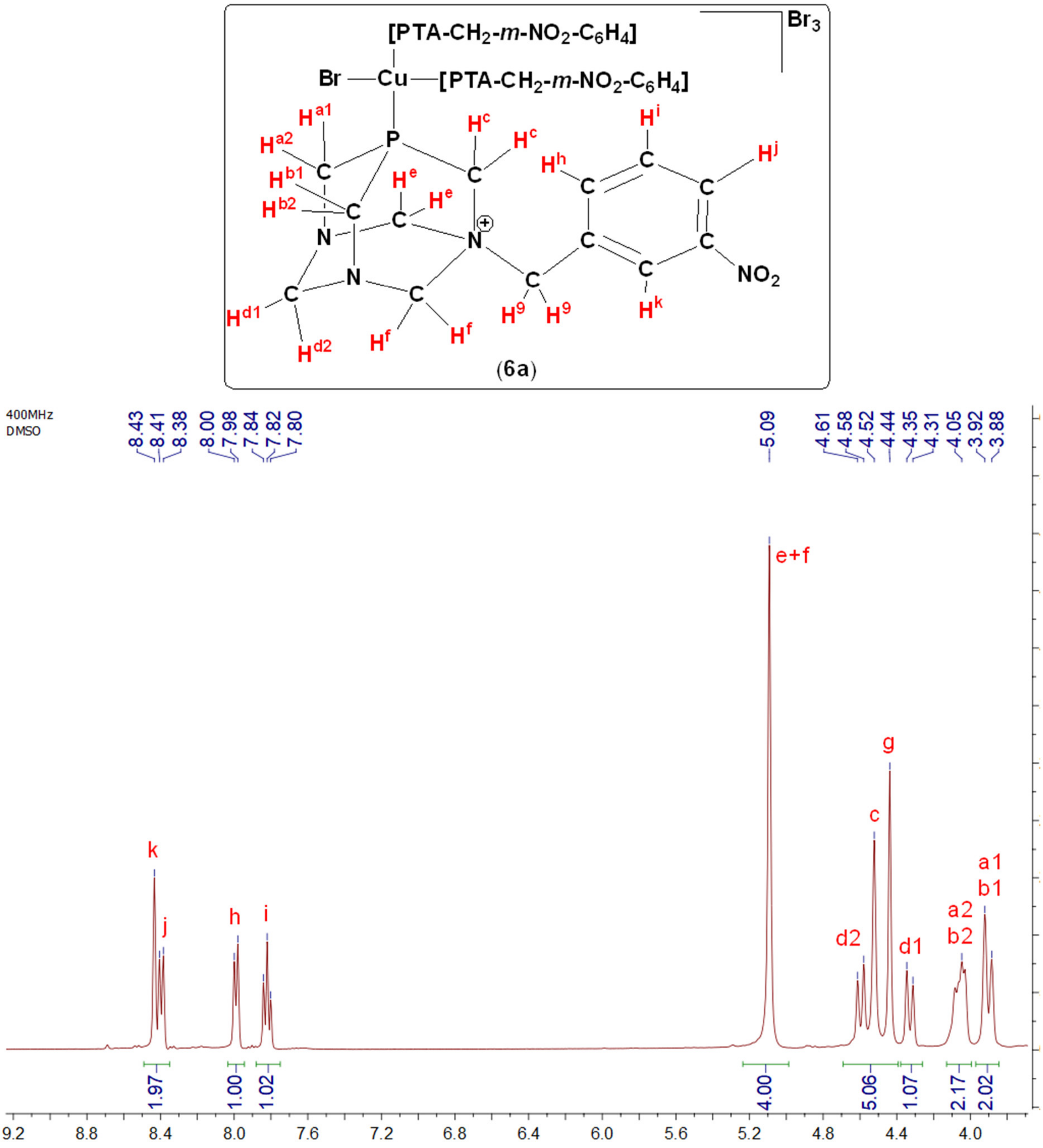

Figure S39. ^{1}H NMR spectrum of [CuBr(PTA-CH2-*m*-NO2-C6H4)3]·Br3 (**6a**) in DMSO-*d*6 (400 MHz).

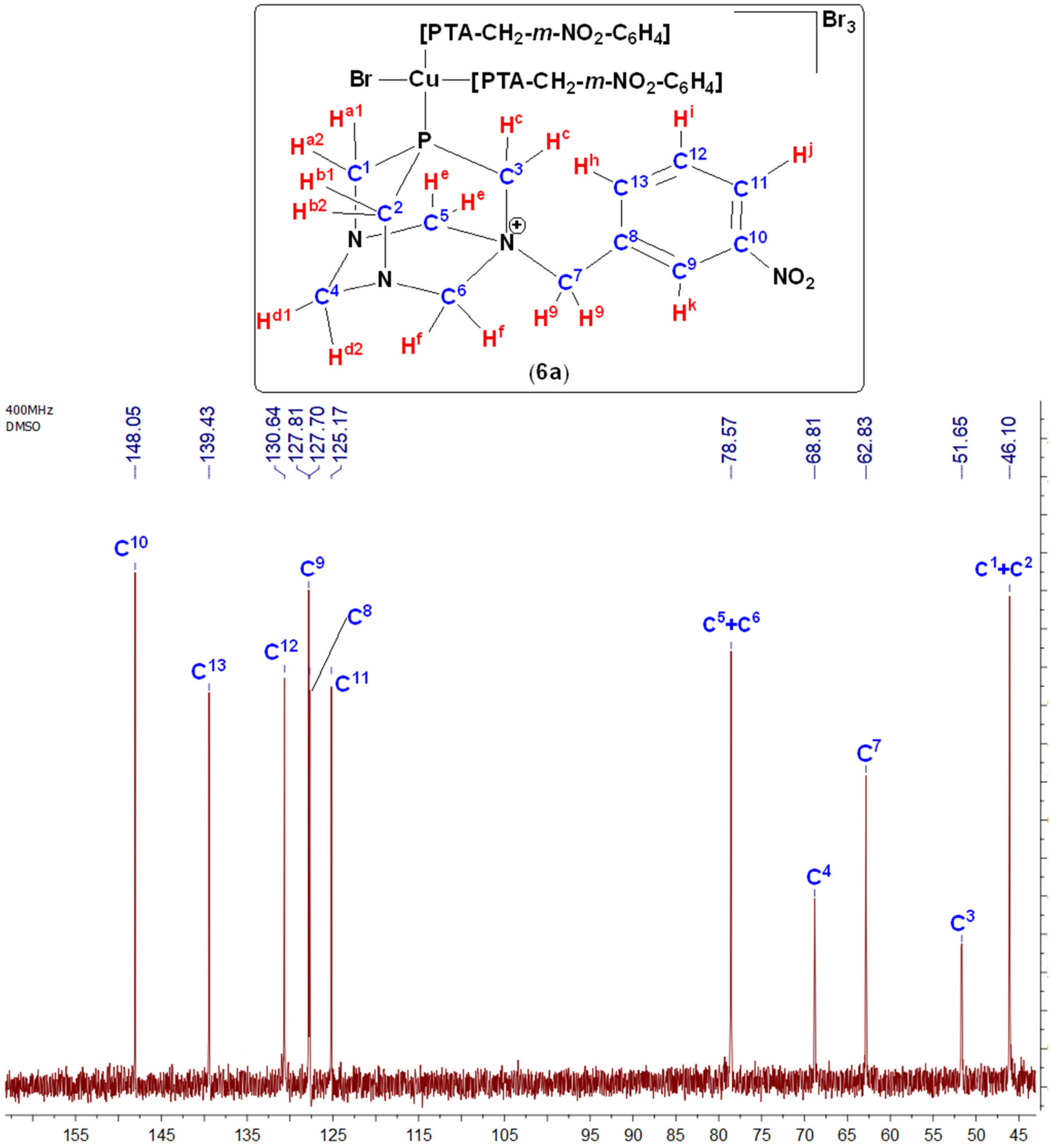

Figure S40. ^{13}C NMR spectrum of [CuBr(PTA-CH$_2$-*m*-NO$_2$-C$_6$H$_4$)$_3$]·Br$_3$ (**6a**) in DMSO-d_6 (400 MHz).

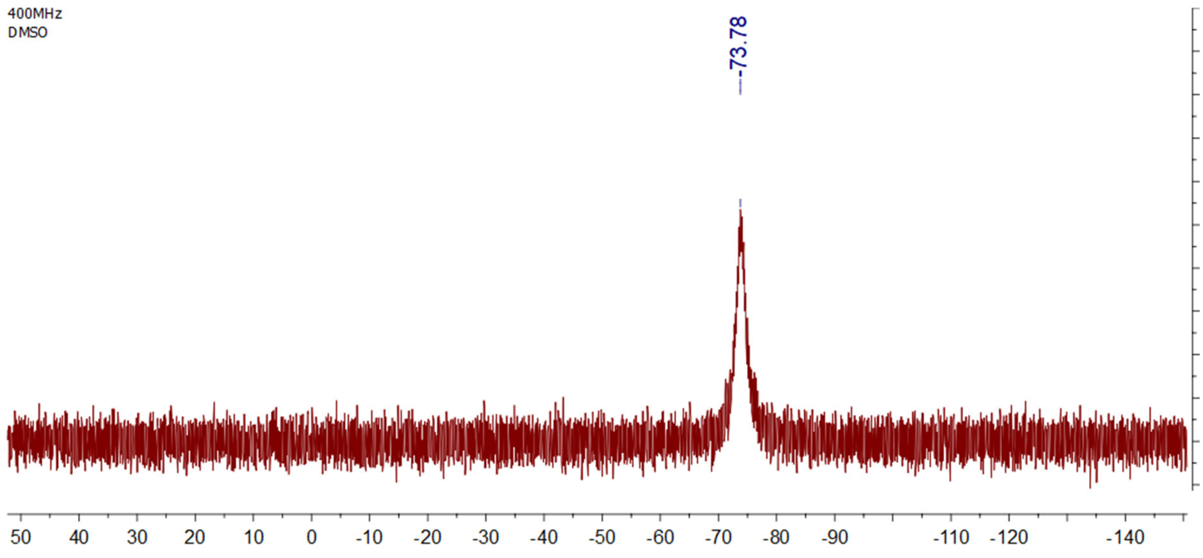

Figure S41. ^{31}P NMR spectrum of [CuBr(PTA-CH_2-*m*-NO_2-C_6H_4)$_3$]·Br_3 (**6a**) in DMSO-d_6 (400 MHz).

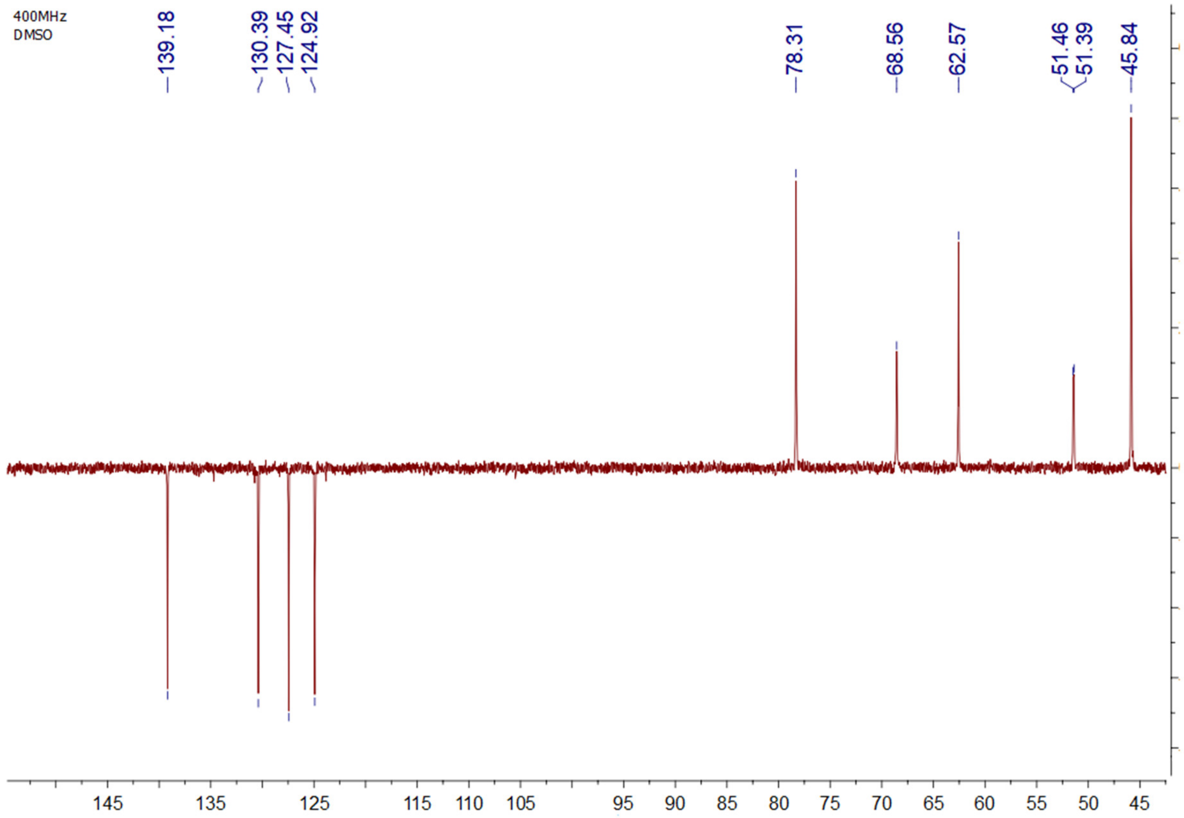

Figure S42. DEPT NMR spectrum of [CuBr(PTA-CH_2-*m*-NO_2-C_6H_4)$_3$]·Br_3 (**6a**) in DMSO-d_6 (400 MHz).

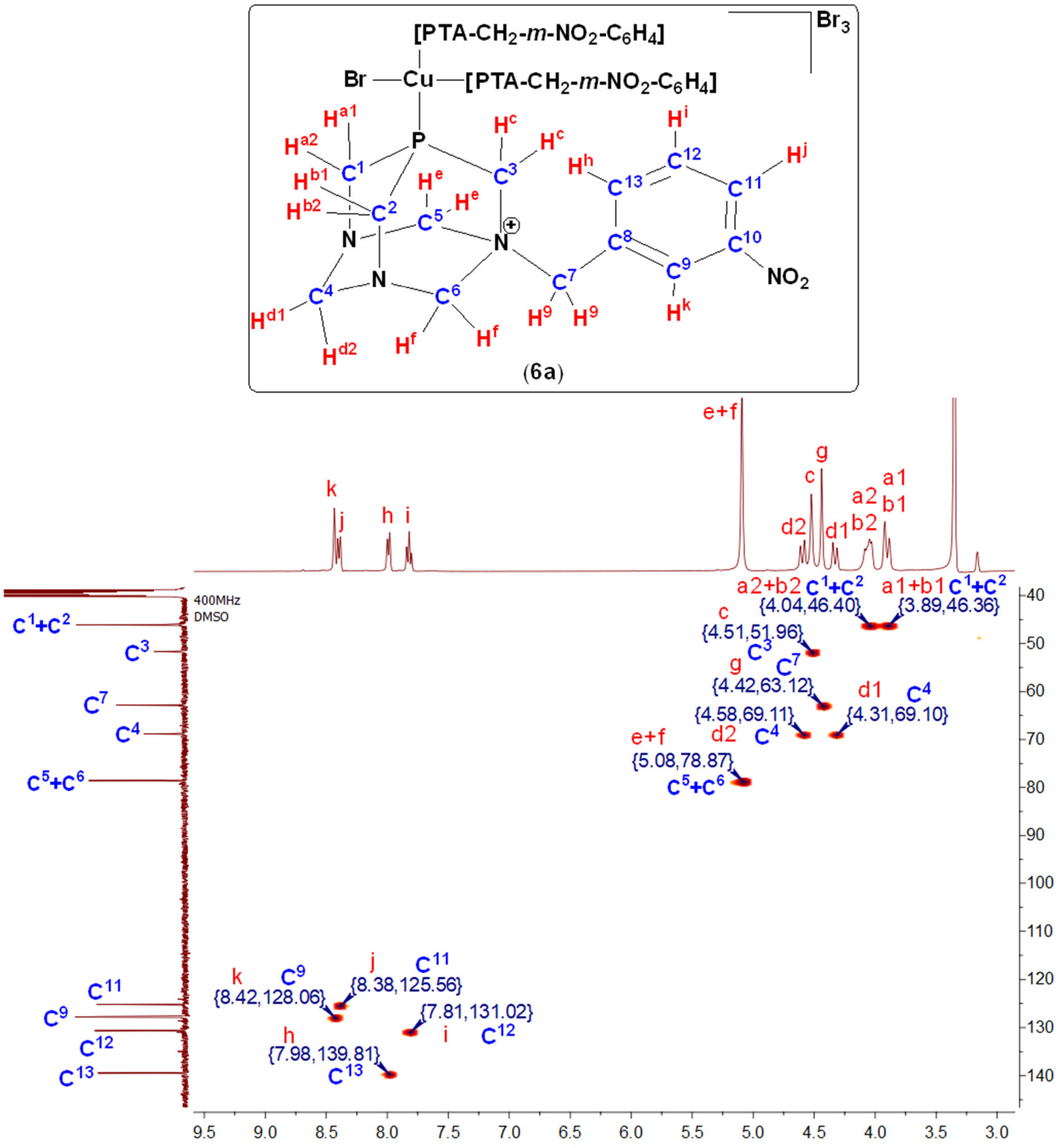

Figure S43. HSQC spectrum of [CuBr(PTA-CH$_2$-*m*-NO$_2$-C$_6$H$_4$)$_3$]·Br$_3$ (**6a**) in DMSO-d_6 (400 MHz).

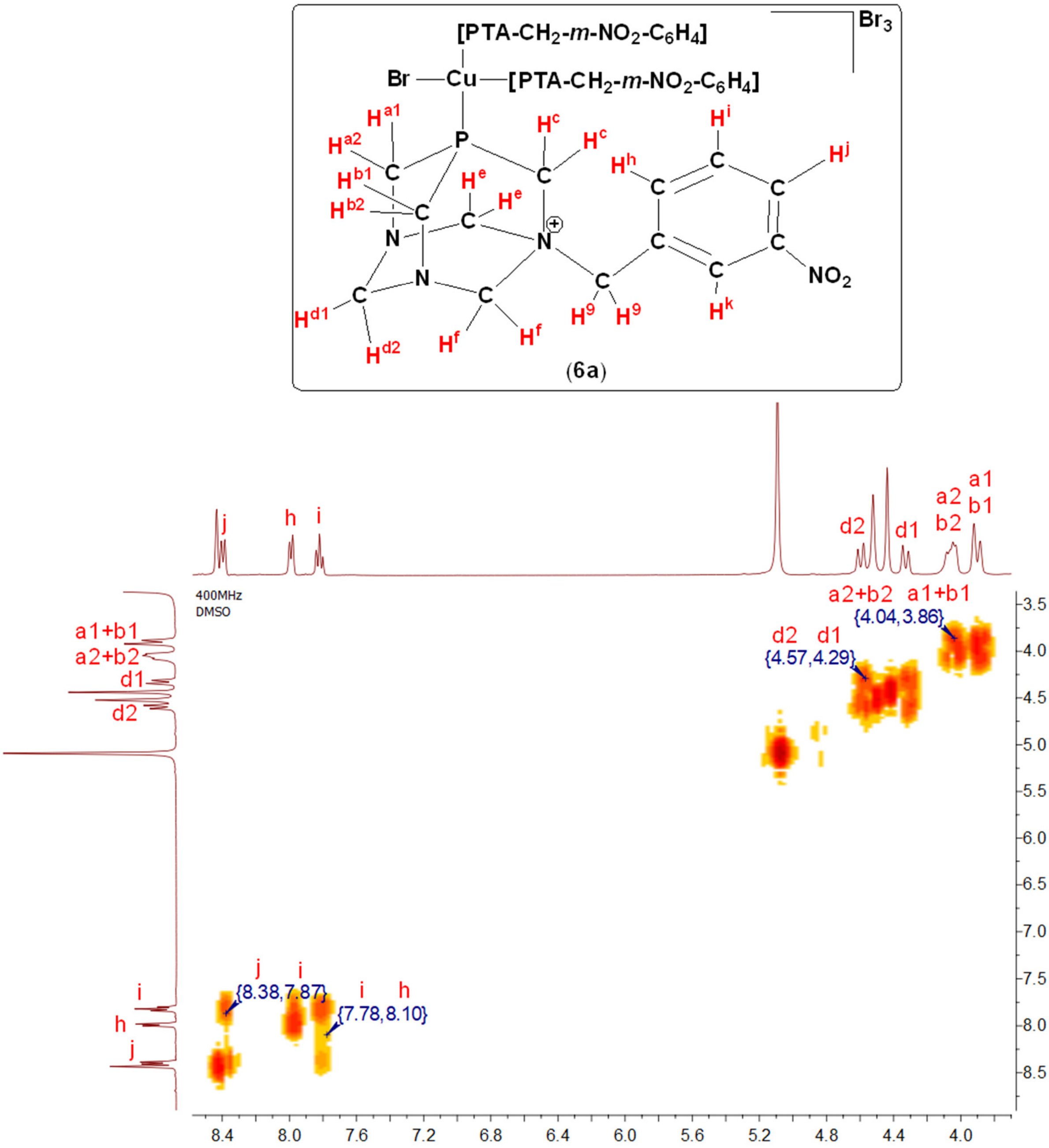

Figure S44. COSY spectrum of [CuBr(PTA-CH$_2$-*m*-NO$_2$-C$_6$H$_4$)$_3$]·Br$_3$ (**6a**) in DMSO-*d*$_6$ (400 MHz).

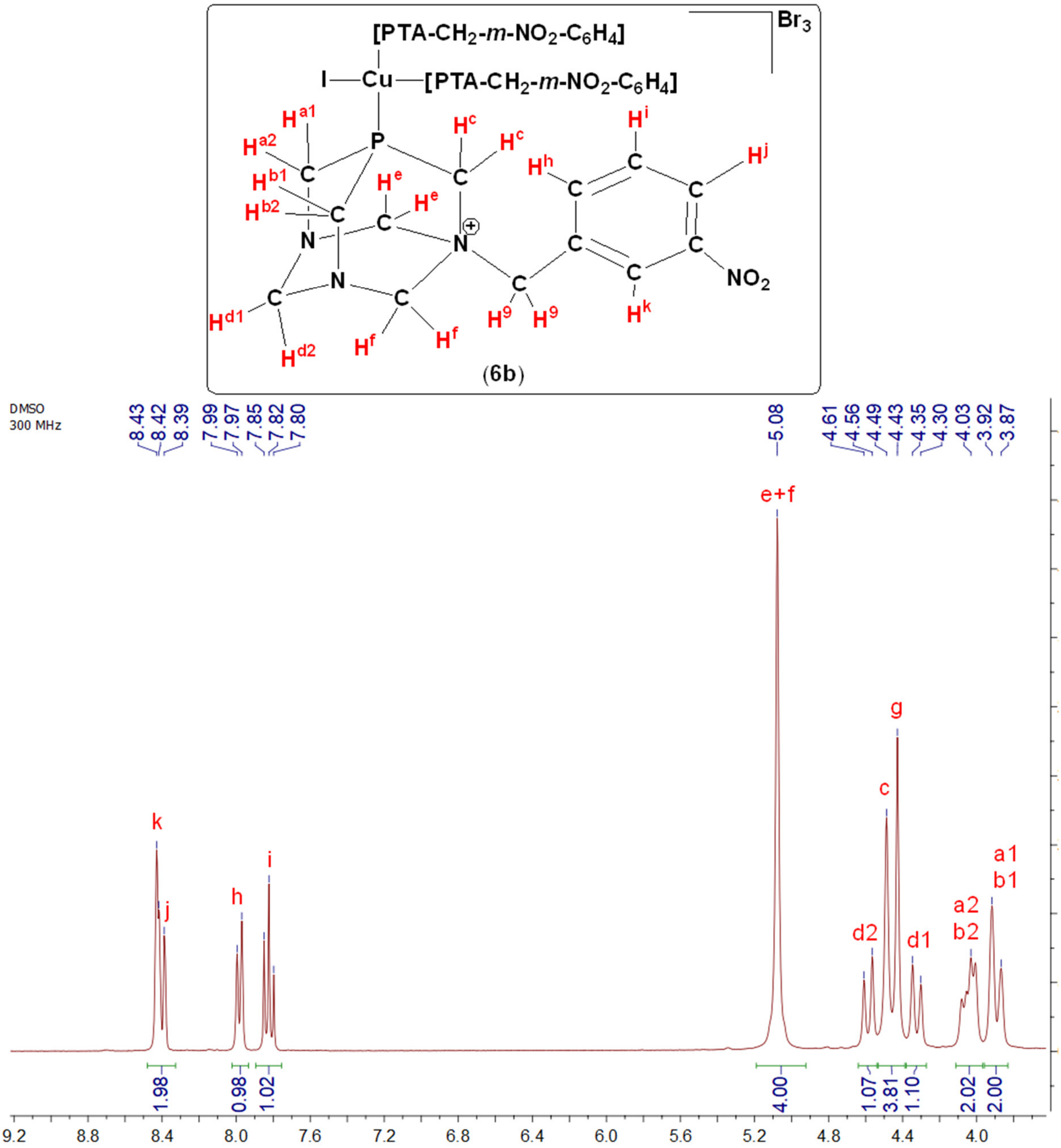

Figure S45. ^{1}H NMR spectrum of [CuI(PTA-CH$_2$-*m*-NO$_2$-C$_6$H$_4$)$_3$]·Br$_3$ (**6b**) in DMSO-d_6 (300 MHz).

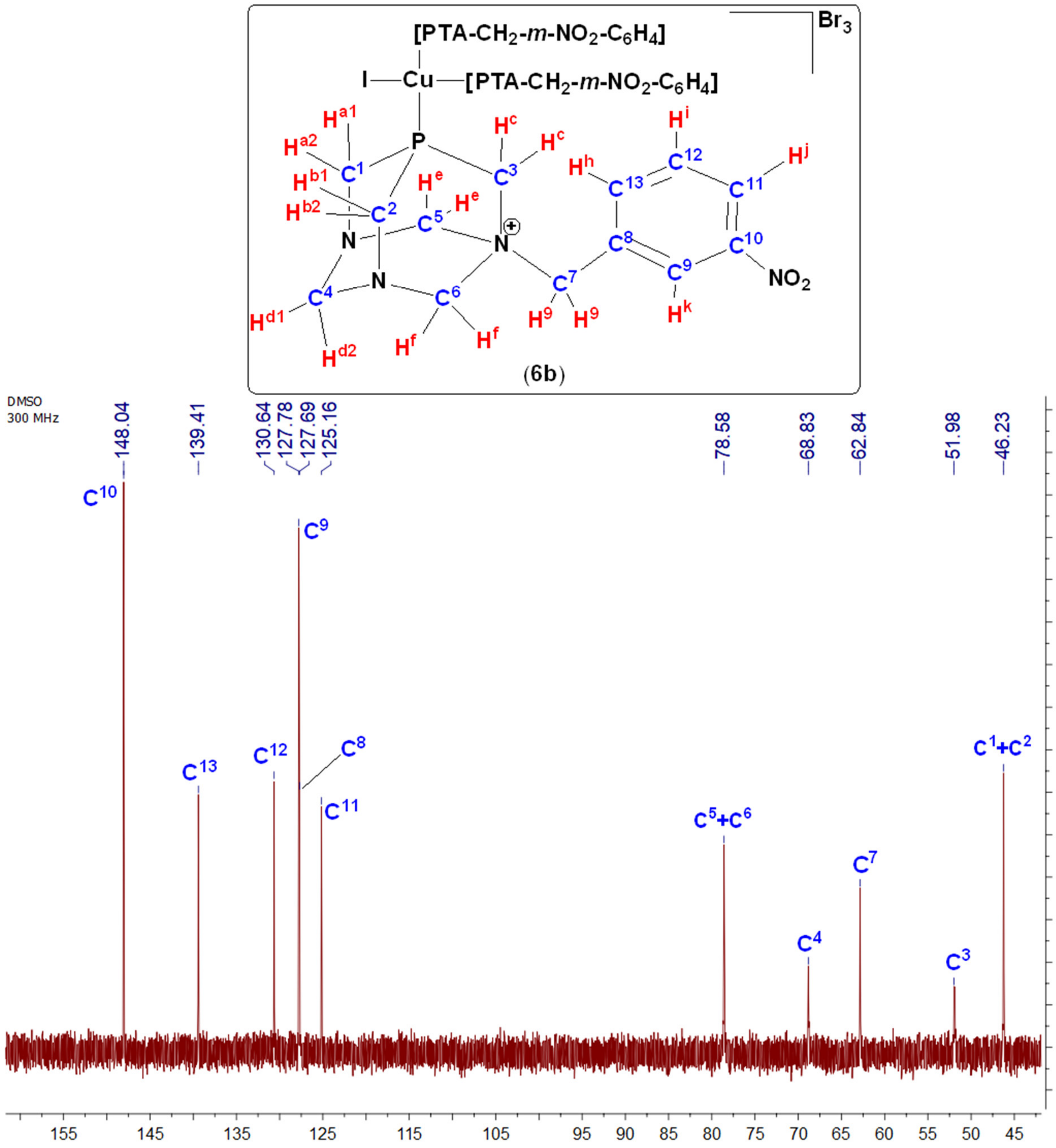

Figure S46. ^{13}C NMR spectrum of [CuI(PTA-CH$_2$-*m*-NO$_2$-C$_6$H$_4$)$_3$]·Br$_3$ (**6b**) in DMSO-*d*$_6$ (300 MHz).

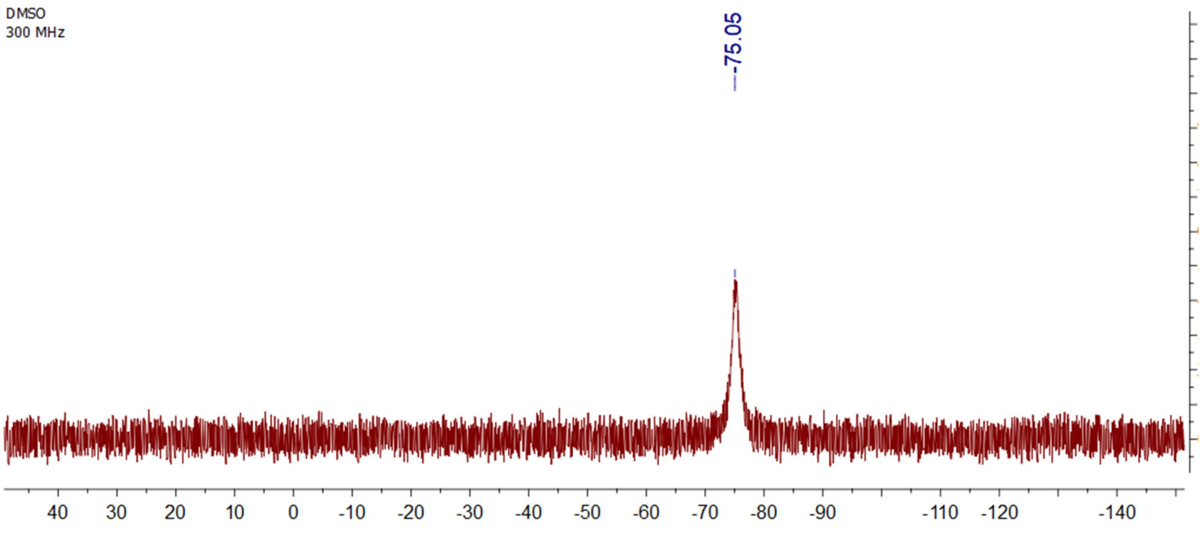

Figure S47. ^{31}P NMR spectrum of [CuI(PTA-CH_2-*m*-NO_2-C_6H_4)$_3$]·Br_3 (**6b**) in DMSO-*d*$_6$ (300 MHz).

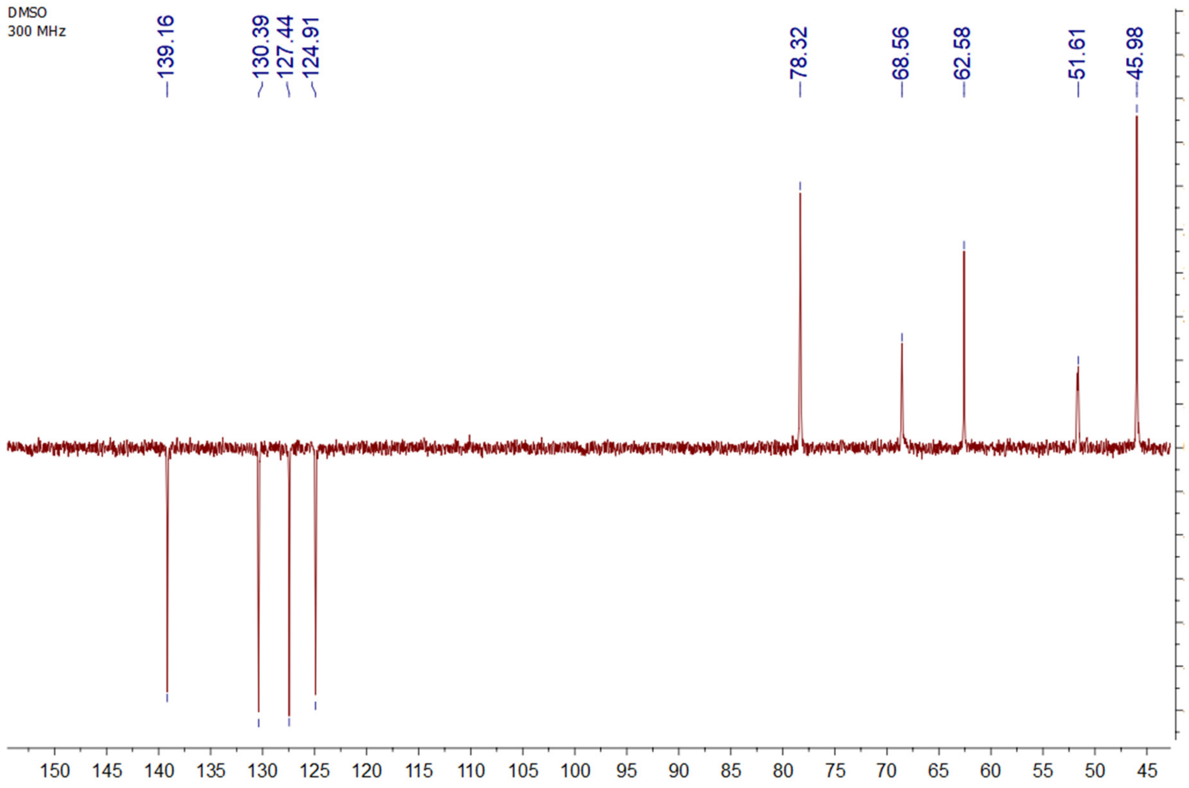

Figure S48. DEPT NMR spectrum of [CuI(PTA-CH_2-*m*-NO_2-C_6H_4)$_3$]·Br_3 (**6b**) in DMSO-*d*$_6$ (300 MHz).

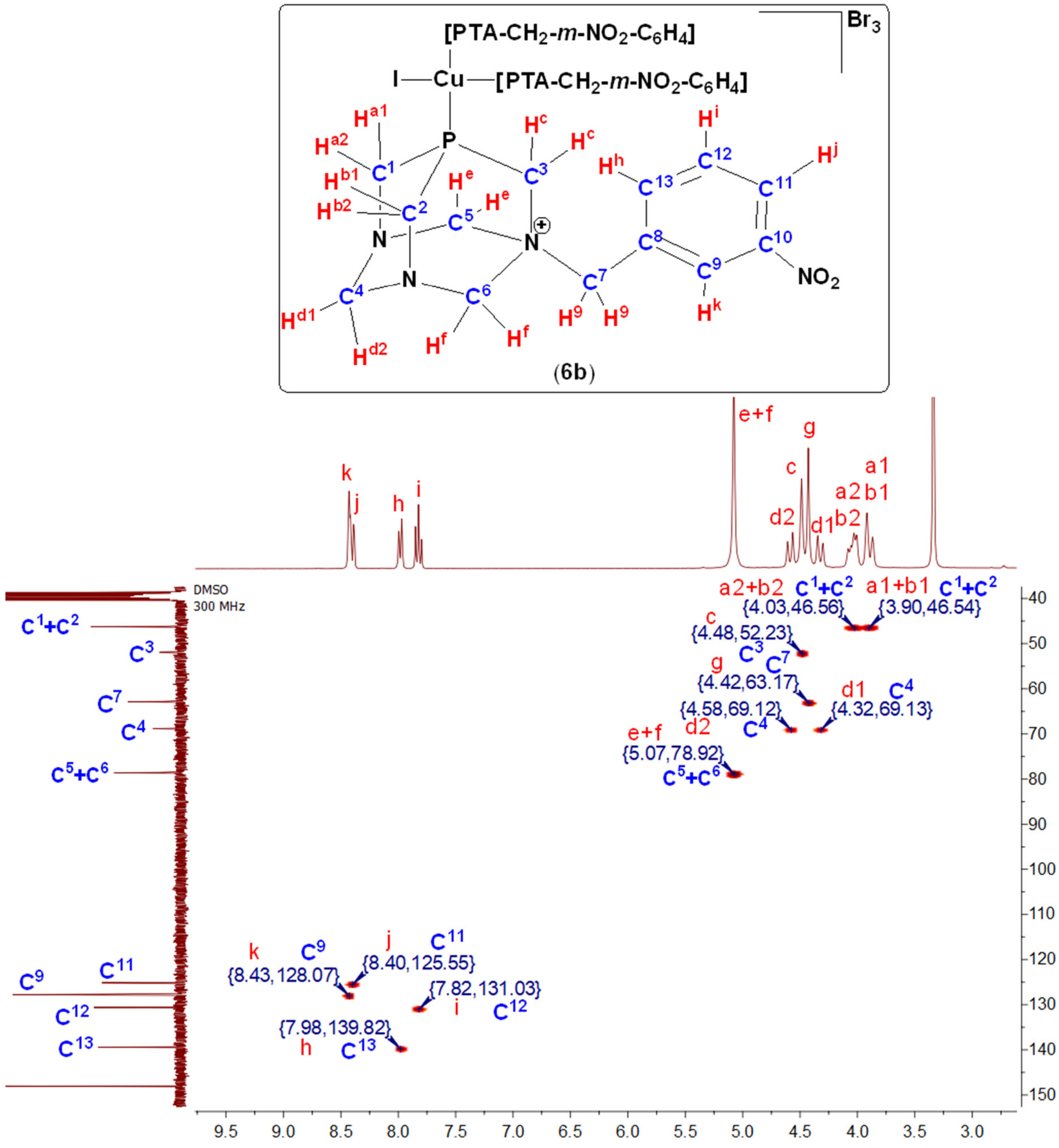

Figure S49. HSQC spectrum of [CuI(PTA-CH2-*m*-NO2-C6H4)3]·Br3 (**6b**) in DMSO-*d*6 (300 MHz).

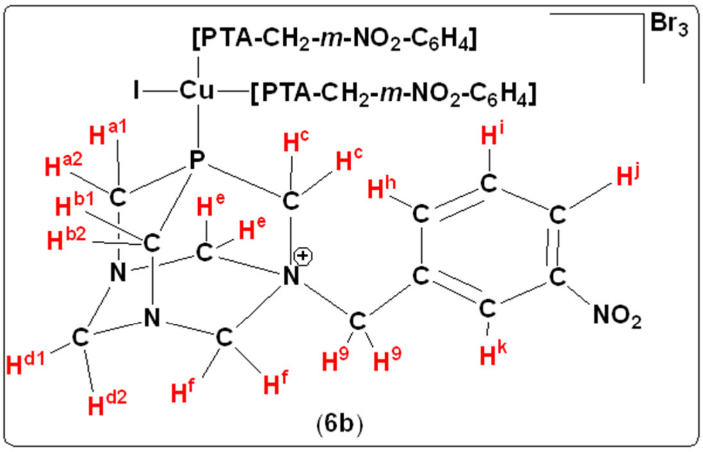

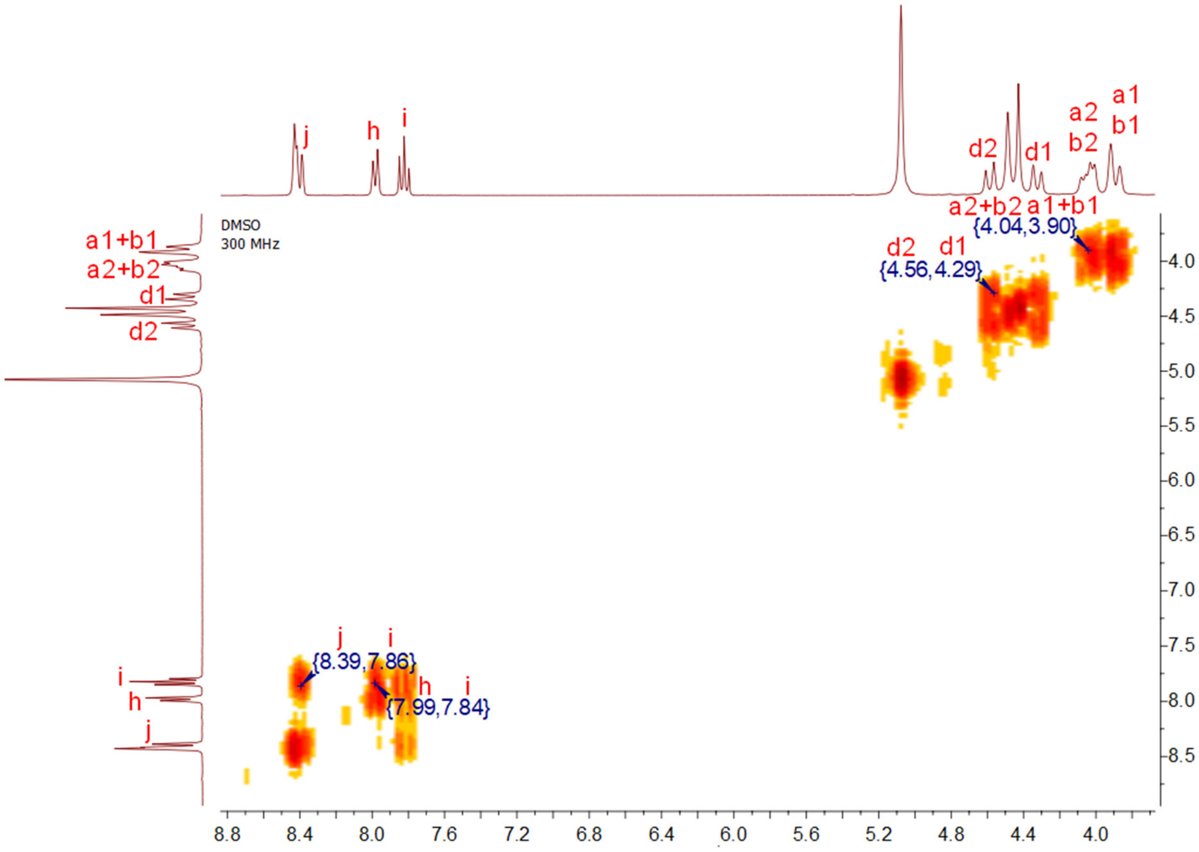

Figure S50. COSY spectrum of [CuI(PTA-CH$_2$-*m*-NO$_2$-C$_6$H$_4$)$_3$]·Br$_3$ (**6b**) in DMSO-d_6 (300 MHz).

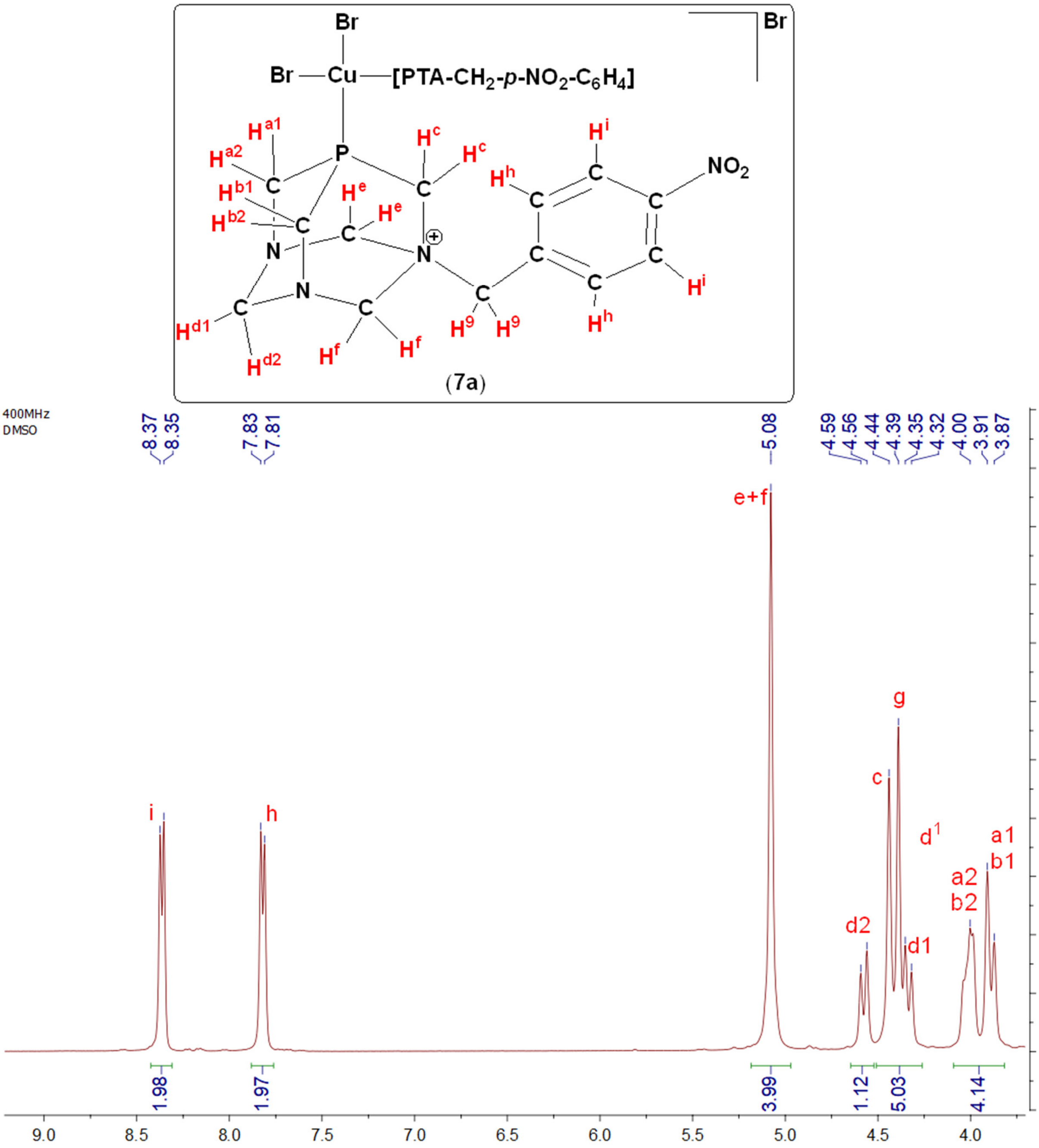

Figure S51. ^{1}H NMR spectrum of [CuBr$_2$(PTA-CH$_2$-*p*-NO$_2$-C$_6$H$_4$)$_2$]·Br (**7a**) in DMSO-d_6 (400 MHz).

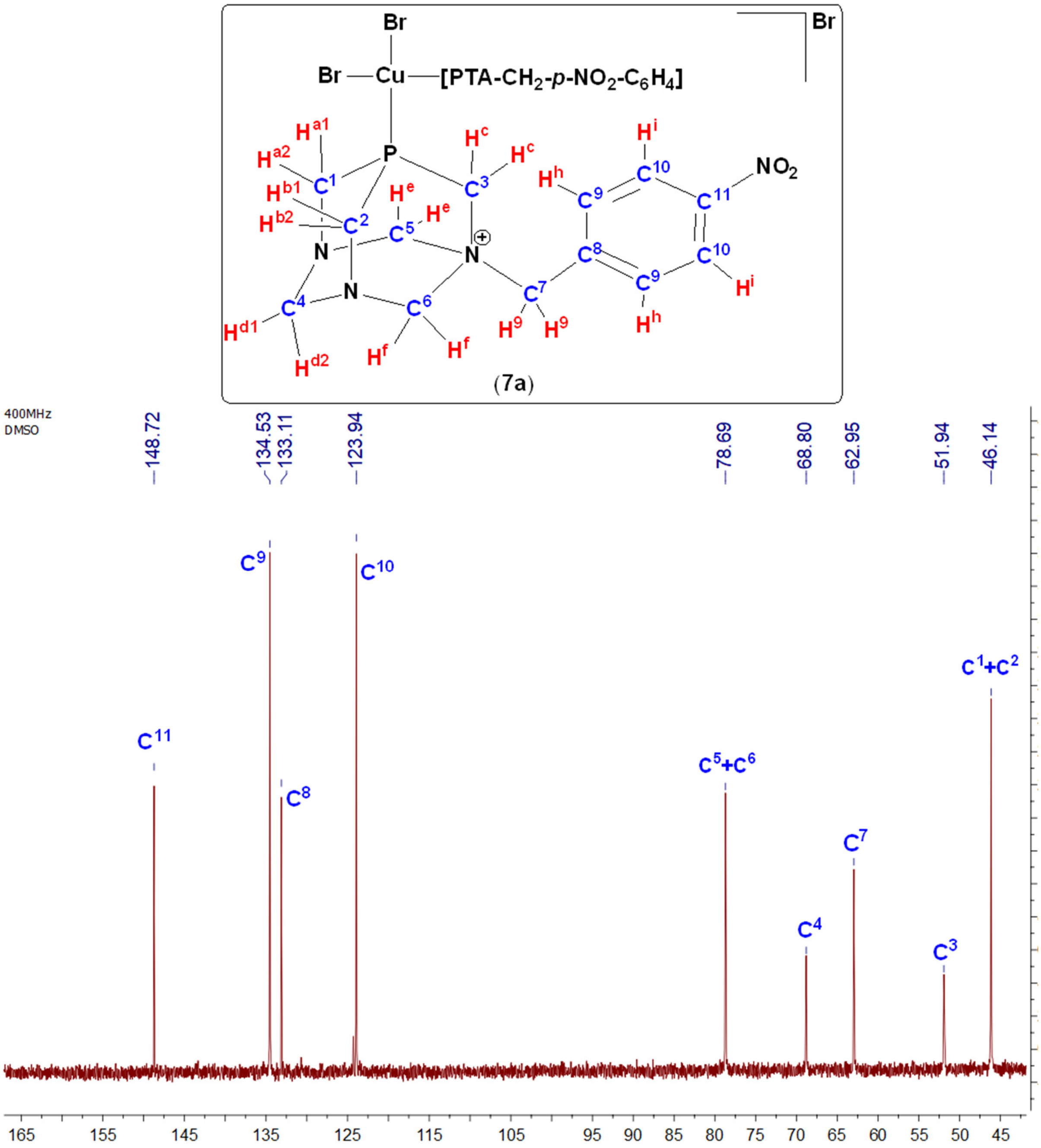

Figure S52. ^{13}C NMR spectrum of [$CuBr_2$(PTA-CH_2-*p*-NO_2-C_6H_4)$_2$]·Br (**7a**) in DMSO-d_6 (400 MHz).

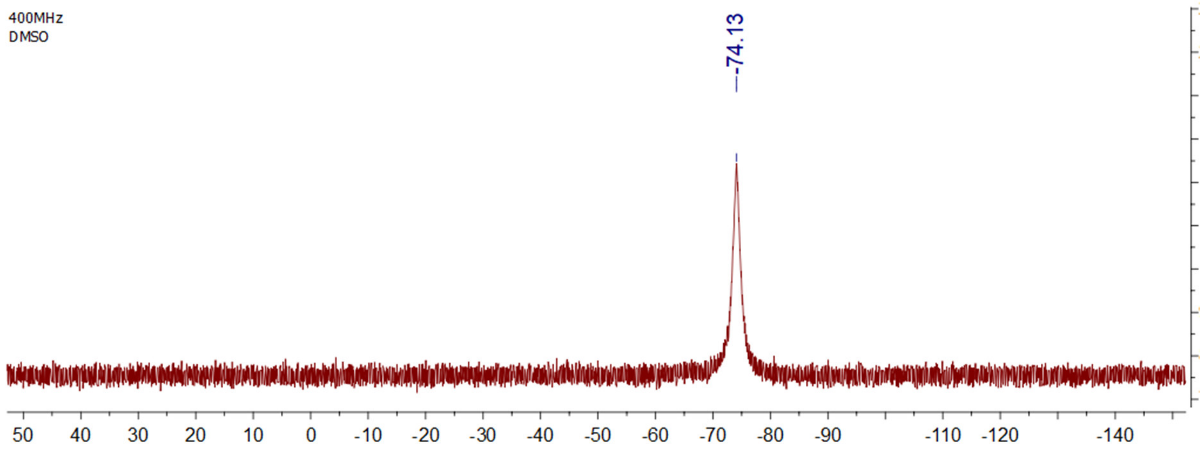

Figure S53. ^{31}P NMR spectrum of $[CuBr_2(PTA\text{-}CH_2\text{-}p\text{-}NO_2\text{-}C_6H_4)_2]\cdot Br$ (**7a**) in DMSO-d_6 (400 MHz).

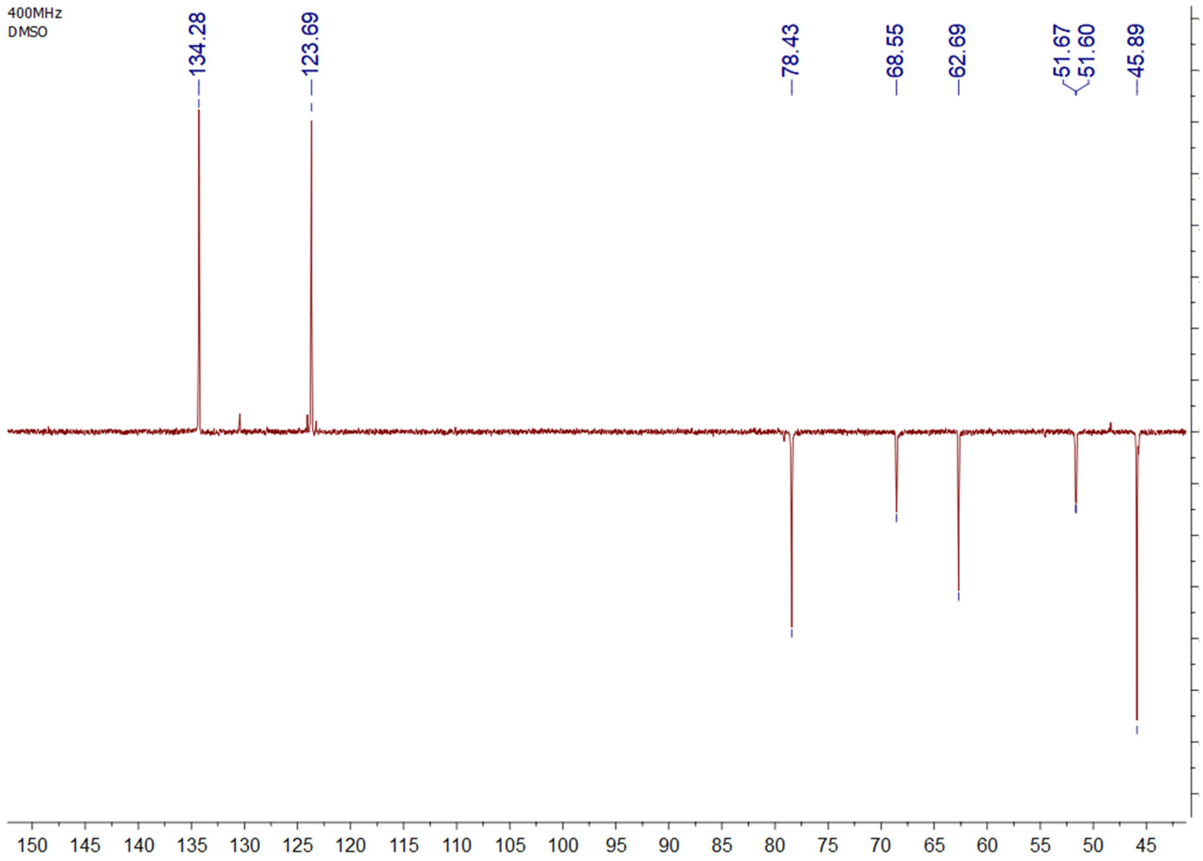

Figure S54. DEPT NMR spectrum of $[CuBr_2(PTA\text{-}CH_2\text{-}p\text{-}NO_2\text{-}C_6H_4)_2]\cdot Br$ (**7a**) in DMSO-d_6 (400 MHz).

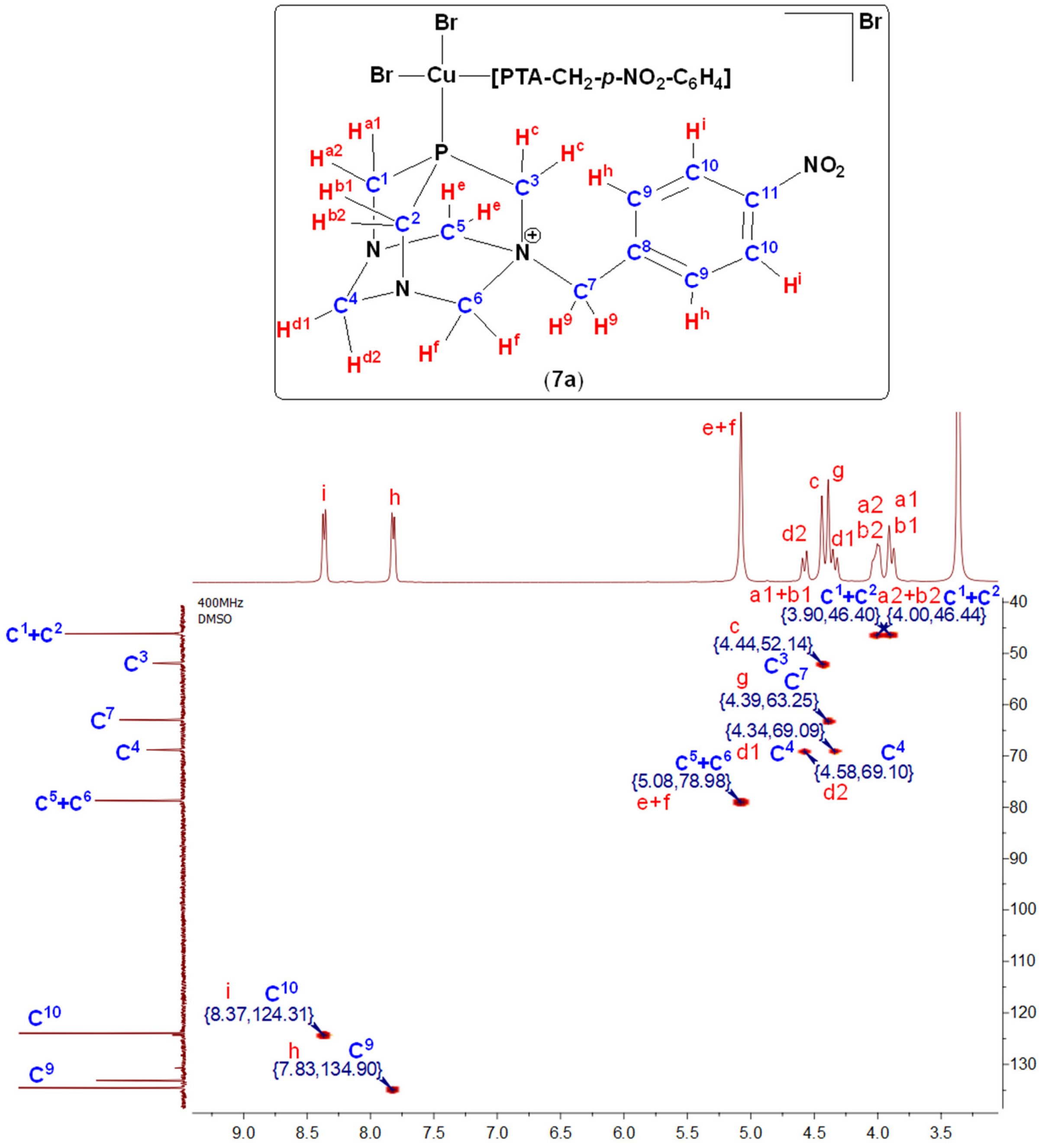

Figure S55. HSQC spectrum of [CuBr$_2$(PTA-CH$_2$-*p*-NO$_2$-C$_6$H$_4$)$_2$]·Br (**7a**) in DMSO-d_6 (400 MHz).

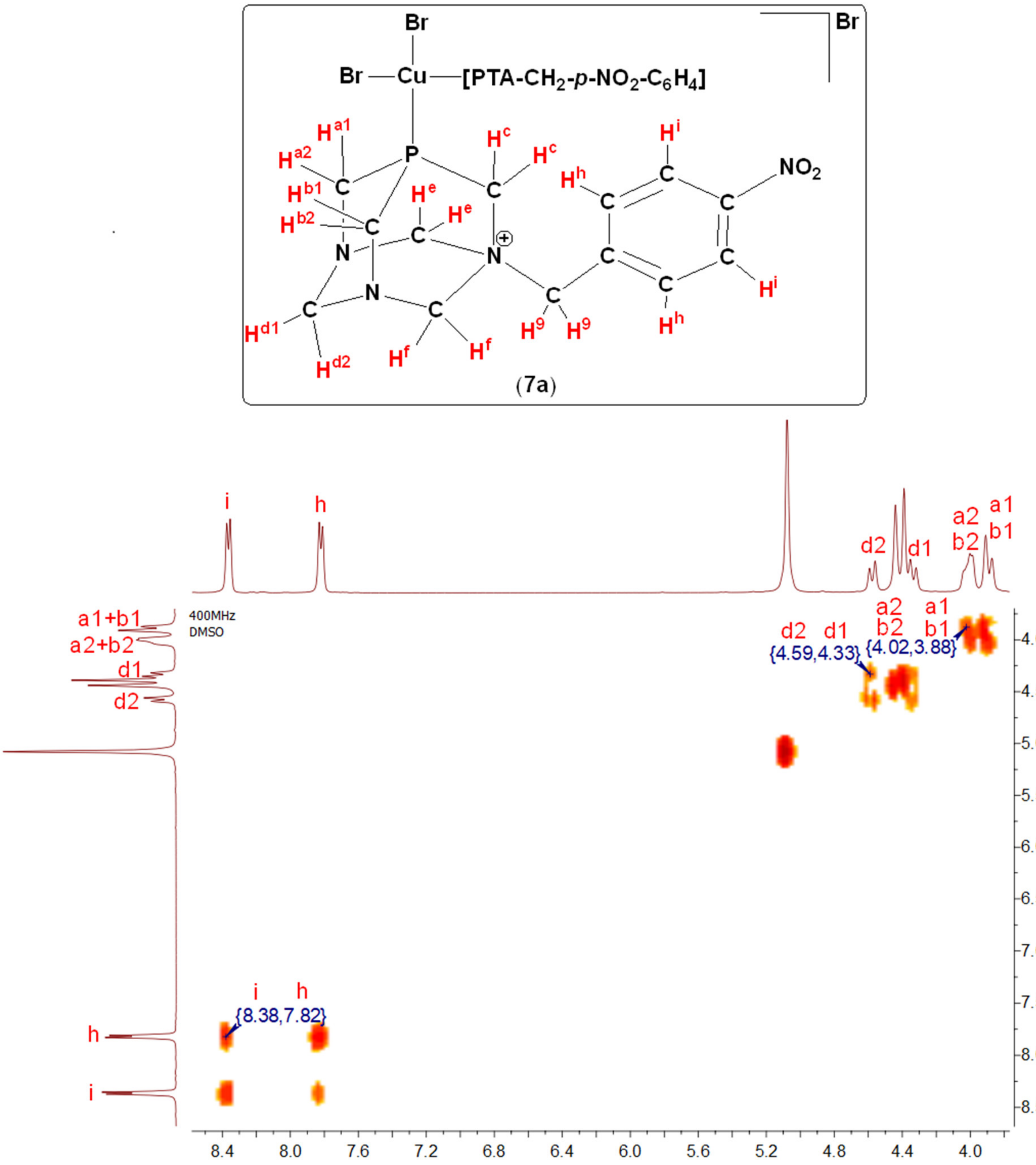

Figure S56. COSY spectrum of $[CuBr_2(PTA\text{-}CH_2\text{-}p\text{-}NO_2\text{-}C_6H_4)_2]\cdot Br$ (**7a**) in DMSO-d_6 (400 MHz).

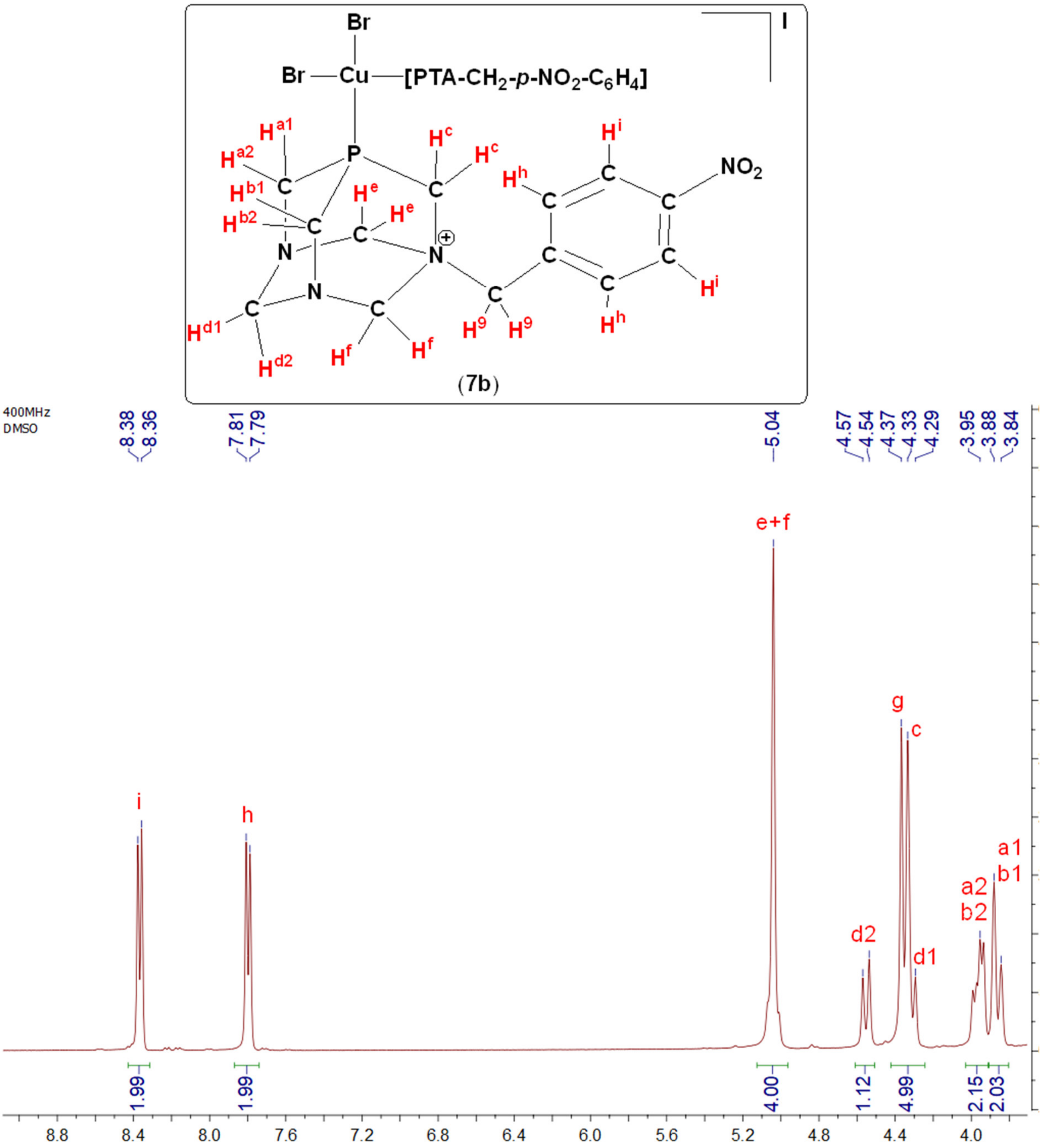

Figure S57. ^{1}H NMR spectrum of [CuBr$_2$(PTA-CH$_2$-*p*-NO$_2$-C$_6$H$_4$)$_2$]·I (**7b**) in DMSO-d_6 (400 MHz).

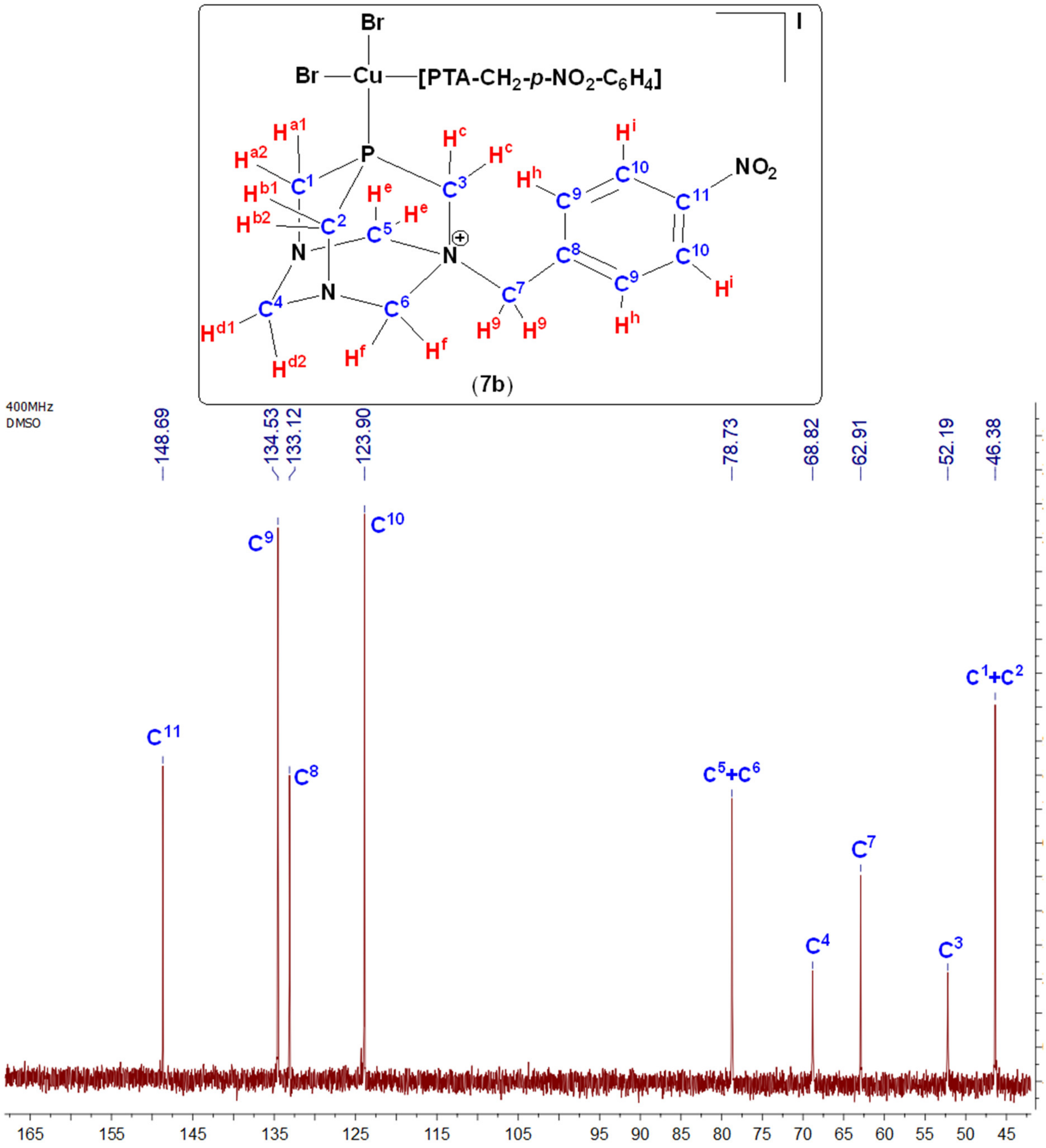

Figure S58. ^{13}C NMR spectrum of $[CuBr_2(PTA\text{-}CH_2\text{-}p\text{-}NO_2\text{-}C_6H_4)_2]\cdot I$ (**7b**) in DMSO-d_6 (400 MHz).

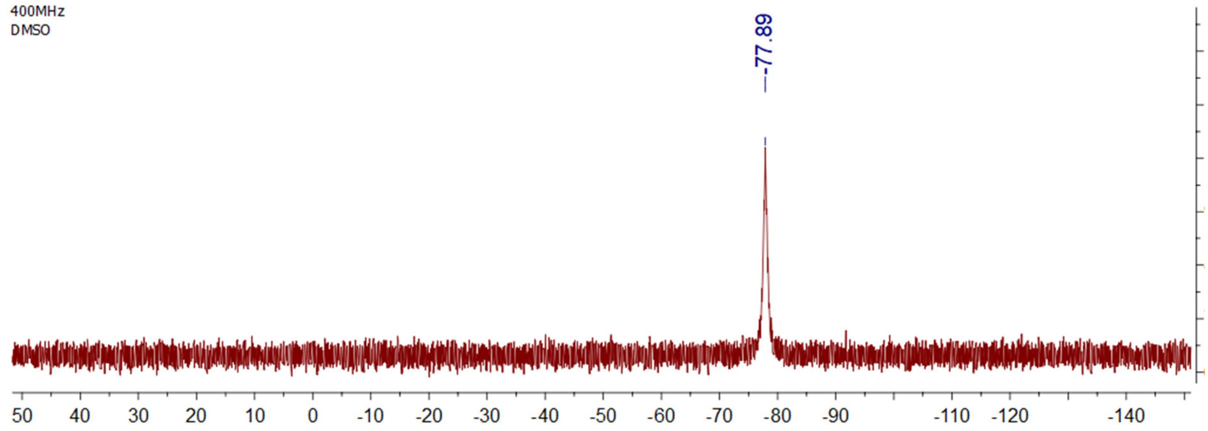

Figure S59. ^{31}P NMR spectrum of [$CuBr_2$(PTA-CH_2-*p*-NO_2-C_6H_4)$_2$]·I (**7b**) in DMSO-d_6 (400 MHz).

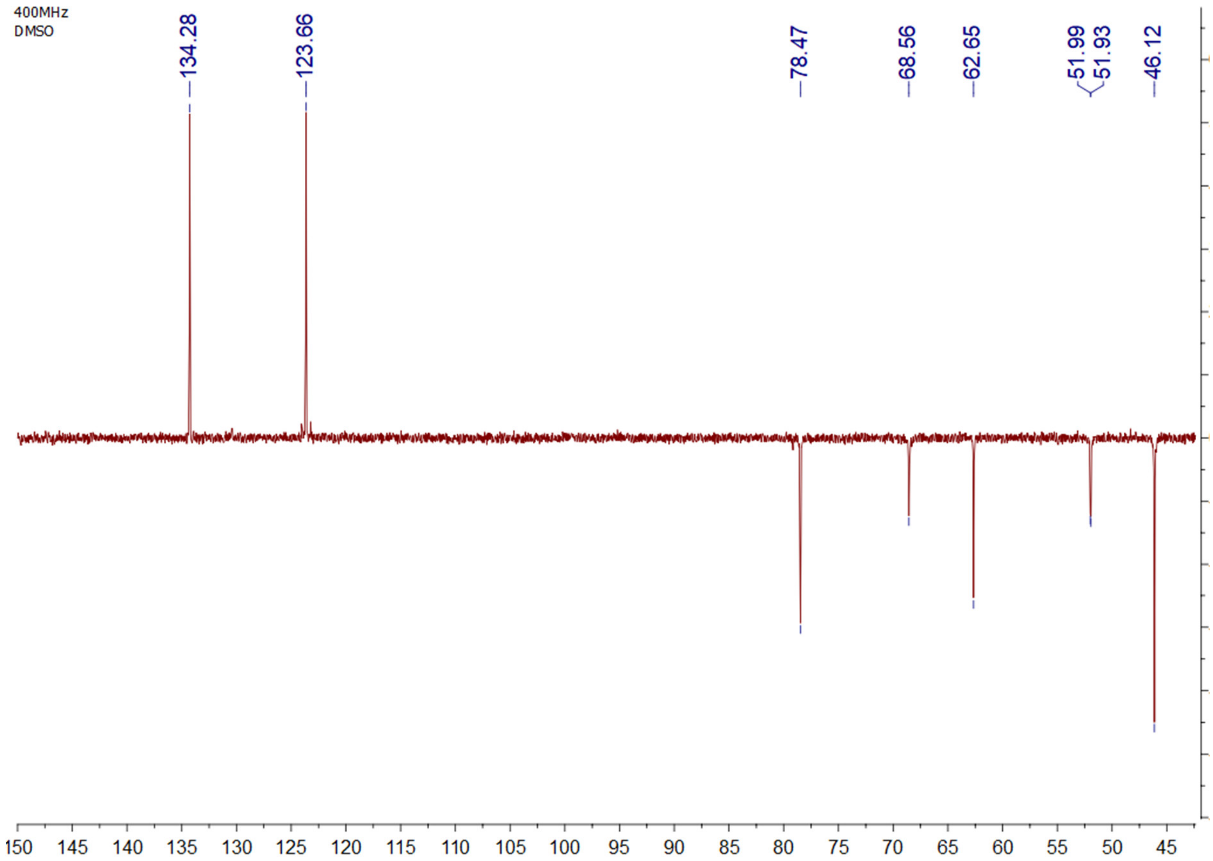

Figure S60. DEPT NMR spectrum of [$CuBr_2$(PTA-CH_2-*p*-NO_2-C_6H_4)$_2$]·I (**7b**) in DMSO-d_6 (400 MHz).

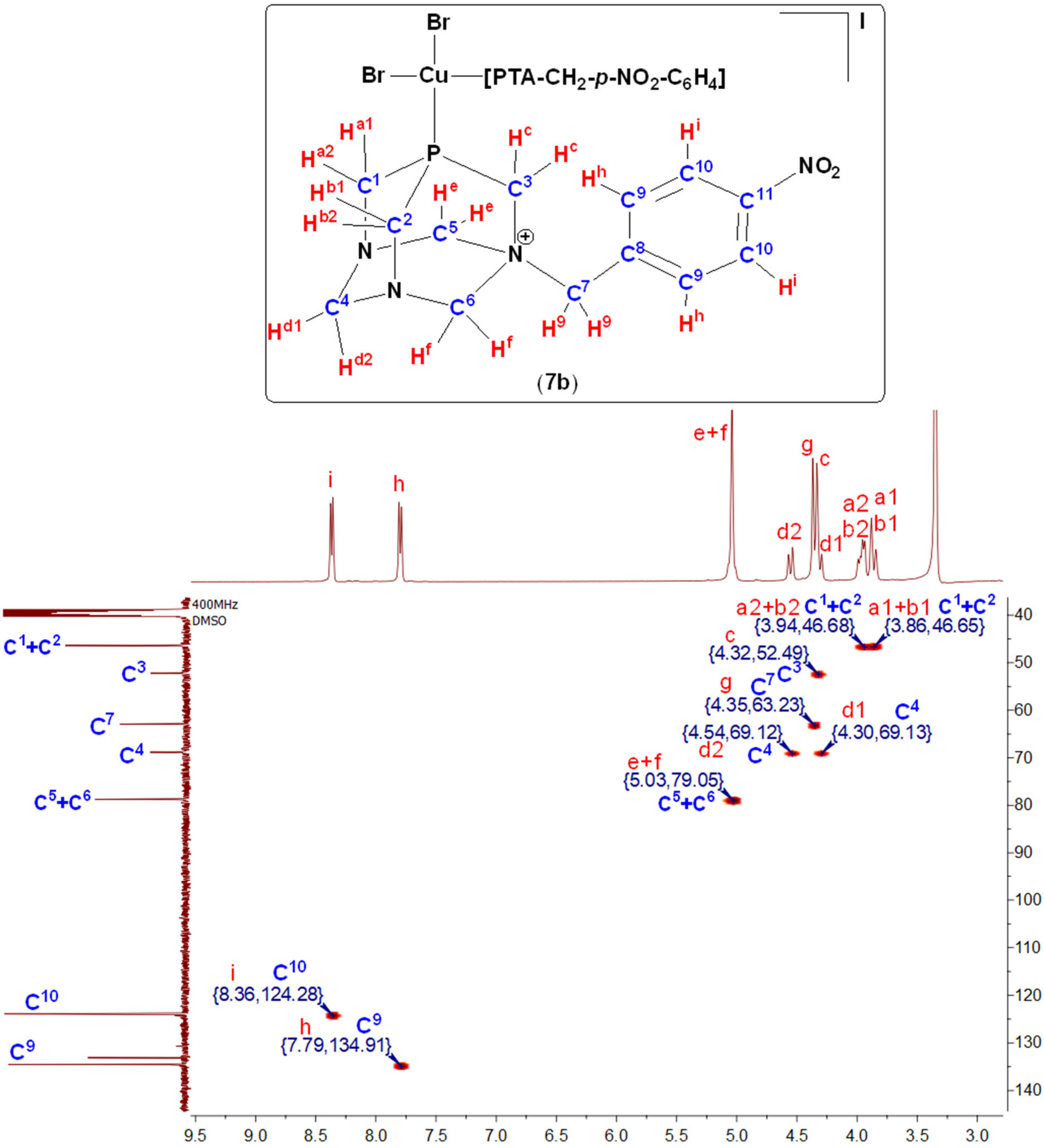

Figure S61. HSQC spectrum of [CuBr2(PTA-CH2-*p*-NO2-C6H4)2]·I (**7b**) in DMSO-*d*6 (400 MHz).

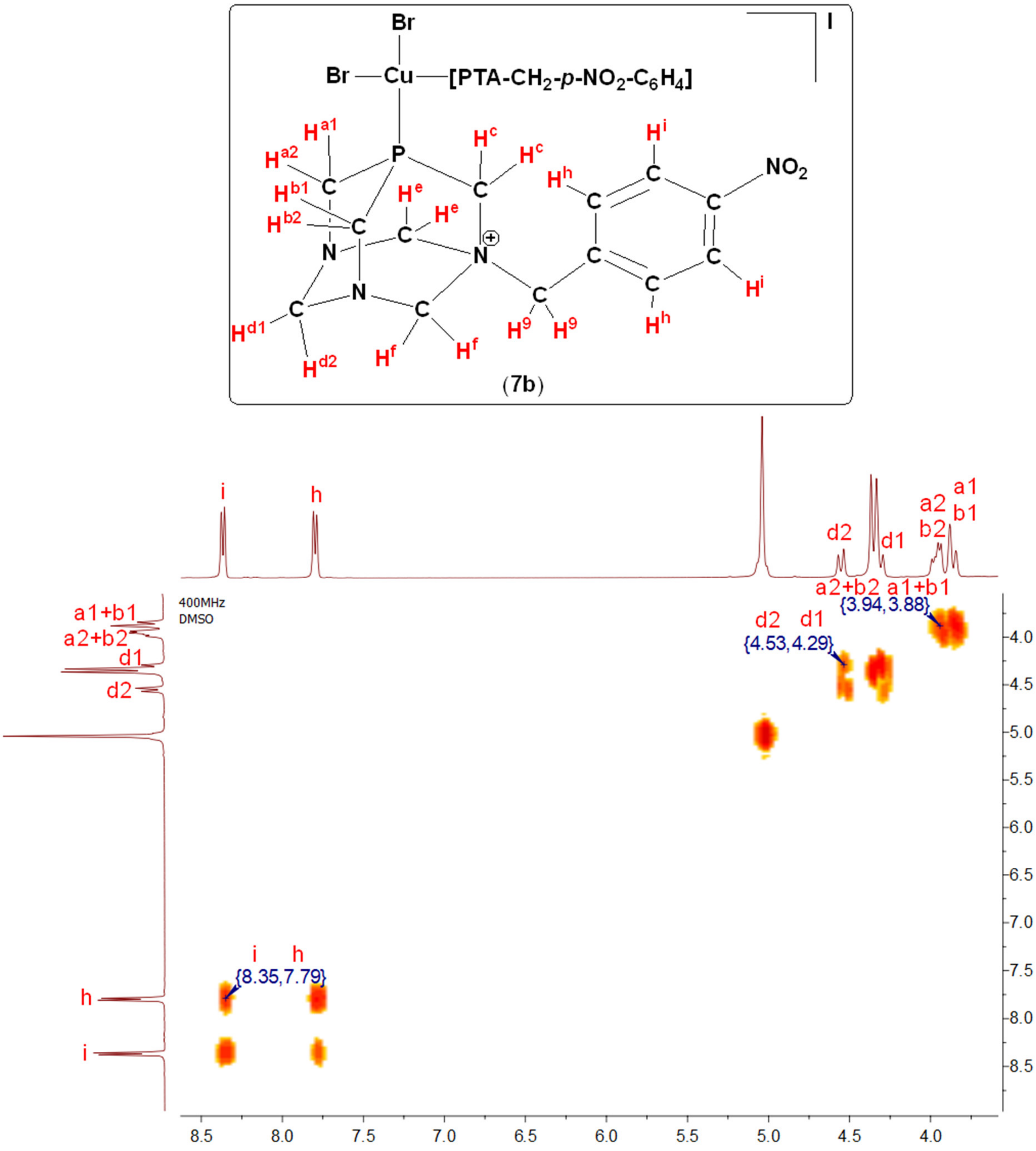

Figure S62. COSY spectrum of [$CuBr_2(PTA\text{-}CH_2\text{-}p\text{-}NO_2\text{-}C_6H_4)_2$]·I (**7b**) in DMSO-$d_6$ (400 MHz).

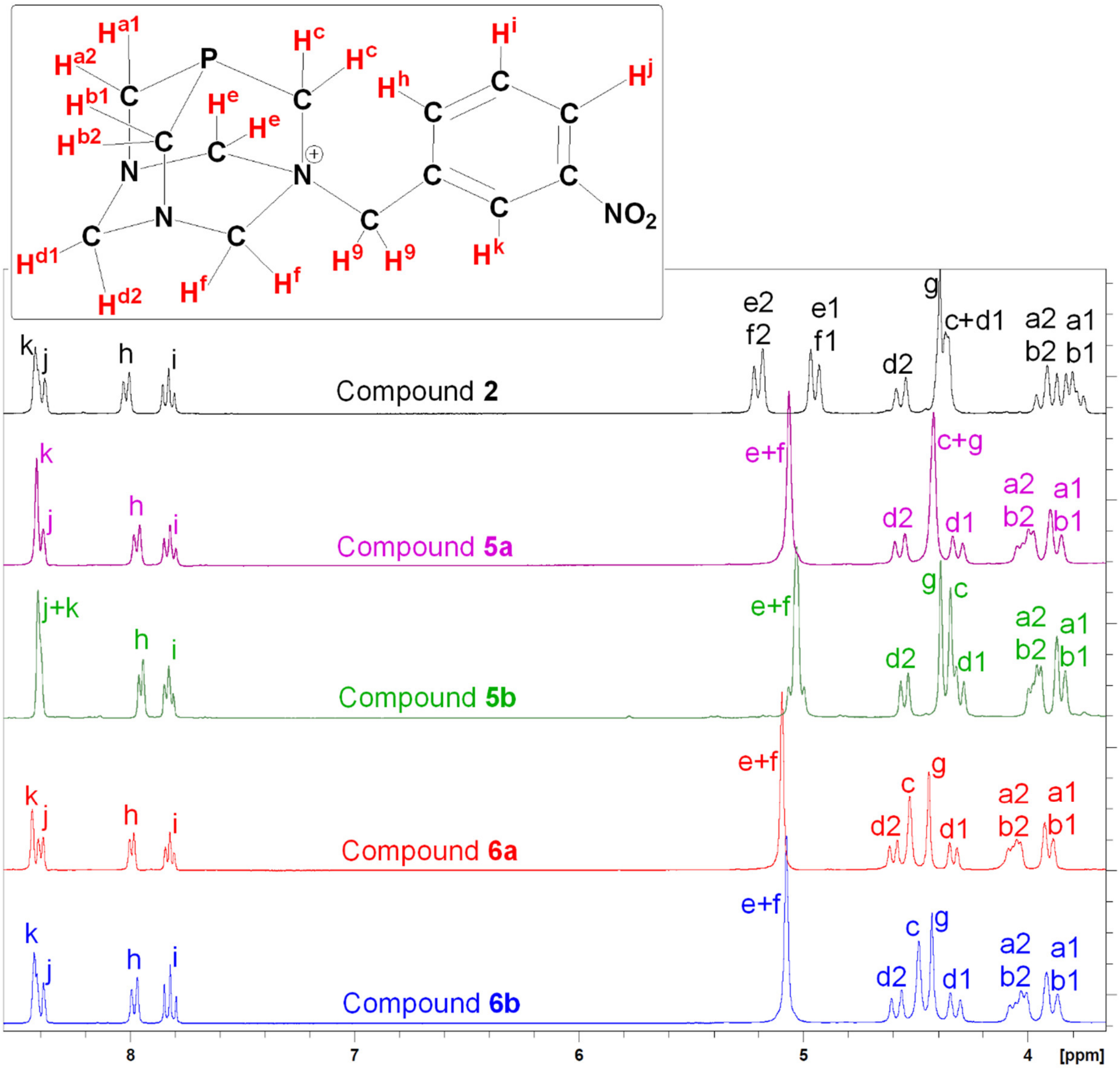

Figure 63. ^{1}H NMR spectra of the free ligand **2** and its copper complexes **5a**-**6b** in DMSO-d_6.

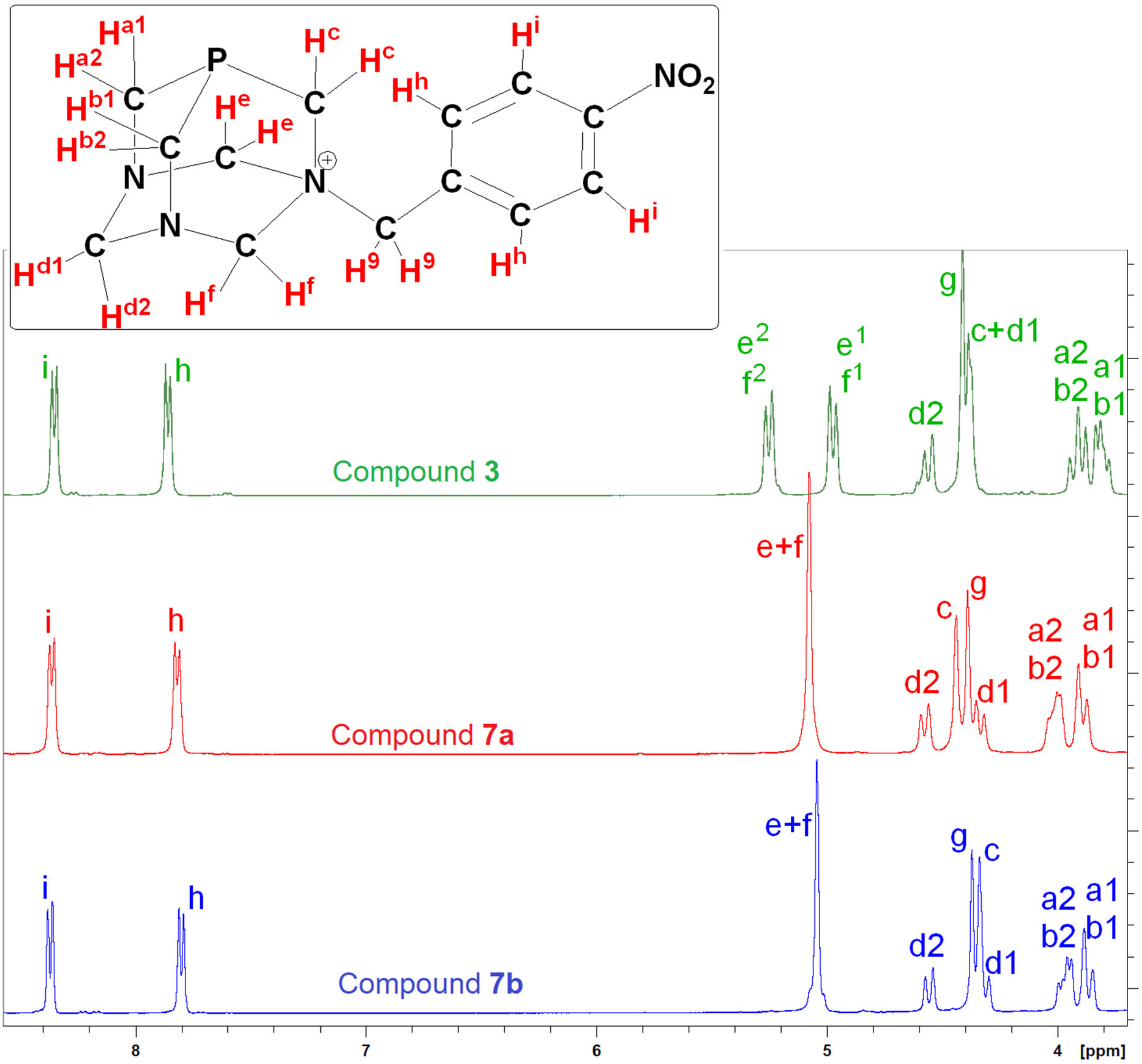

Figure S64. ^{1}H NMR spectra of (PTA-CH_2-*p*-NO_2-C_6H_4)Br (**3**) and its copper complexes **7a** and **7b** in DMSO-d_6.

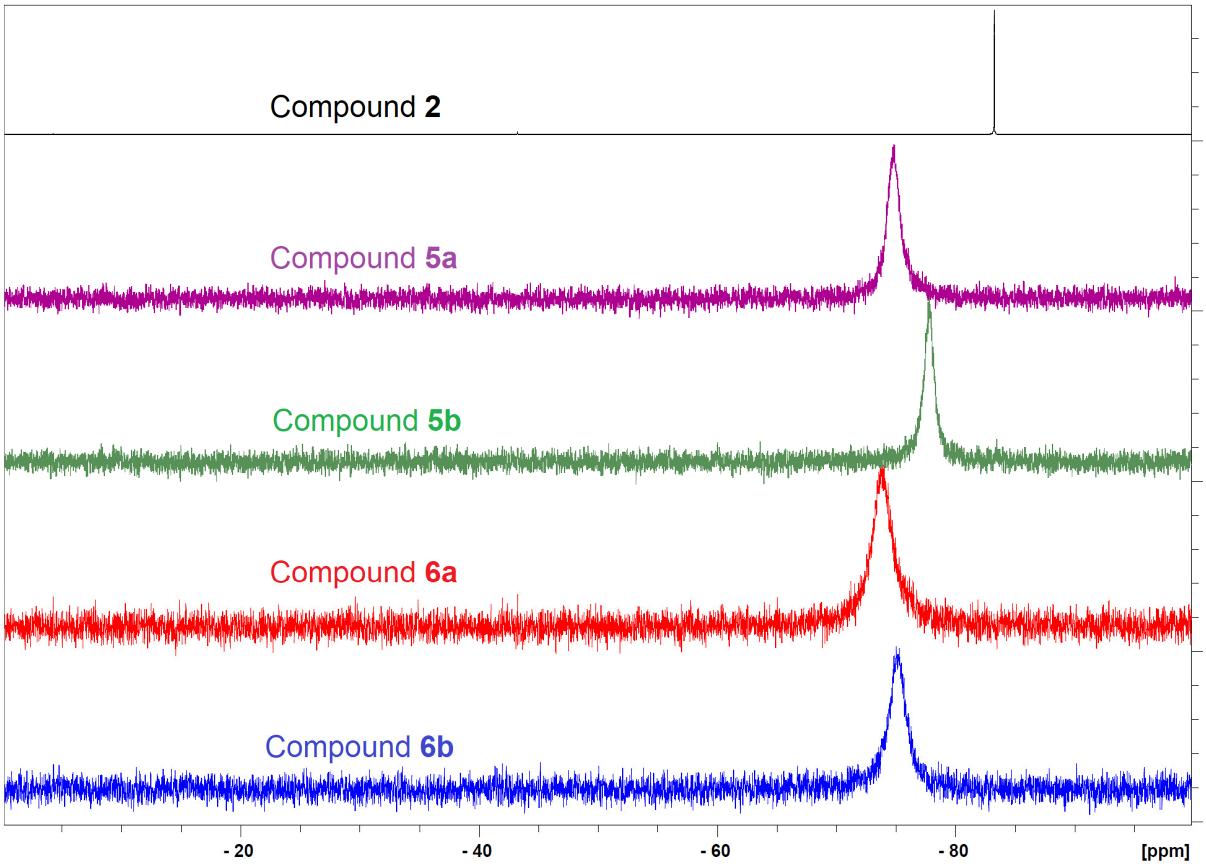

Figure S65. ^{31}P NMR spectra of (PTA-CH_2-*m*-NO_2-C_6H_4)Br (**2**) and its copper complexes **5a**-**6b** in DMSO-d_6.

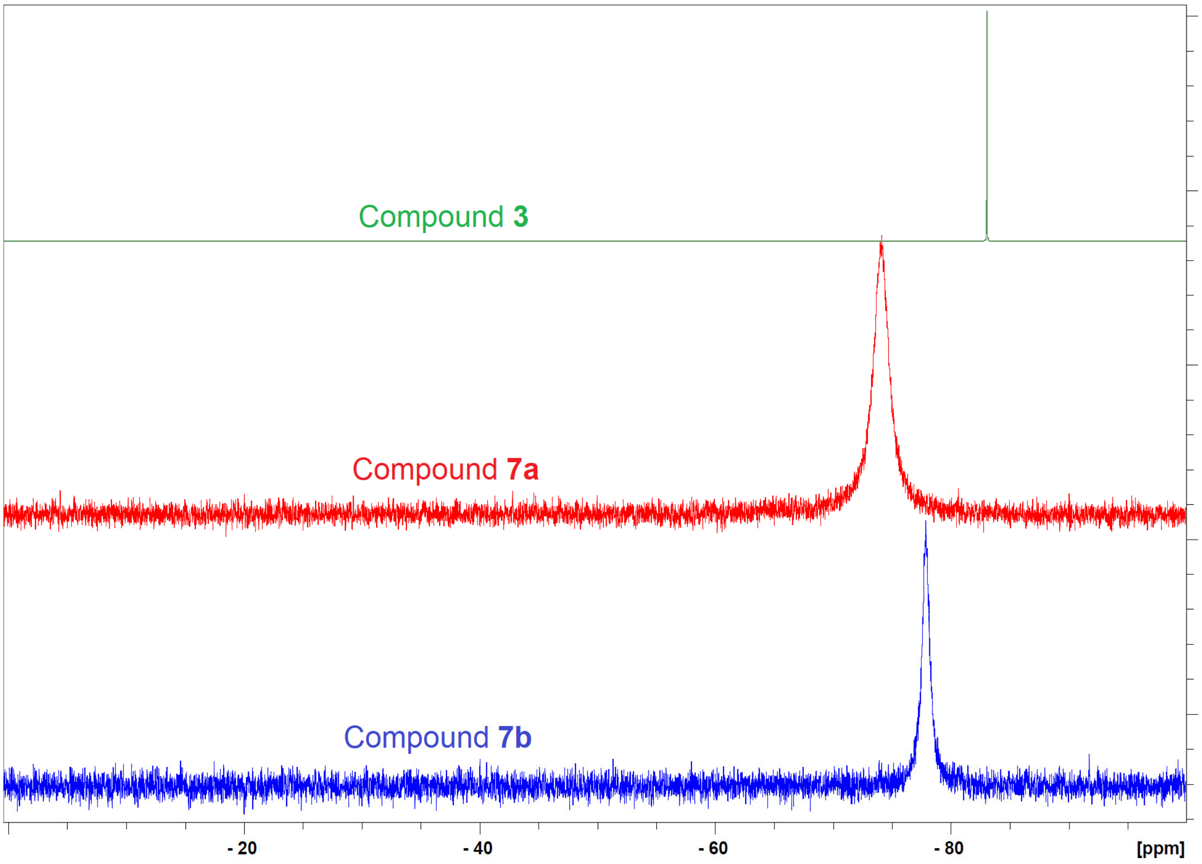

Figure S66. ^{31}P NMR spectra of (PTA-CH_2-*p*-NO_2-C_6H_4)Br (**3**) and its copper complexes **7a** and **7b** in DMSO-d_6.

2. X-Ray Data and Analysis

Table S1. Crystallographic data and structure refinement details for **2** and **3**.

	**2**	**3**	**5a**·$3H_2O$
Empirical formula	$C_{13}H_{18}BrN_4O_2P$	$C_{28}H_{60}Br_5CuN_{12}P_4$	$C_{26}H_{42}Br_3CuN_8O_7P_2$
Formula Weight	373.19	1151.85	943.88
Crystal system	Monoclinic	Triclinic	Orthorhombic
Space group	P 21/n	P -1	P n m a
Temperature/K	298(2)	298(2)	296(2)
a/Å	7.8684(4)	13.1151(10)	11.6280(5)
b/Å	11.4752(5)	13.3747(13)	16.0923(6)
c/Å	16.8805(7)	14.5809(9)	18.8040(8)
α/°	90	110.207(3)	90
β/°	90.996(2)	95.396(6)	90
γ/°	90	96.922(6)	90
V (Å^3)	1523.93(12)	2357.3(3)	3518.6(3)
Z	4	2	4
D_{calc} (g cm^{-3})	1.627	1.623	1.782
F000	760	1152	1896
μ(Mo Kα) (mm^{-1})	2.812	4.871	4.173
Rfls. collected/unique/observed	21169/2803/2218	64428/8688/6895	53275/3348/2635
R_{int}	0.0607	0.0749	0.0784
Final $R1^a$, $wR2^b$ ($I \geq 2\sigma$)	0.0398, 0.1051	0.0630, 0.1915	0.0345, 0.0787
Goodness-of-fit on F^2	1.042	0.722	1.074

$^a R = \Sigma||F_o|-|F_c||/\Sigma|F_o|$; $^b wR(F^2) = [\Sigma w(|F_o|^2 - |F_c|^2)^2/\Sigma w|F_o|^4]^{½}$.

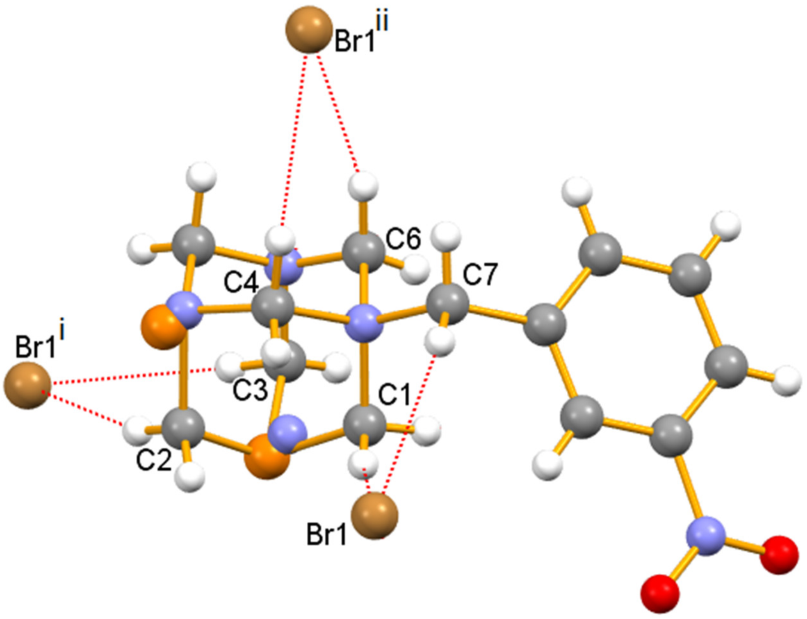

Figure S67. Intermolecular interactions between the cation and anion moieties of compound **2** (i: -1/2-x,1/2+y,1.5-z, ii: 1/2-x,1/2+y,1.5-z).

ligand.

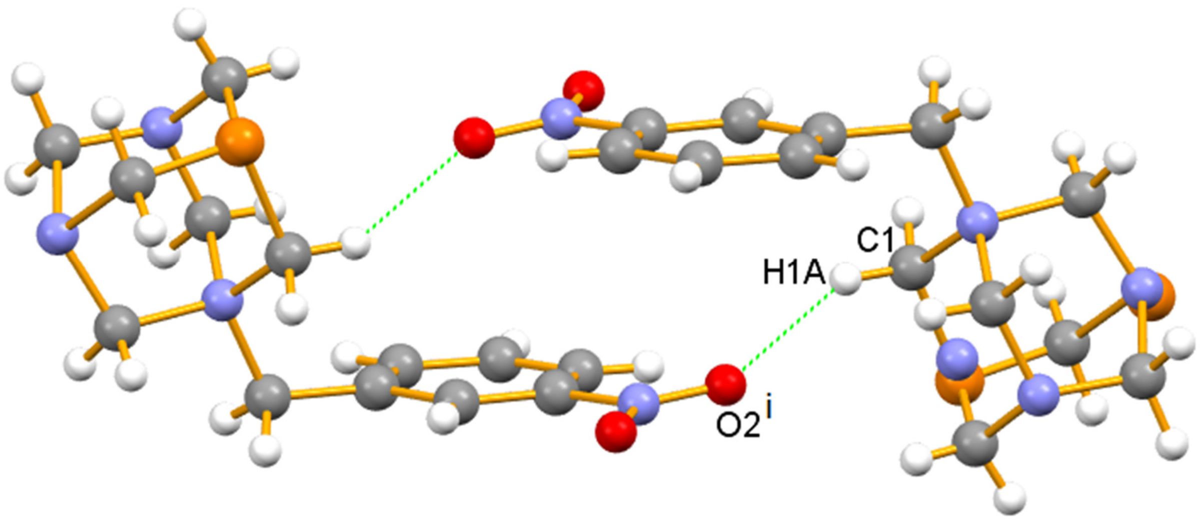

Figure S68. Intermolecular hydrogen bonding between two cations of compound **2** (i: -x,-y,1-z).

3. Plausible Anchorage of Cationic Cu(I) Complexes [CuBr$_2$(PTA-CH$_2$-*m*-NO$_2$-C$_6$H$_4$)$_2$]$^+$ on Carbon Materials

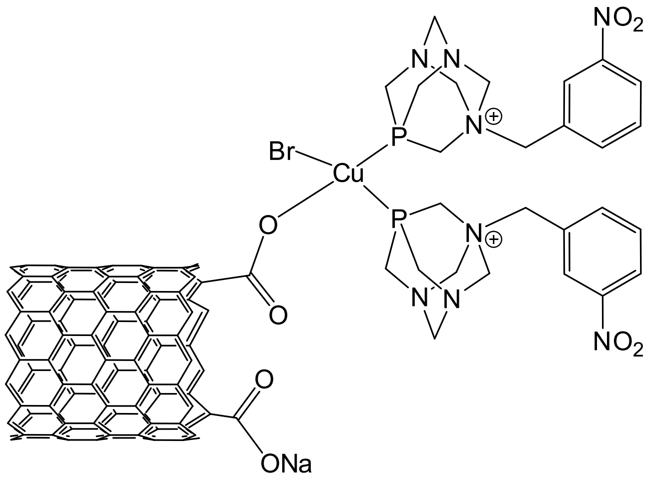

Figure S69. Coordination at the Cu centre through a carboxylate group upon replacement of a bromo ligand.

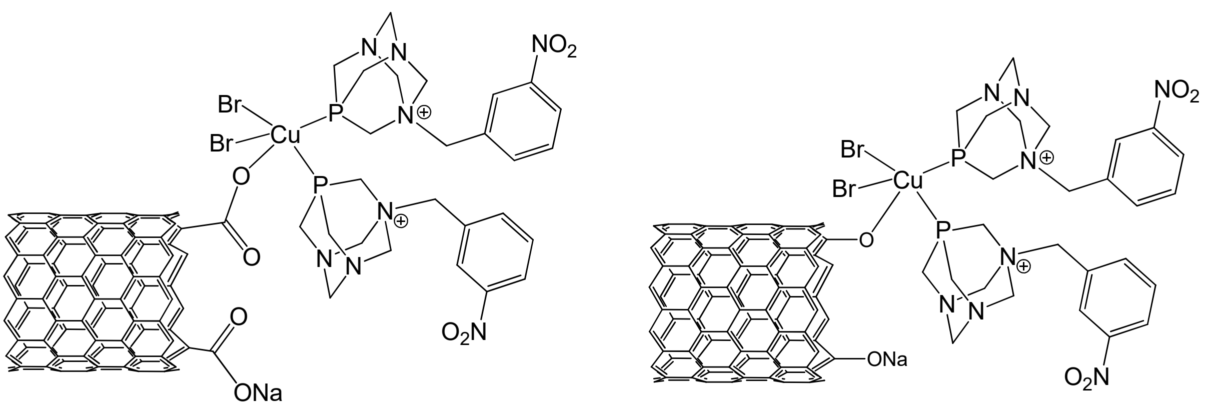

Figure S70. Coordination at the Cu centre through a carboxylate or a phenolate group.

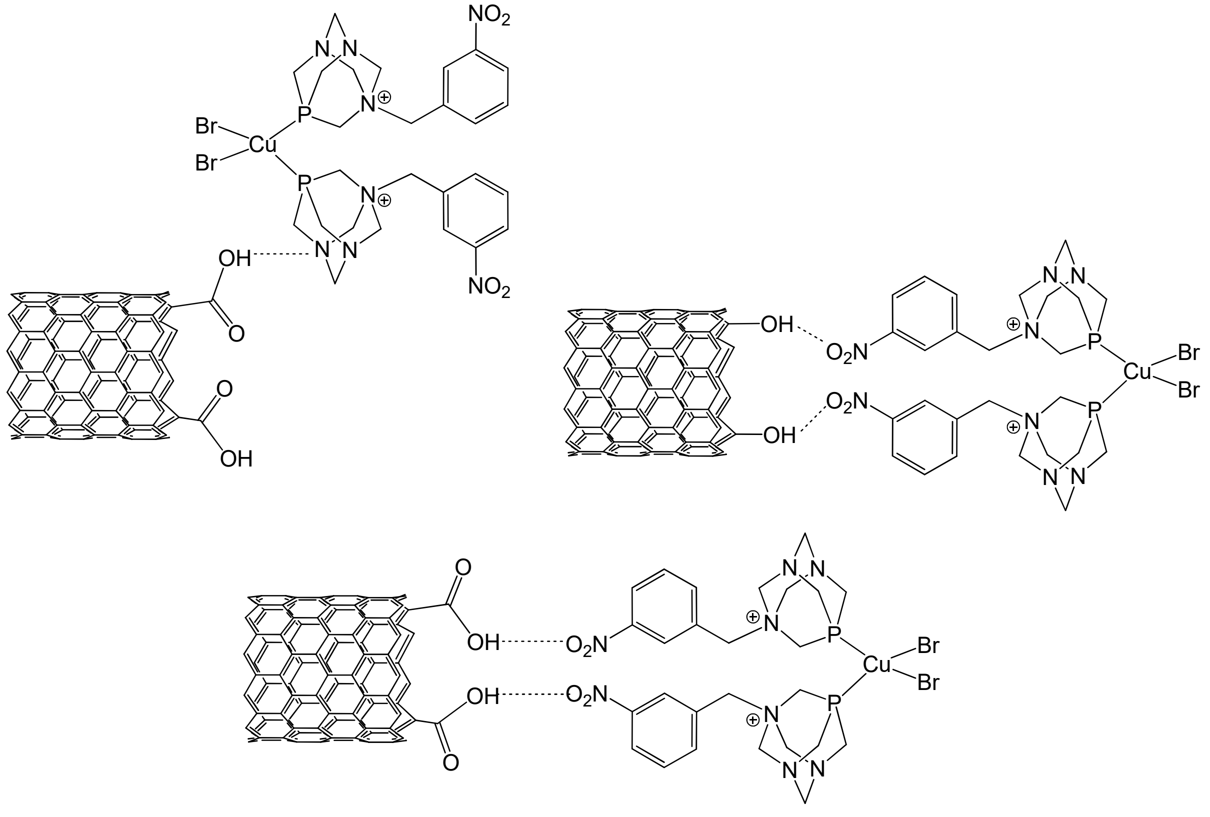

Figure S71. Via hydrogen bonding from a carboxylic or phenolic group.

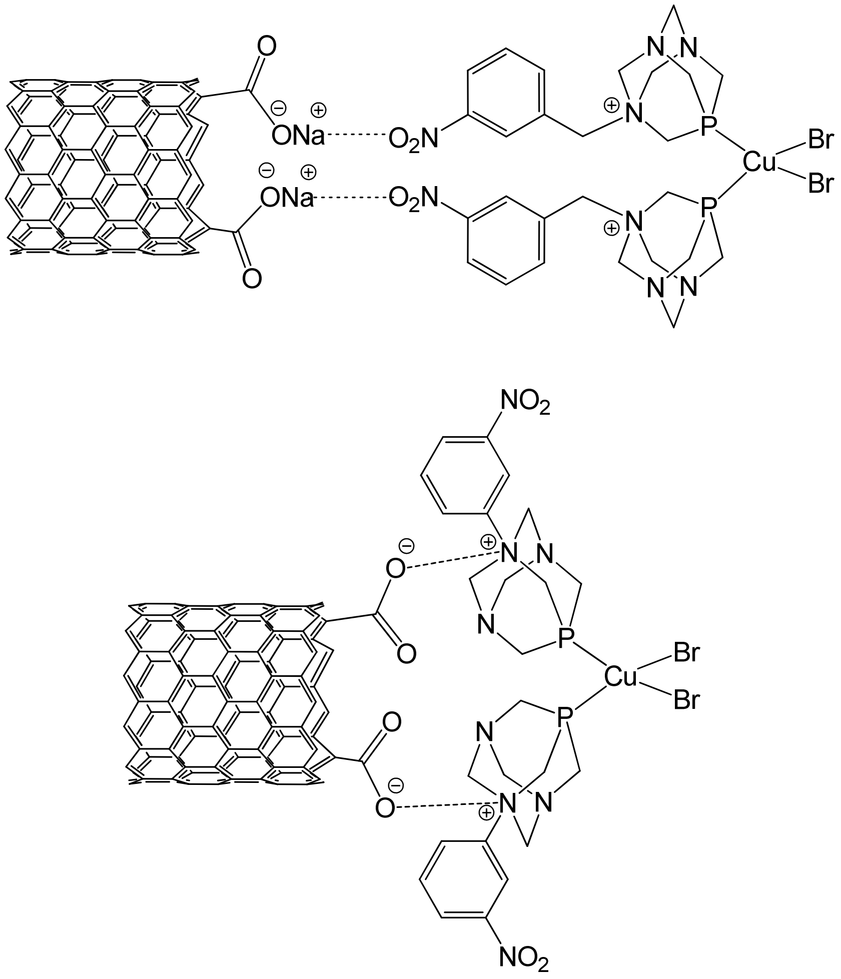

Figure S72. Via ionic interactions.

4. FTIR Spectra of CNT-ox-Na Materials (2000–400 cm^{-1})

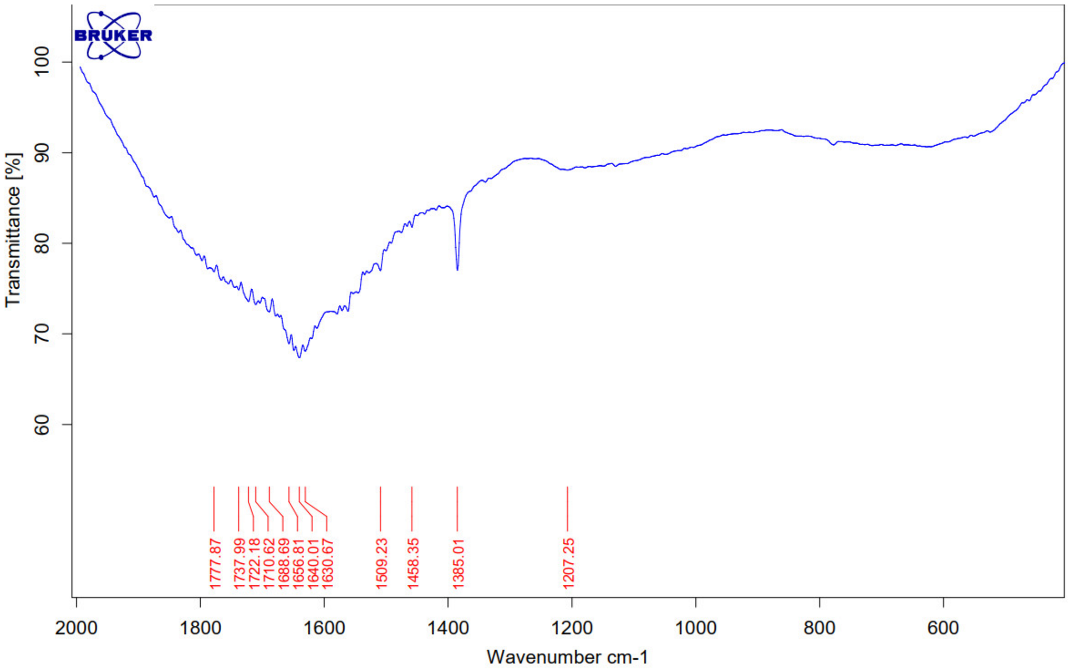

Figure S73. FTIR spectrum of CNT-ox-Na support.

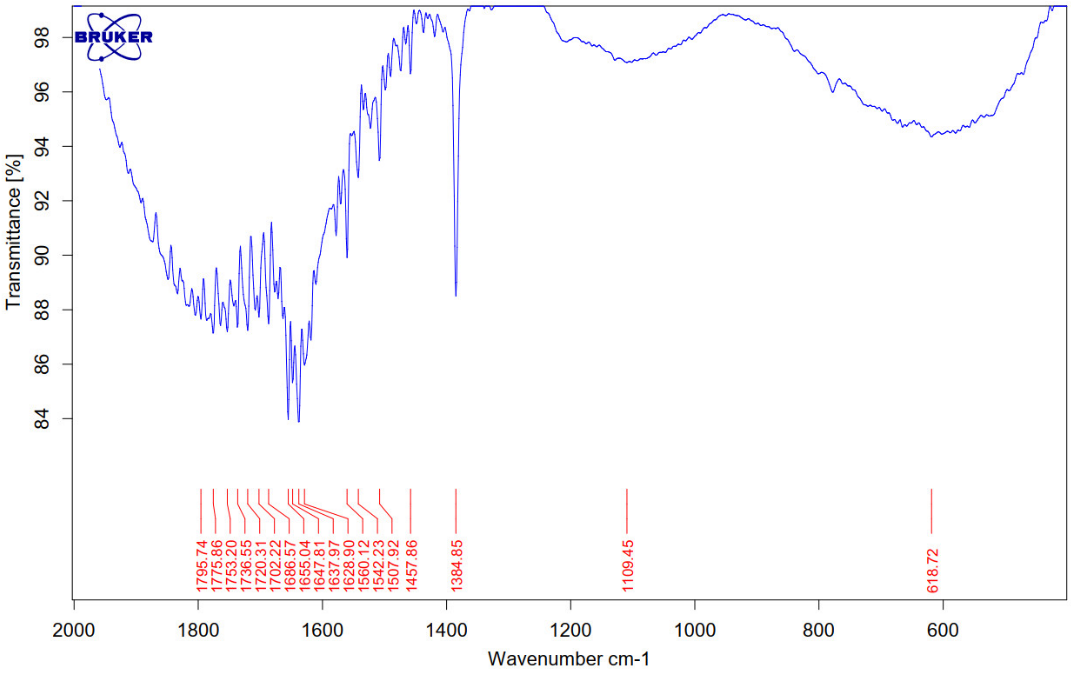

Figure S74. FTIR spectrum of **5a**_CNT-ox-Na.

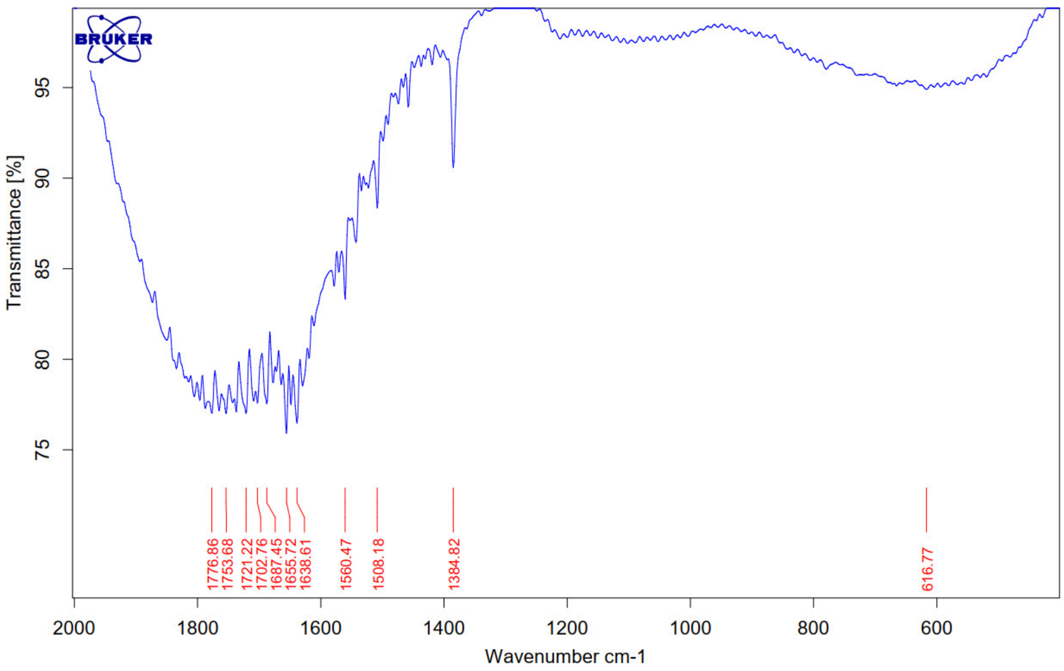

Figure S75. FTIR spectrum of **5b**_CNT-ox-Na.

5. NMR Spectra of Triazole Products

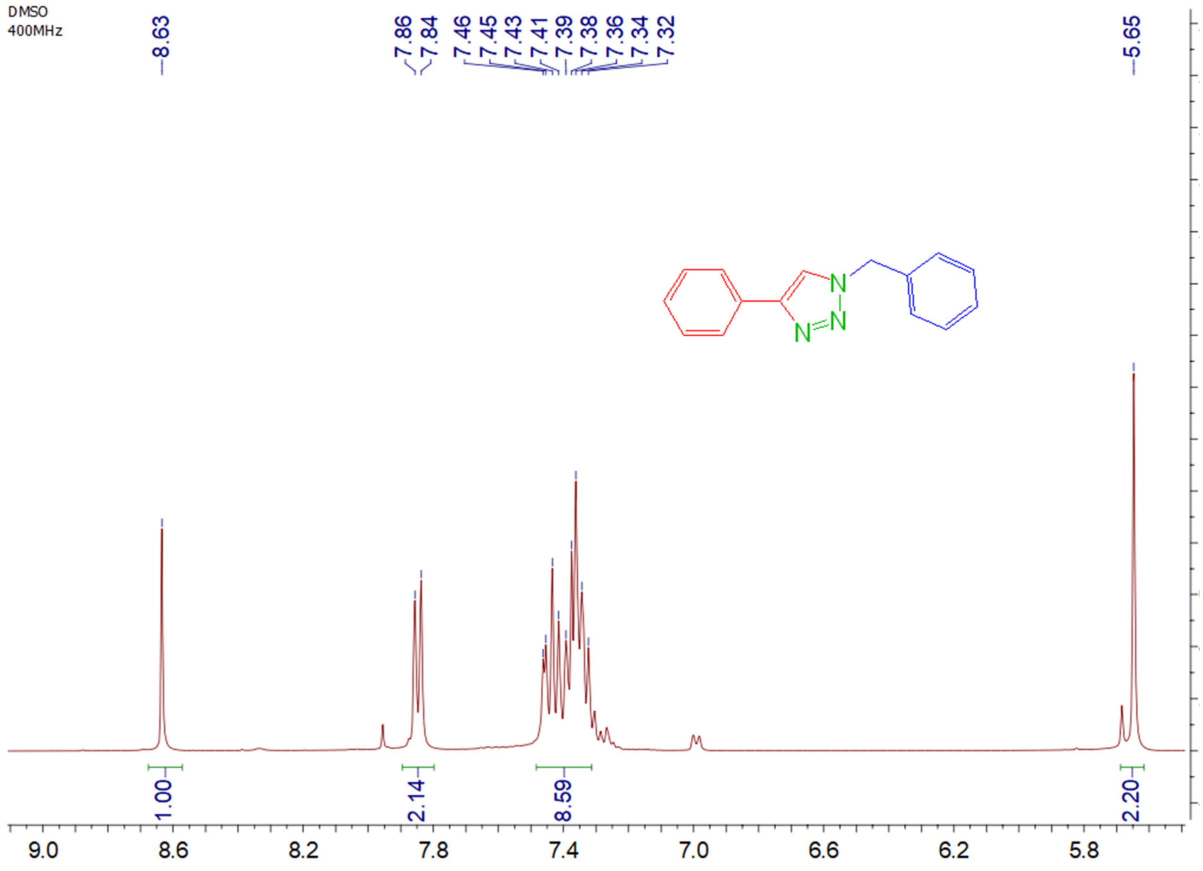

Figure S76. ^{1}H NMR spectrum of 1-benzyl-4-phenyl-1H-1,2,3-triazole.

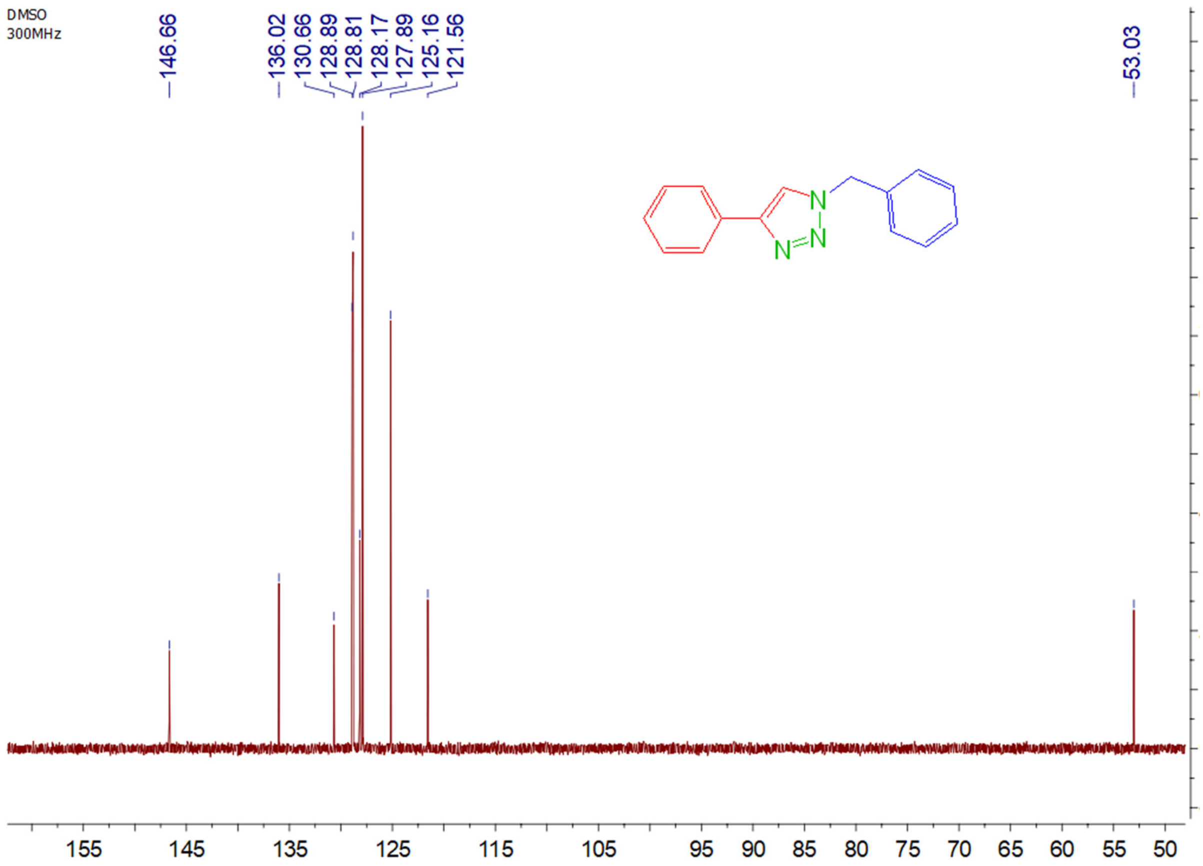

Figure S77. ^{13}C NMR spectrum of 1-benzyl-4-phenyl-1H-1,2,3-triazole.

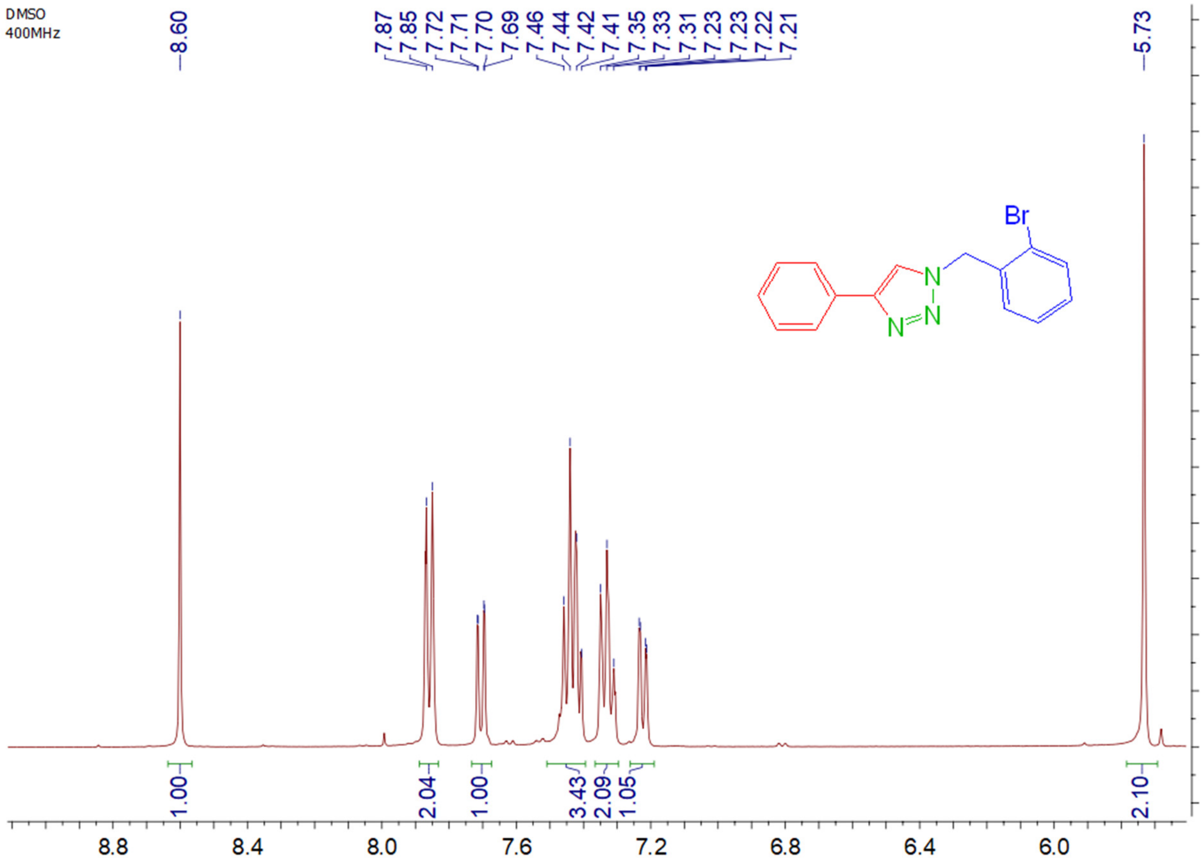

Figure S78. ^{1}H NMR spectrum of 1-(2-bromobenzyl)-4-phenyl-1H-1,2,3-triazole.

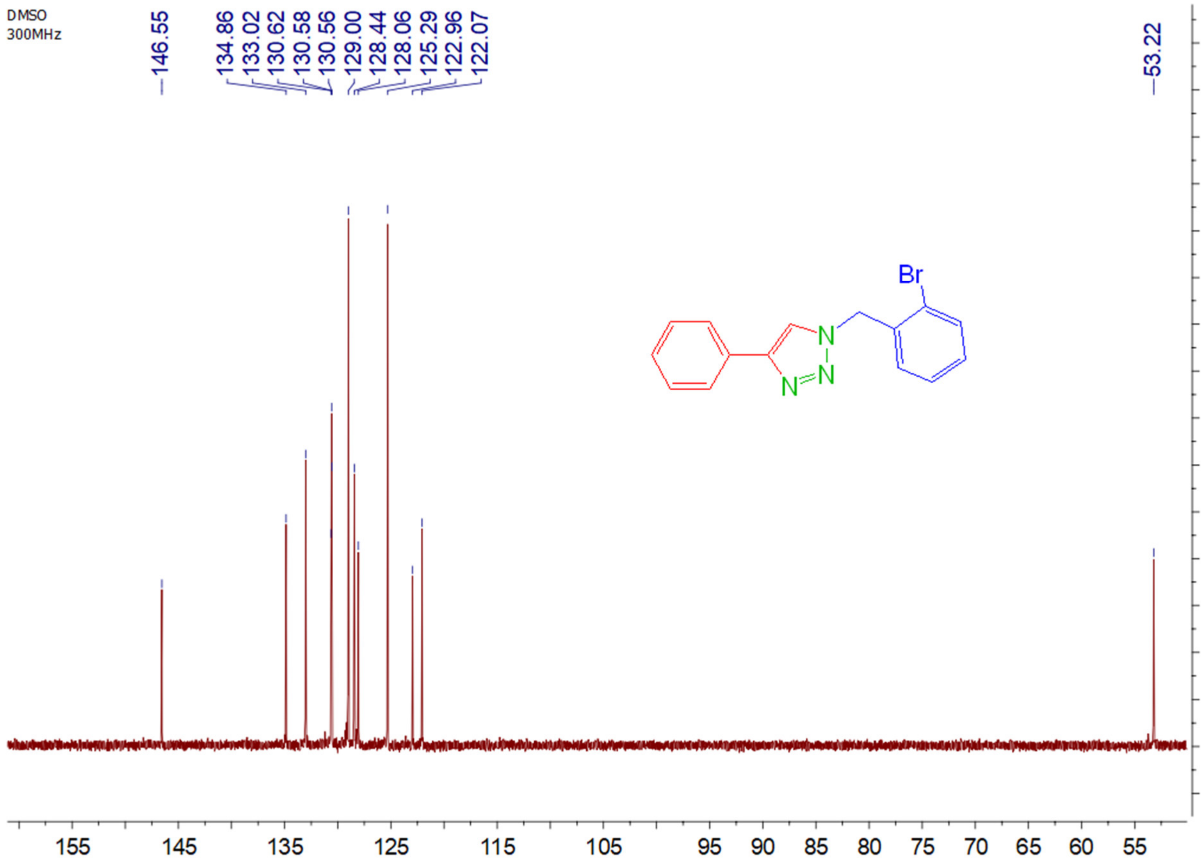

Figure S79. ^{13}C NMR spectrum of 1-(2-bromobenzyl)-4-phenyl-1H-1,2,3-triazole.

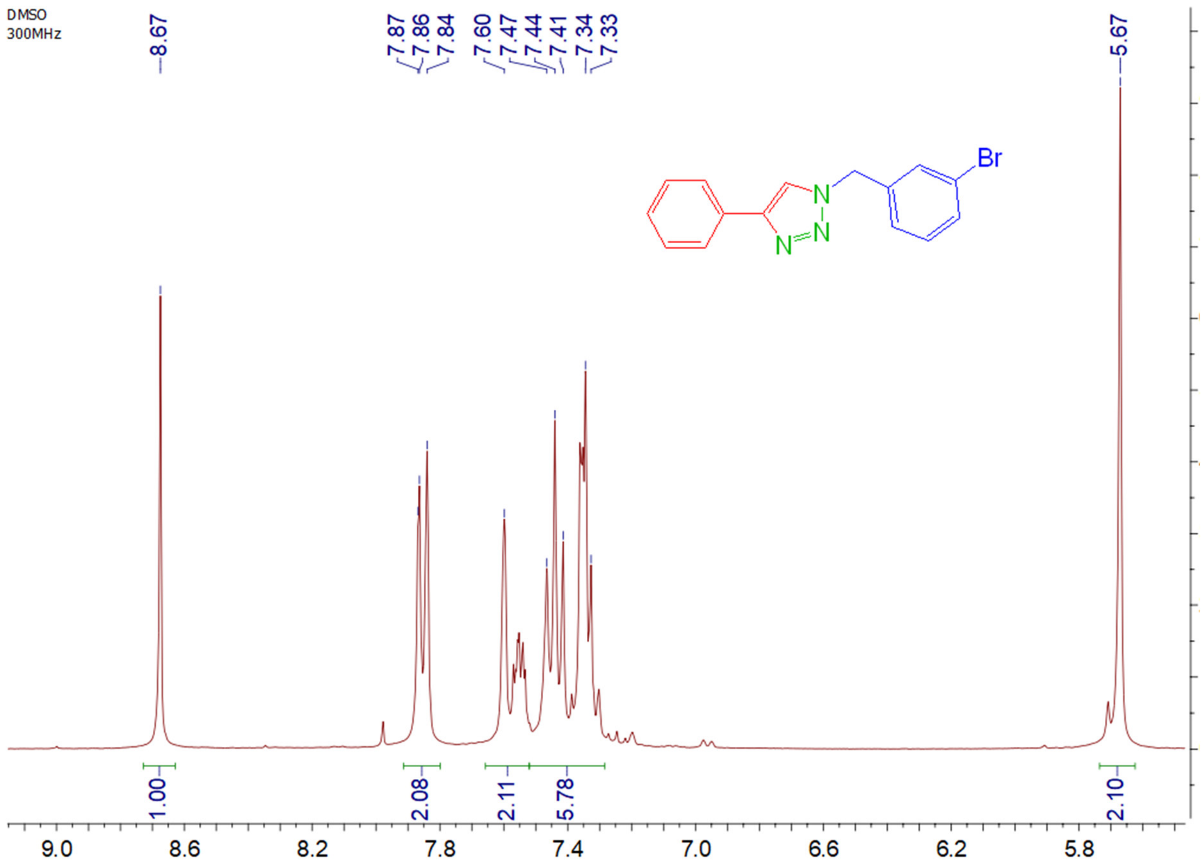

Figure S80. ^{1}H NMR spectrum of 1-(3-bromobenzyl)-4-phenyl-1H-1,2,3-triazole.

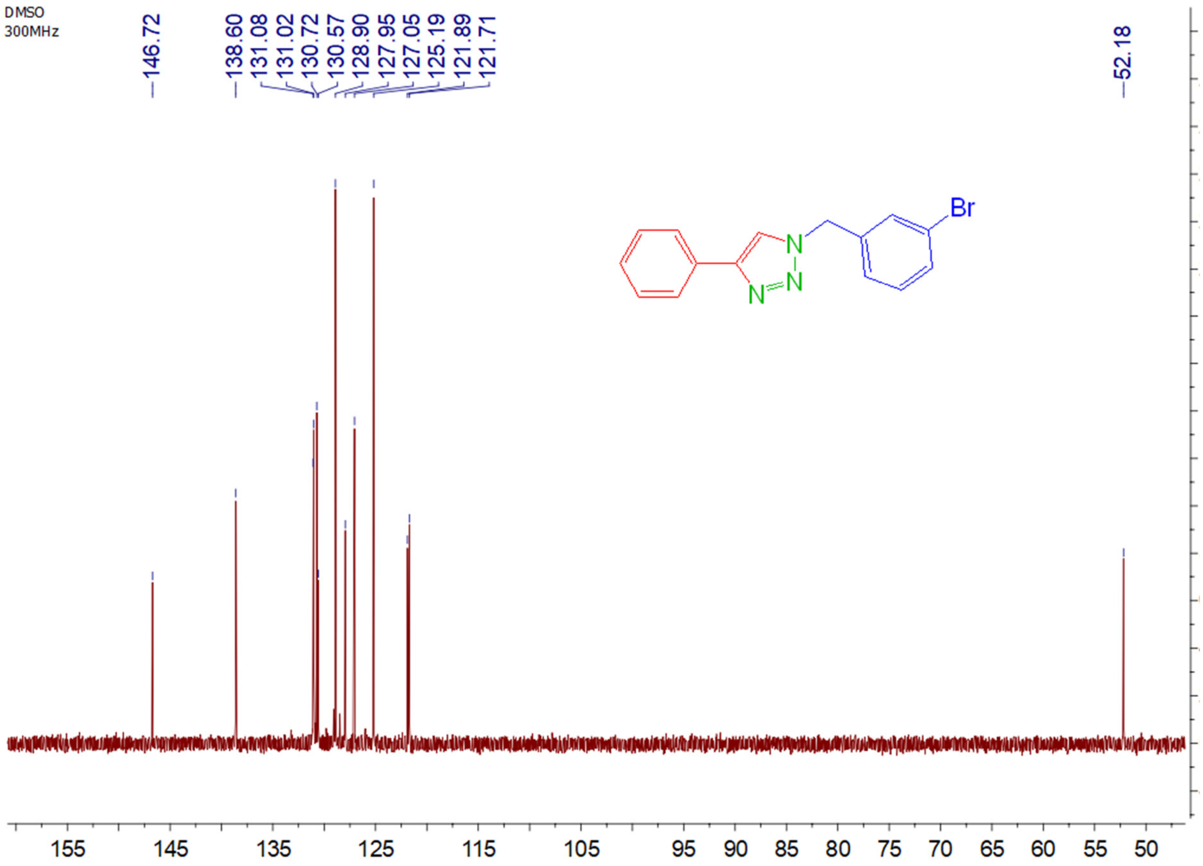

Figure S81. ^{13}C NMR spectrum of 1-(3-bromobenzyl)-4-phenyl-1H-1,2,3-triazole.

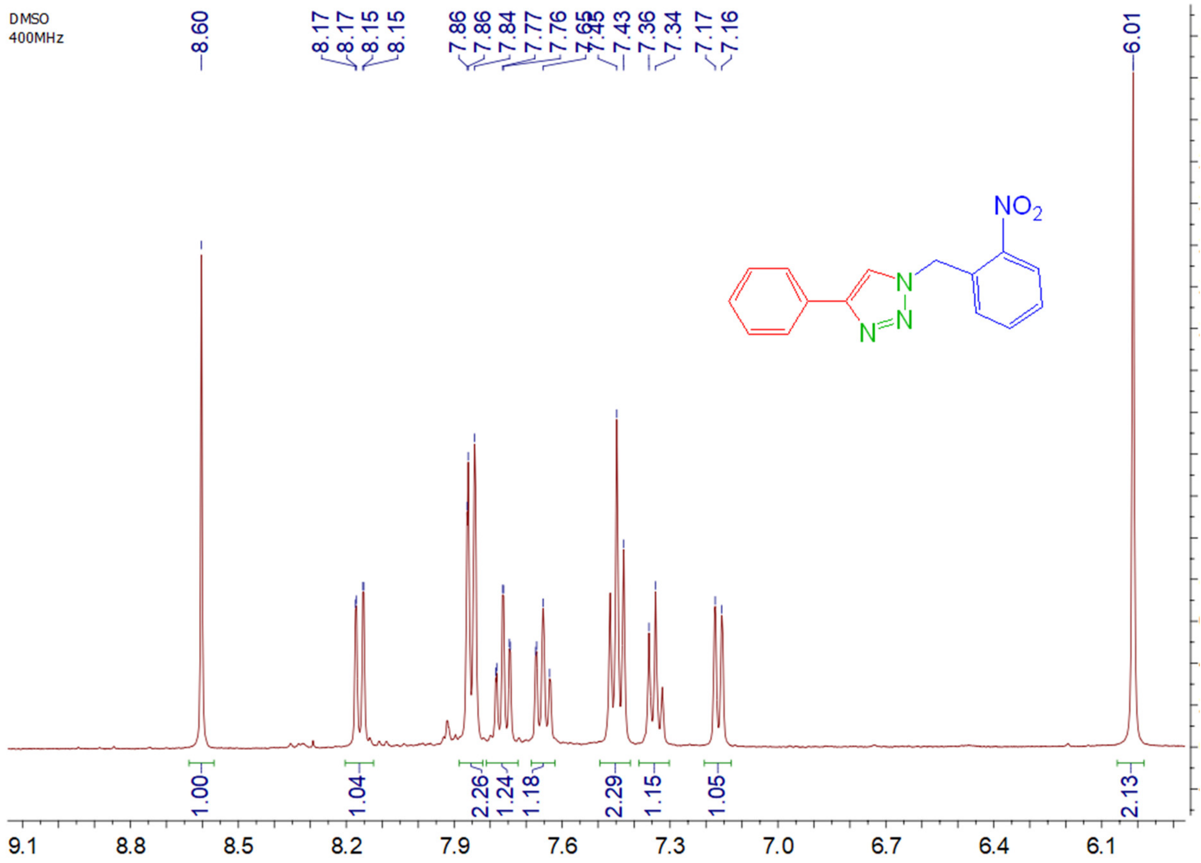

Figure S82. ^{1}H NMR spectrum of 1-(2-nitrobenzyl)-4-phenyl-1H-1,2,3-triazole.

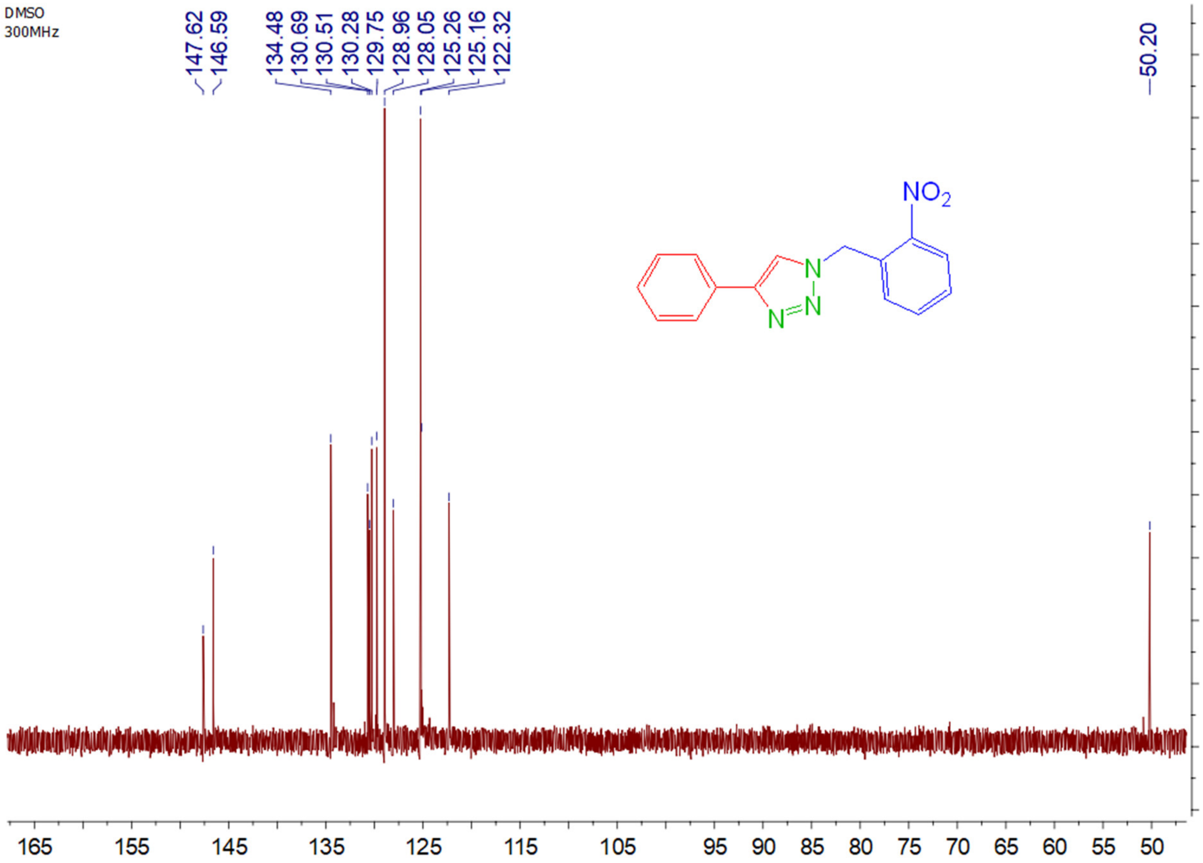

Figure S83. ^{13}C NMR spectrum of 1-(2-nitrobenzyl)-4-phenyl-1H-1,2,3-triazole.

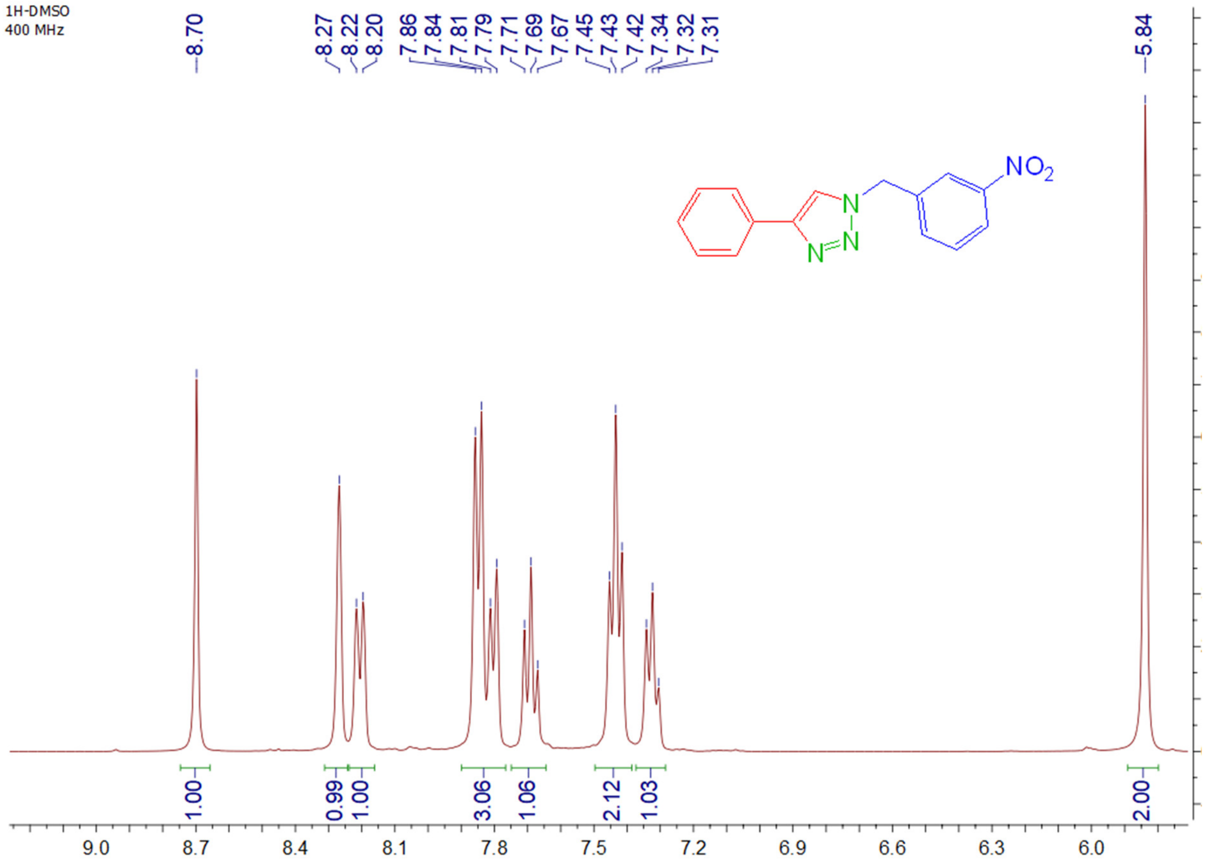

Figure S84. ^{1}H NMR spectrum of 1-(3-nitrobenzyl)-4-phenyl-1H-1,2,3-triazole.

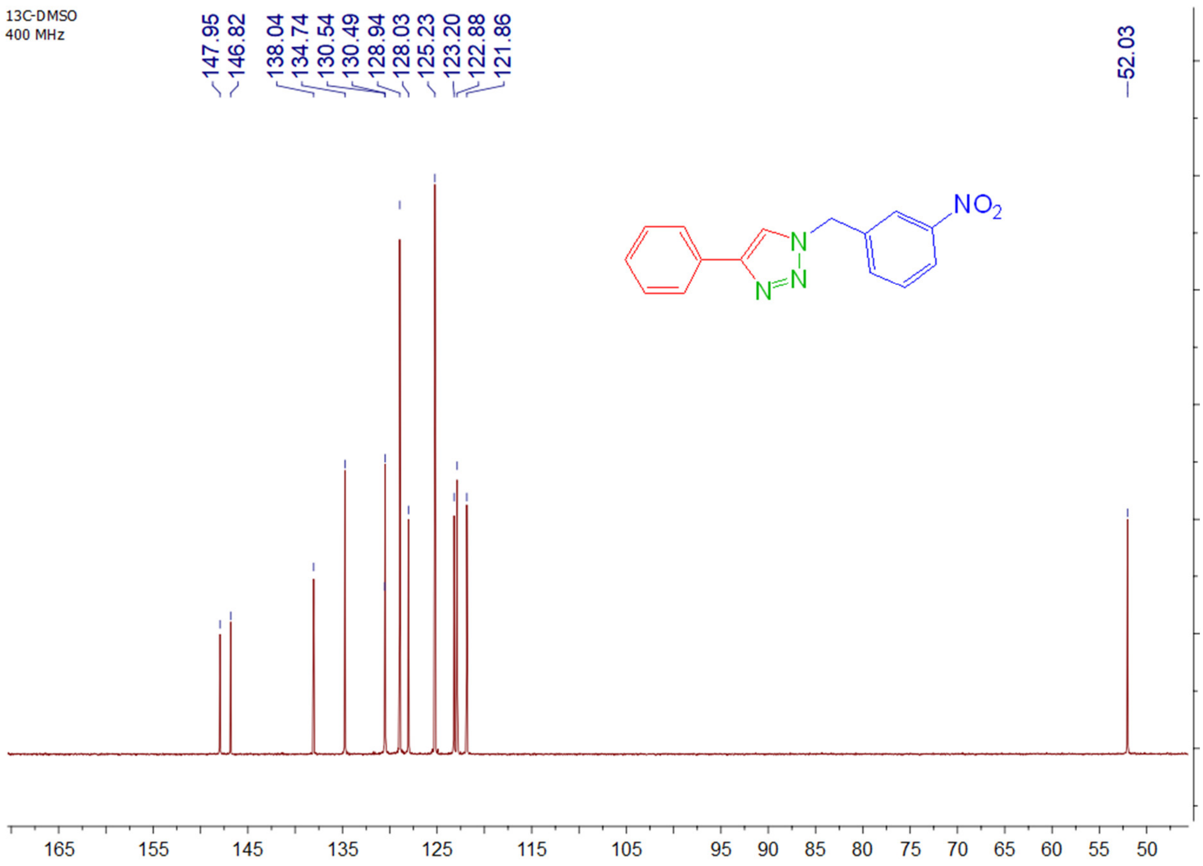

Figure S85. ^{13}C NMR spectrum of 1-(3-nitrobenzyl)-4-phenyl-1H-1,2,3-triazole.

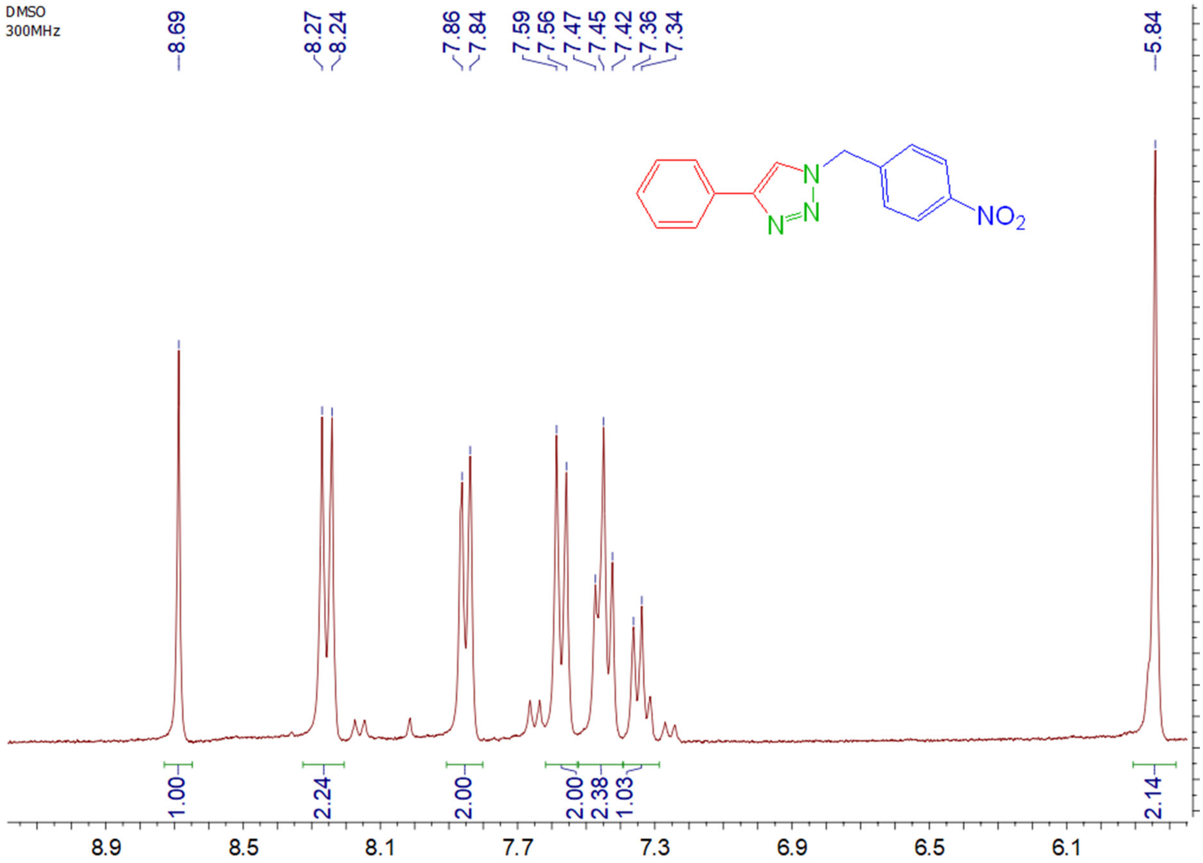

Figure S86. ^{1}H NMR spectrum of 1-(4-nitrobenzyl)-4-phenyl-1H-1,2,3-triazole.

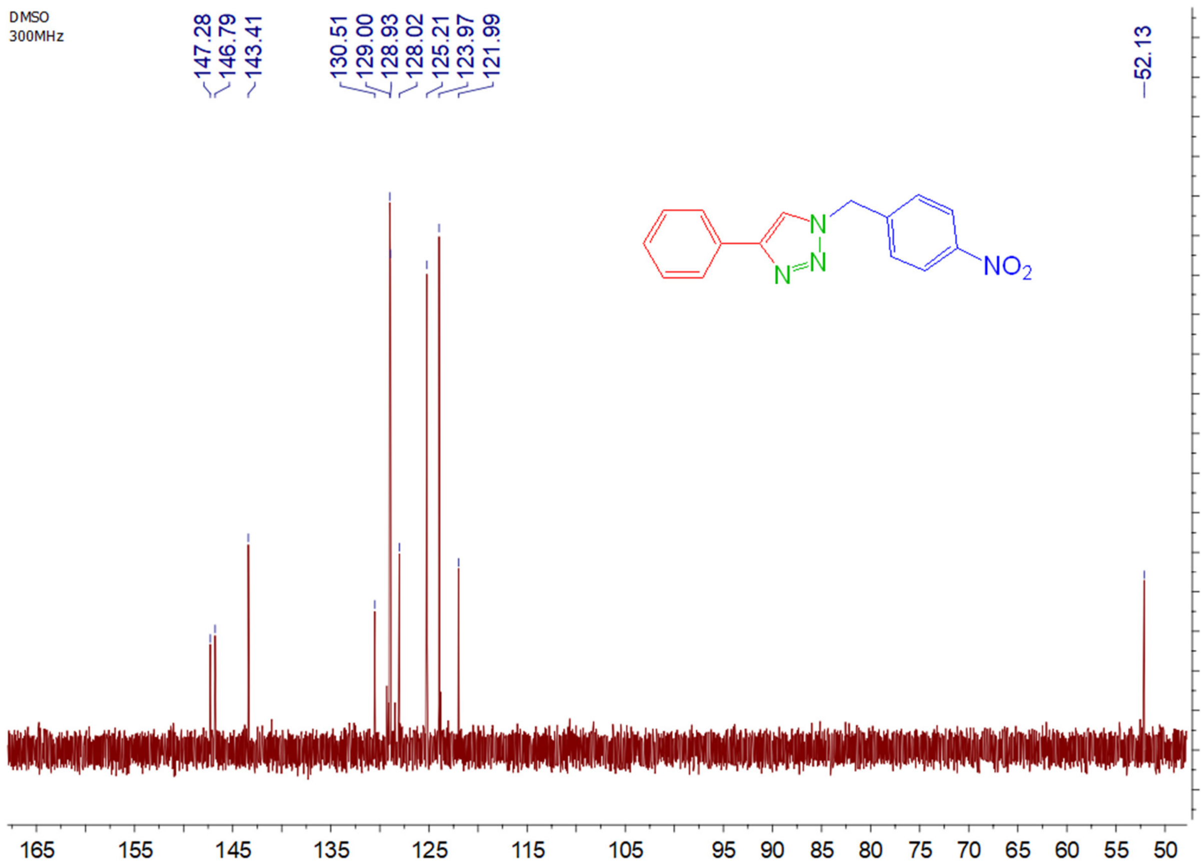

Figure S87. ^{13}C NMR spectrum of 1-(4-nitrobenzyl)-4-phenyl-1H-1,2,3-triazole.

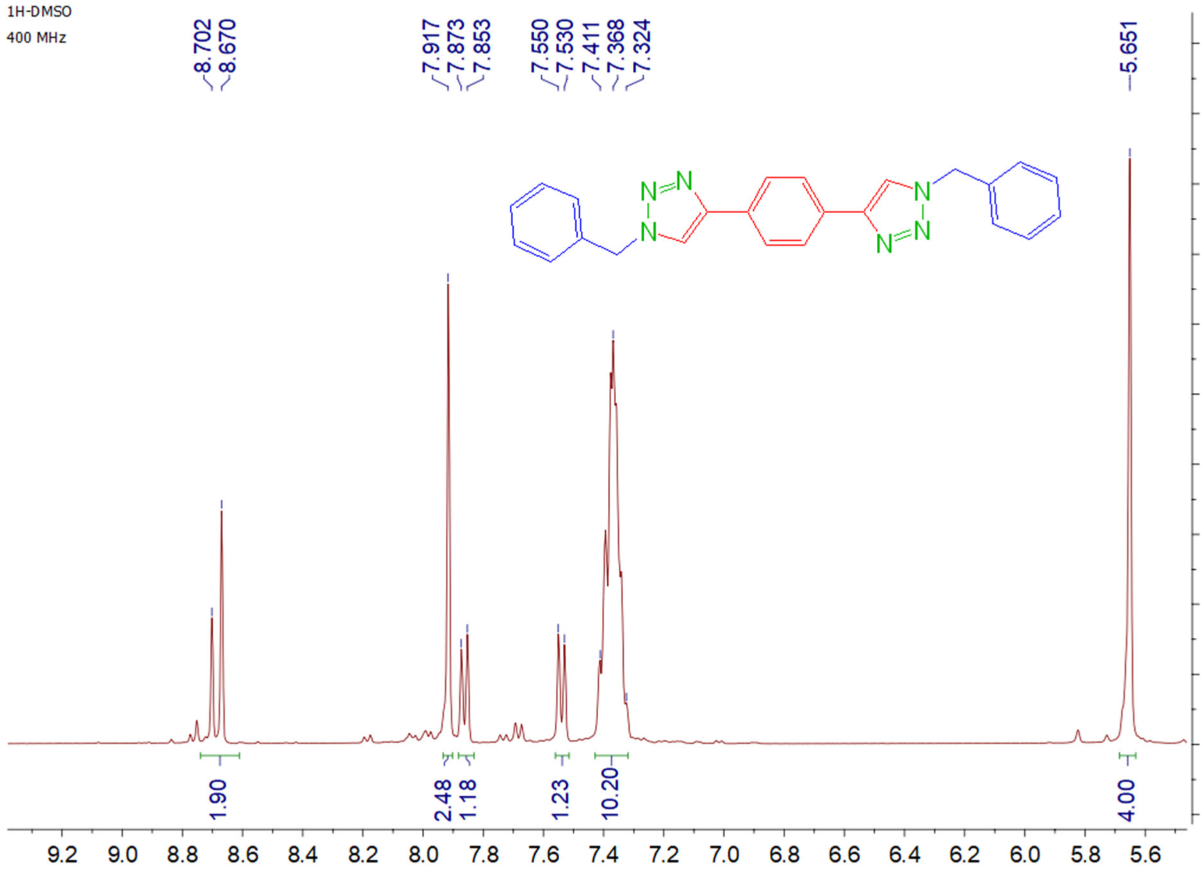

Figure S88. ^{1}H NMR spectrum of 1,4-bis(1-benzyl-1H-1,2,3-triazol-4-yl)benzene.

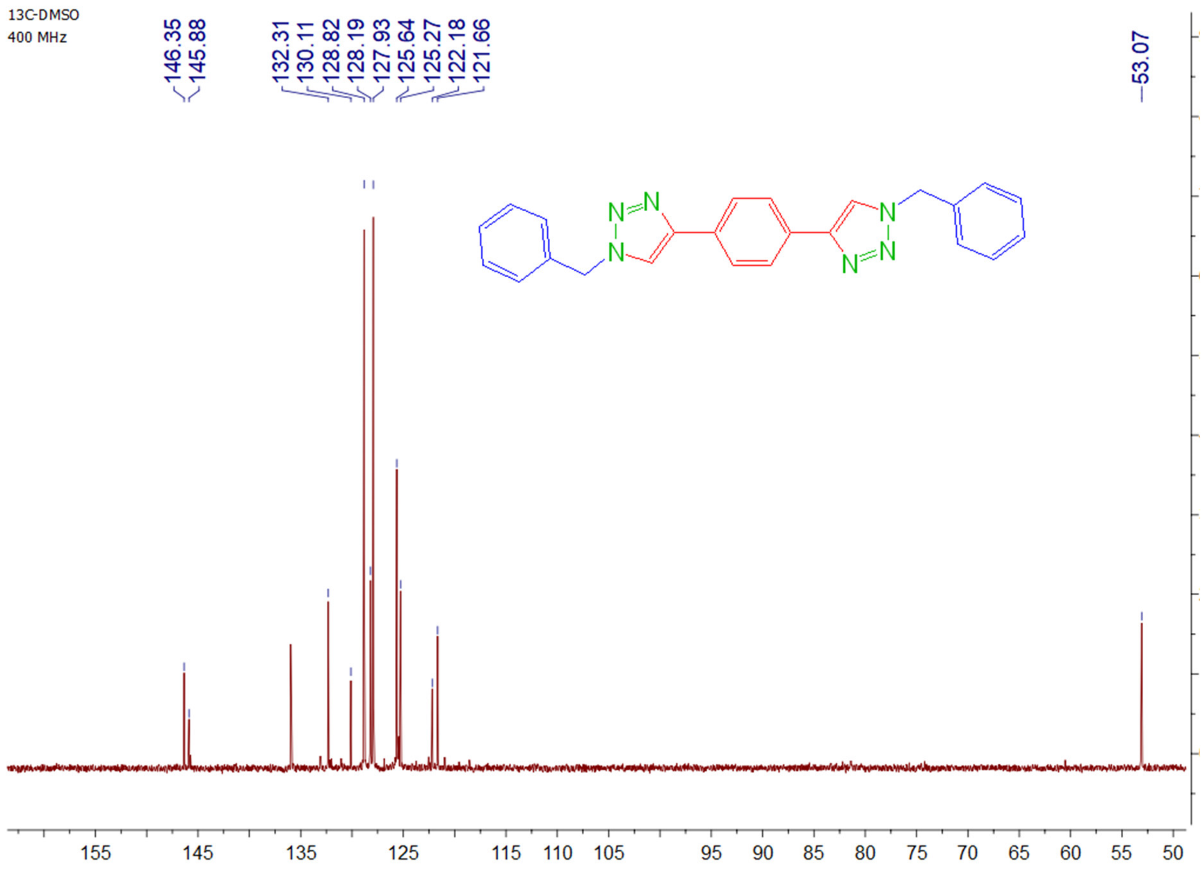

Figure S89. ^{13}C NMR spectrum of 1,4-bis(1-benzyl-1H-1,2,3-triazol-4-yl)benzene.

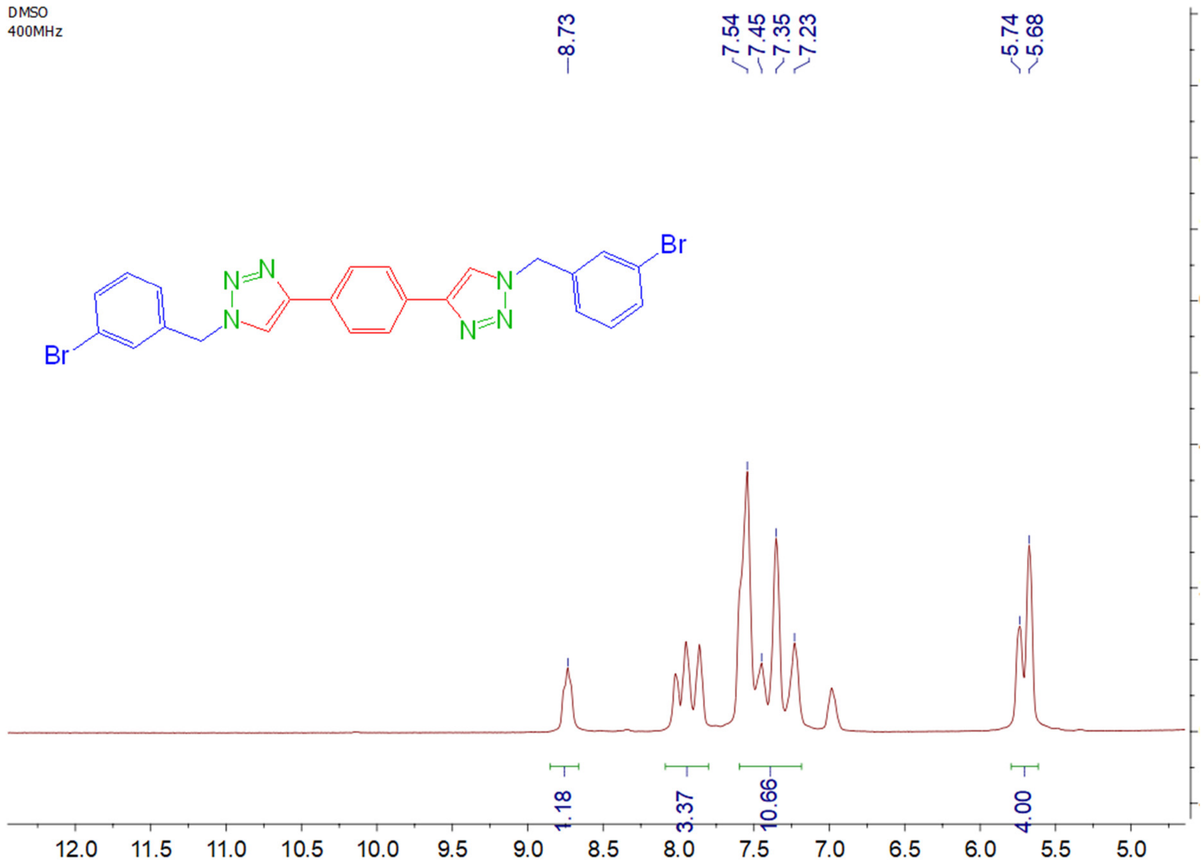

Figure S90. ^{1}H NMR spectrum of 1,4-bis(1-(3-bromobenzyl)-1H-1,2,3-triazol-4-yl)benzene.

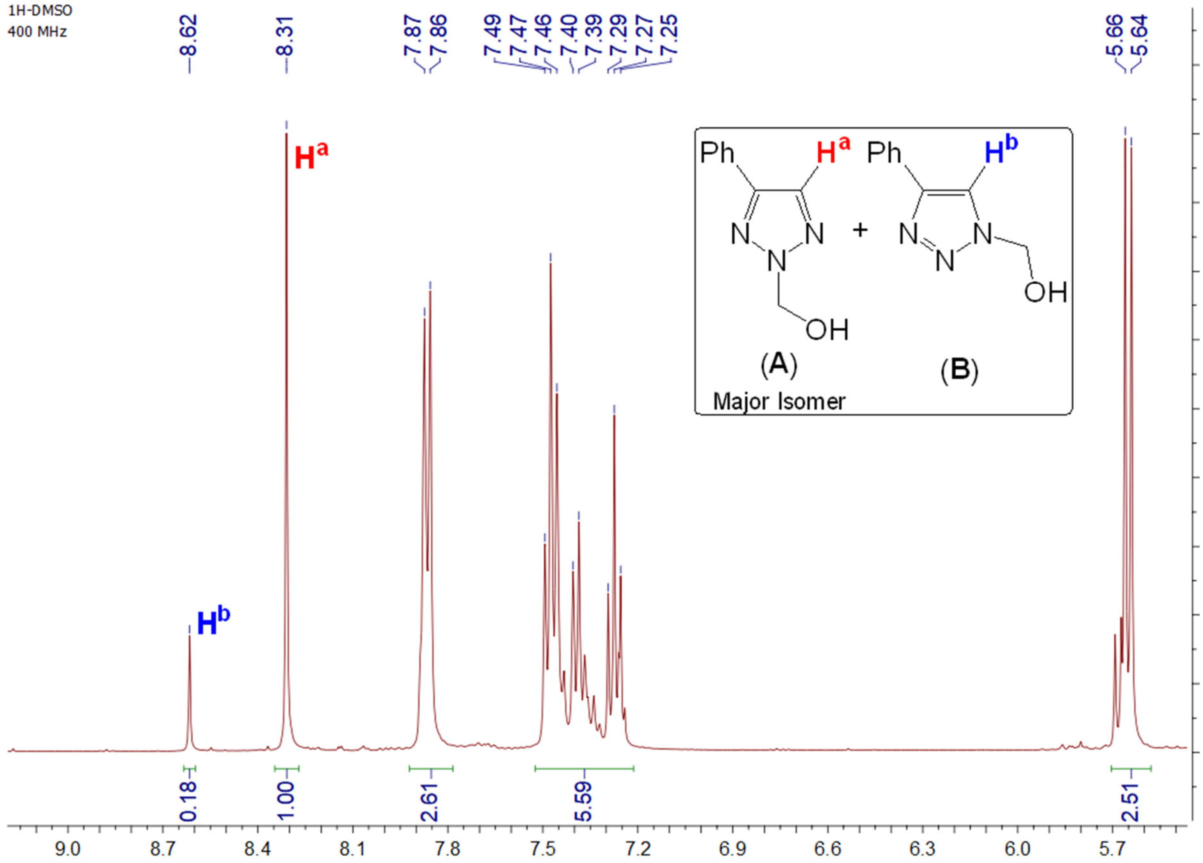

Figure 91. ^{1}H-NMR spectrum (DMSO-d_6) of 1- and 2-hydroxymethyl-4-phenyl-1,2,3-triazole regioisomeric mixture obtained from the reaction described in Table 7, entry 3.

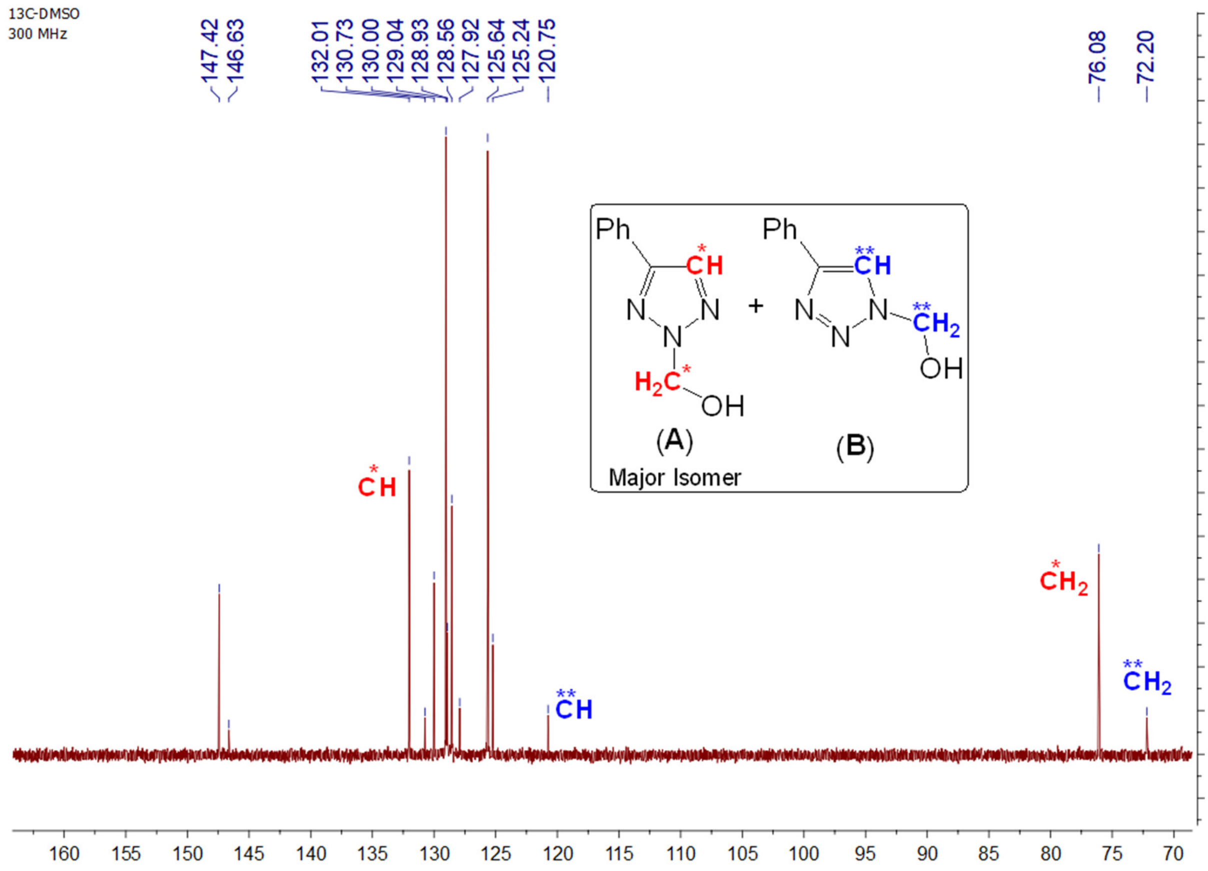

Figure 92. ^{13}C-NMR spectrum (DMSO-d_6) of 1- and 2-hydroxymethyl-4-phenyl-1,2,3-triazole regioisomeric mixture obtained from the reaction described in Table 7, entry 3.

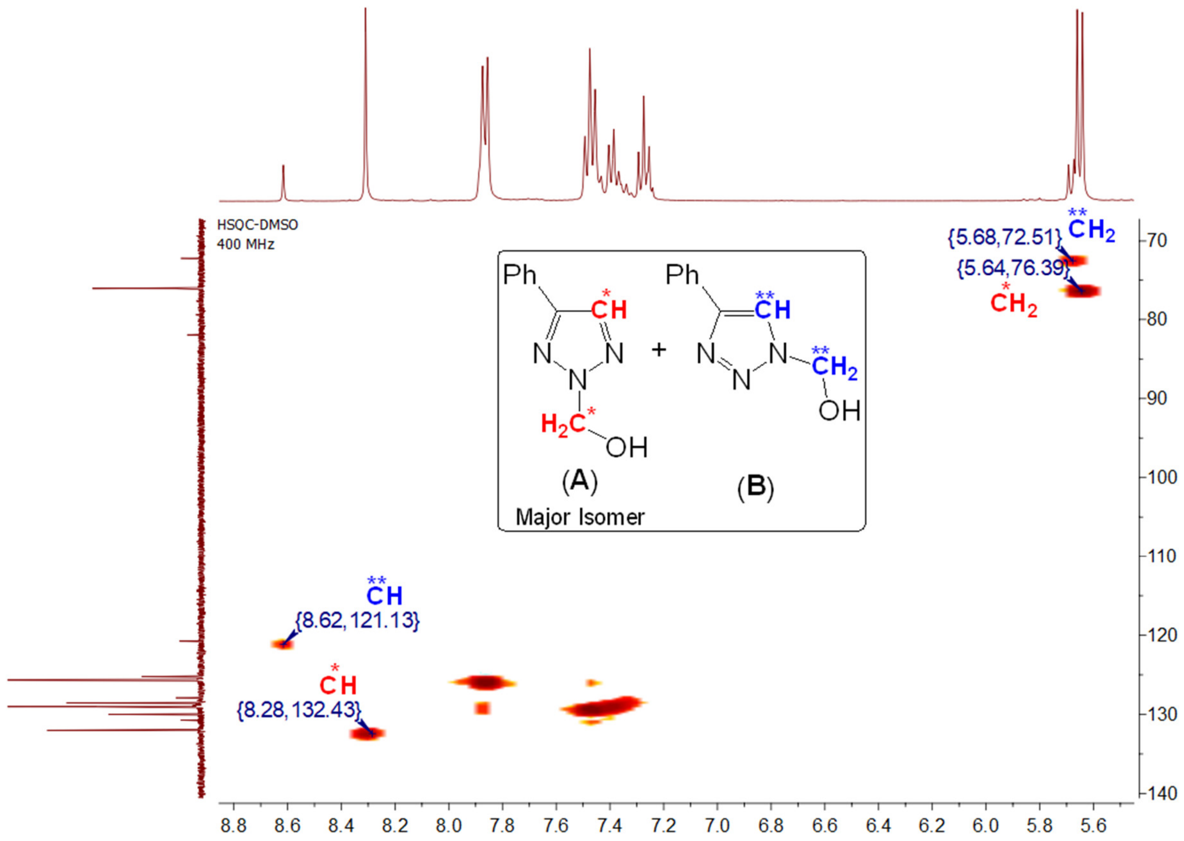

Figure 93. HSQC spectrum (DMSO-d_6) of 1- and 2-hydroxymethyl-4-phenyl-1,2,3-triazole regioisomeric mixture obtained from the reaction described in Table 7, entry 3.

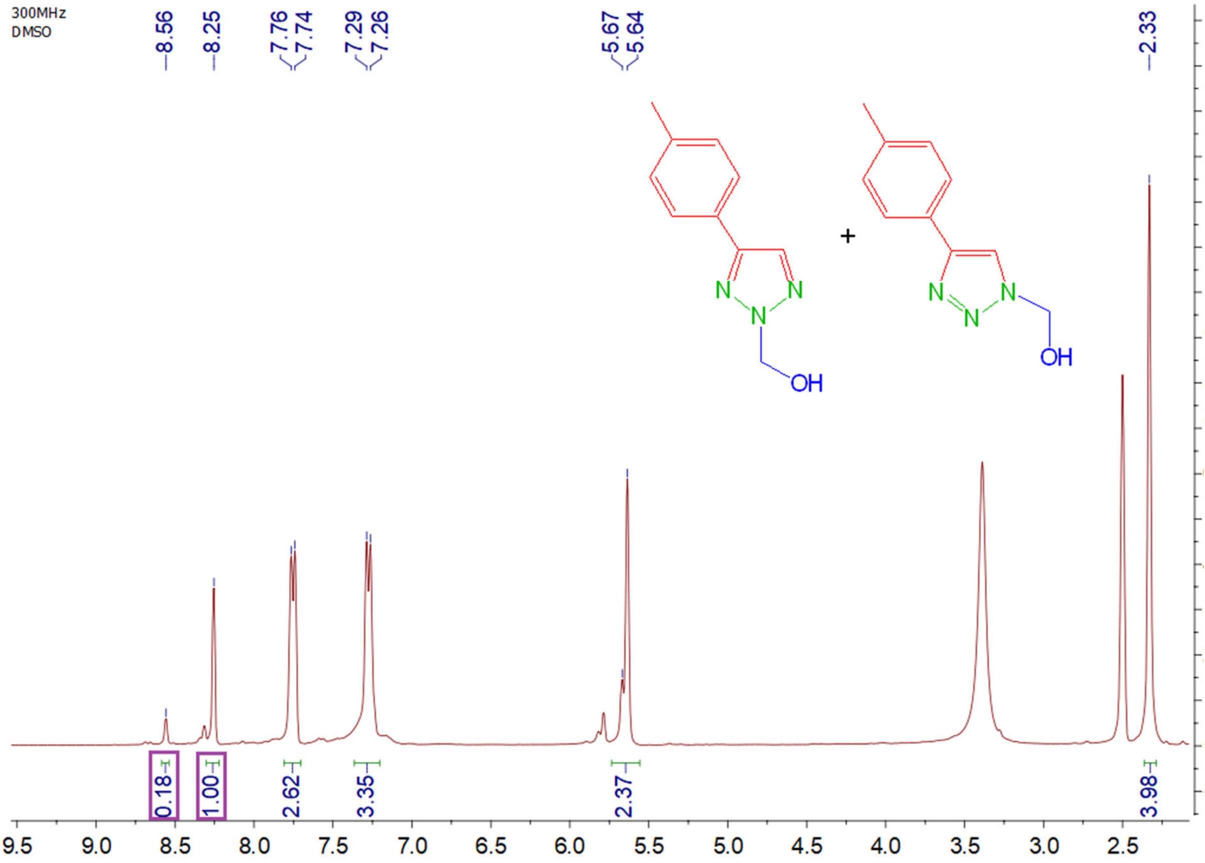

Figure S94. ^{1}H NMR spectrum of (4-(*p*-tolyl)-2*H*-1,2,3-triazol-2-yl)methanol (the major isomer).

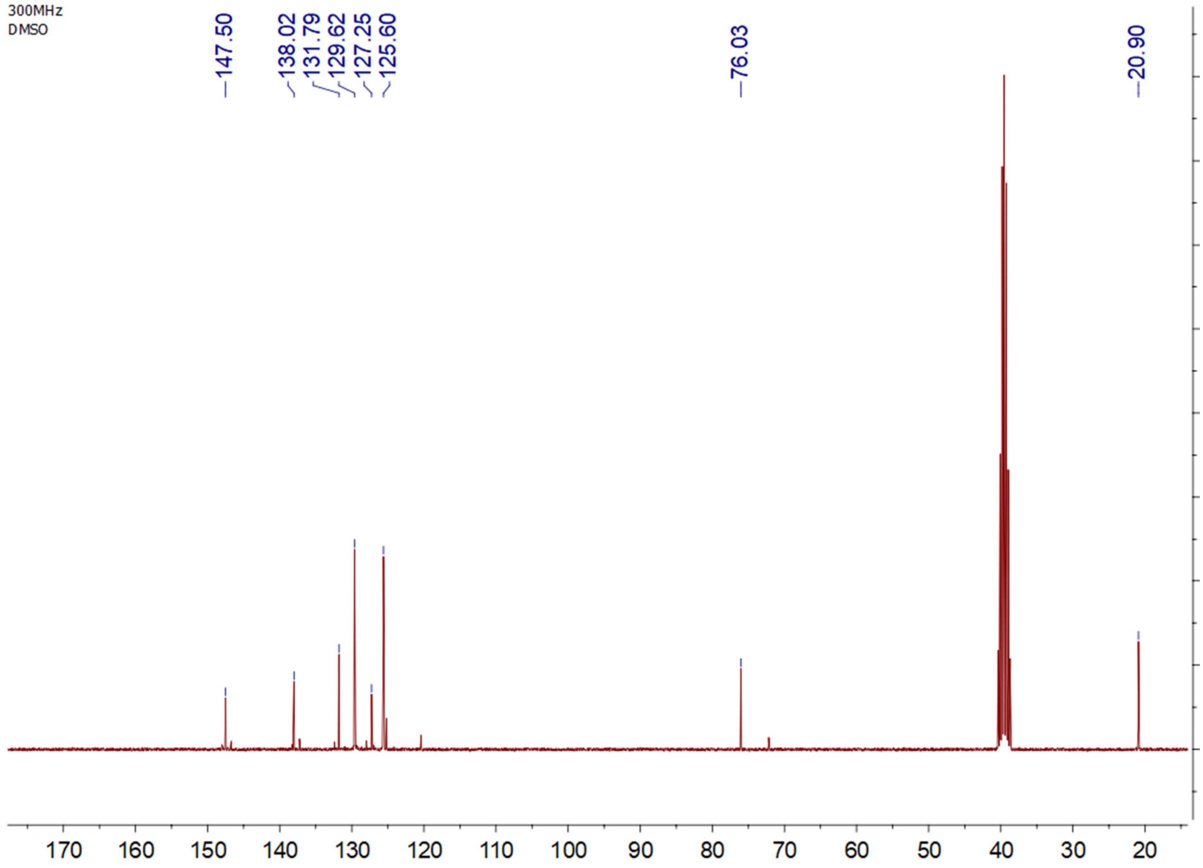

Figure S95. ^{13}C NMR spectrum of (4-(*p*-tolyl)-2*H*-1,2,3-triazol-2-yl)methanol (the major isomer).

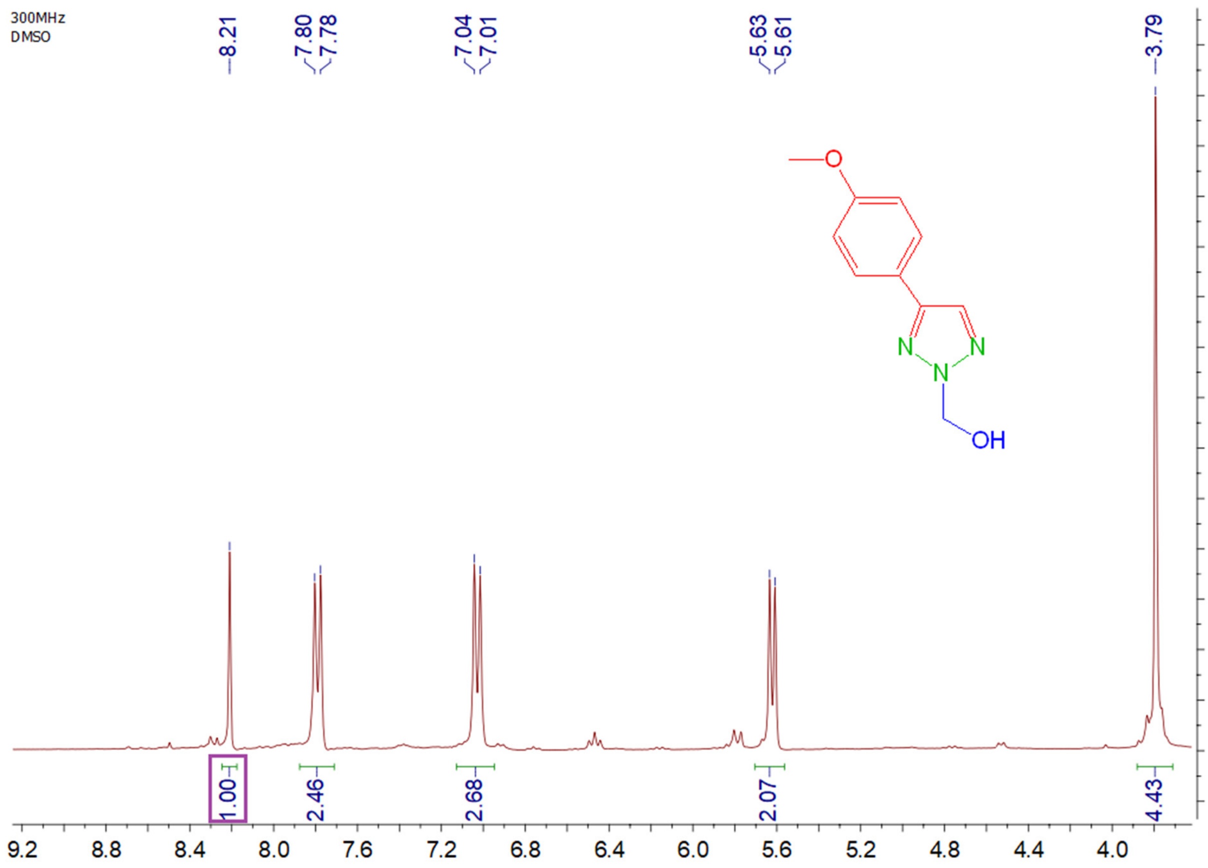

Figure S96. ^{1}H NMR spectrum of (4-(4-methoxyphenyl)- 2*H*-1,2,3-triazol-2-yl)methanol.

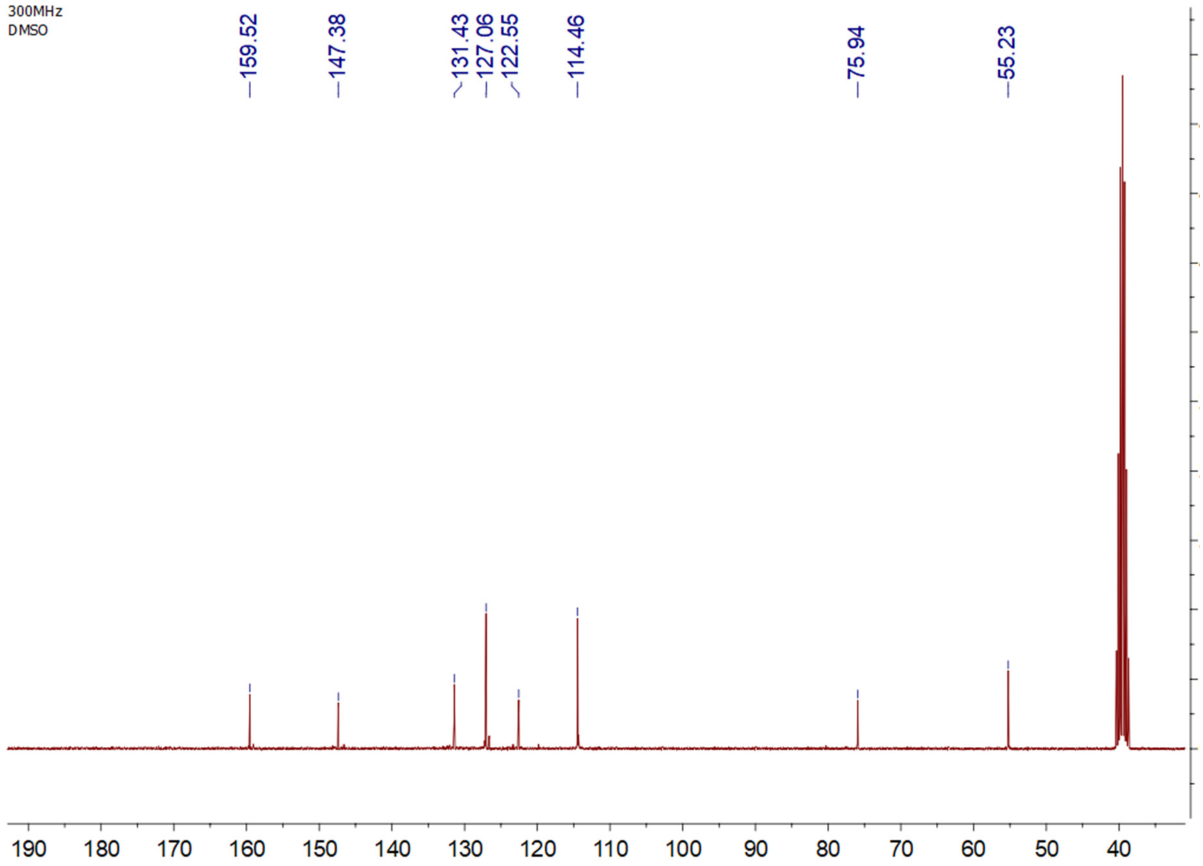

Figure S97. ^{13}C NMR spectrum of (4-(4-methoxyphenyl)-2*H*-1,2,3-triazol-2-yl)methanol.

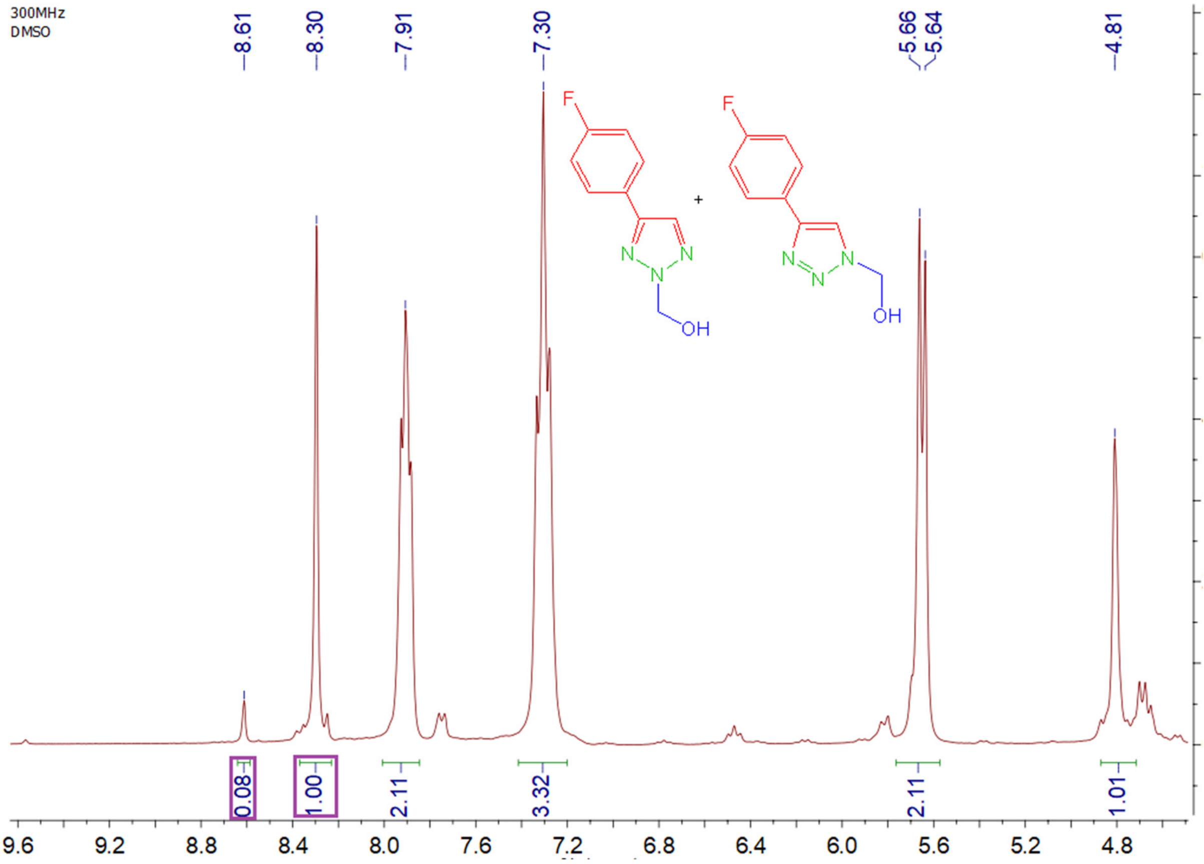

Figure S98. ^{1}H NMR spectrum of (4-(4-fluorophemyl)-2*H*-1,2,3-triazol-2-yl)methanol (the major isomer).

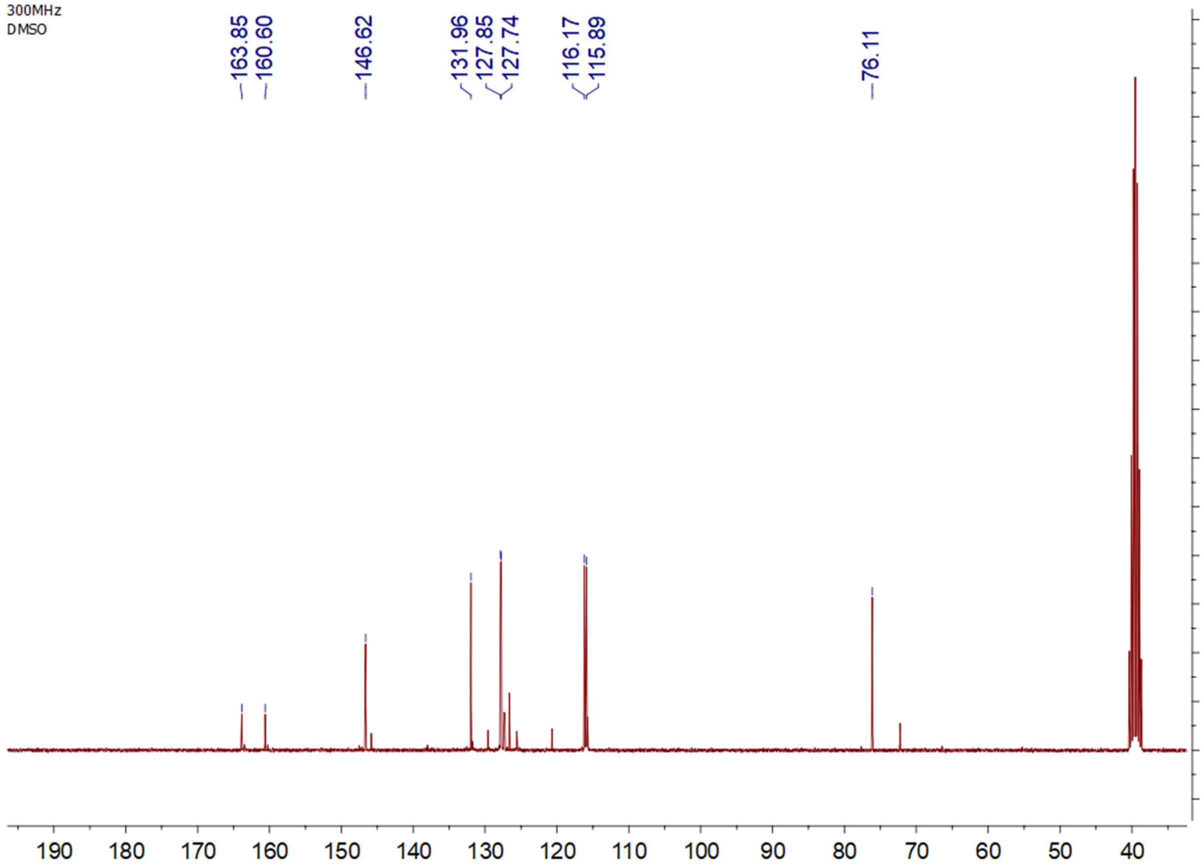

Figure S99. ^{13}C NMR spectrum of (4-(4-fluorophemyl)-2*H*-1,2,3-triazol-2-yl)methanol (the major isomer).

6. ESI-MS (Positive Mode) Spectra of Triazole Products

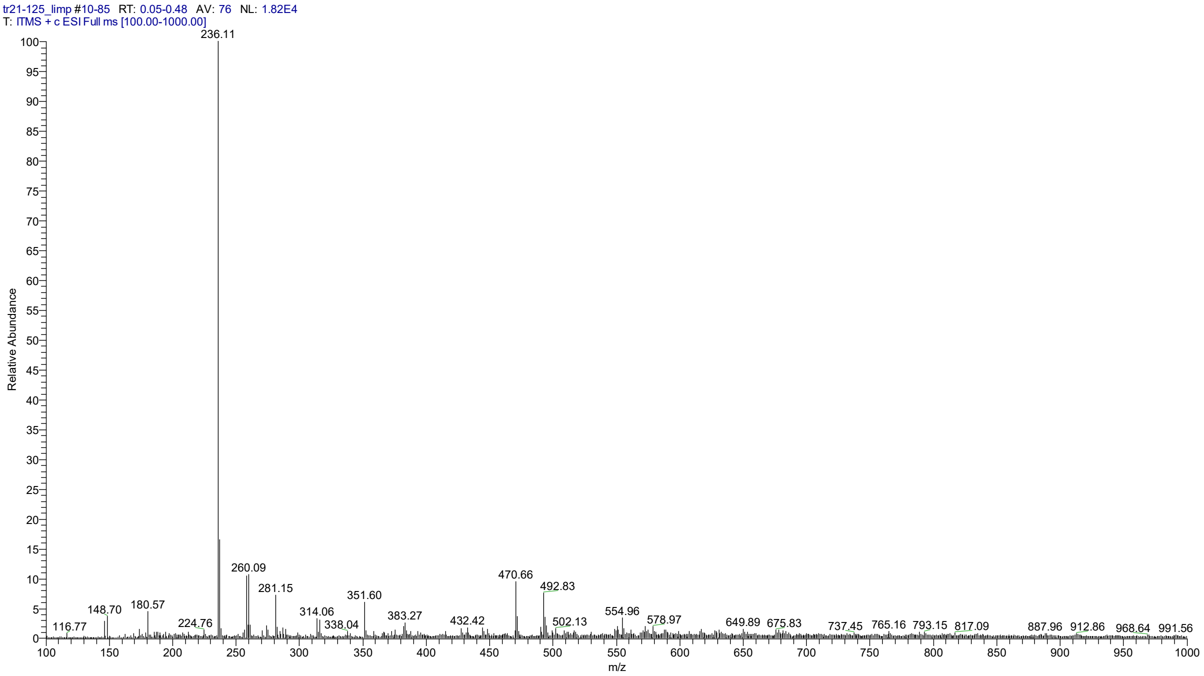

Figure S100. ESI-MS spectra of 1-benzyl-4-phenyl-1H-1,2,3-triazole.

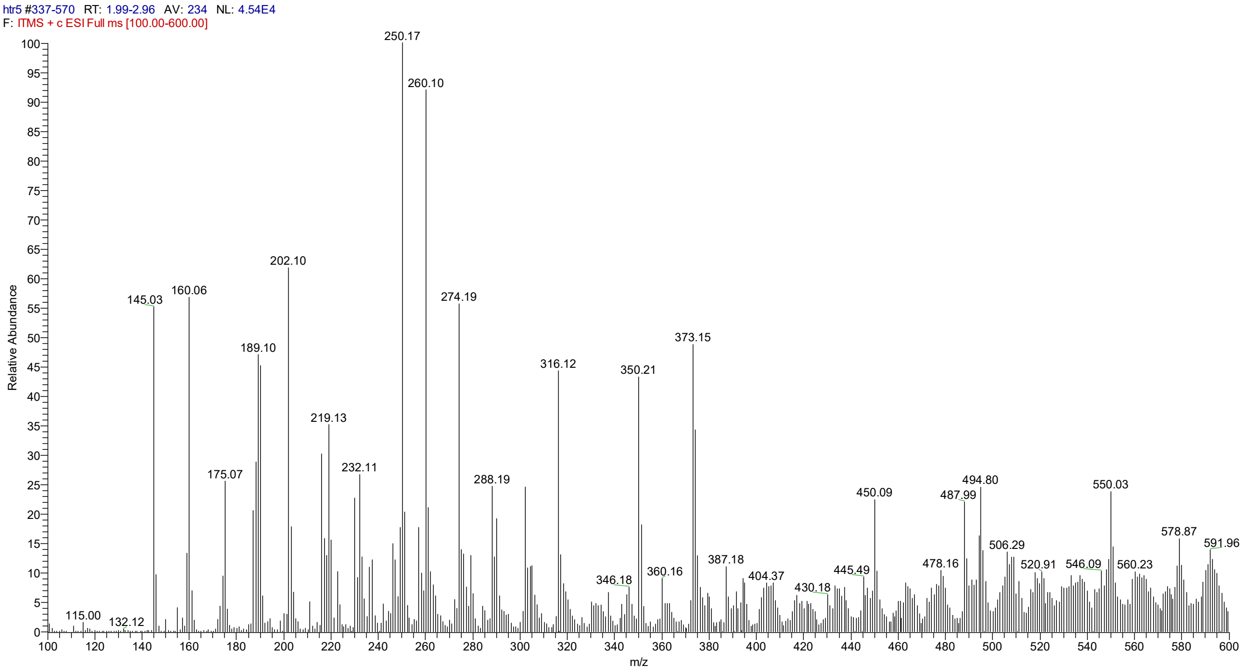

Figure S101. ESI-MS spectra of Hydroxymethyl-1,2,3-triazole.

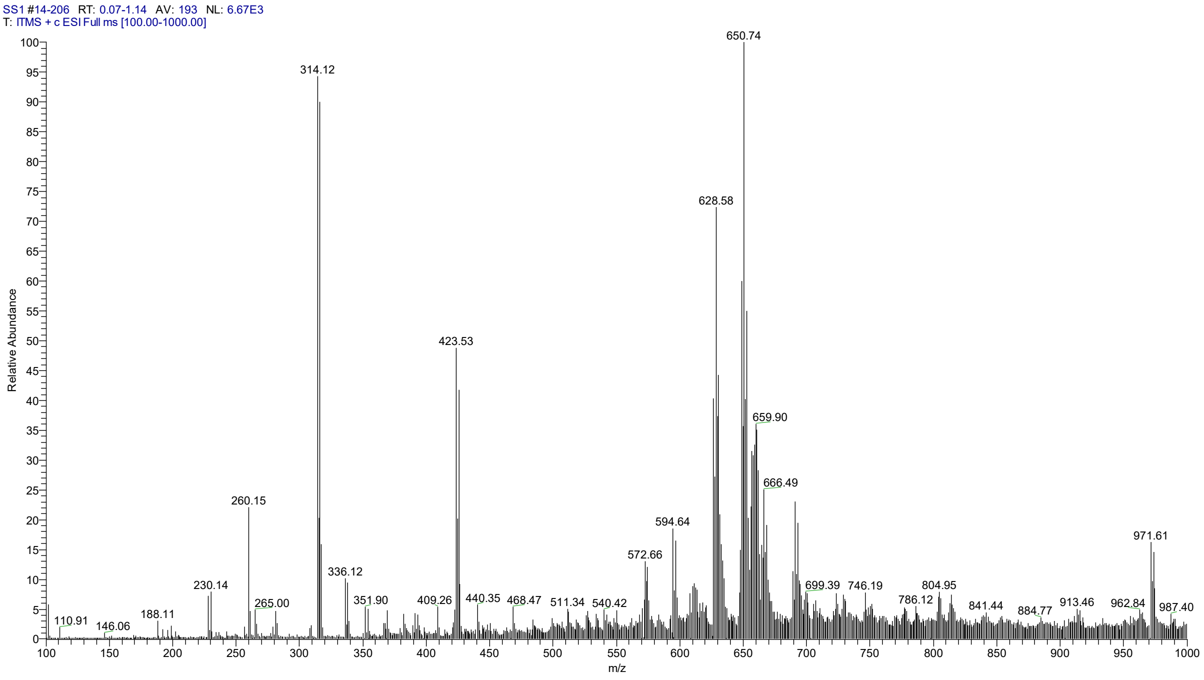

Figure S102. ESI-MS spectra of 1-(2-bromobenzyl)-4-phenyl-1H-1,2,3-triazole

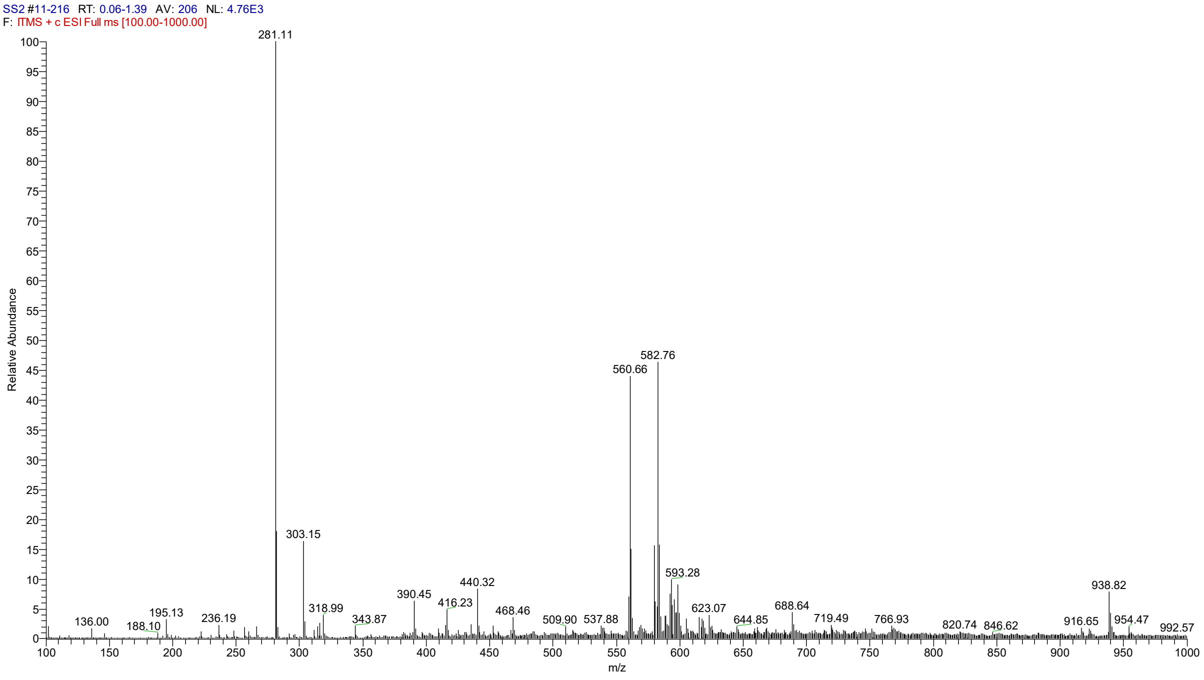

Figure S103. ESI-MS spectra of 1-(2-nitrobenzyl)-4-phenyl-1H-1,2,3-triazole.

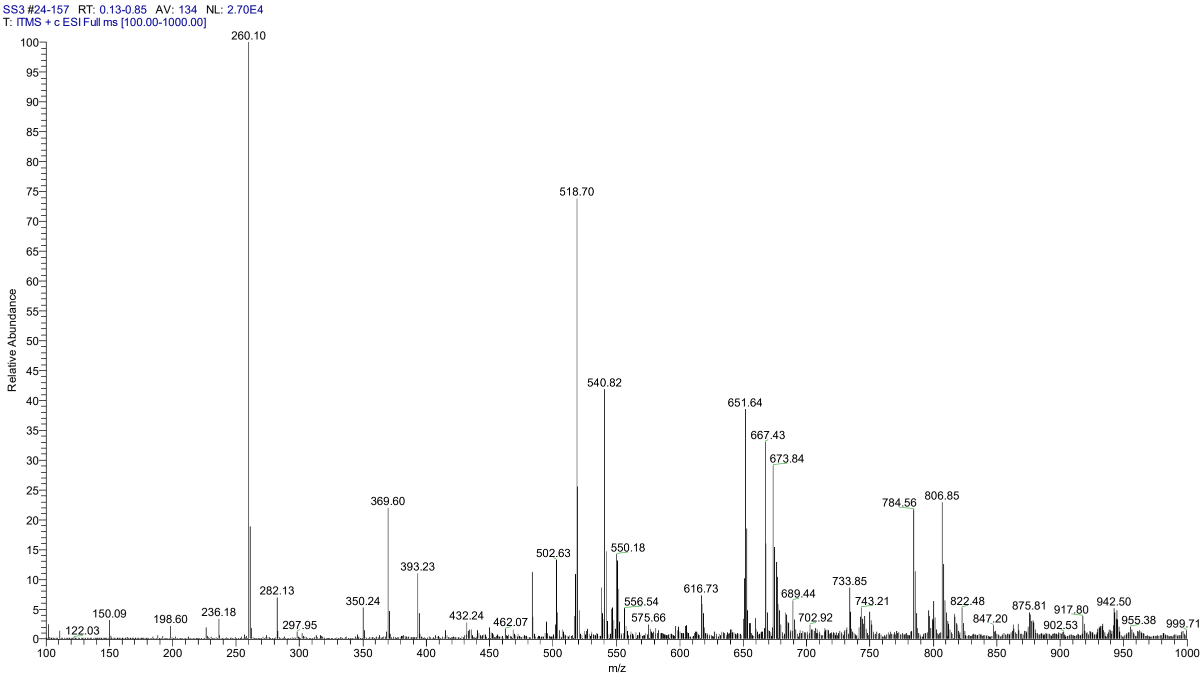

Figure S104. ESI-MS spectra of 1,4-bis(1-benzyl-1H-1,2,3-triazol-4-yl)benzene.

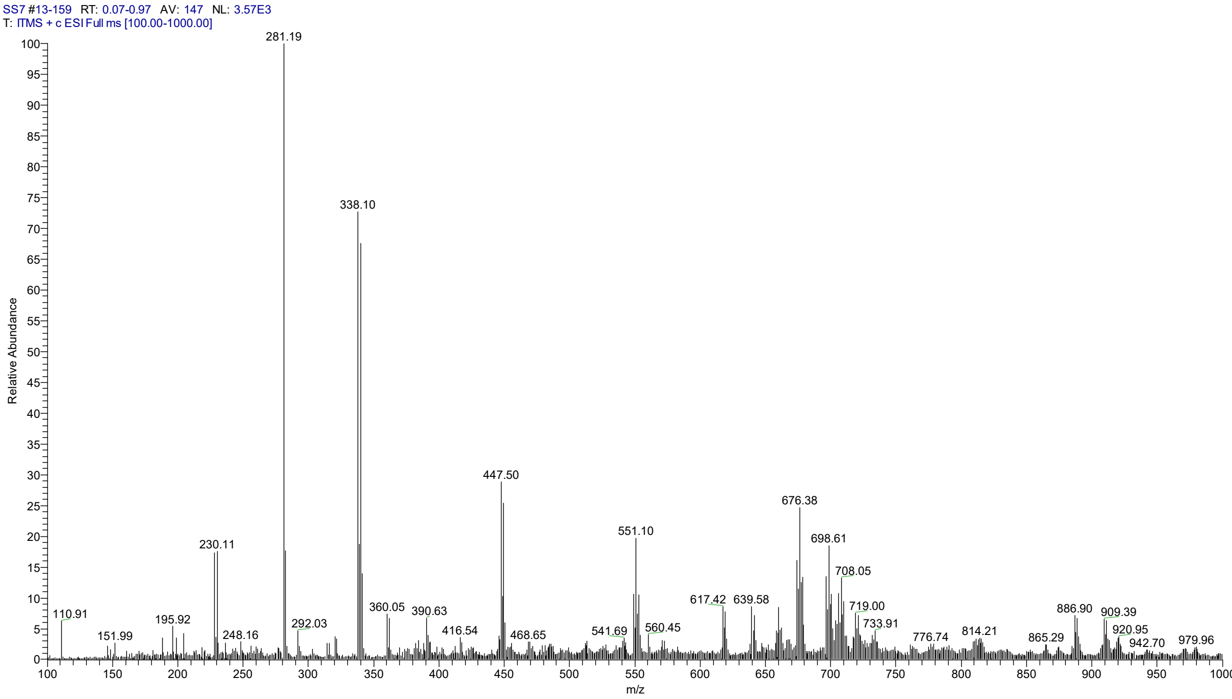

Figure S105. ESI-MS spectra of 1,4-bis(1-(3-bromobenzyl)-1H-1,2,3-triazol-4-yl)benzene.

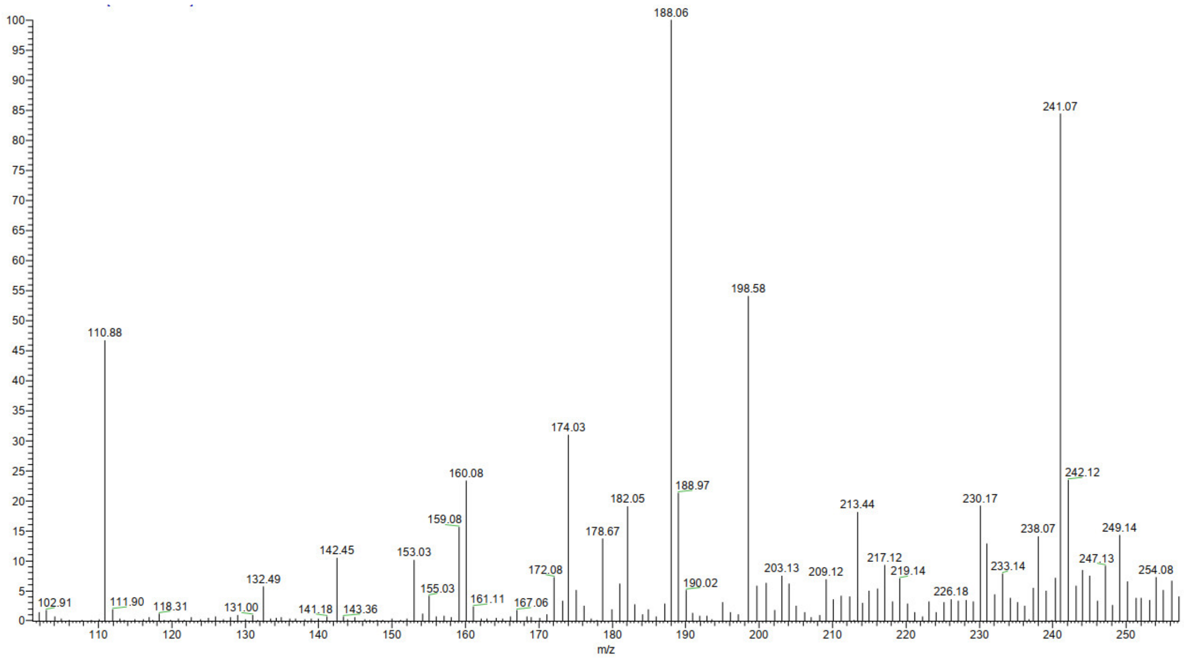

Figure S106. ESI-MS spectra of (4-(p-tolyl)-2H-1,2,3-triazol-2-yl)methanol.

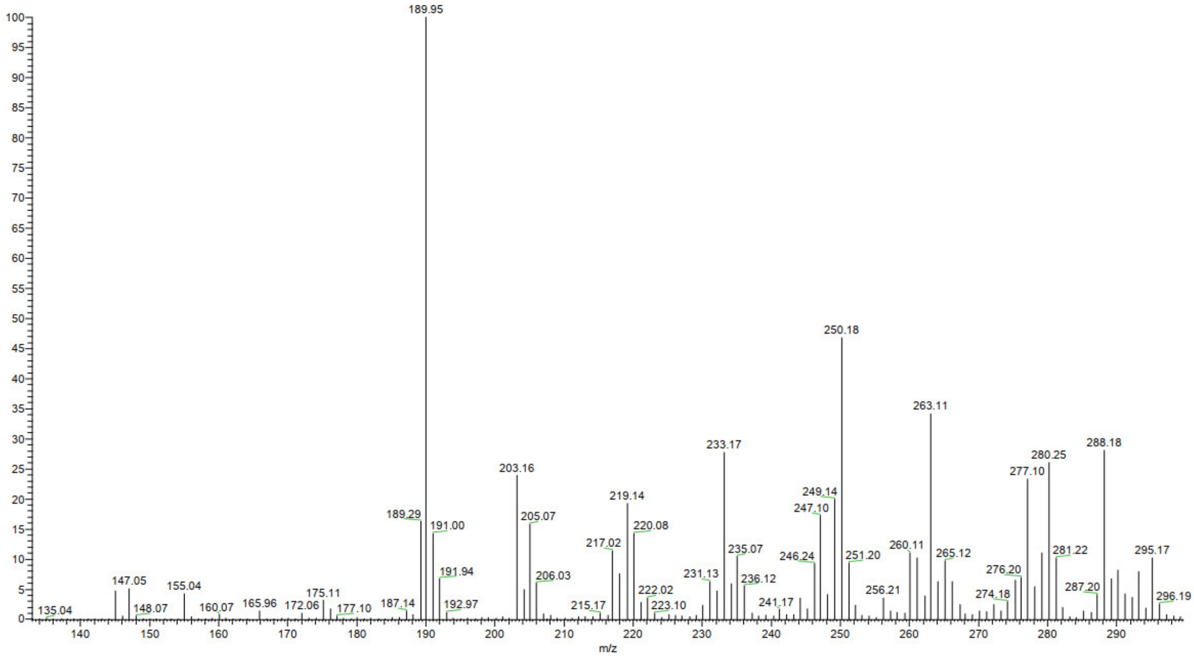

Figure S107. ESI-MS spectra of (4-(4-methoxyphenyl)-2H-1,2,3-triazol-2-yl)methanol.

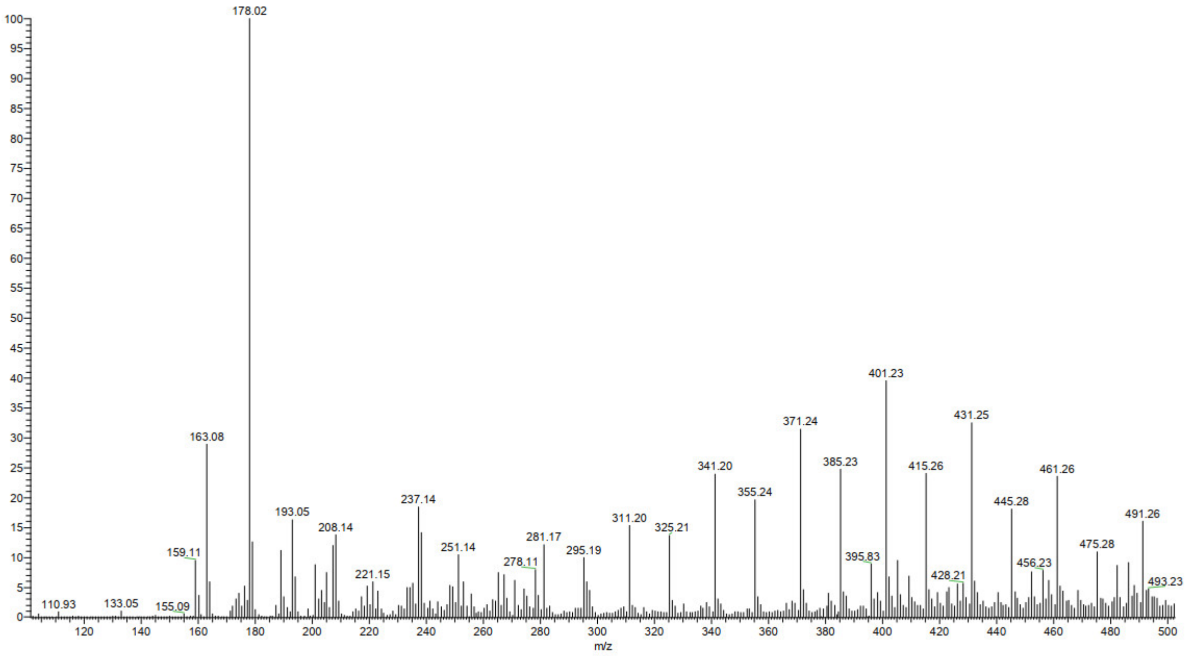

Figure S108. ESI-MS spectra of (4-(4-fluorophenyl)-2H-1,2,3-triazol-2-yl)methanol.

Table S2. Optimization parameters for **5b** complex as a homogeneous catalyst for the microwave-assisted synthesis of 1-benzyl-4-phenyl-1H-1,2,3-triazole [a].

Entry	Cat. Load[b] (mol%)	Time (min.)	Solvent	Total volume of solvent mixture (mL)	Yield[c] (%)	TON[d]
1	3	15	-	-	-	-
2	3	15	H_2O:MeCN	1.5	47.17	16
3	3	15	H_2O:MeCN	1.0	84.43	28
4	3	15	H_2O:MeCN	0.5	91.8	31
5	3	15	H_2O:DMF	1.0	51.14	17
6	3	15	H_2O:DMSO	1.0	56.1	19
7	3	15	H_2O:1,4-dioxane	1.0	71.23	24
8	3	60	H_2O:MeCN	1.0	88.54	30
9	3	60	H_2O:MeCN	0.5	93.21	31
10	3	60	H_2O:MeCN	0.25	89.81	30
11	3	60	H_2O:1,4-dioxane	0.5	84.01	28
12	3	60	H_2O:1,4-dioxane	1.0	94.21	31
13	5	15	H_2O:MeCN	1.5	76.50	15
14	5	15	H_2O:DMF	1.0	47.74	10
15	5	15	H_2O:DMSO	1.0	50.15	10
16	5	15	H_2O:1,4-dioxane	1.0	83.86	17
17	5	30	H_2O:MeCN	1.0	80.75	16
18	5	60	H_2O:MeCN	1.5	77.77	16
19	5	60	H_2O:MeCN	1.0	97.04	19
20	5	60	H_2O:MeCN	0.5	98.74	20
21	5	60	H_2O:1,4-dioxane	1	90.66	18

[a] Reaction conditions: benzyl bromide (0.3 mmol), phenylacetylene (0.3 mmol), NaN_3 (0.3 mmol), catalyst **5b**, solvent mixture H_2O/organic (1:1 v/v), MW irradiation (30 W), at 125 ^{o}C. [b] Isolated yield. [c] Turnover number (moles of product per moles of catalyst).

Table S3. Comparison of the present system with other reported C-supported catalysts for the synthesis of 1,4-disubstituted 1,2,3-triazoles.

Entry	Catalyst (loading)	Reaction Conditions	Yield (%)	Reference
1	Gerhardite [$Cu_2(NO_3)(OH)_3$] (Cu/C)	10 mol% of the catalyst, dioxane, (1 equiv.) Et_3N, 60 ^oC and 10-120 min	92-99	[72]
2	Cu(I)/GO	2 mol% of the catalyst at 40 ^oC for 48 h in inert conditions using deuterated THF	80-99	[73]
3	Cu(I)/RGO	8 mol % of the catalyst, THF,40 ^oC and 24 h	85-99	[55]
4	Cu/C NPs	0.5 mol% of the catalyst, H_2O, 70 ℃ and 4 h	78-98	[74]
5	Cu(I)-DAPTA complex/CNT-ox-Na	0.5 mol % of the catalyst, H_2O/MeCN, MW, 80 ℃, 15-60 min	32-48	[28]
6	Cu(I)-*N*-alkylated PTA complex (**5b**)/CNT-ox-Na	1.2 mol % of the catalyst, H_2O/MeCN (1:1), MW, 125 ℃, 60 min	29-80	This work
7	**5b** (Unsupported homogeneous system)	5 mol % of the catalyst, H_2O/MeCN (1:1), MW, 125 ℃, 60 min	99	This work

Abbreviations: Charcoal (C), graphene oxide (GO), reduced graphene oxide (RGO), nanoparticles (NPs), 3,7-diacetyl-1,3,7-triaza-5-phosphabicyclo[3.3.1]nonane (DAPTA) and multi-walled carbon nanotubes treated with nitric acid and NaOH (CNT-ox-Na).
